# Progression subtypes in Parkinson’s disease identified by a data-driven multi cohort analysis

**DOI:** 10.1038/s41531-024-00712-3

**Published:** 2024-05-02

**Authors:** Tom Hähnel, Tamara Raschka, Stefano Sapienza, Jochen Klucken, Enrico Glaab, Jean-Christophe Corvol, Björn H. Falkenburger, Holger Fröhlich

**Affiliations:** 1 Department of Bioinformatics, Fraunhofer Institute for Algorithms and Scientific Computing (SCAI), Sankt Augustin, Germany; 2 Department of Neurology, Medical Faculty and University Hospital Carl Gustav Carus, TUD Dresden University of Technology, Dresden, Germany; 3 Bonn-Aachen International Center for IT, University of Bonn, Bonn, Germany; 4 Biomedical Data Science, Luxembourg Centre for Systems Biomedicine (LCSB), University of Luxembourg, Esch-sur-Alzette, Luxembourg; 5 Luxembourg Institute of Health (LIH), Strassen, Luxembourg; 6 Centre Hospitalier de Luxembourg (CHL), Strassen, Luxembourg; 7 Sorbonne Université, Paris Brain Institute – ICM, Inserm, CNRS, Assistance Publique Hôpitaux de Paris, Pitié-Salpêtrière Hospital, Department of Neurology, Paris, France; 8 German Center for Neurodegenerative Diseases (DZNE), Dresden, Germany

**Keywords:** Parkinson's disease, Parkinson's disease, Clinical trials, Biomarkers

## Abstract

The progression of Parkinson’s disease (PD) is heterogeneous across patients, affecting counseling and inflating the number of patients needed to test potential neuroprotective treatments. Moreover, disease subtypes might require different therapies. This work uses a data-driven approach to investigate how observed heterogeneity in PD can be explained by the existence of distinct PD progression subtypes. To derive stable PD progression subtypes in an unbiased manner, we analyzed multimodal longitudinal data from three large PD cohorts and performed extensive cross-cohort validation. A latent time joint mixed-effects model (LTJMM) was used to align patients on a common disease timescale. Progression subtypes were identified by variational deep embedding with recurrence (VaDER). In each cohort, we identified a fast-progressing and a slow-progressing subtype, reflected by different patterns of motor and non-motor symptoms progression, survival rates, treatment response, features extracted from DaTSCAN imaging and digital gait assessments, education, and Alzheimer’s disease pathology. Progression subtypes could be predicted with ROC-AUC up to 0.79 for individual patients when a one-year observation period was used for model training. Simulations demonstrated that enriching clinical trials with fast-progressing patients based on these predictions can reduce the required cohort size by 43%. Our results show that heterogeneity in PD can be explained by two distinct subtypes of PD progression that are stable across cohorts. These subtypes align with the brain-first vs. body-first concept, which potentially provides a biological explanation for subtype differences. Our predictive models will enable clinical trials with significantly lower sample sizes by enriching fast-progressing patients.

## Introduction

Parkinson’s disease (PD) is the fastest-growing neurological disease and the second most common neurodegenerative disease^1^. Recent randomized clinical trials (RCTs) have investigated potentially disease-modifying treatments, but have failed to reach their primary endpoints^2–5^. This raises the question of whether our understanding of PD pathogenesis is insufficient or whether RCTs were inadequately designed to demonstrate treatment effects on disease progression. The high heterogeneity observed in people with PD (PwPD)^6^ limits the statistical power of clinical trials. Furthermore, the observed heterogeneity suggests the existence of PD subtypes which might show different treatment responses.

The construct of PD as a heterogeneous group of different subtypes has been proposed in several concepts. Some concepts categorize PwPD by single clinical features like age of onset, motor phenotype, or onset of dementia^7^. The brain-first vs. body-first concept explains heterogeneity observed in imaging data by different routes of alpha-synuclein spreading through the nervous system^8^. This model is further extended by the alpha-synuclein origin site and connectome (SOC) model which suggests that alpha-synuclein spreading from one brain hemisphere to the other is less common^9^. Other researchers identified subtypes using data-driven methods and machine learning^10–14^. These approaches have the advantages of being hypothesis-free and being able to capture more complex patterns from multivariate data. Subtypes were mostly inferred based on cross-sectional differences^10^, but some researchers also investigated differences in disease progression using longitudinal data from single cohorts^11,12^.

Our study aims to identify PD subtypes with a focus on differences in disease *progression*, inferred from multimodal longitudinal cohort data. We extensively characterized PD subtypes regarding differences in motor and non-motor symptom progression, demographic factors, mortality, treatment response, DaTSCAN imaging and digital gait biomarkers, comorbidities, co-medications, blood markers, and cerebrospinal fluid markers. In particular, we investigated the generalizability of our findings by external validation in additional and highly diverse cohorts. Further, we developed a strategy to enrich for PwPD of one subtype within a study cohort. We then analyzed how this enrichment reduces the required sample size and increases the statistical power of clinical trials.

## Results

### Demographic and clinical characteristics

Overall, 1,124 PwPD from three cohorts were analyzed. In general, the cohorts exhibited different clinical characteristics related to disease duration at baseline: LuxPARK included advanced disease stages compared to ICEBERG and PPMI with mostly early disease stages (Table 1). Significant differences across cohorts were observed for age, disease duration, Hoehn & Yahr stage, UPDRS I-IV, PIGD, MoCA, and SCOPA.

**Table 1:** Demographic and clinical characteristics

	PPMI	ICEBERG	LuxPARK	PPMI vs ICEBERG *p*-values	PPMI vs LuxPARK *p*-values	ICEBERG vs LuxPARK *p*-values
**Number of PwPD**	409	154	561	··	··	··
**Age, years**	63.0 [55.2–69.3]	63.7 [57.1–69.4]	67.8 [59.4–73.1]	1.0	<0.0001	<0.0001
**Sex**	··	··	··	0.71	1.0	0.49
Male	66.7% [273]	62.3% [96]	67.6% [379]	··	··	··
Female	33.3% [136]	37.7% [58]	32.4% [182]	··	··	··
**Hoehn & Yahr**	··	··	··	<0.0001	<0.0001	0.0076
H&Y I	43.8% [179]	2.6% [4]	18.7% [105]	··	··	··
H&Y II	56.2% [230]	93.5% [144]	67.0% [376]	··	··	··
H&Y III	0	3.9% [6]	9.1% [51]	··	··	··
H&Y IV	0	0	3.7% [21]	··	··	··
H&Y V	0	0	1.4% [8]	··	··	··
**Disease duration, years**	0.3 [0.2–0.6]	1.2 [0.6–2.3]	2.9 [0.9–6.5]	<0.0001	<0.0001	<0.0001
**Number of visits**	14 [12–16]	5 [4,5]	4 [3–6]	<0.0001	<0.0001	0.13
**Follow up, years**	7.0 [5.0–7.0]	4.1 [3.0–4.4]	4.0 [2.4–5.0]	<0.0001	<0.0001	1.0
**UPDRS I**	5 [3–7]	9 [6–12]	9 [5–13]	<0.0001	<0.0001	1.0
**UPDRS II**	5 [3–8]	8 [5–10]	10 [5–15]	<0.0001	<0.0001	0.002
**UPDRS III**	20 [14–26]	29 [24–35]	32 [22–44]	<0.0001	<0.0001	0.047
**UPDRS IV**	..	0 [0-0]	0 [0-1]	··	··	<0.0001
**PIGD**	0.2 [0.0–0.4]	0.2 [0.0–0.4]	0.4 [0.2–1.0]	0.26	<0.0001	<0.0001
**MoCA**	28 [26–29]	28 [26–29]	25 [23–28]	0.11	<0.0001	<0.0001
**SCOPA**	8 [6–12]	11 [7–17]	14 [9–20]	0.00034	<0.0001	0.0015

PwPD baseline characteristics and study characteristics for PPMI, ICEBERG, and LuxPARK cohort. For sex and H&Y, relative and absolute frequencies are shown. For other characteristics, median and first/third quartiles are reported. Corresponding *p*-values were corrected for multiple testing. UPDRS IV was not assessed at baseline in PPMI. Significant *p*-values are emphasized in italics.

*H&Y* Hoehn & Yahr, *MoCA* Montreal Cognitive Assessment, *PIGD* Postural Instability, and Gait Dysfunction score, *SCOPA* Scales for Outcomes in Parkinson’s Disease-Autonomic Dysfunction, *UPDRS* Unified Parkinson’s Disease Rating Scale.

### Identification of PD progression subtypes

**Fig. 1:**
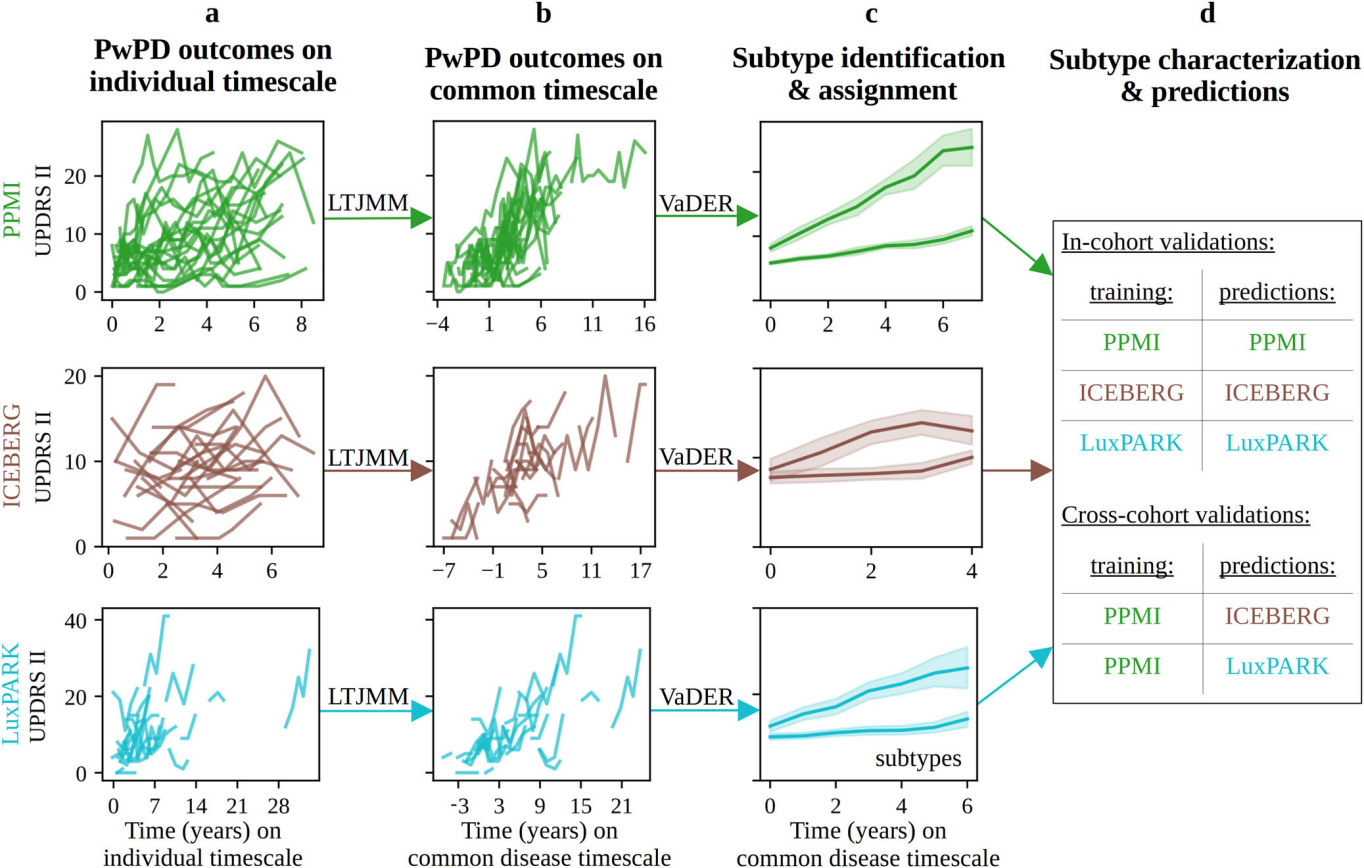
Model training and validation procedure for subtype identification and predictions. Individual PwPD outcomes (**a**) were aligned on a common timescale (**b**) using a latent time joint mixed-effects model (LTJMM). The UPDRS II values of 25 randomly sampled PwPD are shown for visualization. Subsequently, two distinct progression subtypes were identified (**c**) using a variational deep embedding with recurrence (VaDER). Subtypes were further characterized and models were trained to predict subtype associations from baseline (**d**). VaDER and predictive models were trained and evaluated on each cohort separately and results were compared across cohorts (in-cohort validation). Additionally, PPMI-trained models were applied to ICEBERG and LuxPARK and results were compared with results of the in-cohort approach (cross-cohort validation). UPDRS Unified Parkinson’s Disease Rating Scale.

First, we aligned PwPD to a common disease timescale by applying a latent time joint mixed-effects model (LTJMM) to the longitudinal data of the PPMI, ICEBERG and LuxPARK cohorts (Fig. 1a/b, Supplementary Fig. 1). Next, we identified distinct PD progression subtypes using variational deep embedding with recurrence (VaDER) and assigned each PwPD to one of these subtypes (Fig. 1c). Finally, we compared different approaches for predicting PD progression subtypes using baseline data or baseline data with a short follow up (Fig. 1d).

We repeated these steps in a cross-cohort validation fashion to explore the generalizability of our approach. This was done by training our model on the publicly available PPMI data and using this model for PwPD subtype assignments and predictions in ICEBERG and LuxPARK.

### PD subtypes exhibit different symptom characteristics

**Fig. 2:**
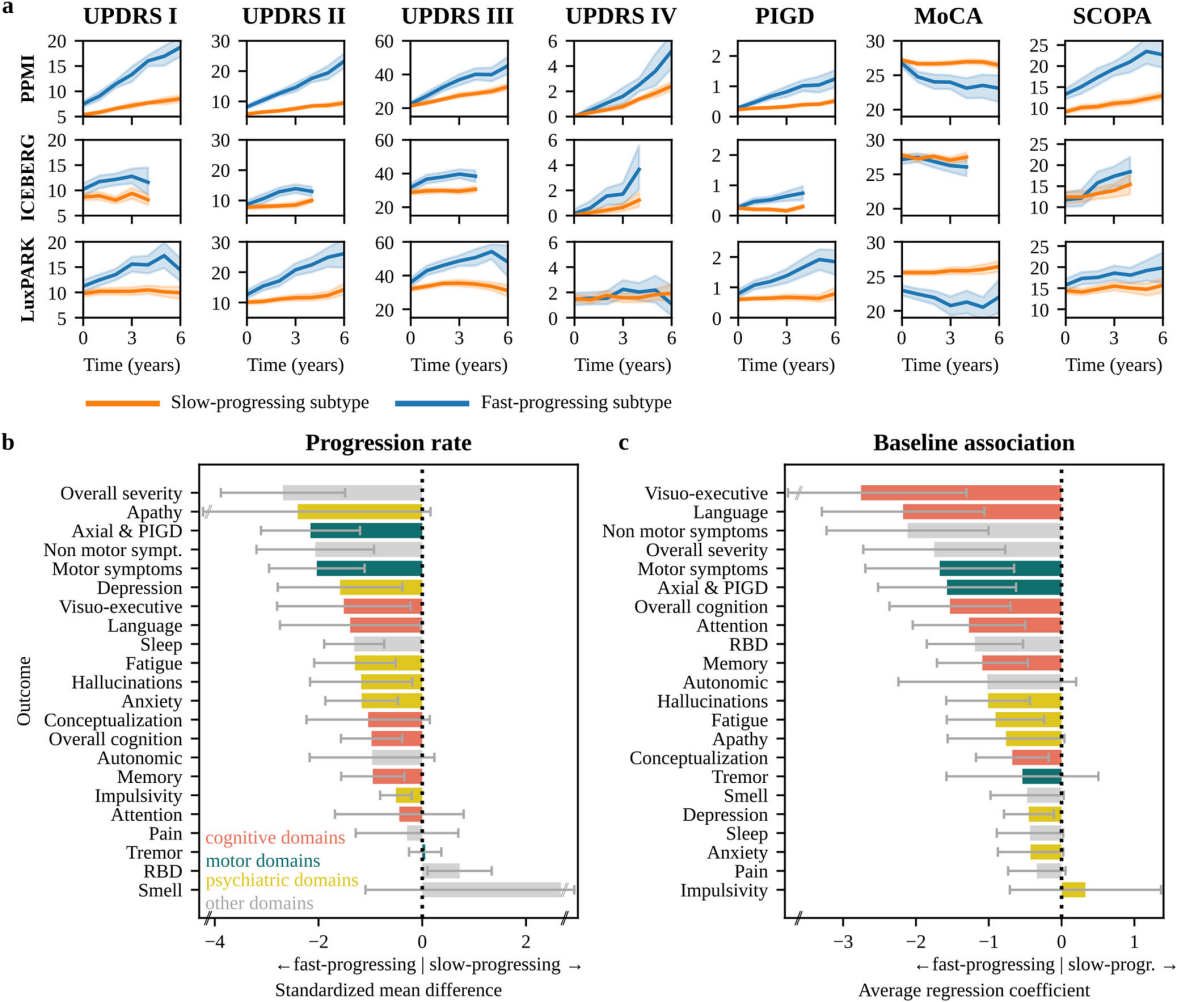
Disease progression and baseline characteristics of subtypes. **a** Progression of motor scores (UPDRS II/III/IV, PIGD) and non-motor scores (UPDRS I, MoCA, SCOPA) for the slow-progressing subtype (orange) and fast-progressing subtype (blue) for PPMI, ICEBERG, and LuxPARK. Mean and 95% confidence intervals for each subtype are shown. ICEBERG data is shown up to four years as only a few ICEBERG PwPD had longer follow up. **b** Standardized mean differences (SMD) of progression speed between both subtypes for different symptom domains (orange: cognition, green: motor, yellow: psychiatric, gray: other). Negative SMD values indicate that the fast-progressing subtype shows a faster progression. **c** Average regression coefficients showing associations of symptom domains at baseline with subtypes. Negative values indicate that more severe symptoms at baseline are associated with the faster subtype. 95% confidence intervals are shown and were corrected for multiple testing. MoCA Montreal Cognitive Assessment, PIGD Postural Instability and Gait Dysfunction score, SCOPA Scales for Outcomes in Parkinson’s Disease-Autonomic Dysfunction, RBD REM behavior sleep disorder, UPDRS Unified Parkinson’s Disease Rating Scale.

Following the approach outlined in Fig. 1, we identified two subtypes for each of the three cohorts. Subsequently, we explored baseline and progression characteristics of clinical symptoms between these two PD subtypes. Focusing on the motor (UPDRS II/III/IV, PIGD) and non-motor (UPDRS I, MoCA, SCOPA) outcomes assessed in all three cohorts, we observed minor differences at baseline but large differences in progression speed. One subtype exhibited significantly faster progression for all symptoms and thereby was named *fast-progressing* subtype compared to the second *slow-progressing* subtype (Fig. 2a). Most PwPD were assigned to the slow-progressing subtype (PPMI: 335 slow/74 fast, ICEBERG: 112 slow/42 fast, LuxPARK: 408 slow/153 fast). While mean progression trajectories were clearly separated for most outcomes, we observed some overlap in ICEBERG for autonomic symptoms reported by SCOPA and also for cognition reported by MoCA. However, ICEBERG is the smallest cohort, and PwPD in ICEBERG presents with only minimal cognitive impairment at baseline. Also, the trend to more rapid progression in the fast-progressing subtype was still similar to PPMI and LuxPARK. We also observed some overlap of subtypes in terms of motor fluctuations reported by UPDRS IV in the LuxPARK cohort while there was a better separation for PPMI and ICEBERG.

Both progression subtypes showed similar sex distributions and disease durations (Supplementary Table 1). Fast-progressing PwPD had a higher median age in PPMI (67.6 vs. 62.0 years, *p* < 0.0001) and LuxPARK (70.1 vs 66.8 years, *p* < 0.0001). Similarly, fast-progressing PwPD had higher median disease onset in PPMI (67.1 vs. 61.4 years, *p* < 0.0001) and LuxPARK (67.0 vs 60.5 years, *p* < 0.0001). Fast-progressing PwPD in ICEBERG also had a higher age at baseline and age at PD diagnosis than slow-progressing PwPD, but the differences were not significant (Supplementary Table 1). These findings are in line with previous research indicating a slower progression and better prognosis in people with young PD onset^13^.

The results shown in Fig. 2a comprise only a small subset of symptom domains affected in PD. We were wondering whether all symptoms progress more rapidly in the fast-progressing subtype, or whether the pattern of progression rates differs between the two subtypes. Therefore, we aggregated single questions, sub-scores, and total scores from different assessments into 22 distinct symptom domains (Supplementary Table 2). Indeed, overall disease severity, axial and PIGD symptoms progressed much more rapidly in the fast-progressing subtype as compared to the slow-progressing subtype (Fig. 2b). In contrast, the rate of progression for most cognitive domains differed less between both subtypes. Interestingly, there was no difference in tremor progression between subtypes, and the slow-progressing subtype exhibited faster progression of REM behavior sleep disorder (RBD) symptoms than the fast-progressing subtype.

To examine subtype differences at baseline, we analyzed the statistical association of baseline outcomes with the progression subtypes after correcting for differences in disease duration on the common disease timescale (Fig. 2c). Overall, cognitive domains exhibited more pronounced differences than other domains at baseline (Fig. 2c). Fast-progressing PwPD exhibited already higher RBD values at baseline, thereby providing an explanation for the slower RBD progression observed in the fast-progressing subtype. Similar to progression characteristics, tremor had no significant baseline association with subtypes. Visuo-executive function and language function exhibited the largest baseline and progression differences between both subtypes compared to the other cognitive domains.

Progression characteristics and baseline characteristics were mostly similar between the three cohorts and reproducible in the cross-cohort validation (Supplementary Fig. 2-4), thereby supporting generalizability of these findings. In addition, we compared baseline outcomes directly, i.e., without a correction for differences in disease duration (Supplementary Fig. 5). The results of this analysis were mostly similar to Fig. 2c.

### Mortality and treatment response

**Fig. 3:**
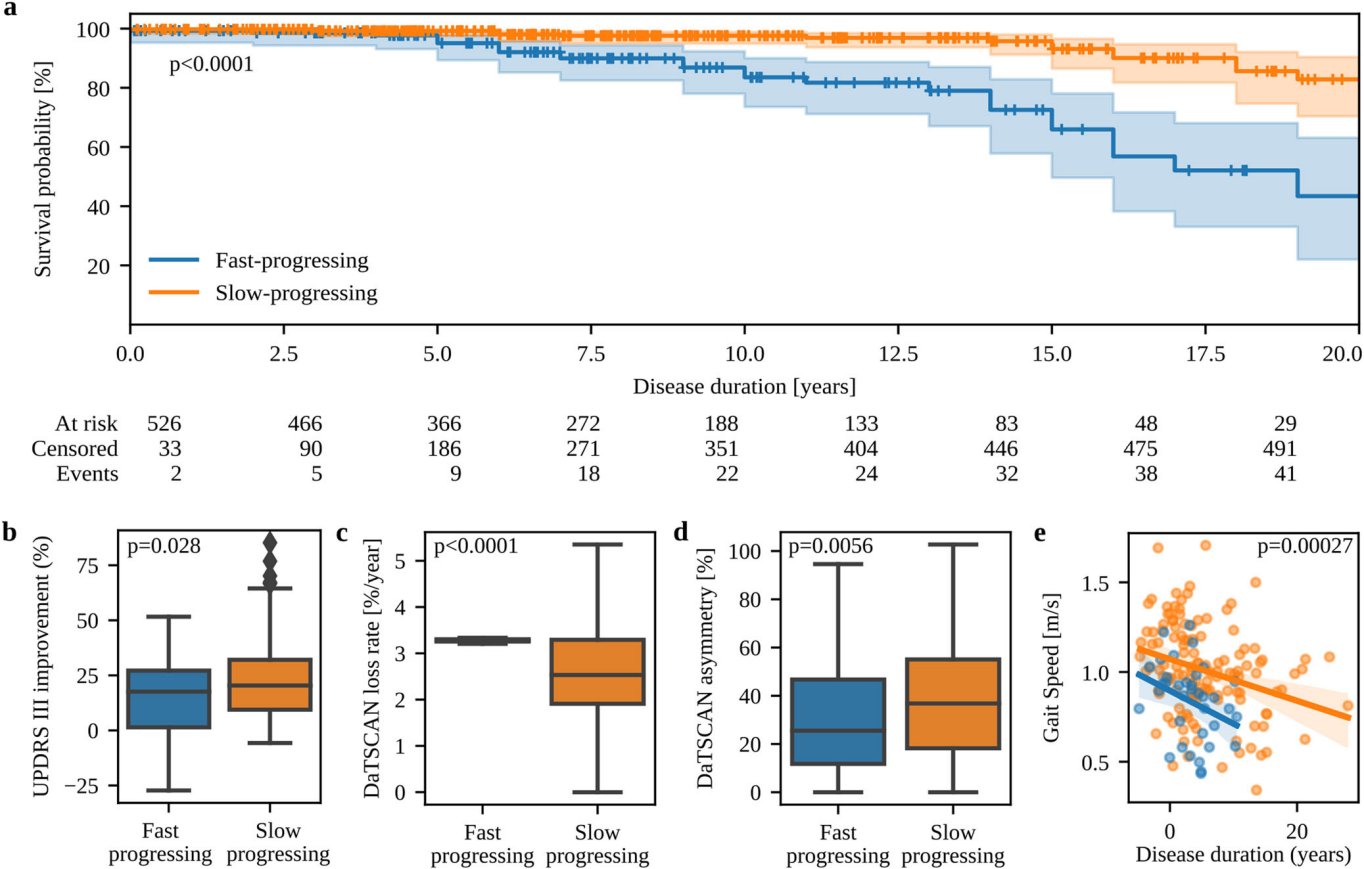
Mortality, treatment response and biomarker differences between subtypes. **a** Kaplan-Meier estimator for survival probability on the common disease timescale for fast-progressing (blue) and slow-progressing (orange) PwPD in LuxPARK. Censored observations are indicated by small vertical ticks. The corresponding p-value for the subtype covariate from the cox proportional hazard model is reported. 95% confidence intervals are shown. **b** Mean UPDRS III improvement of PwPD in PPMI after dopaminergic drug intake compared to OFF state. **c** Progression of DaTSCAN uptake loss for fast-progressing and slow-progressing progressing PwPD. **d** DaTSCAN asymmetry index at baseline for slow-progressing and fast-progressing PwPD. **e** Correlation of gait speed with disease duration on the common timescale for fast-progressing (blue) and slow-progressing (orange) PwPD. Only the most significant digital gait parameter is shown here while correlations of all gait parameters are presented in the supplement. The corresponding p-value from the ANCOVA analysis is shown and was corrected for multiple testing of all digital gait parameters. 95% confidence intervals are shown. The boxplots are displayed with a median line, box borders representing the interquartile range (IQR), whiskers extending to 1.5 times the IQR, and outliers depicted as diamonds beyond the whiskers. Abbreviations: UPDRS: Unified Parkinson’s Disease Rating Scale.

Mortality data was only available for LuxPARK and showed an increased hazard ratio (HR) for death for the fast-progressing subtype (HR = 3.4, 95% CI: 1.9 – 6.2, *p* < 0.0001, Fig. 3a). Similar findings were obtained in the cross-cohort validation approach (HR = 3.7, 95% CI: 1.9–7.1, *p* < 0.0001, Supplementary Fig. 6).

Treatment responses were available for PPMI and indicated a worse response to dopaminergic treatment for fast-progressing PwPD (*p* = 0.028, Fig. 3b).

### Imaging and gait biomarkers

In addition to clinical outcomes, we investigated whether subtype differences were also reflected by biomarkers. DaTSCAN was performed for *n* = 392 PwPD at baseline in PPMI while longitudinal DaTSCAN measurements were available for *n* = 367 PwPD in PPMI. Baseline DaTSCAN imaging showed no subtype difference (*p* = 0.37), but DaTSCAN progression differed significantly (*p* < 0.0001, Fig. 3c). DaTSCAN asymmetry was more pronounced for the slow-progressing subtype at baseline (*p* = 0.0056, Fig. 3d). Over time, differences in DaTSCAN asymmetry between subtypes narrowed, as DaTSCAN asymmetry increased for fast-progressing PwPD (+ 1.1%/year) and decreased for slow-progressing PwPD (−1.0%/year) with high significance between subtypes (p < 0.0001).

Digital gait assessments were performed for *n* = 177 PwPD in LuxPARK at a single visit. We analyzed 15 digital gait parameters (Supplementary Table 3), of which seven exhibited significant differences between subtypes. Specifically, the fast-progressing subtype expressed lower gait speed (*p* = 0.00027, Fig. 3e), shorter stride length (*p* = 0.00027), a larger toe-off angle (*p* = 0.002), lower toe clearance (*p* = 0.006), shorter relative swing time (*p* = 0.013), higher relative stance time (*p* = 0.013) and a lower heel clearance (*p* = 0.027). Correlations of all digital gait parameters are presented in the supplement (Supplementary Fig. 7). Furthermore, gait speed (*p* = 0.0055), stride length (*p* = 0.0055), and toe-off angle (*p* = 0.0055) remained also significant in the cross-cohort validation using the PPMI-trained model (Supplementary Fig. 8).

The correlation structure of the digital gait parameters was assessed through an exploratory factor analysis. This analysis revealed that three factors accounted for 69% of the gait parameter variance (Supplementary Figs. 9 and 10A). Most gait parameters were highly correlated. (Supplementary Fig. 10B).

### External factors influencing PD progression subtypes

We investigated the association of several external factors, that are not specific for PD, but were discussed as potentially related to PD diagnosis or PD progression, such as education, PD family history, comorbidities, and comedications^15,16^. The analysis was conducted using PPMI data, which only included de novo PwPD to minimize the risk of reverse causation, i.e., differences in PD severity leading to differences in these external factors. Additionally, education was evaluated in ICEBERG and LuxPARK as it is clearly related to the time before PD diagnosis.

We identified an association of higher education with the slow-progressing subtype for PPMI (*p* = 0.022) and ICEBERG (*p* = 0.019). In LuxPARK, we still observed a higher level of education in slow-progressing PwPD, but the difference was not statistically significant (*p* = 0.13, Supplementary Fig. 11).

Interestingly, higher weight (*p* = 0.049) and height (*p* = 0.012) were associated with the fast-progressing subtype, whereas body mass index showed no association (*p* = 0.46, Supplementary Fig. 12). Family history was not associated with any particular progression subtype (*p* = 0.15). Neither systolic blood pressure, diastolic blood pressure, nor the drop in blood pressure after standing up were associated with one of the progression subtypes (Supplementary Table 4).

Moreover, we investigated several blood markers that have been discussed in the context of PD risk or PD progression rate. We found no significant association of serum glucose (*p* = 0.92), serum uric acid (p = 0.92), high density lipoprotein (*p* = 0.92), low-density lipoprotein (*p* = 0.92), or total cholesterol (*p* = 0.92) with the progression subtypes.

Additionally, no significant association was found between progression subtypes and several comorbidities: hypertension (*p* = 0.86), diabetes (*p* = 0.92), hypercholesterolemia (*p* = 0.92), gout (*p* = 0.92), cancer (*p* = 0.92) and appendectomy (*p* = 0.92). The analysis was repeated for 15 disease groups, such as pulmonary or cardiovascular diseases, but no significant association with PD progression subtypes was found (Supplementary Table 5).

Specifically, we examined the correlation of cerebrospinal fluid markers of Alzheimer’s disease with progression subtypes. Thereby, we found a significantly lower amyloid beta 1-42 (*p* = 0.043) and a significantly higher p-tau/amyloid beta 1-42 ratio (*p* = 0.00065) for fast-progressing PwPD. In contrast, there was no significant difference in p-tau levels (*p* = 0.18, Supplementary Fig. 13).

Examining comedication, we analyzed the association of NSARs (all NSARs, ASS only, all NSARs except ASS), beta agonists, beta antagonists, calcium channel blockers, statins, and contraceptives with progression subtypes. However, we did not find any significant associations (Supplementary Table 6).

All analyses conducted in this section were corrected for age and sex. P-values have been corrected for multiple testing.

### Enriching clinical trials with fast-progressing PwPD

**Fig. 4:**
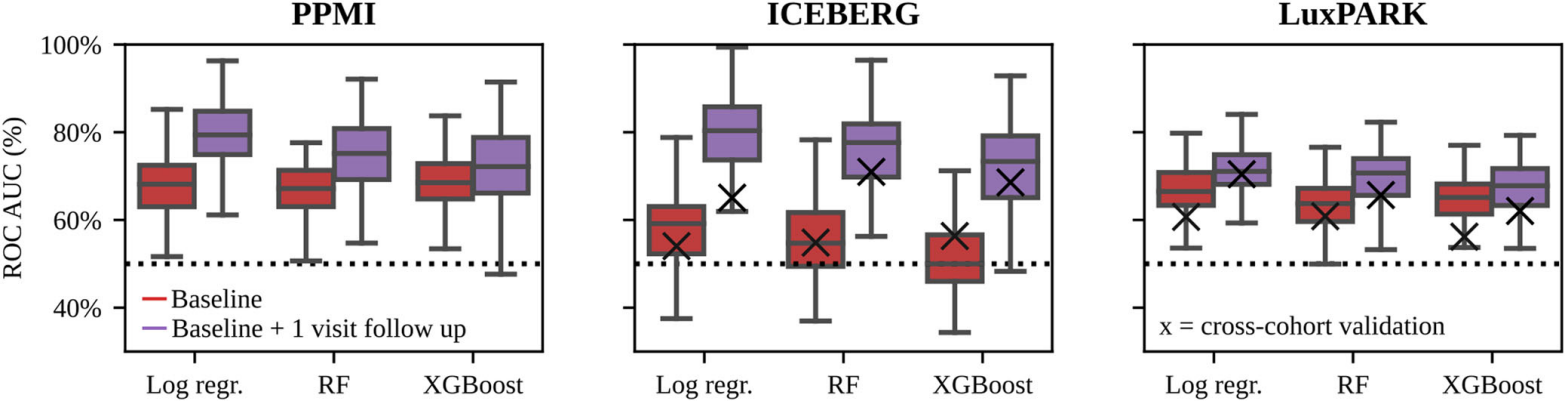
Evaluation of subtype predictions using different machine learning models. Subtypes of individual PwPD were predicted from baseline data (red) or baseline data with one follow-up visit (purple) using three different predictive models (Logistic Regression, Random Forest, XGBoost). Models were trained using repeated nested cross-fold validation. ROC-AUC of the subtype predictions is shown for PPMI, ICEBERG, and LuxPARK. Additionally, cross-cohort validation was performed using the PPMI-trained model for ICEBERG and LuxPARK predictions (black cross for ICEBERG and LuxPARK figures). The boxplots are displayed with a median line, box borders representing the interquartile range (IQR), whiskers extending to 1.5 times the IQR, and outliers depicted as diamonds beyond the whiskers. Log Regr Logistic Regression, RF Random Forrest, ROC-AUC receiver operating characteristics-area under the curve, XGBoost eXtreme Gradient Boosting.

Finally, we assessed the feasibility of using PD subtypes for stratification in clinical trials based on machine learning subtype predictions. PD subtypes could be predicted from baseline data with ROC-AUC up to 68% for PPMI, 58% for ICEBERG, and 67% for LuxPARK using nested cross-validation (Fig. 4, Supplementary Fig. 14). Including data from one additional follow-up visit for predictions, ROC-AUCs increased to 79% for PPMI, 79% for ICEBERG and 67% for LuxPARK. Cross-cohort validation resulted in ROC-AUCs close to the chance level: 56% for ICEBERG and 61% for LuxPARK using baseline data. However, the inclusion of only one follow-up visit increased the cross-cohort ROC-AUCs to 71% for ICEBERG and 70% for LuxPARK. Altogether this demonstrates that a short follow-up period opens the possibility to make reliable predictions about PD progression subtypes.

**Fig. 5:**
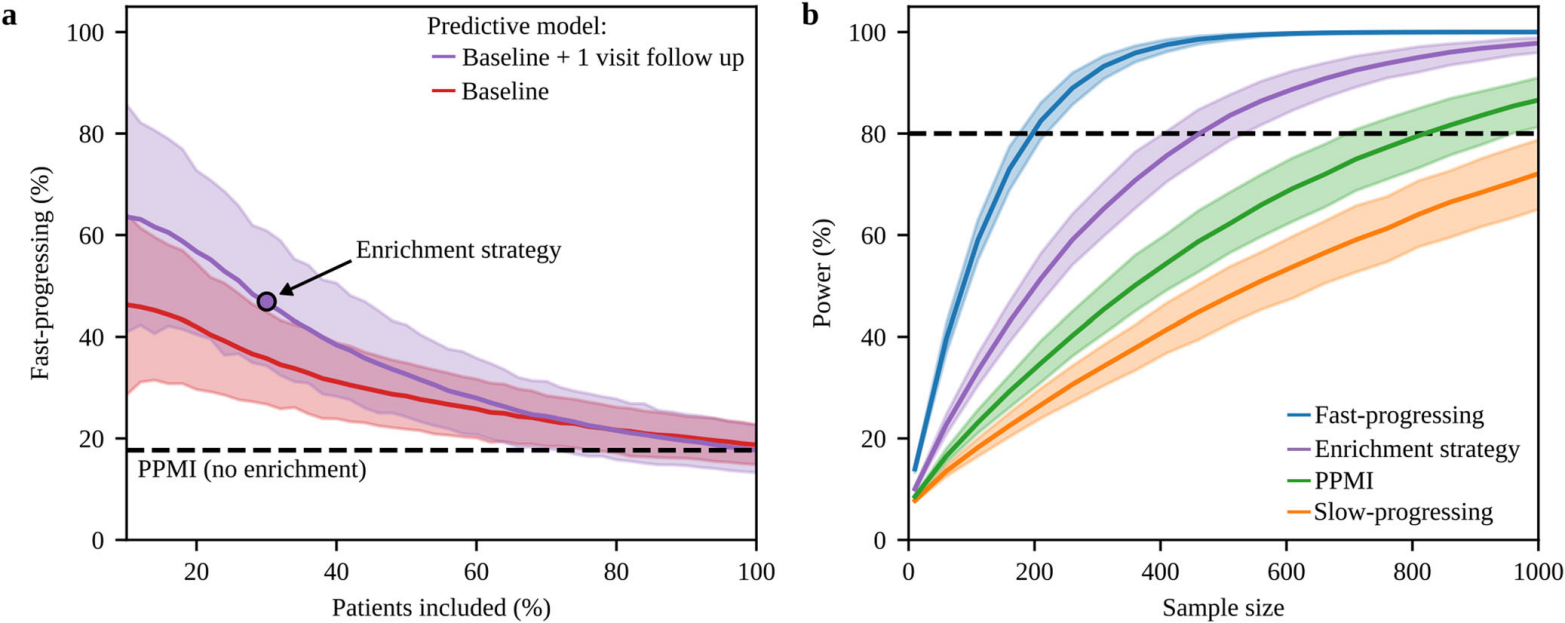
Subtype enrichment for sample size reduction in clinical trials. **a** Probabilities for PwPD in PPMI of belonging to the fast-progressing subtype predicted from baseline (red) and baseline with one follow-up visit (purple) were calculated using the logistic regression model. The figure depicts the percentage of fast-progressing PwPD and the number of PwPD which would be still eligible for study inclusion depending on the threshold applied to the predicted probabilities. The black dashed line indicates the percentage of fast-progressing PwPD observed in the complete PPMI cohort. When using the predictions from baseline + 1 visit follow-up data, 47% enrichment can be achieved with still 30% of PwPD being eligible for study inclusion (purple circle). **b** Estimated power and sample sizes required for a clinical trial depending on the percentage of fast-progressing PwPD, assuming the same treatment effect on disease progression for both subtypes: a theoretical cohort of only fast-progressing PwPD (blue), enrichment strategy presented in A (purple), default PPMI cohort without enrichment (green), the theoretical cohort of only slow-progressing PwPD (orange). A treatment effect of 30% on the progression rate of UPDRS I-III, one-year observation period and significance level α = 0.1 were assumed. The dashed black line indicates 80% power. 95% confidence intervals are shown for both sub-figures.

These predictive models can be used to enroll PwPD in a clinical study with a high predicted probability for the fast-progressing subtype. Thereby, there is a trade-off between the desired percentage of fast-progressing PwPD in the study and the number PwPD being still eligible for study inclusion. If a high percentage of fast-progressing PwPD is desired, fewer PwPD will be eligible for the study (Supplementary Fig. 15). Furthermore, the achievable enrichment level of fast-progressing PwPD depends on the performance of the predictive model. Without enrichment, 18% of PwPD in PPMI, 27% of PwPD in ICEBERG, and 26% of PwPD in LuxPARK belonged to the fast-progressing subtype. Using our predictive models that include baseline data only, a percentage of 36% (PPMI), 43% (ICEBERG), and 47% (LuxPARK) fast-progressing PwPD in a study cohort could be achieved by still allowing the inclusion of 30% of all PwPD. Using our predictive models with one year of follow-up, the fractions of fast-progressing PwPD increased to 47% (PPMI, Fig. 5a), 65% (ICEBERG) and 53% (LuxPARK).

We repeated these steps as a cross-cohort validation using the predictive model trained on PPMI to enrich fast-progressing PwPD in ICEBERG and LuxPARK. Using the predictive model including only baseline data, we observed no enrichment for ICEBERG and a small enrichment of 38% for LuxPARK. This is consistent with the baseline cross-cohort validation of the predictive models described above, which showed ROC-AUC values close to the chance level for ICEBERG and LuxPARK. After including also one year follow-up data in the predictive model, the fractions of fast-progressing PwPD increased to 38% (ICEBERG) and 41% (LuxPARK) (Supplementary Table 7).

Finally, we simulated an RCT inspired by a currently ongoing trial^17^. Applying our enrichment strategy to PPMI, we observed a sample size reduction of 30% using baseline data only and a 43% sample size reduction using one-year follow-up for predictions. A – theoretical – cohort of only fast-progressing PwPD would reduce the sample size by even 76% (Fig. 5b). Thereby, using the UPDRS I-III sum as primary study outcome resulted in the lowest required sample size along all analyzed outcomes with our enrichment strategy reducing required sample sizes for many clinical scores (Supplementary Fig. 16).

For ICEBERG and LuxPARK, we observed broadly similar sample size reductions ranging from 32% up to 56% (Supplementary Table 8). We then repeated these steps as a cross-cohort validation using the predictive model trained on PPMI to enrich fast-progressing PwPD in ICEBERG and LuxPARK. Using only baseline data, we observed no sample size reduction for ICEBERG and a sample size reduction of 28% for LuxPARK. After including also one year of follow-up data in the predictive model, the sample size reduction increased to 36% (ICEBERG) and 34% (LuxPARK, Supplementary Table 8).

## Discussion

In this study, we identified subtypes of PD *progression* and demonstrated generalizability in multiple external cohorts by using a combination of LTJMM and VaDER. This approach offers several advantages over traditional methods and captures heterogeneity in PD at several levels. By estimating random effects in LTJMM, we were able to account for individual differences in progression rates and baseline levels of each outcome. Sex- and age-specific covariates were integrated for each outcome in LTJMM to account for potential differences in the influence of these covariates on motor and non-motor symptoms. By aligning PwPD on a common disease timescale, we accommodated heterogeneity and uncertainty in diagnosis time and prevented an important bias in the data. For example, fast-progressing PwPD or PwPD with tremor as a diagnosis-leading symptom are likely to be diagnosed earlier. In this situation, the bias in diagnosis time would be corrected automatically by shifting the PwPD to an earlier disease time on the common disease timescale. Thereby, we include non-motor symptoms for time-aligning to reflect that neurodegeneration does not systematically start in the substantia nigra^18^ and non-motor symptoms precede motor symptoms in PD^7^. Subsequently, the VaDER model allowed for nonlinear interactions between outcomes for subtype identification.

By using this approach, we were able to demonstrate the high generalizability of our progression subtypes across heterogeneous cohorts with significant differences, e.g. differences in disease stages, disease severity, age, and PD diagnosis criteria. Benefits in disease progression modeling using such a temporal synchronization technique have recently been demonstrated for other neurodegenerative diseases^19,20^.

Recent research on PD progression has identified varying numbers of subtypes, i.e., two^21–24^, three^11–14,21,25,26^, or even four^27,28^ distinct subtypes. A notable challenge in this field that leads to ambiguity is the lack of a standardized method for determining the optimal number of PD subtypes. Various criteria such as Hartigan’s rule^12,26^, the Calinski-Harabasz pseudo-F value^25,27^, a priori decisions based on clinical interpretability^22^, Bayesian Information Criterion (BIC)^13,14^, cross-validation information criterion^24^, and clustering silhouettes^23^ have been employed, yet no consensus exists. Our approach, aligning with the methodologies of de Jong and Birkenbihl^11,29^, utilizes prediction strength to select a model with a minimal number of clusters that at the same time effectively captures the heterogeneity observed in PD. This choice does not negate the potential existence of more subtypes but emphasizes a model that simplifies the complexity inherent in PD. Additionally, the determination of subtypes is influenced not only by the chosen decision rule but also by the specific data utilized.

Our study focuses on clinical subtyping of disease progression, thereby differing from most previous research based on cross-sectional clinical data^13,22,25,26^. Other researchers have also demonstrated the identification of PD subtypes using neurodegeneration data^24^, genetics^28^, RT-QuiC kinetics^23^, or external PD risk factors^27^. Our model provides new insights showing that subtypes differ in aspects such as motor and non-motor symptoms, DaTSCAN results, treatment response, survival rates, digital gait assessments, education and Alzheimer’s disease pathology. Thereby, we could validate our findings across multiple cohorts, underscoring its robustness and applicability in understanding PD progression.

Interestingly, the results from our hypothesis-free data-driven approach are surprisingly consistent with the brain-first vs body-first subtype concept^8,9^. The fast-progressing subtype exhibits a higher portion of RBD symptoms, more severe non-motor symptoms, and cognitive impairment at baseline, and a more rapid progression of most symptoms, thereby aligning with the body-first subtype. Contrarily, slow-progressing PwPD exhibit more RBD progression, potentially reflecting the fact that brain-first PwPD would develop RBD after the onset of parkinsonism. Our modeling approach assigned 18% (PPMI) and 27% (ICEBERG, LuxPARK) of PwPD to the fast-progressing subtype. This aligns well with the fact that RBD can be diagnosed via polysomnography in about 25% of PwPD at disease onset, indicating a body-first subtype in these PwPD^30^.

Investigating DaTSCAN results as a direct marker of neurodegeneration in the substantia nigra, we found a similar uptake ratio in both subtypes at baseline. This can be explained by the fact that PD diagnosis depends on the onset of motor symptoms and thus a specific degree of nigrostriatal degeneration. Similar findings have been observed for the brain-first vs body-first concept^8^. DaTSCAN was more asymmetric in slow-progressing PwPD – as proposed for the brain-first subtype where alpha-synuclein spreading starts within one hemisphere^9^. Longitudinally, DaTSCAN asymmetry decreased in the slow-progressing subtype, potentially reflecting the brain-first concept that alpha-synuclein increasingly spreads to the contralateral hemisphere. In contrast, the fast-progressing subtype exhibited a longitudinal increase of DaTSCAN asymmetry which cannot be solely explained by the body-first concept. Although our DaTSCAN findings were mostly consistent with these concepts, a recent study found no differences in gray matter volume loss patterns between PwPD of the body-first and brain-first subtype, indicating the need for further investigations^31^.

Recent studies indicate that PwPD of the body-first subtype exhibits hyposmia more frequently than brain-first PwPD^9,32^. Furthermore, hyposmia is even more common in prodromal body-first PwPD, i.e., in patients with isolated RBD, than in an overall PwPD population which likely consists predominantly of brain-first individuals^33^. For most hyposmic isolated RBD patients, alpha-synuclein was detected in the olfactory mucosa using real-time quaking-induced conversion (RT-QuIC), suggesting alpha-synuclein spreading as the cause of hyposmia in these patients. Our finding of greater olfactory impairment in the fast-progressing subtype is consistent with these previous studies and provides further evidence to this still controversial topic.

The higher portion of gait impairment in fast-progressing PwPD is reflected by differences in a variety of digital gait markers and is consistent with the idea that the brain stem is earlier affected in body-first PwPD^8^, thus confirming our subtype concept by digital biomarkers.

Notably, the brain-first vs body-first concept represents an a priori approach and is still discussed controversially. Other factors, such as demographics, comorbidities, and comedications, have been discussed by others as potential risks or protection for PD diagnosis and/or PD progression^15,16,34^. We investigated several of these factors. In contrast to previous publications, we focused on the association with progression subtypes. We did not observe associations with specific medications or comorbidities. Cerebrospinal fluid markers of Alzheimer’s disease pathology were associated with the fast-progressing subtype, which is consistent with previous reports of faster motor and cognitive worsening in PwPD with additional Alzheimer’s disease pathology^35^. In addition, we observed that higher education was associated with the slow-progressing subtype, a finding similar to the hypothesis of cognitive reserve being a protective factor in Alzheimer’s disease^36^. Interestingly, also greater weight and height were associated with a fast-progressing subtype, possibly because these PwPD may require higher doses of dopaminergic treatment to achieve similar drug concentrations and treatment effects. However, these findings need to be replicated in other cohorts and are difficult to interpret.

PD has traditionally been classified into two subtypes based on the predominant motor phenotype: Postural Instability and Gait Difficulty (PIGD) and Tremor Dominant (TD). The PIGD subtype is associated with faster cognitive decline, more severe non-motor symptoms, and generally faster disease progression, leading to the interpretation that, in contrast, tremor is a predictor for a benign disease course. Multiple studies have observed clinical differences between TD and PIGD subtypes^37^. However, the general idea of this subtype concept has been increasingly challenged over time^37–39^.

One critical observation is that PIGD symptoms typically manifest in the advanced stages of PD, whereas tremor progression is relatively slower, advancing at only half the rate of other motor symptoms^40^. Since PIGD and TD subtypes are typically determined based on the ratio of TD and PIGD symptoms and these symptoms do not progress equally, patients may change subtypes over time. Supporting this, a study revealed that over 50% of patients initially categorized as TD transitioned to the PIGD subtype within eight years, while less than 5% switched in the opposite direction^41^. Thus, it seems likely that the TD and PIGD subtypes represent different stages of the disease rather than distinct disease subtypes.

Our findings align with these critical perspectives. We observed differences in PIGD baseline symptoms between fast- and slow-progressing subtypes, whereas tremor severity at baseline did not show significant differences. This supports the interpretation that tremors should not be considered as a predictor of PD progression.

Our analysis shows significant differences across progression subtypes in features extracted from DaTSCANs and digital gait assessments. This confirms the biological basis of the two progression subtypes and suggests these methods effectively report disease progression. In particular, sensor-based gait assessments are rather inexpensive and could be performed in an at-home setting. Hence, our results contribute to the growing body of literature suggesting the idea to systematically monitoring motor symptoms via such technologies, opening up the possibility for a better-individualized treatment of PD in the future^42^. However, as opposed to most authors we base this conclusion not on a discrimination of PD versus healthy controls, but on a differentiation between PD progression subtypes.

Enriching fast-progressing PwPD in a cohort reduces the variance of progression rates. In addition, neuroprotective effects are potentially higher in fast-progressing PwPD. Both factors enhance the statistical power of clinical trials. Yet, the presumed biological difference between PD subtypes suggests that they may require different treatments. Our simulation of an enrichment trial using PPMI data from a one-year follow-up reduced the required sample size in RCTs by approximately 43%. Depending on the concrete validation scenario and cohort, sample size reductions varied in the range of 0% up to 56% in ICEBERG and LuxPARK. This is in a similar range as demonstrated for Huntington’s disease using a comparable stratification approach^19^. Among the outcomes we investigated, the MDS-UPDRS I-III sum score achieved the highest statistical power compared to other potential primary outcomes, in line with the design of ongoing PD trials^17^. Although our investigated enrichment strategy allows for a significant reduction in sample size, it requires an additional visit before actual study inclusion to achieve optimal results, which may prolong the process of study recruitment. However, the benefit of sample size reduction could compensate for this.

Using LTJMM, we assume an approximately linear progression for the outcomes used in LTJMM. Important to note, no linear progression is assumed across the entire PD course. Rather, we assumed an approximately linear progression only within each cohort, thereby allowing different progression rates in early PD (PPMI, ICEBERG) and advanced PD (LuxPARK). However, other researchers have shown that at least some markers of PD progression demonstrate a non-linear progression profile^43^. Therefore, using an exponential or sigmoid function could potentially be indeed more realistic for some markers, but would at the same time result in a significantly more complex model requiring also more visits per PwPD to accurately estimate model parameters. Despite this simplification, LTJMM has been applied successfully for disease progression modeling in other neurodegenerative disorders^20^.

Another limitation arises from the heterogeneous set of outcomes measured in PPMI, ICEBERG, and LuxPARK. Our choice of outcomes for model training was a trade-off between assessing relevant symptoms and having outcomes measured across all three cohorts. Other outcome choices may be advantageous but would have hampered cross-cohort validation and thus, the question of generalizability.

Although our findings are strengthened by the diversity of our three cohorts, we cannot fully exclude the possibility of bias due to differences between cohorts in disease severity or other cohort-specific characteristics, such as diagnostic criteria, cultural or national differences in healthcare systems, or lifestyle differences. Most of our results were replicated in all three cohorts, minimizing this risk. However, the findings on DaTSCAN, digital gait assessment, levodopa response, survival data and external factors may have limitations in generalizability as they were only based on data available in single cohorts.

Unanswered questions involve how biomarkers like alpha-synuclein pathology assessed by real-time quaking-induced conversion (RT-QuiC) and other digital biomarkers relate to the subtypes. Recently, it has been discussed if different alpha-synuclein strain types may depict the biological basis of the brain-first and body-first subtype^44^. There is also a need to explore how different genetic mutations are related to these subtypes. For example, GBA mutation carriers are suggested to have a shorter PD prodromal phase and present more often RBD, thus relating potentially to the fast-progressing subtype^45–47^. On the other hand, PwPD with LRRK2 G2019S mutation shows less RBD symptoms and hyposmia^45,48^, which may be related to the slow-progressing subtype. Also, future research is needed to assess the association of additional external factors, such as frailty or lifestyle factors, with progression subtypes of PD.

The concept of motor reserve is an emerging topic in PD research. It aims to explain the variation in motor symptoms despite similar dopaminergic deficits^49^. Our subtyping approach is solely based on clinical data and thus ignorant to this concept. Performing a subtyping approach directly on both clinical and neuroimaging data would be interesting for further studies, allowing for subtyping based directly on differences in motor reserve.

Ideally, new clinical trials should assess these biological and digital markers along with a comparable set of clinical markers including the outcomes used in this publication for subtype identification. This will allow researchers to relate the biomarkers to the slow-progressing and fast-progressing subtype, thereby leading to an even more precise description and prediction of PD subtypes.

We provide compelling evidence for the existence of a fast- and slow-progressing subtype in PD as our conclusions are derived from prospective, longitudinal cohorts including more than 1,100 PwPD and were replicated in three distinct PD cohorts. Our findings are in accordance with the body-first vs brain-first and the SOC model, which could potentially provide a biological explanation for the subtypes. Our results enhance the understanding of PD progression heterogeneity and highlight the potential of digital gait assessments to objectively monitor motor symptom progression. Finally, we offer a promising strategy to optimize clinical trial designs or investigate new therapeutic strategies in PD subtypes.

## Methods

### Clinical cohorts

We analyzed PwPD from three cohort studies: (I) de novo PwPD from the Parkinson’s Progression Markers Initiative (PPMI, NCT04477785), (II) early disease stage PwPD from the French ICEBERG cohort study (NCT02305147), and (III) PwPD from all disease stages from the Luxembourg Parkinson’s Study (LuxPARK, NCT05266872)^50^.

### PPMI cohort

We analyzed 409 PwPD from PPMI with clinical visits between 2011 and 2020. All PwPD had a clinical diagnosis of Parkinson’s Disease and a pathological dopamine transporter SPECT (DaTSCAN). We restricted our analysis to untreated de-novo PwPD. Therefore, we included only PwPD with a clinical diagnosis not more than two years before the baseline visit, Hoehn & Yahr stage 0-2, and no dopaminergic treatment at the baseline visit. We further restricted our analysis to PwPD with age > 30 years and at least one additional visit as we require longitudinal information. In addition to clinical scores, DaTSCANs were performed at screening visits and up to three additional visits. Written informed consent to data collection and sharing was obtained from all PwPD by PPMI. Ethical guidelines on human data collection were adhered to. The PPMI project was approved by the Institutional Review Board or Independent Ethics Committee of all participating sites in Europe, including Attikon University Hospital (Greece), Hospital Clinic de Barcelona and Hospital Universitario Donostia (Spain), Innsbruck University (Austria), Paracelsus-Elena-Klinic Kassel/University of Marburg (Germany), Imperial College London (UK), Pitié-Salpêtrière Hospital (France), University of Salerno (Italy), and in the USA, including Emory University, Johns Hopkins University, University of Alabama at Birmingham, PD and Movement Disorders Center of Boca Raton, Boston University, Northwestern University, University of Cincinnati, Cleveland Clinic Foundation, Baylor College of Medicine, Institute for Neurodegenerative Disorders, Columbia University Medical Center, Beth Israel Medical Center, University of Pennsylvania, Oregon Health and Science University, University of Rochester, University of California at San Diego, and University of California, San Francisco.

### ICEBERG cohort

We analyzed 154 PwPD from ICEBERG, an ongoing four-year observational study of PwPD with recent onset of PD conducted at the Paris Brain Institute (Institut du Cerveau-ICM, Pitié-Salpêtrière Hospital, Paris, France). Visits were performed between 2014 and 2022. PD was diagnosed according to UK Parkinson’s Disease Society Brain Bank criteria and PwPD with DaTSCANs showing no dopaminergic deficit were excluded. Inclusion was restricted to disease onset not more than three years before the baseline visit. We further restricted our analysis to PwPD with at least two visits as we require longitudinal information. Written informed consent was obtained and ethical guidelines were adhered to. ICEBERG received approval from the local ethical committee (IRBParis VI, RCB: 2014-A00725-42).

### LuxPARK cohort

We analyzed 561 PwPD from LuxPARK, an ongoing observational study of all disease stages PwPD from Luxembourg and the Greater Region with up to four years follow-up. Visits were performed between 2015 and 2022. PD was diagnosed according to UK Parkinson’s Disease Society Brain Bank criteria. We restricted our analysis to PwPD with at least two visits as we require longitudinal information. In addition to clinical scores, digital gait measurements were performed at one visit for a subset of 177 patients. Written informed consent was obtained and ethical guidelines were adhered to. LuxPARK was approved by the National Ethics Board in Luxembourg (CNER Ref: 201407/13).

### Aligning PwPD trajectories on a common disease timescale

To address temporal heterogeneity between the cohorts, we aligned PwPD on a comparable *common disease timescale* using a latent time joint mixed-effects model (LTJMM)^20^. In brief, LTJMM models a linear progression of multiple clinical outcomes over time and estimates the deviance of an individual PwPD’s progression compared to a “mean PwPD”. Thereby, we estimated how much the timescale of each individual PwPD is shifted from the timescale of the mean PwPD, i.e. where the PwPD is aligned on a common disease timescale using the mean PwPD as reference. For instance, a PwPD with diagnosis in a very early PD stage may exhibit a negative time since diagnosis on the common disease timescale as a mean PwPD won’t be diagnosed at this time. In contrast, PwPD diagnosed at a more advanced stage of PD will present with a higher time since diagnosis at the common disease timescale. To achieve comparability, we used the time since diagnosis as a timescale in all cohorts. The following clinical scores were used as outcomes in LTJMM as they measure a wide variety of motor and non-motor symptoms and were assessed in all three cohorts: Unified Parkinson’s Disease Rating Scale (UPDRS) I-IV, Postural Instability and Gait Dysfunction score (PIGD), Montreal Cognitive Assessment (MoCA) and Scales for Outcomes in Parkinson’s Disease-Autonomic Dysfunction (SCOPA). We accounted for age and sex as covariates.

### Latent time joint mixed-effects model

Disease progression was modeled in each cohort as a linear process using LTJMM:

1 $${y}_{{ijk}}={x}_{i}{\beta }_{k}+{\gamma }_{k}\left({t}_{{ijk}}+{\delta }_{i}\right)+{\alpha }_{0{ik}}+{\alpha }_{1{ik}}{t}_{{ijk}}+{\epsilon }_{{ijk}}$$

Thereby, we denoted *y*_*ijk*_ as outcome *k* observed at measurement *j* for an individual *i*. We accounted for age and sex differences by including age at diagnosis and sex as covariates *x*_*i*_ into the model with *β*_*k*_ as corresponding coefficient shared across all individuals. The coefficient ${\gamma }_{k}$ represented the mean slope of the cohort for each outcome *k* and was thereby shared across all individuals. We used the time since diagnosis as *t*_*ijk*_ and shifted all measurements of an individual by a PwPD specific time shift *δ*_*i*_ shared across all outcomes. Additionally, we included random intercepts *α*_*0ik*_ and random slopes *α*_*1ik*_ for each individual and outcome. As usual, measurement errors *ε*_*ijk*_ and time shifts *δ*_*i*_ were both assumed to be drawn from normal distributions with a mean of zero. Random intercepts and slopes follow a multivariate normal distribution with mean of zero.

Fitting was performed using a Markov chain Monte Carlo (MCMC) algorithm with 4 chains, 25000 iterations, and 12500 warm-up steps. Analyses were performed using the R packages ltjmm^51^ and rstan^52^. All outcomes were min-max-normalized on the theoretical range of the scores. MoCA scores were inverted to ensure positive slopes for all outcomes. Convergence of MCMCs and normal distribution of parameter estimates were inspected manually. In addition, $\hat{R}$ statistics were calculated and ensured to be below 1.05.

To visualize and validate the effect of aligning PwPD on a common timescale, we inspected the distributions of Hoehn & Yahr (H&Y) stages which were not used for fitting the LTJMM model. Thereby, we observed a clearer separation of H&Y stages after applying LTJMM to the data (Supplementary Fig. 1).

Further, we inspected the accuracy of our LTJMM approach in predicting outcomes at the next visit. Therefore, we re-trained LTJMM, but excluded the last measurement of all outcomes. Using this LTJMM model, we predicted these last measurements of all outcomes and calculated the coefficient of determination (R^2^) for these predictions. Thereby, we obtained reasonable R^2^ values: 55% (PPMI), 53% (ICEBERG), 61% (LuxPARK).

### Subtype identification using VaDER

Parkinson’s disease (PD) progression subtypes were identified using variational deep embedding with recurrence (VaDER). Briefly, VaDER implements a recurrent variational autoencoder, in which each data point in the latent space (representing a multivariate patient trajectory) is mapped to a mixture of Gaussians rather than to a single Gaussian. Using these techniques, VaDER identifies subtypes in multivariate longitudinal data for short time series. A further distinction point of VaDER is that the input data is passed through an imputation layer. That means that VaDER can directly deal with longitudinal data containing missing values (including those that may occur not at random) and does not require any error-prone pre-imputation. A detailed technical description of the VaDER algorithm can be obtained from the original publication^29^.

We used the predicted LTJMM outcomes $\hat{{y}_{{ijk}}}$ of UPDRS I-IV, PIGD, MoCA and SCOPA on the common timescale to calculate *outcome progression scores*. The outcomes used are slightly different from our recent work to allow comparability between the different cohorts^11^. Outcome progression scores were calculated for each PwPD by subtracting the outcome at *t* = 0 from all outcomes and dividing it by the standard deviation of the outcome at *t* = 0. Outcome progression scores were used as input for VaDER.

Hyperparameter optimization was performed using a random search through the following grid: learning rate = {0.0001, 0.001, 0.01, 0.1}, batch size = {16, 32, 64} number of nodes in the hidden layers = {1, 2, 4, 8, 16, 32, 64}. Overall, 360 hyperparameter sets were sampled from the grid and evaluated for k = {2, 3, 4, 5} number of clusters with 50 epochs used for VaDER training.

We have chosen the number of subtypes based on the prediction strength method described in the original VaDER publication^29^, which is itself an adoption of Tibshirani & Walther^53^. Different numbers of subtypes were considered in VaDER, ranging from two to five. VaDER was trained for each number of subtypes, and the model’s performance was evaluated by comparing its prediction strength against a random subtyping of the same data. We chose the model with the smallest number of subtypes, which demonstrated a statistically significant difference to a random clustering in terms of achieved prediction strength. The increase in prediction strength compared to the null model was already significant for *k* = 2 in all cohorts, thus we considered two clusters as appropriate. The final models were trained 20 times and consensus clustering was used to finally assign PwPD to the clusters. The hyperparameters obtained from this approach are presented in Supplementary Table 9 and were used for all following calculations.

### Cross-cohort validation

To achieve optimal representation in each cohort, LTJMM and VaDER were applied separately to each cohort. The generalizability of our findings was evaluated by a cross-cohort validation using PPMI for training and validating the VaDER and predictive models on ICEBERG and LuxPARK. PPMI was chosen as a training model as it is publicly available.

### Symptom domain comparisons

To allow a more comprehensive comparison and validation of the variety of outcomes captured across the three cohorts, we grouped 114 outcomes (including single questions, scores and sub-scores from questionnaires and clinical assessments) into 22 symptom domains (Supplementary Table 2). The choice of the 22 symptom domains represents a trade-off between capturing most clinically relevant motor and non-motor symptoms and which outcomes had been assessed in the three cohorts. For each cohort, we included only outcomes where at least 30 PwPD in total and 5 PwPD per subtype were assessed. In addition, at least two measurements per PwPD were required for the progression analysis. For the baseline characteristics analysis, we only evaluated values at baseline and screening visits.

To assess the progression characteristics of both subtypes regarding the defined symptom domains, we applied the following steps: Outcomes were normalized using min-max-normalization. Scales where high values report a low symptom severity were inverted, thus we ensured that high outcome scores always correspond to a high symptom severity. Depending on the scale of each outcome, we modeled the outcome progression using a linear mixed-effects model, a binary mixed-effects model, or an ordinal mixed-effects model on the common disease timescale. We used one model per subtype. For each outcome, coefficients depicting outcome progression were extracted for each PwPD and standardized mean differences (SMDs) between subtypes were calculated. Next, we conducted a three-level meta-analysis with random effects for each symptom domain. Thereby, we first calculated for each cohort an overall SMD estimate across all outcomes of one symptom domain. Subsequently, we calculated an overall SMD estimate of the symptom domain across the three cohorts (see forest plots at the end of the supplement). P-values and 95% confidence intervals (CI) were corrected for multiple testing across the 22 symptom domains using the Benjamini-Hochberg procedure^54^.

To assess the association of baseline characteristics with the subtypes regarding the defined symptom domains, we applied the following steps: Outcomes were normalized using min-max-normalization. Scales where high values report a low symptom severity were inverted, thus we ensured that high outcome scores always correspond to a high symptom severity. For each outcome, we trained a logistic regression model to predict the subtype based on the baseline outcome value while using disease duration on the common disease timescale as covariate. For each outcome, the logistic regression coefficient was extracted. Next, we conducted a three-level meta-analysis with random effects for each symptom domain. Thereby, we first calculated an overall regression coefficient estimate across all outcomes of one symptom domain per cohort. Subsequently, we calculated an overall regression coefficient estimate of the symptom domain across the three cohorts (see forest plots at the end of the supplement). P-values and 95% confidence intervals (CI) were corrected for multiple testing across the 22 symptom domains using the Benjamini-Hochberg procedure^54^.

In addition, we compared baseline outcomes directly, i.e., without a correction for differences in disease duration (Supplementary Fig. 5).

Both analyses were repeated as a cross-cohort validation (see forest plots at the end of the supplement).

Analyses were performed using the R-packages lme4^55^, ordinal^56^ and meta^57^.

### Survival analysis

To compare mortality between subtypes, we performed a survival analysis for LuxPARK using a Cox proportional hazards model with subtype, age, and sex as covariates and the common disease timescale as time variable. The analysis was implemented using the Python lifelines package^58^.

### Treatment response analysis

UPDRS III in PPMI is reported at annual visits in the OFF state defined by the last medication intake at least 6 h ago and after medication intake in the ON state. We calculated the treatment response as the relative improvement in UPDRS III after medication intake and averaged responses to overall clinical visits at which UPDRS III was performed in ON and OFF states.

### DaTSCAN analysis

In PPMI, DaTSCAN analysis was performed at screening visits and up to three additional visits. We compared the signal binding ratio (SBR) and asymmetry index between subtypes obtained at screening visits using a t-test. For PwPD with longitudinal DaTSCAN measurements available, we modeled SBR and asymmetry index changes over time using a linear mixed-effects model. Subsequently, we compared the obtained progression rates of SBR and asymmetry index between subtypes using a *t*-test.

### Gait analysis

In LuxPARK, PwPD completed a standardized gait assessment at one visit using the automated gait assessment system eGaIT^59^. PwPD underwent a timed up-and-go (TUG) task using accelerometer and gyroscope sensors attached to their shoes. 15 digital gait parameters were calculated based on straight steps from the TUG task. Gait differences between subtypes were analyzed by conducting an ANCOVA and controlling for disease duration on the common disease timescale. P-values and CI were corrected for multiple testing using the Benjamini-Hochberg procedure. The digital gait parameters are described in Supplementary Table 3. The correlation structure of the gait parameters was assessed by an exploratory factor analysis using the Python package factor-analyzer^60^. Cronbach’s alpha was calculated using the Python package pingouin^61^. The number of three factors was determined visually from the scree plot (Supplementary Fig. 9).

### Assessment of external factors

External factors were analyzed at baseline in PPMI. If parameters were not measured at baseline, the screening visit was used for analysis instead. The relationship between the external factor and the progression subtype was analyzed using the following logistic regression formula with the Python package statsmodel^62^, including the covariates age and sex: *progression_subtype ~ external factor + age + sex*. Correction for multiple testing was performed using the Benjamini-Hochberg procedure within each group of analyses (i.e., education, body measures, specific comorbidities, comorbidity groups, comedications, Alzheimer’s disease pathology).

Comorbidities were analyzed at the group level and at the individual disease level. Group-level analyses were performed for the 15 categories defined by PPMI: dermatological, ophthalmological, ENT, pulmonary, cardiovascular, gastrointestinal, hepatobiliary, renal, gynecological/urologic, musculoskeletal, metabolic/endocrine, hemato/lymphatic, psychiatric, allergy/immunologic, other.

The analysis of individual diseases was performed for the following diseases: hypertension, hypercholesterolemia, diabetes, gout, cancer, head injury, appendectomy, oophorectomy. Therefore, we searched for disease terms that contained related strings (hypertension; hypercholesterolemia, cholesterol; diabetes, diabetic; gout, hyperuricemia; cancer, neoplasm, tumor, carcinoma, malignancy, sarcoma, lymphoma, melanoma, leukemia; head injury; appendectomy, appendix, appendicectomy; oophorectomy, ovary removal, ovarian surgery, salpingectomy) and manually checked the search results. We searched for entries for hypertension, diabetes, hypercholesterolemia, and gout in the current medical conditions log. Appendectomy, oophorectomy, and head injury entries were searched in the medical history log. Cancer records were searched in both logs. Head injury and oophorectomy were only documented in one PwPD and were not analyzed further.

Comedications were analyzed at the following group levels by including the most common medications from each group. We evaluated groups by searching the following strings in the medication log: NSAR (ASS, Aspirin, Ibuprofen, Naproxen, Diclofenac, Celecoxib, Indomethacin), calcium channel antagonists (Amlodipine, Diltiazem, Nifedipine, Verapamil, Felodipine, Nicardipine, Isradipine), beta agonists (Salbutamol, Terbutaline, Formoterol, Salmeterol), beta antagonists (Atenolol, Metoprolol, Bisoprolol, Carvedilol, Nebivolol, Propranolol, Sotalol), contraceptives (Estradiol, Levonorgestrel, Norethindrone, Desogestrel, Drospirenone, Medroxyprogesterone) and statins (Atorvastatin, Simvastatin, Rosuvastatin, Pravastatin, Lovastatin, Fluvastatin, Pitavastatin). In addition, we analyzed separately NSAR without ASS, ASS, and Ibuprofen. Past and current comedication were taken into account.

### Subtype prediction models

We developed several models to predict PwPD subtypes from (I) baseline and (II) baseline and one additional visit. Therefore, we used penalized Logistic Regression with L2 regularization, Random Forest^63^, and eXtreme Gradient Boosting (XGBoost)^64^. These predictive models were implemented using the python packages scikit-learn^65^ and xgboost^64^. We used UPDRS I-III, PIGD, MoCA, and SCOPA as baseline predictors as they capture a variety of motor and non-motor symptoms and were measured across the three cohorts. UPDRS IV was not included as it was mostly not assessed at baseline. Hyperparameter optimization was performed using grid search (Logistic Regression) or randomized search (Random Forest, XGBoost) with 50 iterations in an inner repeated cross-fold validation using 5 folds with 20 repeats (Supplementary Table 10). Class weights were used to accommodate unbalanced classes. Estimates for receiver operating characteristics-area under the curve (ROC-AUC) were obtained from an outer repeated cross-fold validation using 5 folds with 20 repeats. Cross-cohort validation was performed by training the predictive model on the complete PPMI dataset using the best parameters from the hyperparameter optimization and predicting the subtype assignments of ICEBERG/LuxPARK. Furthermore, we assessed how much these predictions can be improved if short follow-up data is included in the models. Therefore, we repeated the steps above using outcomes at baseline and at one-year follow-up.

### Sample size estimation using subtype predictions

To assess the effect of enriching fast-progressing PwPD in clinical trials on required sample sizes and power, we simulated a randomized controlled trial (RCT) for a potential disease-modifying drug based on considerations of an ongoing clinical trial^17^. We assumed measurements of UPDRS I-III sum score every 60 days for a total of one year of observation time. A power of 80% and a significance level of 0.1 was chosen. We assumed a treatment effect of a 30% reduction in disease progression speed^17^. For simplification, we used equally sized control and treatment groups without different treatment dosage arms. Power and sample size were calculated based on PPMI data using a linear mixed-effects model, thereby assuming a linear UPDRS I-III increase over time. Calculations were based on a method from Edland et al.^66^ and implemented using the R package longpower^67^. Sample size and power calculations were performed for different percentages of fast-progressing PwPD. Enrichment of fast-progressing PwPD was simulated using predictions of the PPMI logistic regression models as this model outperformed Random Forest and XGBoost in predicting PwPD subtypes.

### Statistical analysis

For comparison of cohort characteristics, the following tests were applied: Sex was compared using Fisher’s exact test, Hoehn & Yahr using the Kolmogorov-Smirnov test, and all other characteristics were compared using the Mann-Whitney U test. P-values were adjusted for multiple tests using the Benjamini-Hochberg procedure.

All statistical tests were conducted as two-tailed tests with a significance level of 0.05.

### Reporting summary

Further information on research design is available in the Nature Research Reporting Summary linked to this article.

### Supplementary information

Supplementary Material

Reporting summary

## Data Availability

As this study is a retrospective analysis, the availability of the clinical data depends on the individual study groups (PPMI: www.ppmi-info.org, ICEBERG: marie.vidailhet@psl.aphp.fr, LuxPARK: rejko.krueger@uni.lu).

## Code availability

The underlying code used for training LTJMM, VaDER, and all relevant statistical analyses for this study will be published on GitHub upon acceptance of the paper under the CC BY-NC-ND 4.0 license and can be accessed via https://github.com/SCAI-BIO/PD-progression-types.

## References

[1] Feigin VL (2017). Global, regional, and national burden of neurological disorders during 1990–2015: a systematic analysis for the Global Burden of Disease Study 2015. Lancet Neurol..

[2] Kieburtz K (2015). Effect of Creatine monohydrate on clinical progression in patients with Parkinson Disease: A randomized clinical trial. JAMA.

[3] Lang AE (2022). Trial of Cinpanemab in early Parkinson’s disease. N. Engl. J. Med.

[4] Pagano G (2022). Trial of Prasinezumab in early-stage Parkinson’s disease. N. Engl. J. Med.

[5] The Parkinson Study Group SURE-PD3 Investigators et al. (2021). Effect of urate-elevating Inosine on early Parkinson disease progression: The SURE-PD3 randomized clinical trial. JAMA.

[6] Greenland JC, Williams-Gray CH, Barker RA (2019). The clinical heterogeneity of Parkinson’s disease and its therapeutic implications. Eur. J. Neurosci..

[7] Berg D (2021). Prodromal Parkinson disease subtypes — key to understanding heterogeneity. Nat. Rev. Neurol..

[8] Horsager J (2020). Brain-first versus body-first Parkinson’s disease: a multimodal imaging case-control study. Brain.

[9] Borghammer P (2021). The α-Synuclein Origin and Connectome Model (SOC Model) of Parkinson’s Disease: Explaining motor asymmetry, non-motor phenotypes, and cognitive decline. JPD.

[10] Gerraty RT (2023). Machine learning within the Parkinson’s progression markers initiative: Review of the current state of affairs. Front. Aging Neurosci..

[11] Birkenbihl C (2023). Artificial intelligence-based clustering and characterization of Parkinson’s disease trajectories. Sci. Rep..

[12] Zhang X (2019). Data-driven subtyping of Parkinson’s disease using longitudinal clinical records: a cohort study. Sci. Rep..

[13] Fereshtehnejad S-M (2015). New clinical subtypes of Parkinson disease and their longitudinal progression: a prospective cohort comparison with other phenotypes. JAMA Neurol..

[14] Dadu A (2022). Identification and prediction of Parkinson’s disease subtypes and progression using machine learning in two cohorts. npj Parkinsons Dis..

[15] Belvisi D (2020). Modifiable risk and protective factors in disease development, progression and clinical subtypes of Parkinson’s disease: What do prospective studies suggest?. Neurobiol. Dis..

[16] Mollenhauer B (2019). Baseline predictors for progression 4 years after Parkinson’s disease diagnosis in the De Novo Parkinson Cohort (DeNoPa). Mov. Disord..

[17] UCB Biopharma SRL. *A Double-Blind, Placebo-Controlled, Randomized, 18-Month Phase 2a Study to Evaluate the Efficacy, Safety, Tolerability, and Pharmacokinetics of Oral UCB0599 in Study Participants With Early Parkinson’s Disease*. https://clinicaltrials.gov/study/NCT04658186 (2023).

[18] Braak H (2003). Staging of brain pathology related to sporadic Parkinson’s disease. Neurobiol. Aging.

[19] Koval I (2022). Forecasting individual progression trajectories in Huntington disease enables more powered clinical trials. Sci. Rep..

[20] Li D, Iddi S, Thompson WK, Donohue MC, Alzheimer’s Disease Neuroimaging Initiative (2019). Bayesian latent time joint mixed effect models for multicohort longitudinal data. Stat. Methods Med. Res..

[21] Post B, Speelman JD, Haan RJ, on behalf of the CARPA-Study Group (2008). Clinical heterogeneity in newly diagnosed Parkinson’s disease. J. Neurol..

[22] Belvisi D (2021). The pathophysiological correlates of Parkinson’s disease clinical subtypes. Mov. Disord..

[23] Vivacqua G (2023). Salivary α‐Synuclein RT‐QuIC Correlates with Disease Severity in de novo Parkinson’s Disease. Mov. Disord..

[24] Zhou C (2023). Two distinct trajectories of clinical and neurodegeneration events in Parkinson’s disease. npj Parkinsons Dis..

[25] Erro R (2016). Clinical clusters and dopaminergic dysfunction in de-novo Parkinson disease. Parkinsonism Relat. Disord..

[26] Fereshtehnejad S-M, Zeighami Y, Dagher A, Postuma RB (2017). Clinical criteria for subtyping Parkinson’s disease: biomarkers and longitudinal progression. Brain.

[27] Belvisi, D. et al. Risk factors of Parkinson disease: Simultaneous assessment, interactions, and etiologic subtypes. *Neurology* **95**, (2020). 10.1212/WNL.0000000000010813 PMC7682833 32943485

[28] Emon MA (2020). Clustering of Alzheimer’s and Parkinson’s disease based on genetic burden of shared molecular mechanisms. Sci. Rep..

[29] de Jong J (2019). Deep learning for clustering of multivariate clinical patient trajectories with missing values. GigaScience.

[30] Mollenhauer B (2013). Nonmotor and diagnostic findings in subjects with de novo Parkinson disease of the DeNoPa cohort. Neurology.

[31] Banwinkler M, Dzialas V, The Parkinson’s Progression Markers Initiative, Hoenig MC, Van Eimeren T (2022). Gray matter volume loss in proposed brain‐first and body‐first Parkinson’s disease subtypes. Mov. Disord..

[32] Kim J (2017). Normal ‘heart’ in Parkinson’s disease: is this a distinct clinical phenotype?. Eur. J. Neurol..

[33] Stefani A (2021). Alpha-synuclein seeds in olfactory mucosa of patients with isolated REM sleep behaviour disorder. Brain.

[34] Belvisi D (2022). The role of frailty in Parkinson’s disease: a cross-sectional study. J. Neurol..

[35] Hall S (2015). CSF biomarkers and clinical progression of Parkinson disease. Neurology.

[36] Stern Y (2012). Cognitive reserve in ageing and Alzheimer’s disease. Lancet Neurol..

[37] Fereshtehnejad S-M, Postuma RB (2017). Subtypes of Parkinson’s disease: what do they tell us about disease progression?. Curr. Neurol. Neurosci. Rep..

[38] Kotagal V (2016). Is PIGD a legitimate motor subtype in Parkinson disease?. Ann. Clin. Transl. Neurol..

[39] Nutt JG (2016). Motor subtype in Parkinson’s disease: Different disorders or different stages of disease?: Motor Subtypes of PD. Mov. Disord..

[40] Vu TC, Nutt JG, Holford NHG (2012). Progression of motor and nonmotor features of Parkinson’s disease and their response to treatment. Brit J. Clin. Pharma.

[41] Alves G, Larsen JP, Emre M, Wentzel-Larsen T, Aarsland D (2006). Changes in motor subtype and risk for incident dementia in Parkinson’s disease. Mov. Disord..

[42] Fröhlich H (2022). Leveraging the potential of digital technology for better individualized treatment of Parkinson’s disease. Front. Neurol..

[43] Kuramoto L (2013). The nature of progression in Parkinson’s Disease: An application of non-linear, multivariate, longitudinal random effects modelling. PLoS ONE.

[44] Just MK (2022). Alpha-Synuclein strain variability in body-first and brain-first Synucleinopathies. Front. Aging Neurosci..

[45] Huang J, Cheng Y, Li C, Shang H (2022). Genetic heterogeneity on sleep disorders in Parkinson’s disease: a systematic review and meta-analysis. Transl. Neurodegener..

[46] Krohn L (2020). *GBA* variants in REM sleep behavior disorder: A multicenter study. Neurology.

[47] Zimmermann M (2019). Patient’s perception: shorter and more severe prodromal phase in GBA ‐associated PD. Eur. J. Neurol..

[48] Gaig C (2014). Nonmotor symptoms in LRRK2 G2019S associated Parkinson’s disease. PLoS ONE.

[49] Hoenig MC, Dzialas V, Drzezga A, Van Eimeren T (2023). The concept of motor reserve in Parkinson’s disease: new wine in old bottles?. Mov. Disord..

[50] Hipp G (2018). The Luxembourg Parkinson’s Study: A comprehensive approach for stratification and early diagnosis. Front Aging Neurosci..

[51] Donohue, M. mdonohue / ltjmm — Bitbucket. https://bitbucket.org/mdonohue/ltjmm/src/master/ (2017).

[52] Carpenter, B. et al. *Stan*: A Probabilistic Programming Language. *J. Stat. Soft*. **76**, (2017). 10.18637/jss.v076.i01 PMC9788645 36568334

[53] Tibshirani R, Walther G (2005). Cluster validation by prediction strength. J. Comput. Graph. Stat..

[54] Benjamini Y, Hochberg Y (1995). Controlling the false discovery rate: a practical and powerful approach to multiple testing. J. R. Stat. Soc.: Ser. B (Methodol.).

[55] Bates, D., Mächler, M., Bolker, B. & Walker, S. Fitting Linear Mixed-Effects Models Using lme4. *J. Stat. Soft*. **67**, (2015).

[56] Christensen, R. H. B. ordinal—Regression Models for Ordinal Data. (2022).

[57] Balduzzi S, Rücker G, Schwarzer G (2019). How to perform a meta-analysis with R: a practical tutorial. Evid. Based Ment. Health.

[58] Davidson-Pilon C (2019). Lifelines: survival analysis in Python. JOSS.

[59] Klucken J (2013). Unbiased and mobile gait analysis detects motor impairment in Parkinson’s disease. PLoS ONE.

[60] Biggs, J. factor-analyzer. A Factor Analysis tool written in Python.

[61] Vallat R (2018). Pingouin: statistics in Python. JOSS.

[62] Seabold, S. & Perktold, J. statsmodels: Econometric and statistical modeling with python. *in 9th Python in Science Conference*, (2010).

[63] Breiman L (2001). Random forests. Mach. Learn..

[64] Chen, T. & Guestrin, C. XGBoost: A Scalable Tree Boosting System. in *Proceedings of the 22nd ACM SIGKDD International Conference on Knowledge Discovery and Data Mining* 785–794 (ACM, San Francisco California USA, 2016). 10.1145/2939672.2939785.

[65] Pedregosa F (2011). Scikit-learn: Machine learning in Python. J. Mach. Learn. Res..

[66] Ard MC, Edland SD (2011). Power calculations for clinical trials in Alzheimer’s disease. JAD.

[67] Iddi S, Donohue MC (2022). Power and sample size for longitudinal models in R - The longpower package and shiny app. R. J..

