# Supplementary material for: Progression subtypes in Parkinson’s disease identified by a data-driven multi cohort analysis
Source: NPJ Parkinsons Dis. 2024 May 2;10:95. doi: 10.1038/s41531-024-00712-3 (PMC11066039; doi:10.1038/s41531-024-00712-3)
Supplement: Supplementary file 1 — Supplementary Material [file 41531_2024_712_MOESM1_ESM.pdf]

Supplementary Materials

Progression subtypes in Parkinson’s disease identified by a data driven multi cohort analysis

Tom Hähnel[1,2*], Tamara Raschka[1,3] Stefano Sapienza[4,5], Jochen Klucken[4,5,6], Enrico Glaab[4], Jean-Christophe Corvol[7], Björn H. Falkenburger[2,8], Holger Fröhlich[1,3]

1. Department of Bioinformatics, Fraunhofer Institute for Algorithms and Scientific Computing (SCAI), Sankt Augustin, Germany
2. Department of Neurology, Medical Faculty and University Hospital Carl Gustav Carus, TUD Dresden University of Technology, Dresden, Germany
3. Bonn-Aachen International Center for IT, University of Bonn, Bonn, Germany
4. Biomedical Data Science, Luxembourg Centre for Systems Biomedicine (LCSB), University of Luxembourg, Esch-sur-Alzette, Luxembourg
5. Luxembourg Institute of Health (LIH), Strassen, Luxembourg
6. Centre Hospitalier de Luxembourg (CHL), Luxembourg
7. Sorbonne Université, Paris Brain Institute – ICM, Inserm, CNRS, Assistance Publique Hôpitaux de Paris, Pitié-Salpêtrière Hospital, Department of Neurology, Paris, France
8. German Center for Neurodegenerative Diseases (DZNE), Tatzberg 41, 01307 Dresden, Germany

Corresponding author:
Dr. med. Tom Hähnel
Department of Bioinformatics
Fraunhofer Institute for Algorithms and Scientific Computing (SCAI)
Schloss Birlinghoven 1
53757 Sankt Augustin, Germany
Phone: +49 (0)351 458 11880
Email: tom.haehnel@scai-extern.fraunhofer.de

Running title: Parkinson's Disease Progression Subtypes

Progression subtypes in Parkinson's disease identified by a data driven multi cohort analysis

Table of Contents

Supplementary Figures and Tables....3
Forest plots for symptom domain progression (in cohort)....31
Forest plots for symptom domain progression (cross-cohort validation)....32
Forest plots for symptom domain baseline associations (in cohort)....33
Forest plots for symptom domain baseline associations (cross-cohort validation)....34
ICEBERG study group....35
NCER-PD/LuxPARK consortium....36

Supplementary Figures and Tables

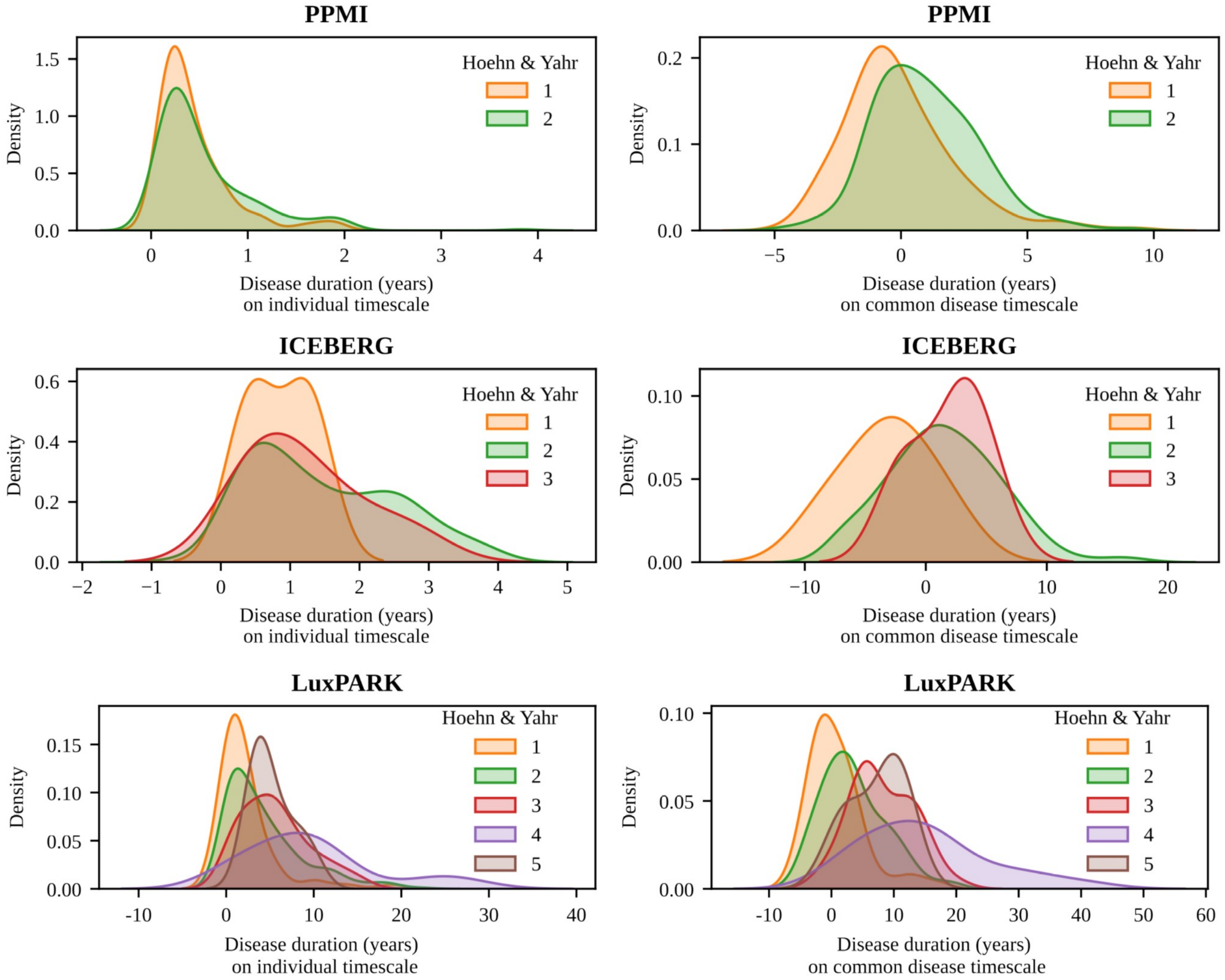

Supplementary Figure 1: Effect of time-aligning PwPD on distributions of H&Y stages

The H&Y distributions from PPMI, ICEBERG and LuxPARK at baseline are depicted. On the left side, H&Y stages are plotted against the original time scale. On the right side, H&Y stages are plotted against the common timescale calculated from the LTJMM.

Abbreviations: H&Y: Hoehn&Yahr, LTJMM: latent time joint mixed-effects model

Study and parameter	Fast-progressing subtype	Slow-progressing subtype	p-value
Age (years)			
PPMI	67.6	62.0	*<0.0001*
ICEBERG	64.0	63.7	0.86
LuxPARK	70.1	66.8	*<0.0001*
Age at onset (years)			
PPMI	67.1	61.4	*<0.0001*
ICEBERG	62.5	61.4	0.86
LuxPARK	67.0	60.5	*<0.0001*
Disease duration (years)			
PPMI	0.3	0.3	0.86
ICEBERG	1.3	1.2	0.86
LuxPARK	2.8	3.0	0.44
Sex (% male)			
PPMI	73.0 %	65.4 %	0.38
ICEBERG	71.4 %	58.9 %	0.38
LuxPARK	73.2 %	65.4 %	0.21

Supplementary Table 1: Demographic differences between progression subtypes

PwPD baseline characteristics for fast-progressing and slow-progressing subtypes for the three cohorts PPMI, ICEBERG and LuxPARK. For sex, percentage of male PwPD is shown. For other characteristics, median values are reported. Corresponding p-values were corrected for multiple testing using Benjamini-Hochberg procedure.

**Symptom domain**	**Outcome**	**Definition/calculation of the outcome**
Anxiety	NMSQ Anxiety	NMSQ item 17
	HADS anxiety	HADS anxiety sub-score
	STA	STA sum score
	PDQ39 Anxiety	PDQ39 item 21
	UPDRS I Anxiety	UPDRS I item 4
Apathy	DAS	DAS sum score
	SAS	SAS sum score
	UPDRS I Apathy	UPDRS I item 5
Autonomic symptoms	NMSQ Autonomic	NMSQ sum of items 4, 5, 6, 7, 8, 9, 19, 20, 28
	SCOPA-AUT	SCOPA sum score
	UPDRS I Autonomic	UPDRS I sum of items 10, 11, 12
Attention	NMSQ Attention	NMSQ item 15
	Letter Number Sequencing	Letter Number Sequencing score
	Digit Span	Digit Span Score: sum of forward scores and backward scores
	MATTIS Attention	MATTIS attention sub-score
	MOCA Attention	MoCA sum of: digits, letters, subtraction points
	MMSE Attention	MMSE attention sub-score
	PDQ39 Attention	PDQ39 item 31
	SIQCDE Attention	Short IQCODE score item 11
Conceptualization	MATTIS Conceptualization	MATTIS conceptualization sub-score
	MOCA Abstraction	MoCA abstraction sub-score
	FAB Conceptualization	FAB item 1
Language	Boston Naming Test	Boston Naming Test sum score
	MOCA Language + Naming	MoCA sum of items: naming, repeat and verbal fluency task
	VFT phonematic F	phonematic VFT F total word count
	VFT phonematic S	phonematic VFT S total word count
	VFT semantic animal	semantic VFT animal total word count
	VFT semantic sum	semantic VFT total word count (sum of tasks colors, fruits, towns, animals)
	VFT semantic supermarket	semantic VFT supermarket total word count
	MMSE Language	MMSE language sub-score
	FAB VFT	FAB lexical fluency item
Memory	NMSQ Memory	NMSQ item 12
	CERAD Words DR	CERAD word count immediate recall
	CERAD Words IR	CERAD word count delayed recall
	CERAD Words Recognition	CERAD recognition (number of correct, Yes + No)
	Hopkins Verbal Learning Test DR	Hopkins Verbal Learning Test delayed recall
	Hopkins Verbal Learning Test IR	Hopkins Verbal Learning Test immediate recall
	MOCA Orientation + Memory	MoCA sum of: memory (uncued only), orientation
	MATTIS Memory	MATTIS memory sub-score
	MMSE Memory	MMSE sum of: orientation (location + time), words memorization
	SIQCDE Memory	Short IQCODE score sum of items 1, 2, 3, 4, 5, 6, 7
	PDQ39 Memory	PDQ39 item 32
Overall Cognition	MATTIS	MATTIS sum score
	MMSE	MMSE sum score
	MoCA	MoCA sum score
	SIQCDE	Short IQCODE score sum score
	FAB	FAB sum score

Symptom domain	Outcome	Definition/calculation of the outcome
	PDQ39 Cognition	PDQ39 sum of items 31, 32
	UPDRS I Cognition	UPDRS I item 1
	NMSQ Cognition	NMSQ sum of items 12, 15
Visu-executive function	MMSE Construction	MMSE item 30
	Judgment Line Orientation	Judgment of Line Orientation sum score
	Symbol Digit Modalities	Symbol Digital Modalities sum score
	Stroop Errors	Stroop test number of errors
	Stroop Time	Stroop test required time
	Trailmaking A	Trail Making Test A time
	Trailmaking B	Trail Making Test B time
	MATTIS Initiation + Construction	MATTIS sum of: sub-score initiation, sub-score construction
	FAB 3-6	FAB sum of items 3, 4, 5, 6
	MOCA Visuspatial/Executive	MoCA visuospatial/executive sub-score
Depression	BDI	BDI sum score
	GDS	GDS sum score
	HADS depression	HADS depression sub-score
	PDQ39 Depression	PDQ39 sum of items 17, 18, 19, 20, 22
	NMSQ Depression	NMSQ sum of items 13, 16
	UPDRS I Depression	UPDRS 1 item 3
Fatigue	UPDRS I Fatigue	UPDRS 1 item 13
Hallucinations	NMSQ Hallucination	NMSQ sum of items 14, 30
	UPDRS I Hallucinations	UPDRS 1 item 2
Impulsivity	QUIP	QUIP sum score
	QUIP-RS	QUIPRS sum score
Motor symptoms (overall)	PDQ39 ADL	PDQ39 ADL sub-score
	Pegboard	PEGBoard sum of: average of left hand, right hand and both hands
	UPDRS II	UPDRS II sum score
	UPDRS III off	UPDRS III sum score (OFF only)
	UPDRS III on	UPDRS III sum score (ON only)
	UPDRS IV	UPDRS IV sum score
Non motor symptoms (overall)	NMSQ	NMSQ sum score
	UPDRS I	UPDRS I sum score
Overall disease severity	UPDRS I-III on	UPDRS I, II, III sum (ON only)
	UPDRS I-III off	UPDRS I, II, III sum (OFF only)
	FAQ	FAQ sum score
	PDQ39	PDQ39 sum score
	SEADL	SEADL score
	H&Y	Hoehn & Yahr
	CGIS	CGI-S score
Pain	NMSQ Pain	NMSQ item 10
	PDQ39 Pain	PDQ39 sum of items 37, 38
	UPDRS I Pain	UPDRS 1 item 9
Axial & PIGD symptoms	UPDRS III axial off	UPDRS III axial score (OFF only)
	UPDRS III axial on	UPDRS III axial score (ON only)
	FOGAC	FOGAC sum score
	FOGQ	FOGQ sum score

Symptom domain	Outcome	Definition/calculation of the outcome
	GABS Examination	GABS sum of items 8-24
	GABS Questionnaire	GABS sum of items 1-7
	NFOGQ	NFOGQ sum score
	PDQ39 Mobility	PDQ39 mobility sub-score
	PIGD off	PIGD score (OFF only)
	PIGD on	PIGD score (ON only)
	TUG	Timed Up and Go time
Sleep (general)	ESS	ESS sum score
	PDSS	PDSS sum score
	UPDRS I Sleep	UPDRS I sum of items 7, 8
	NMSQ Sleep	NMSQ sub of items 22, 23
RBD Sleep	RBD-HK	RBD-HK sum score
	RBD-SQ	RBD-SQ sum score
	NMSQ RBD	NMSQ sum of items 24, 25
Smell	NMSQ Smell	NMSQ item 2
	Sniffin Test	Sniffin Test score
	UPSIT	UPSIT sum score
Tremor	TD off	TD score (OFF only)
	TD on	TD score (ON only)

Supplementary Table 2: Construction of symptom domains

Abbreviations: BDI: Beck Depression Inventory, CERAD: Consortium to Establish a Registry for Alzheimer's Disease, CGIS: Clinical Global Impression-Severity, DAS: Dimensional Apathy Scale, ESS: Epworth Sleepiness Scale, FAB: Frontal Assessment Battery, FAQ: Functional Activities Questionnaire, FOGAC: Freezing of Gait AC, FOGQ: Freezing of Gait Questionnaire, GABS: Clinical Gait and Balance Scale, GDS: Geriatric Depression Scale, H&Y: Hoehn & Yahr scale, HADS: Hospital Anxiety and Depression Scale, MATTIS: Mattis Dementia Rating Scale, MMSE: Mini Mental Status Examination, MOCA: Montreal Cognitive Assessment, NFOGQ: New Freezing of Gait Questionnaire, NMSQ: Non-Motor Symptoms Questionnaire, PDQ39: Parkinson's Disease Questionnaire-39, PDSS: Parkinson's Disease Sleep Scale, PIGD: Postural Instability and Gait Disorder score, QUIP: Questionnaire for Impulsive-Compulsive Disorders, QUIP-RS: QUIP-Rating Scale, RBD-HK: REM Sleep Behavior Disorder Questionnaire-Hong Kong, RBD-SQ: REM Sleep Behavior Disorder Screening Questionnaire, SAS: Starkstein Apathy Scale, SCOPA-AUT: Scales for Outcomes in Parkinson’s Disease-Autonomic Dysfunction, SEADL: Schwab and England Activities of Daily Living Scale, SIQCDE: Short Informant Questionnaire on Cognitive Decline in the Elderly, STA: State-Trait Anxiety Inventory, TD: Tremor Dominance Score, TUG: Timed Up and Go, UPDRS: MDS-Unified Parkinson's Disease Rating Scale, UPSIT: University of Pennsylvania Smell Identification Test, VFT: Verbal Fluency Task

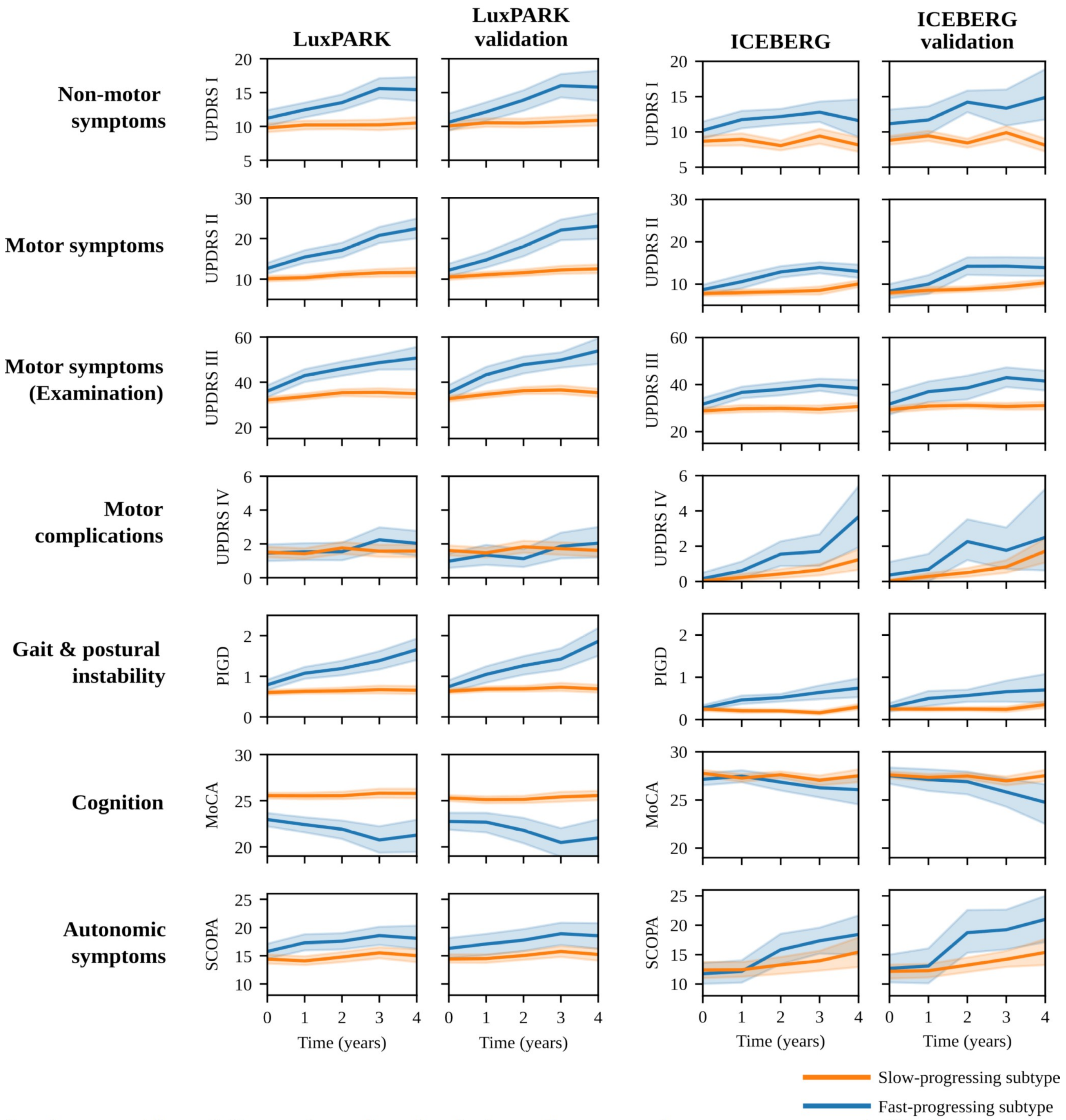

Supplementary Figure 2: Progression trajectories of subtypes for motor and non-motor symptoms (validation)
Progression of motor symptoms (UPDRS II/III/IV, PIGD) and non-motor symptoms (UPDRS I, MoCA, SCOPA) for the slow-progressing subtype (orange) and fast-progressing subtype (blue) for the ICEBERG and LuxPARK cohort. The in-cohort training results and the cross-cohort validation results (models trained on PPMI) are shown side by side. Mean and 95% confidence interval for each subtype are shown.
Abbreviations: MoCA: Montreal Cognitive Assessment, PIGD: Postural Instability and Gait Dysfunction score, SCOPA: Scales for Outcomes in Parkinson's Disease-Autonomic Dysfunction, UPDRS: Unified Parkinson's Disease Rating Scale.

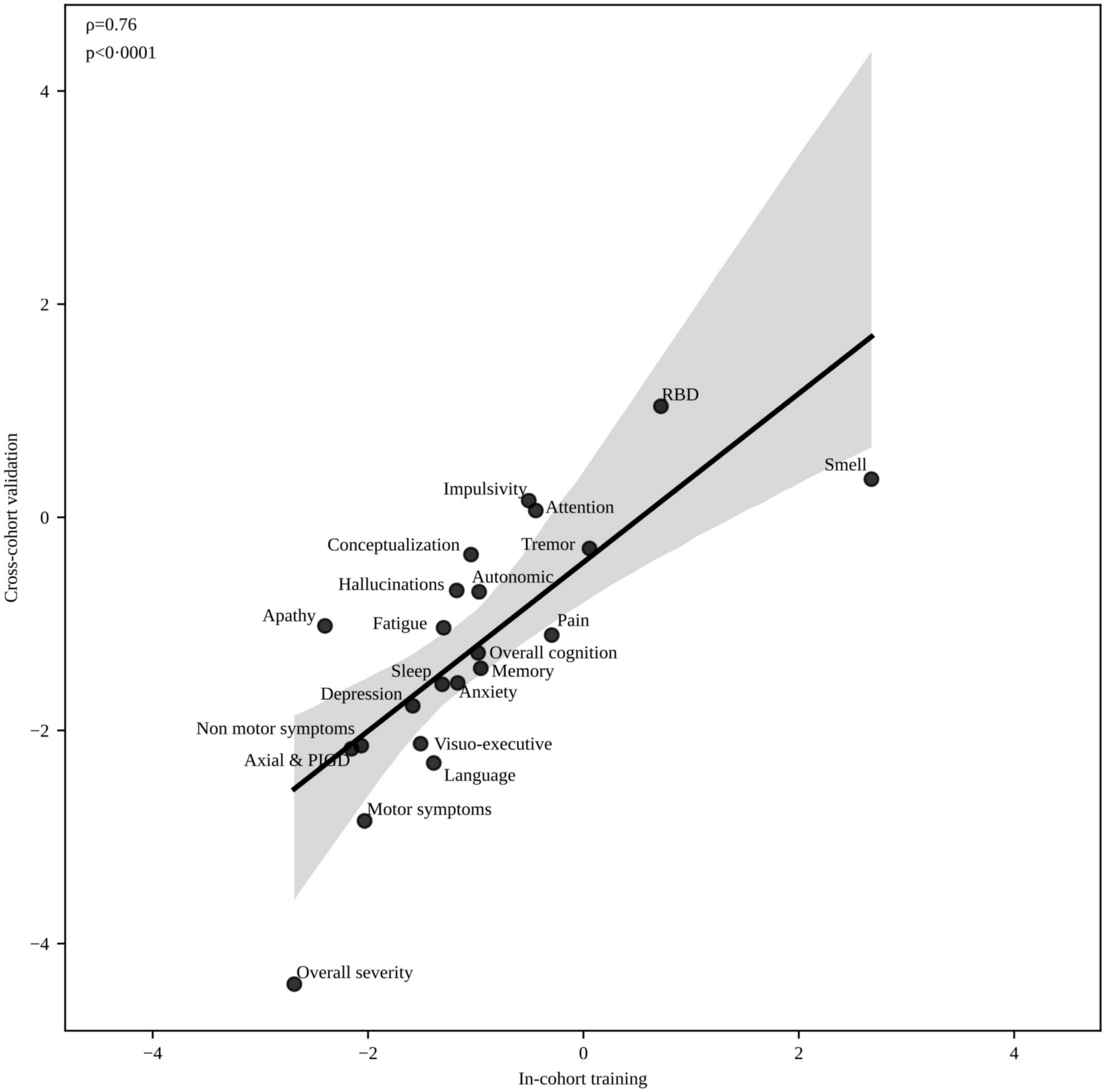

Supplementary Figure 3: Symptom domain progression rate validation

The figure depicts the correlation of standardized mean differences (SMDs) of progression rates calculated for each symptom domain using the in-cohort training approach and the cross-cohort validation approach (i.e. models trained on PPMI). The 95% confidence interval of the regression line is depicted in gray. The Pearson correlation coefficient with corresponding p-value is shown.

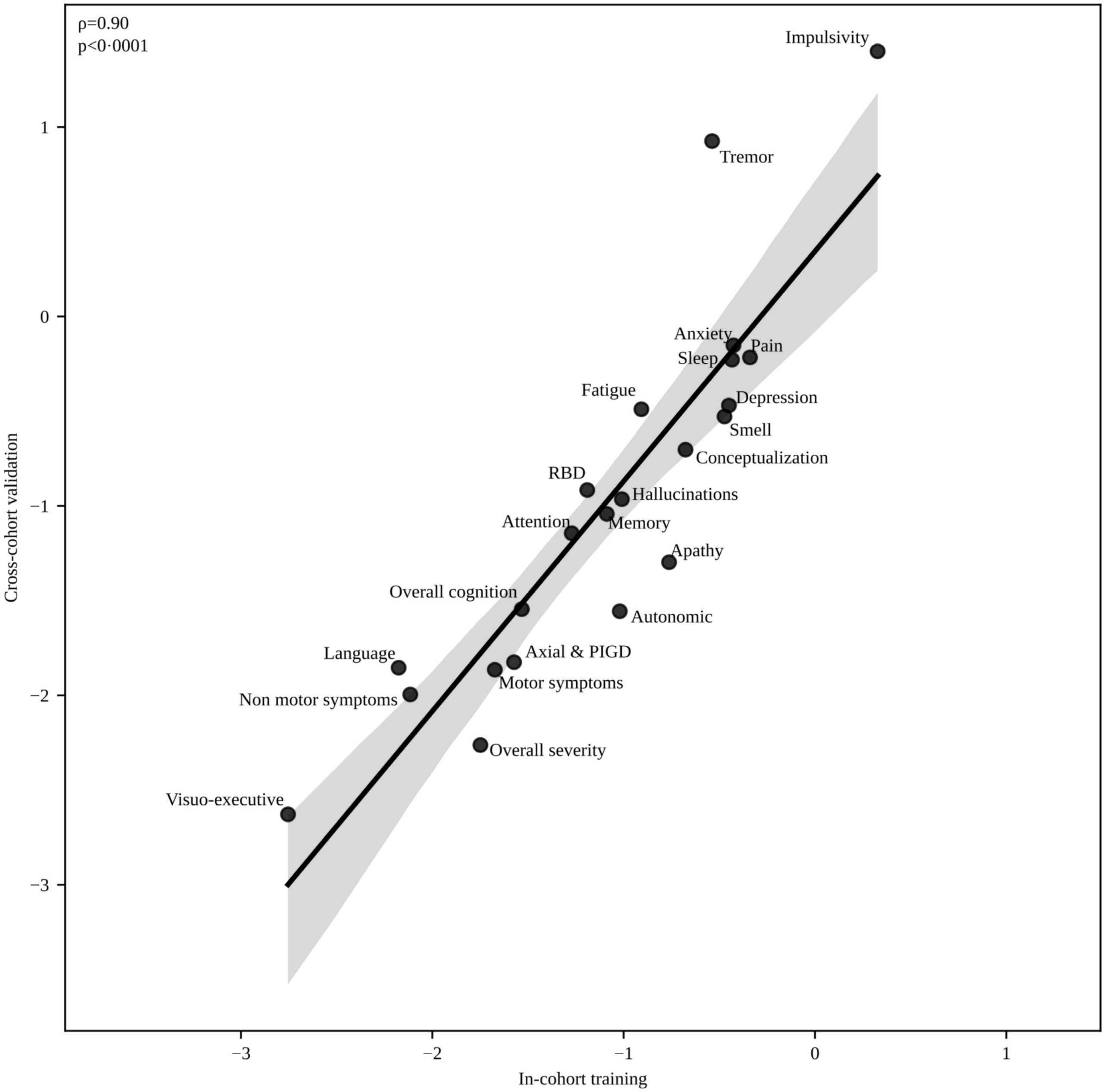

Supplementary Figure 4: Symptom domain baseline associations validation

The figure depicts the correlation of average regression coefficients for baseline outcomes calculated for each symptom domain using the in-cohort training approach and the cross-cohort validation approach (i.e. models trained on PPMI). The 95% confidence interval of the regression line is depicted in gray. The Pearson correlation coefficient with corresponding p-value is shown.

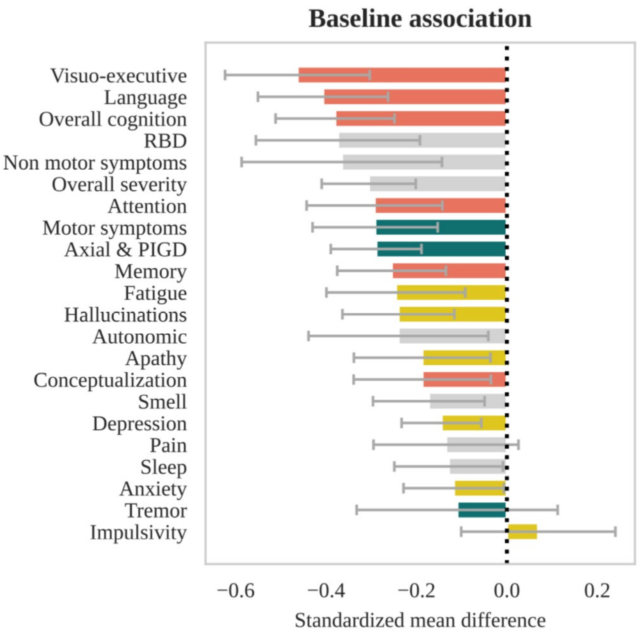

Supplementary Figure 5: Direct comparison of baseline characteristics between progression subtypes
Standardized mean differences (SMD) were calculated for symptom domains at baseline between subtypes. In contrast to Fig. 2C in the main manuscript, baseline outcomes were compared directly without a correction for differences in disease duration. Negative values indicate that more severe symptoms at baseline are associated with the faster subtype. 95% confidence intervals are shown and were corrected for multiple testing.
Abbreviations: PIGD: Postural Instability and Gait Dysfunction score, RBD: REM behavior sleep disorder

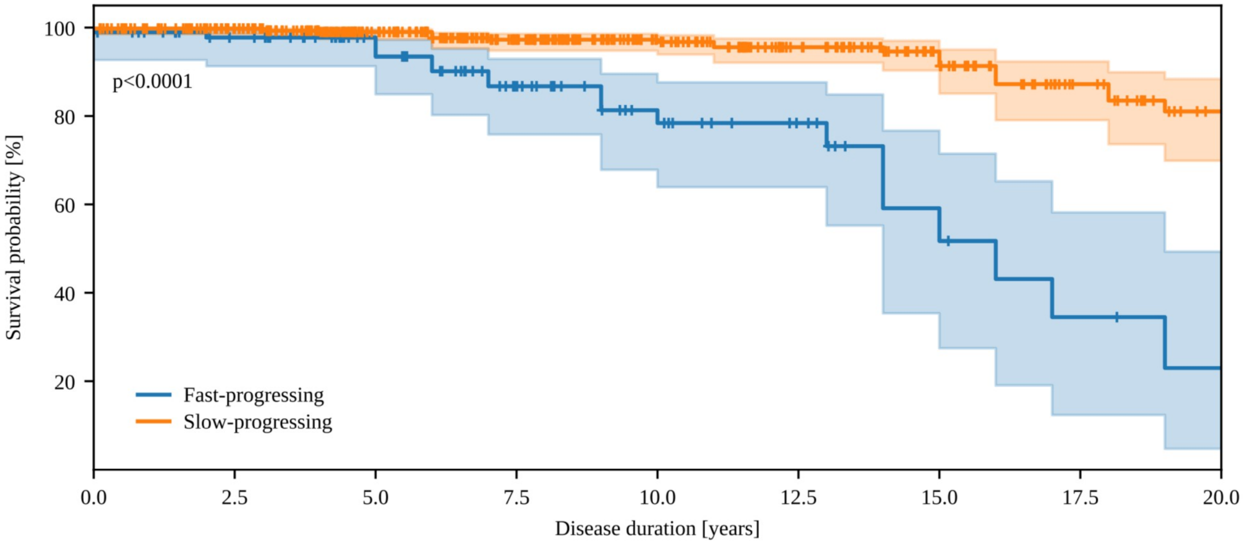

At risk	526	466	366	272	188	133	83	48	29
Censored	33	90	186	271	351	404	446	475	491
Events	2	5	9	18	22	24	32	38	41

Supplementary Figure 6: Survival curves (validation)
Kaplan-Meier estimator for survival probability on the common disease timescale for fast-progressing (blue) and slow-progressing (orange) PwPD in LuxPARK. Right-censored observations are indicated by a small vertical tick. The corresponding p-value for the subtype covariate from the cox proportional hazard model is reported. 95% confidence intervals are shown. The analysis was done using the PPMI-trained model as cross-cohort validation.

Gait parameter	Unit	Description
Gait speed	m/s	The average walking speed.
Heel strike angle	degree	The angle between the toes and the surface when the foot lands.
Landing impact	g	The maximum vertical acceleration during landing of the foot.
Max. foot clearance	cm	The maximum elevation of the foot from the ground during the swing phase.
Max. heel clearance	cm	The maximum elevation of the heel from the ground during the swing phase.
Max. toe clearance	cm	The maximum elevation of the toe from the ground during the swing phase.
Max. lateral excursion	cm	The maximum lateral deviation of the foot in the swing phase, measured from an imaginary line between the foot’s position at start and end of the swing phase.
Stance time (absolute)	s	Duration from initial contact of the foot with the surface until start of next swing phase of the foot.
Stance time (relative)	%	Proportion of stance time divided by the total duration of the stride.
Stride length	cm	The length of one stride.
Stride time	s	Sum of stance time and swing time.
Swing time (absolute)	s	Duration from start of swing until next foot contact with the surface.
Swing time (relative)	%	Proportion of swing time divided by the total duration of the stride.
Toe off angle	degree	The angle between the heel and the surface at the beginning of the swing phase.
Turning angle	degree	The angle between the direction of the last swing phase (imaginary line between foot position at the beginning and end of the swing phase) and the orientation of the foot in the next stance phase.

Supplementary Table 3: Description of digital gait biomarkers.
Gait parameters were calculated as mean of all straight steps from the Timed Up and Go task. Turning steps were excluded from the calculation.
Abbreviations: max: maximum, min: minimum

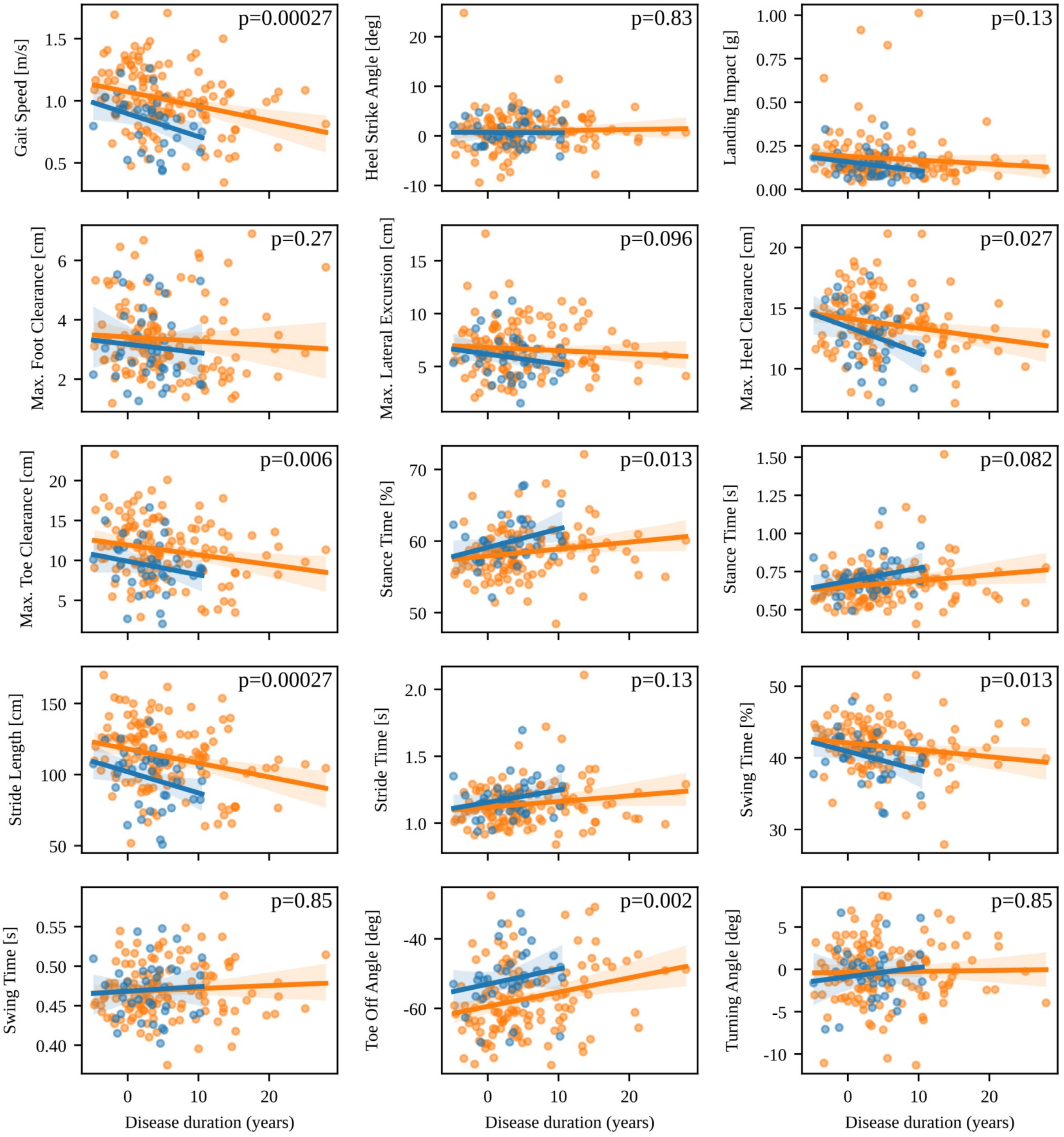

Supplementary Figure 7: Digital gait biomarkers

Correlation of all digital gait markers with disease duration on the common disease timescale for fast-progressing (blue) and slow-progressing (orange) PwPD. The corresponding p-values from the ANCOVA analyses are shown and were corrected for multiple testing. 95% confidence intervals are depicted.
Abbreviations: deg: degree.

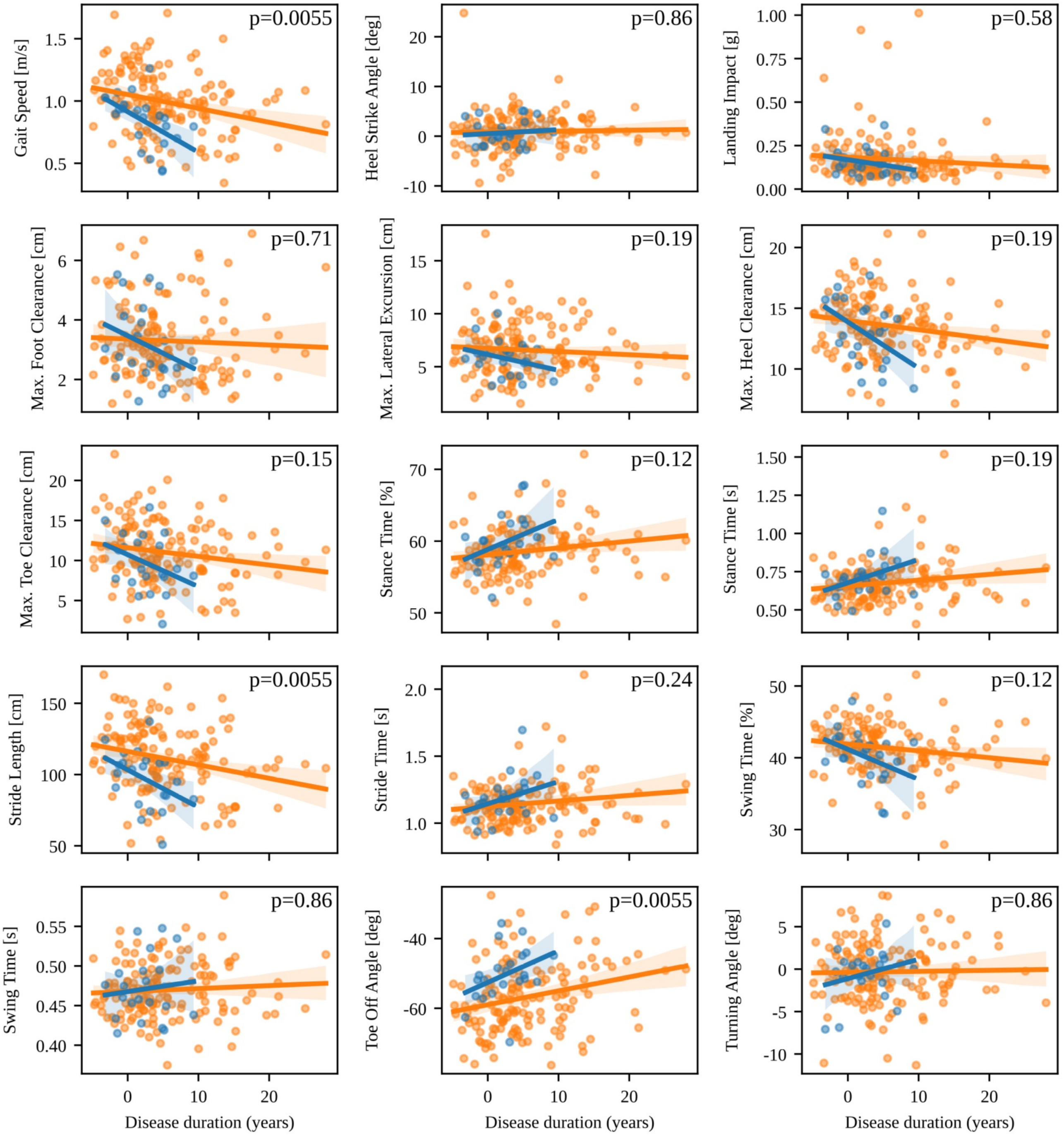

Supplementary Figure 8: Digital gait biomarkers (validation)

Correlation of all digital gait markers with disease duration on the common disease timescale for fast-progressing (blue) and slow-progressing (orange) PwPD. The corresponding p-values from the ANCOVA analyses are shown and were corrected for multiple testing. 95% confidence intervals are shown. The analysis was done using the PPMI-trained model as cross-cohort validation.

Abbreviations: deg: degree.

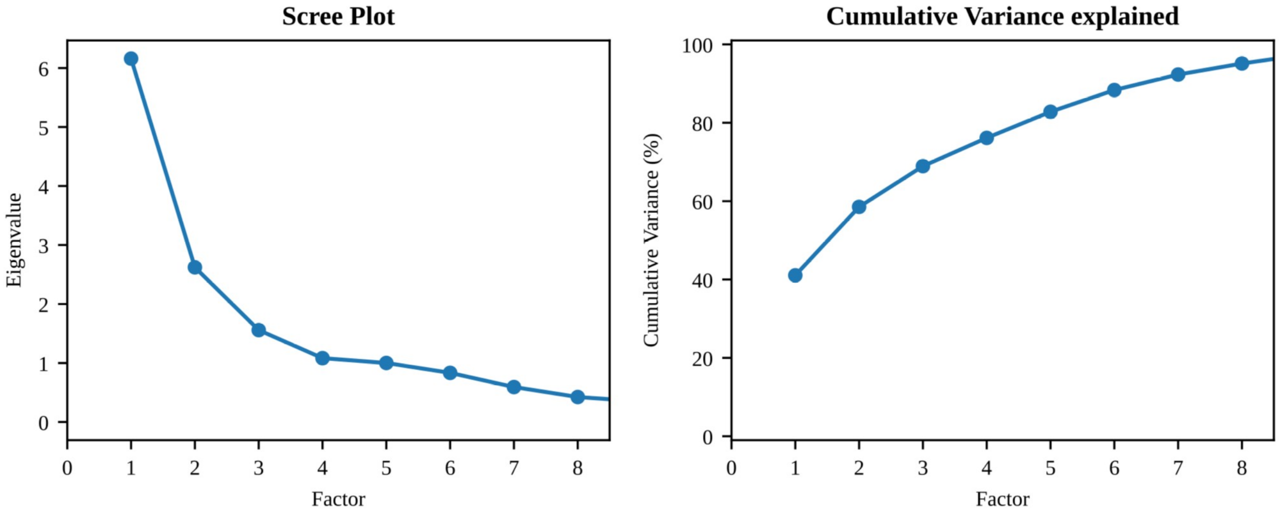

Supplementary Figure 9: Scree plot and variance explained for exploratory factor analysis of digital gait parameters
An exploratory factor analysis was performed using the LuxPARK gait data. The figure shows the Eigenvalues (left) and cumulative variance explained (right) depending on the chosen number of factors. Based on the Scree-Plot, we decided to use three factors.

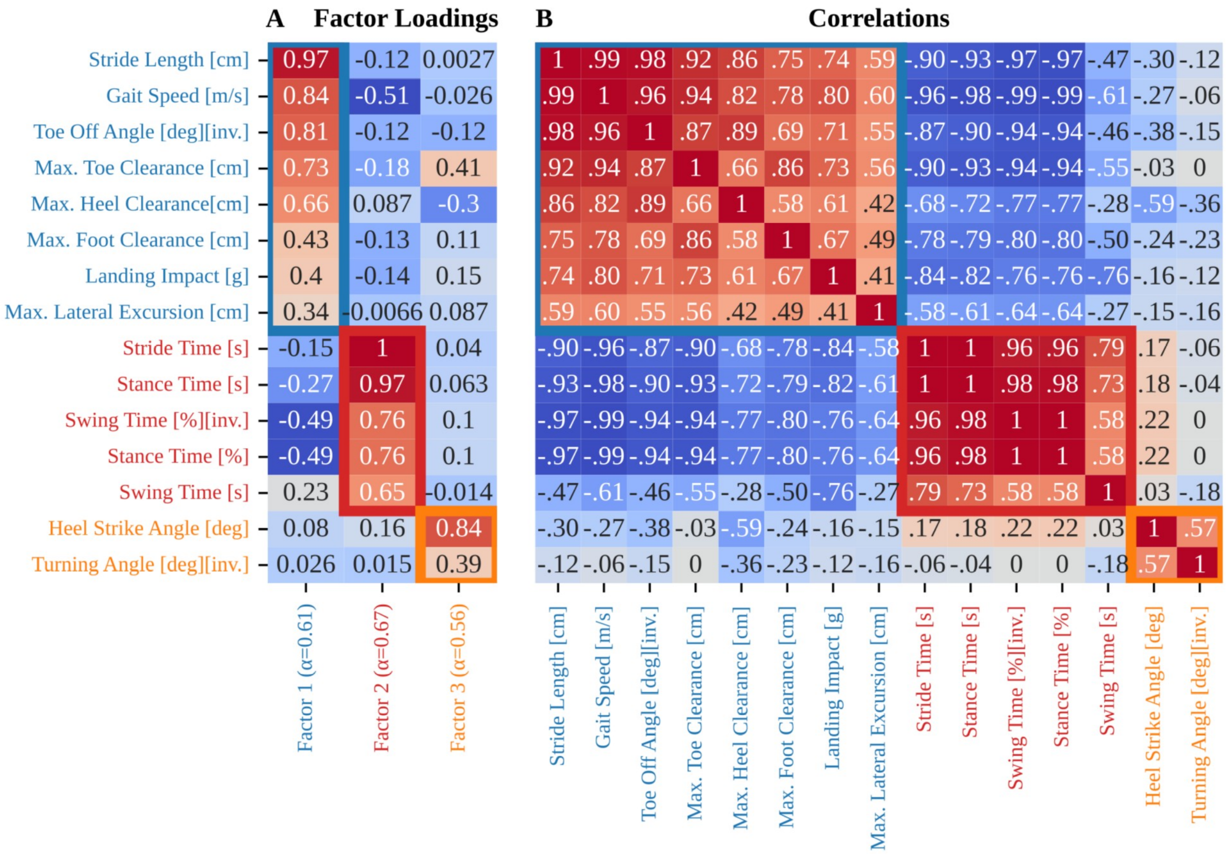

Supplementary Figure 10: Factor analysis and correlation matrix for digital gait data
***A**: Factor loadings for all gait features and the three factors. The choice of three factors was derived from the scree plot displayed in Supplementary Figure 9. The gait features which are mostly determined by the same factor are surrounded by a colored rectangle. The features Toe Off Angle, Swing Time, Turning Angle (marked by "[inv.]") had been inverted to enforce positive factor loadings for better visualization and for the calculation of Cronbach's alpha, which is displayed below each column. The first factor primarily encompassed the spatial sequence of the step, including measures of length and height of the step. In contrast, the second factor was based on the timing of the stance and stride phases of the steps. The third factor incorporated two angles, i.e., the heel strike angle and turning angle. **B**: Correlation heatmap between gait features sorted by the loadings of each factor. Gait features belonging to the same factor are surrounded by a colored rectangle.*

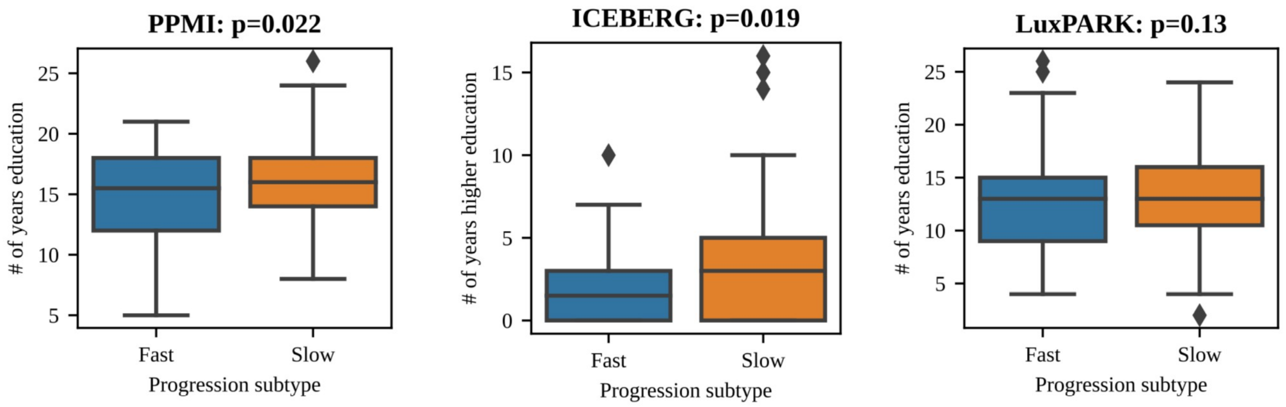

Supplementary Figure 11: Association of PD progression subtypes with educational level

Comparison of education levels between both progression subtypes, i.e., number of education years (PPMI, LuxPARK) and number of years with higher education (ICEBERG). Correction for multiple testing was performed using Benjamini-Hochberg procedure. The boxplots are displayed with a median line, box borders representing the interquartile range (IQR), whiskers extending to 1.5 times the IQR, and outliers depicted as diamonds beyond the whiskers.

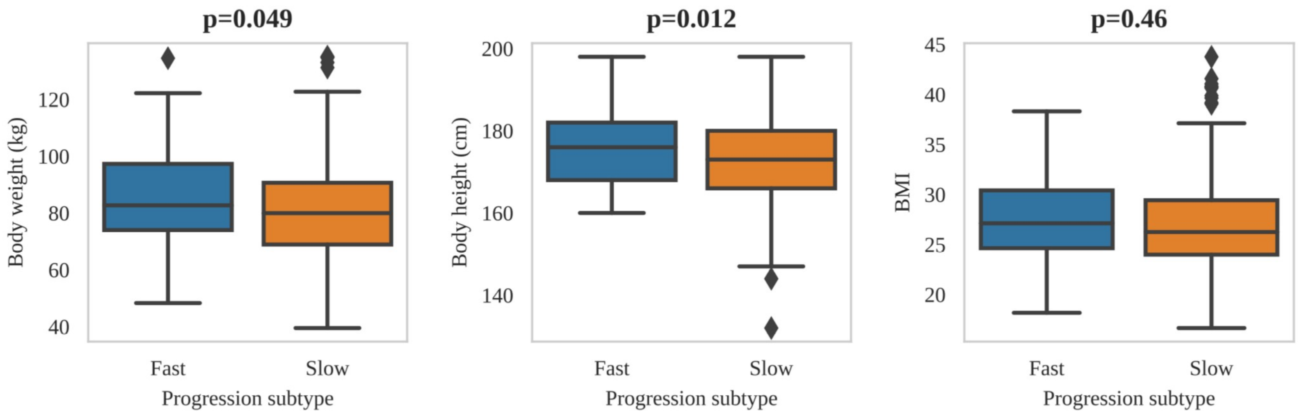

Supplementary Figure 12: Association of PD progression subtypes with body weight, body height and body mass index
All analyzes were performed for PPMI. P-values were corrected for age and sex. Correction for multiple testing was performed using Benjamini-Hochberg procedure. The boxplots are displayed with a median line, box borders representing the interquartile range (IQR), whiskers extending to 1.5 times the IQR, and outliers depicted as diamonds beyond the whiskers.

Blood pressure parameter	p-value (uncorrected)	p-value (corrected for multiple testing)
Systolic blood pressure (supine)	0.45	0.68
Diastolic blood pressure (supine)	0.62	0.74
Systolic blood pressure (standing)	0.44	0.68
Diastolic blood pressure (standing)	0.24	0.68
Systolic blood pressure drop (supine - standing)	0.96	0.96
Diastolic blood pressure drop (supine - standing)	0.38	0.68

Supplementary Table 4: Association of PD progression subtypes with blood pressure

All analyzes were performed for PPMI. P-values were corrected for age and sex. Correction for multiple testing was performed using Benjamini-Hochberg procedure.

Comorbidity group	p-value (uncorrected)	p-value (corrected for multiple testing)
Pulmonary	*0.033*	0.26
Ophthalmological	*0.035*	0.26
Metabolic/Endocrine	0.16	0.81
Dermatological	0.26	0.87
Psychiatric	0.46	0.87
Cardiovascular	0.47	0.87
Musculoskeletal	0.48	0.87
Allergy/immunologic	0.56	0.87
Other	0.67	0.87
Gastrointestinal	0.68	0.87
Gynecological/Urologic	0.71	0.87
Renal	0.75	0.87
Hemato/Lymphatic	0.75	0.87
ENT	0.86	0.92
Hepatobiliary	0.96	0.96

Supplementary Table 5: Association of PD progression subtypes with diagnoses groups
All analyzes were performed for PPMI. P-values were corrected for age and sex. Correction for multiple testing was performed using Benjamini-Hochberg procedure.

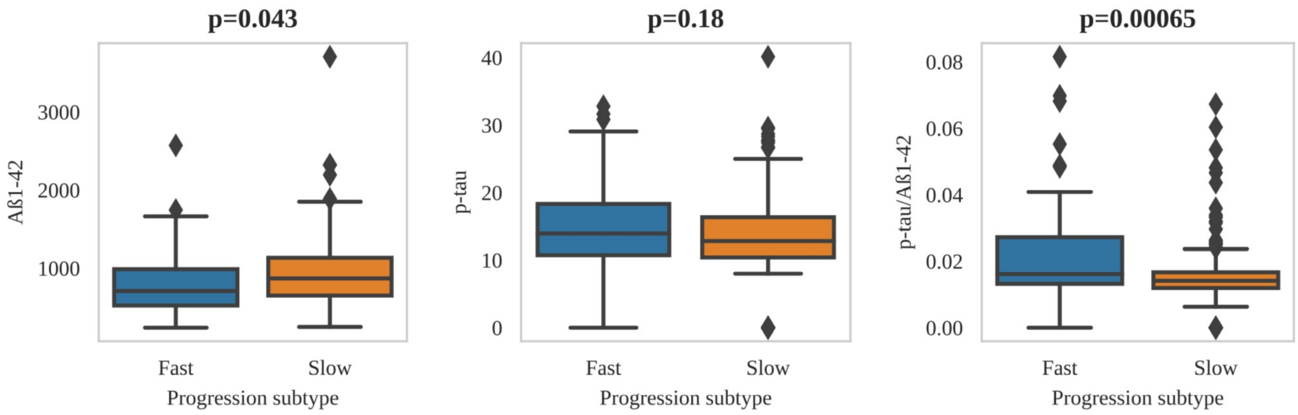

Supplementary Figure 13: Association of PD progression subtypes with Alzheimer's disease pathology cerebrospinal fluid biomarkers

All analyzes were performed for PPMI. P-values were corrected for age and sex. Correction for multiple testing was performed using Benjamini-Hochberg procedure. The boxplots are displayed with a median line, box borders representing the interquartile range (IQR), whiskers extending to 1.5 times the IQR, and outliers depicted as diamonds beyond the whiskers.

Comedication group	p-value (uncorrected)	p-value (corrected for multiple testing)
Calcium Channel Blockers	0.18	0.95
Beta Antagonists	0.31	0.95
NSARs (without ASS)	0.44	0.95
NSAR	0.53	0.95
Ibuprofen	0.63	0.95
Statins	0.73	0.95
ASS	0.83	0.95
Contraceptives	1.0	1.0

Supplementary Table 6: Association of PD progression subtypes with specific medications.
All analyzes were performed for PPMI. P-values were corrected for age and sex. Correction for multiple testing was performed using Benjamini-Hochberg procedure.

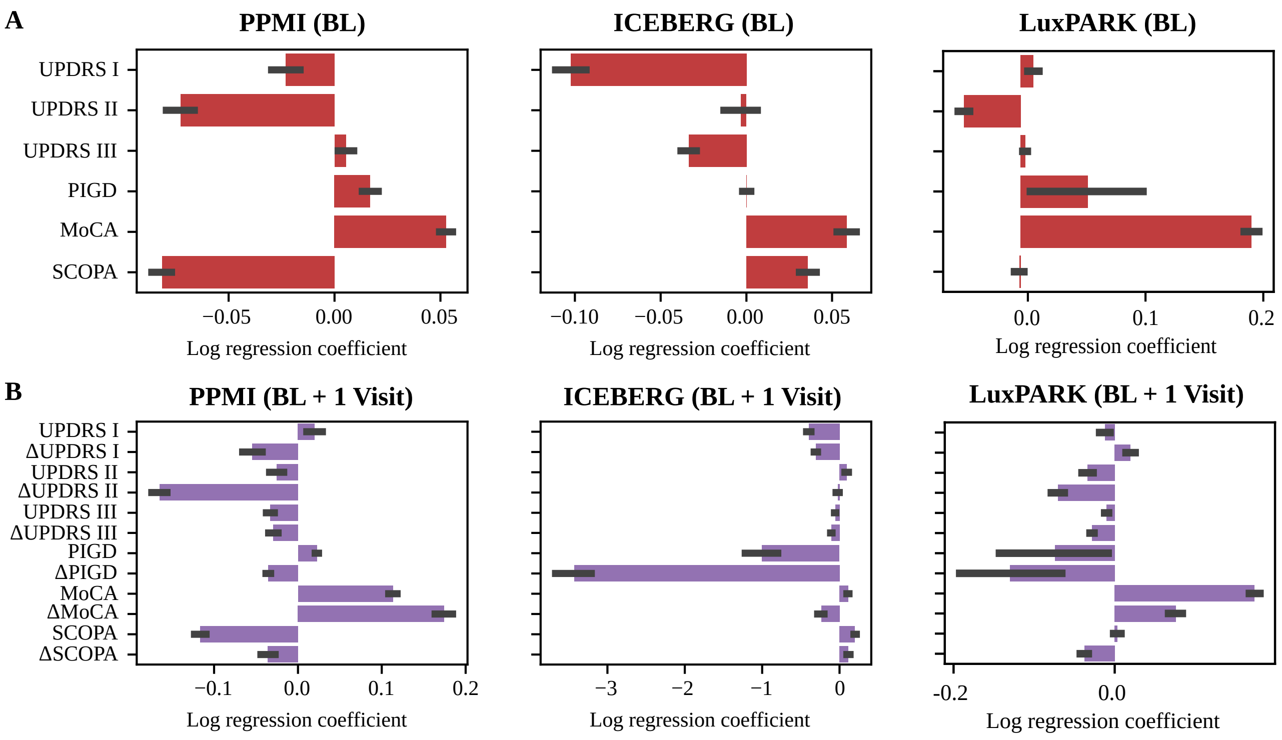

Supplementary Figure 14: Coefficients of logistic regression models for subtype predictions
*Logistic regression coefficients with 95% confidence interval using baseline data (**A**, red) or baseline data with one follow up visit (**B**, purple) from the predictive logistic regression model are shown. Higher outcome scores together with positive coefficients influence the prediction towards the slow-progressing subtype and towards the fast-progressing subtype if coefficients are negative.*
Abbreviations: BL: Baseline, Δ: Difference from first visit to baseline, MoCA: Montreal Cognitive Assessment, PIGD: Postural Instability and Gait Dysfunction score, SCOPA: Scales for Outcomes in Parkinson's Disease-Autonomic Dysfunction, UPDRS: Unified Parkinson's Disease Rating Scale.

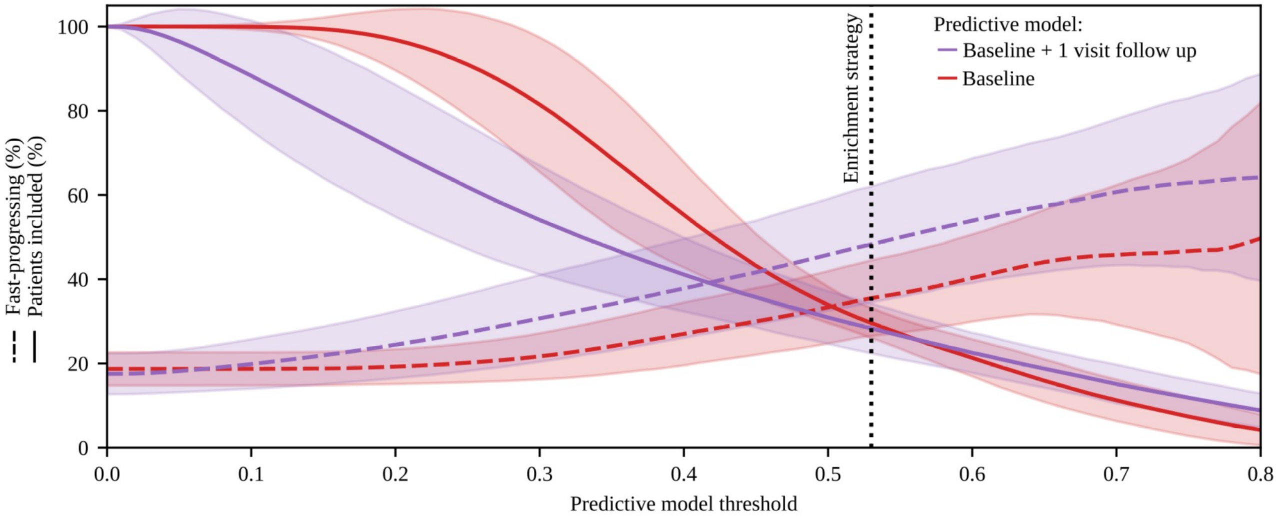

Supplementary Figure 15: Trade-off between enriching fast-progressing PwPD and number of eligible PwPD
Depending on the chosen threshold applied to the logistic predictive model, the percentage of fast-progressing PwPD (dashed lines) and the percentage of PwPD being still eligible for study inclusion (solid lines) changes. Curves are displayed for the predictive model using baseline data (red) and the predictive model using data from baseline and one follow-up visit (purple). The vertical dotted line indicates the threshold of the predictive model, where still 30% of PwPD are eligible for study inclusion and a 47% enrichment of fast-progressing PwPD is achieved using the predictive model based on baseline data and one follow-up visit. 95% confidence intervals are depicted.

**% fast-progressing PwPD**	**PPMI**	**ICEBERG**	**LuxPARK**
No enrichment	18 %	27 %	26 %
BL enrichment (in-cohort)	36 %	43 %	47 %
BL+FU enrichment (in-cohort)	47 %	65 %	53 %
BL enrichment (cross-cohort)	-	27 %	38 %
BL+FU enrichment (cross-cohort)	-	38 %	41 %

Supplementary Table 7:Enrichment of fast-progressing PwPD using different predictive models
Predictive models were based on baseline data (BL) or baseline with one follow up visit (BL+FU). Models were trained on the same data set (in-cohort), or PPMI and cross-validated on ICEBERG/LuxPARK (cross-cohort). Using the predictive models and an appropriate threshold for the three cohorts (PPMI, ICEBERG, LuxPARK), the reported fractions of fast-progressing PwPD can be achieved in a study cohort by still allowing inclusion of 30% of all PwPD. The *“No enrichment” row reports the fraction of fast-progressing in the original cohorts without applying any enrichment strategy.*
Abbreviations: BL: baseline, FU: follow up

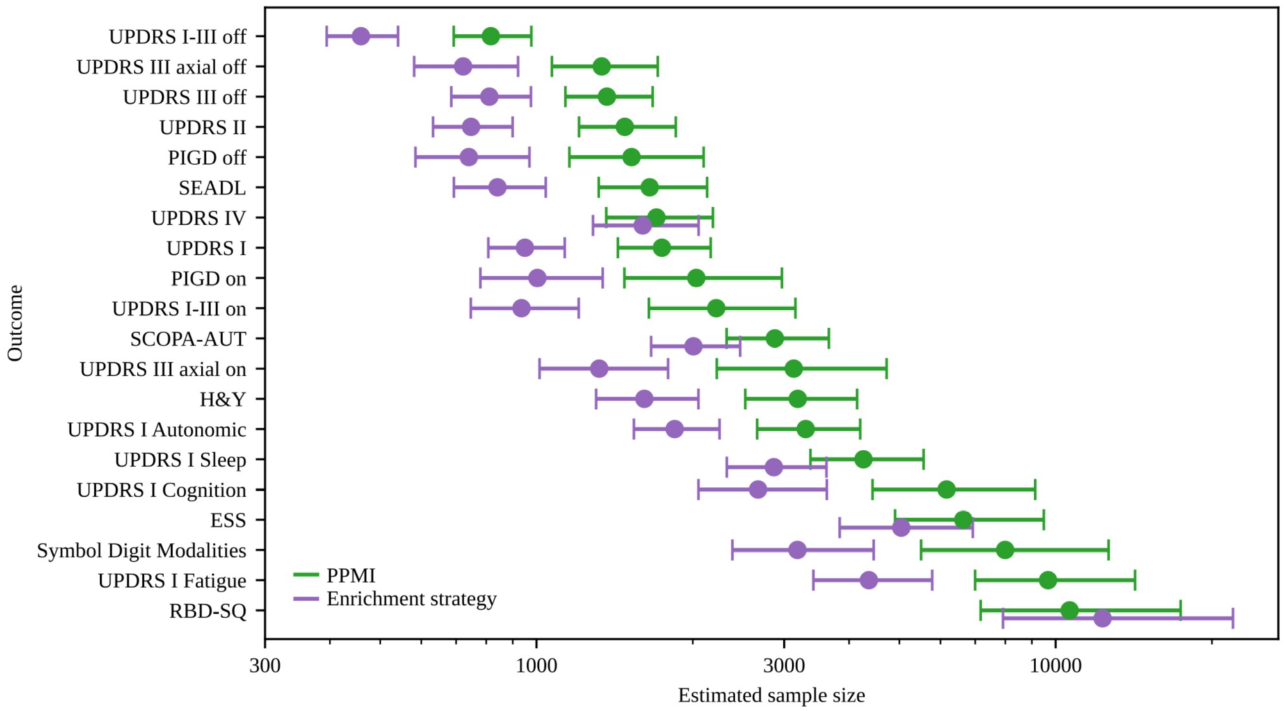

Supplementary Figure 16: Subtype enrichment for sample size reduction in clinical trials using different outcomes
Required sample sizes for a clinical trial depending on the clinical score used as primary outcome in the trial. The required sample sizes calculated using the default PPMI cohort are shown in green. The required sample sizes using the enrichment strategy from Fig. 5A are shown in purple. Mean estimate and 95% confidence intervals are shown. Sample sizes were calculated for all outcomes listed in Supplementary Table 2 and assessed in PPMI. From these outcomes, only the 20 outcomes with lowest required sample sizes are shown. The estimated sample size is shown on a logarithmic axis.
Abbreviations: ESS: Epworth Sleepiness Scale, H&Y: Hoehn & Yahr, PIGD: Postural Instability and Gait Disorder score, RBD-SQ: REM Sleep Behavior Disorder Screening Questionnaire, SCOPA-AUT: Scales for Outcomes in Parkinson’s Disease-Autonomic Dysfunction, SEADL: Schwab and England Activities of Daily Living Scale, UPDRS: Unified Parkinson's Disease Rating Scale.

**% sample size reduction**	**PPMI**	**ICEBERG**	**LuxPARK**
BL (in-cohort)	30 %	32 %	44 %
BL+FU (in-cohort)	43 %	56 %	52 %
BL (cross-cohort)	-	0 %	28 %
BL+FU (cross-cohort)	-	36 %	34 %

Supplementary Table 8: Sample size reduction in a simulated clinical trial using different predictive models for enrichment of fast-progressing PwPD

Predictive models were based on baseline data (BL) or baseline with one follow up visit (BL+FU). Models were trained on the same data set (in-cohort) or PPMI and cross-validated on ICEBERG/LuxPARK (cross-cohort). Using the predictive models and an appropriate threshold to the three cohorts (PPMI, ICEBERG, LuxPARK), the reported sample size reductions can be achieved by still allowing inclusion of 30% of all PwPD.

Abbreviations: BL: baseline, FU: follow up

	PPMI	ICEBERG	LuxPARK
number of clusters	2	2	2
learning rate	0.0001	0.001	0.0001
batch size	16	32	16
number of nodes (first hidden layer)	64	32	32
number of nodes (second hidden layer)	1	1	2

Supplementary Table 9: Results of VaDER hyperparameter optimization for PPMI, ICEBERG and LuxPARK

Model	Parameter	Grid values	Baseline PPMI / ICEBERG / LuxPARK	Baseline + FU PPMI / ICEBERG / LuxPARK
Logistic regression	Lambda	0.001, 0.01, 0.1, 1, 10, 100, 1000	0.01 / 0.01 / 100	0.01 / 10 / 10
Random Forest	Bootstrap	True, False	True / True / True	True / True / True
	Maximum depth	10, 20, 30, 40	40 / 20 / 20	20 / 10 / 10
	Minimum samples per leaf	1, 2, 4	4 / 1 / 1	4 / 2 / 1
	Minimum samples per split	2, 5, 10	10 / 5 / 10	10 / 10 / 10
	Number of Estimators	200, 600, 800, 1000, 1200, 1400, 1600, 1800, 2000	800 / 1400 / 1800	1800 / 2000 / 200
XGBoost	Minimum Child Weight	1, 5, 10	10 / 1 / 1	5 / 1 / 5
	Gamma	0.5, 1, 1.5, 2, 5	2 / 0.5 / 5	0.5 / 1.5 / 1.5
	Subsample	0.6, 0.8, 1.0	0.8 / 0.6 / 0.8	0.8 / 0.6 / 1.0
	Colsample by tree	0.6, 0.8, 1.0	0.8 / 0.6 / 0.6	0.6 / 1.0 / 1.0
	Maximum Depth	3, 4, 5	4 / 4 / 3	4 / 3 / 3

Supplementary Table 10: Predictive model hyperparameter optimization
Hyperparameter space used for hyperparameter optimization. For Logistic regression, L2 penalization and hyperparameter grid search was used. For Random Forest and XGBoost, randomized hyperparameter search with 50 samples was used. Repeated stratified k-fold cross validation was performed using 5 splits and 20 repeats. Results of hyperparameter training for the baseline-only and baseline with one visit follow-up models are presented.
Abbreviations: FU: follow up

Forest plots for symptom domain progression (in cohort)

Forest plot for progression characteristics of symptom domain Axial & PIGD

Study	Slow progressing Total	Slow progressing Mean	Slow progressing SD	Fast progressing Total	Fast progressing Mean	Fast progressing SD	SMD	95%−CI
**Cohort = PPMI**								
UPDRS III axial off	335	0.16	0.0777	74	0.52	0.2251	−3.04	[−3.37; −2.71]
UPDRS III axial on	300	0.09	0.0855	66	0.43	0.2922	−2.33	[−2.64; −2.01]
PIGD off	335	0.13	0.0917	74	0.61	0.3265	−2.97	[−3.30; −2.65]
PIGD on	300	0.10	0.0823	66	0.48	0.3223	−2.49	[−2.81; −2.16]
**Random effects model**	**1270**			**280**			**−2.71**	**[−3.05; −2.36]**
**Cohort = ICEBERG**								
UPDRS III axial off	111	0.09	0.0004	42	0.36	0.1002	−5.16	[−5.85; −4.48]
UPDRS III axial on	105	0.10	0.0011	41	0.17	0.1309	−1.02	[−1.40; −0.64]
GABS Examination	112	0.04	0.0191	42	0.12	0.1273	−1.26	[−1.64; −0.87]
GABS Questionaire	112	0.03	0.0138	42	0.15	0.0932	−2.41	[−2.86; −1.96]
PIGD off	111	0.06	0.0154	42	0.24	0.0735	−4.52	[−5.14; −3.89]
PIGD on	105	0.07	0.0042	41	0.23	0.1364	−2.22	[−2.67; −1.78]
**Random effects model**	**656**			**250**			**−2.75**	**[−4.10; −1.39]**
**Cohort = LuxPARK**								
UPDRS III axial on	380	0.13	0.0510	140	0.30	0.1008	−2.44	[−2.68; −2.19]
FOGAC	63	0.11	0.0767	17	0.11	0.1963	0.03	[−0.51; 0.57]
FOGQ	166	0.17	0.0954	64	0.31	0.2075	−0.97	[−1.28; −0.67]
NFOGQ	19	0.24	0.0939	5	0.91	1.3814	−1.10	[−2.14; −0.05]
PDQ39 Mobility	407	0.18	0.0835	152	0.44	0.1897	−2.12	[−2.34; −1.89]
PIGD on	380	0.14	0.0843	140	0.33	0.1427	−1.89	[−2.11; −1.66]
TuG	154	0.03	0.0318	42	0.05	0.0269	−0.77	[−1.12; −0.42]
**Random effects model**	**1569**			**560**			**−1.35**	**[−2.00; −0.70]**
**Random effects model**	**3495**			**1090**			**−2.15**	**[−2.77; −1.54]**

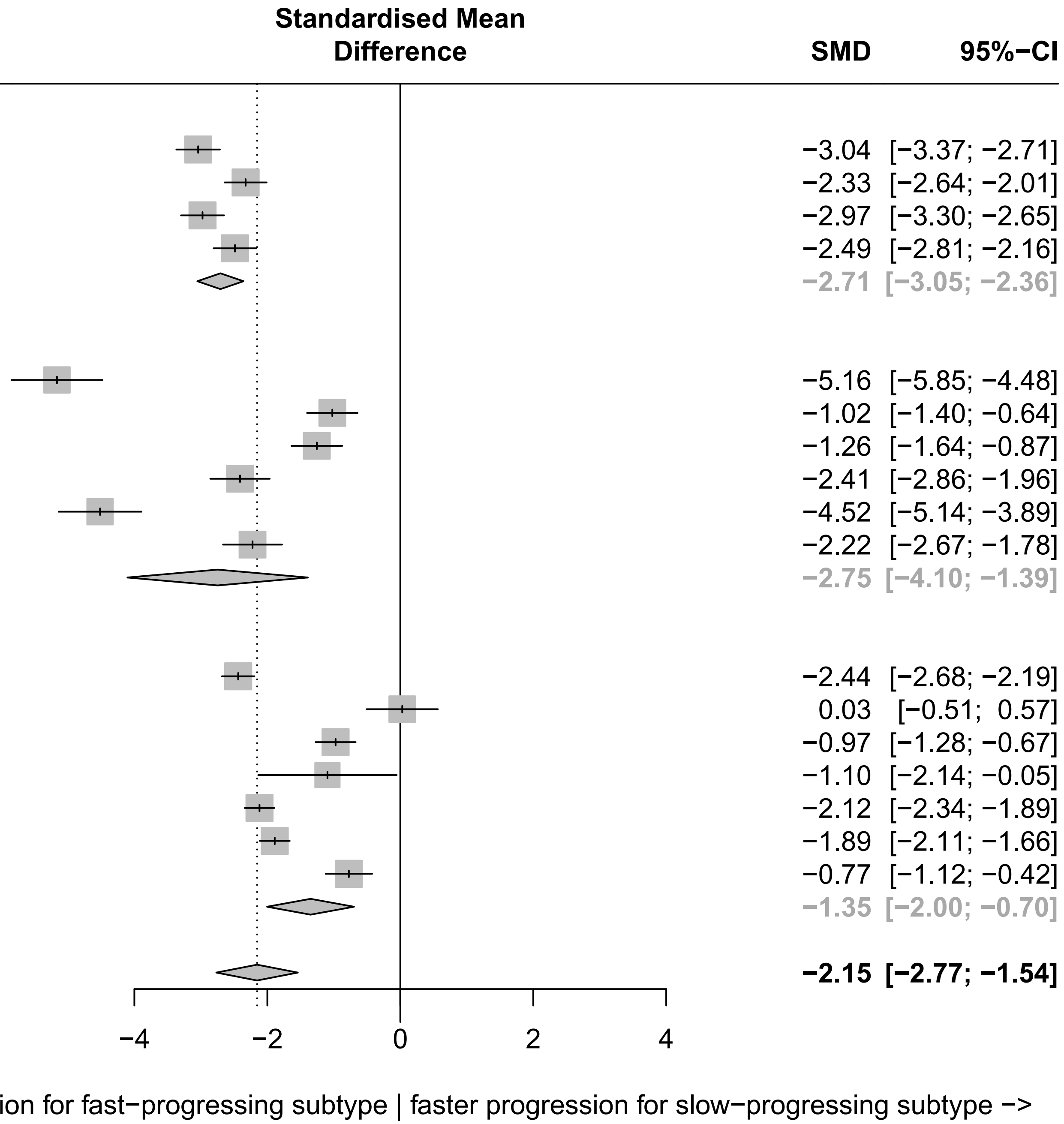

Three−level metaanalysis using random effects to calculate an overall SMD estimate for Axial & PIGD across cohorts.
The dashed line indicates the overall mean SMD estimate. The solid line indicates no effect.

Forest plot for progression characteristics of symptom domain Depression

Study	Slow progressing Total	Slow progressing Mean	Slow progressing SD	Fast progressing Total	Fast progressing Mean	Fast progressing SD	SMD	95%−CI
**Cohort = LuxPARK**								
BDI	406	0.04	0.0176	149	0.14	0.0358	−4.19	[−4.50; −3.88]
NMSQ Depression	408	1.04	0.5080	152	2.15	0.2609	−2.43	[−2.67; −2.20]
PDQ39 Depression	407	0.06	0.0499	152	0.18	0.0605	−2.31	[−2.54; −2.08]
UPDRS I Depression	408	0.84	0.1665	153	1.53	0.4505	−2.51	[−2.75; −2.28]
**Random effects model**	**1629**			**606**			**−2.86**	**[−3.72; −1.99]**
**Cohort = PPMI**								
GDS	324	0.05	0.1681	73	0.35	0.1441	−1.78	[−2.06; −1.50]
UPDRS I Depression	335	1.16	1.4484	74	2.83	2.1275	−1.04	[−1.31; −0.78]
**Random effects model**	**659**			**147**			**−1.41**	**[−2.13; −0.69]**
**Cohort = ICEBERG**								
HADS depression	112	0.10	0.0186	42	0.16	0.0578	−1.60	[−1.99; −1.20]
NMSQ Depression	112	2.00	0.4071	42	1.42	1.4046	0.72	[0.35; 1.08]
UPDRS I Depression	112	1.54	0.5269	42	0.83	1.2172	0.91	[0.54; 1.28]
**Random effects model**	**336**			**126**			**0.01**	**[−1.56; 1.59]**
**Random effects model**	**2624**			**879**			**−1.58**	**[−2.64; −0.53]**

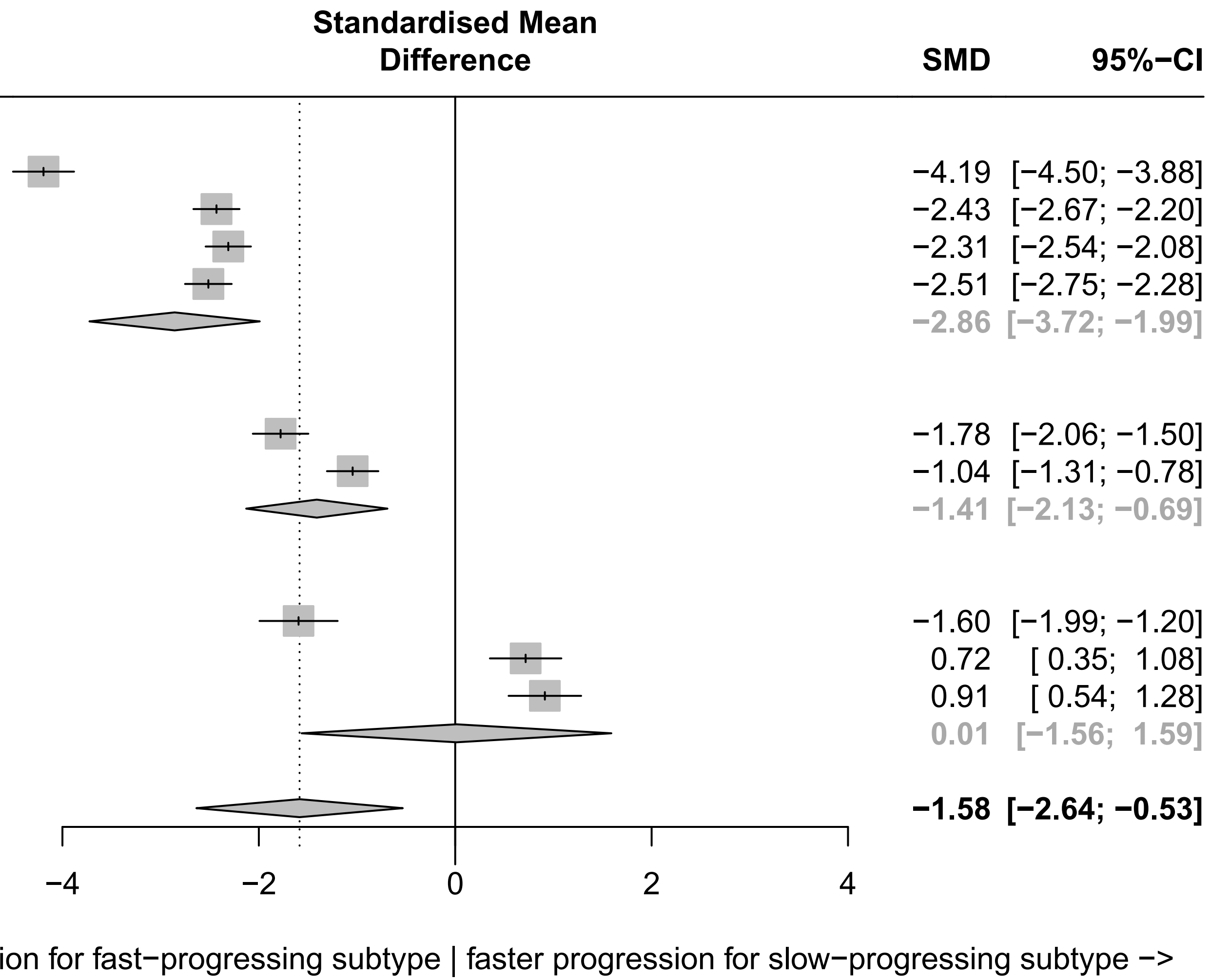

Three−level metaanalysis using random effects to calculate an overall SMD estimate for Depression across cohorts.
The dashed line indicates the overall mean SMD estimate. The solid line indicates no effect.

Forest plot for progression characteristics of symptom domain Overall severity

Study	Slow progressing Total	Slow progressing Mean	Slow progressing SD	Fast progressing Total	Fast progressing Mean	Fast progressing SD	SMD	95%−CI
**Cohort = ICEBERG**								
CGIS	112	1.07	0.0666	42	2.39	0.8408	−2.99	[−3.48; −2.50]
H&Y	112	−0.40	0.3209	42	0.57	0.1069	−3.45	[−3.97; −2.92]
SEADL	112	0.05	0.0260	42	0.13	0.1255	−1.17	[−1.55; −0.79]
UPDRS I−III off	111	0.14	0.0092	42	0.30	0.0654	−4.65	[−5.28; −4.01]
UPDRS I−III on	105	0.11	0.0103	41	0.23	0.0362	−5.75	[−6.51; −4.99]
**Random effects model**	**552**			**209**			**−3.58**	**[−5.10; −2.06]**
**Cohort = LuxPARK**								
FAQ	354	0.09	0.0549	124	0.30	0.1342	−2.47	[−2.73; −2.22]
H&Y	408	2.37	1.5849	153	2.93	2.3835	−0.31	[−0.49; −0.12]
PDQ39	403	0.10	0.0470	149	0.28	0.1213	−2.33	[−2.56; −2.10]
UPDRS I−III on	377	0.14	0.0350	138	0.34	0.1151	−2.99	[−3.26; −2.72]
**Random effects model**	**1542**			**564**			**−2.02**	**[−3.18; −0.86]**
**Cohort = PPMI**								
H&Y	335	4.88	1.8738	74	4.44	3.7381	0.19	[−0.06; 0.44]
SEADL	335	0.14	0.0788	74	0.58	0.2890	−3.09	[−3.42; −2.76]
UPDRS I−III off	335	0.26	0.1092	74	0.64	0.1259	−3.38	[−3.72; −3.04]
UPDRS I−III on	300	0.11	0.0952	66	0.46	0.2094	−2.77	[−3.10; −2.44]
**Random effects model**	**1305**			**288**			**−2.26**	**[−3.88; −0.63]**
**Random effects model**	**3399**			**1061**			**−2.68**	**[−3.55; −1.82]**

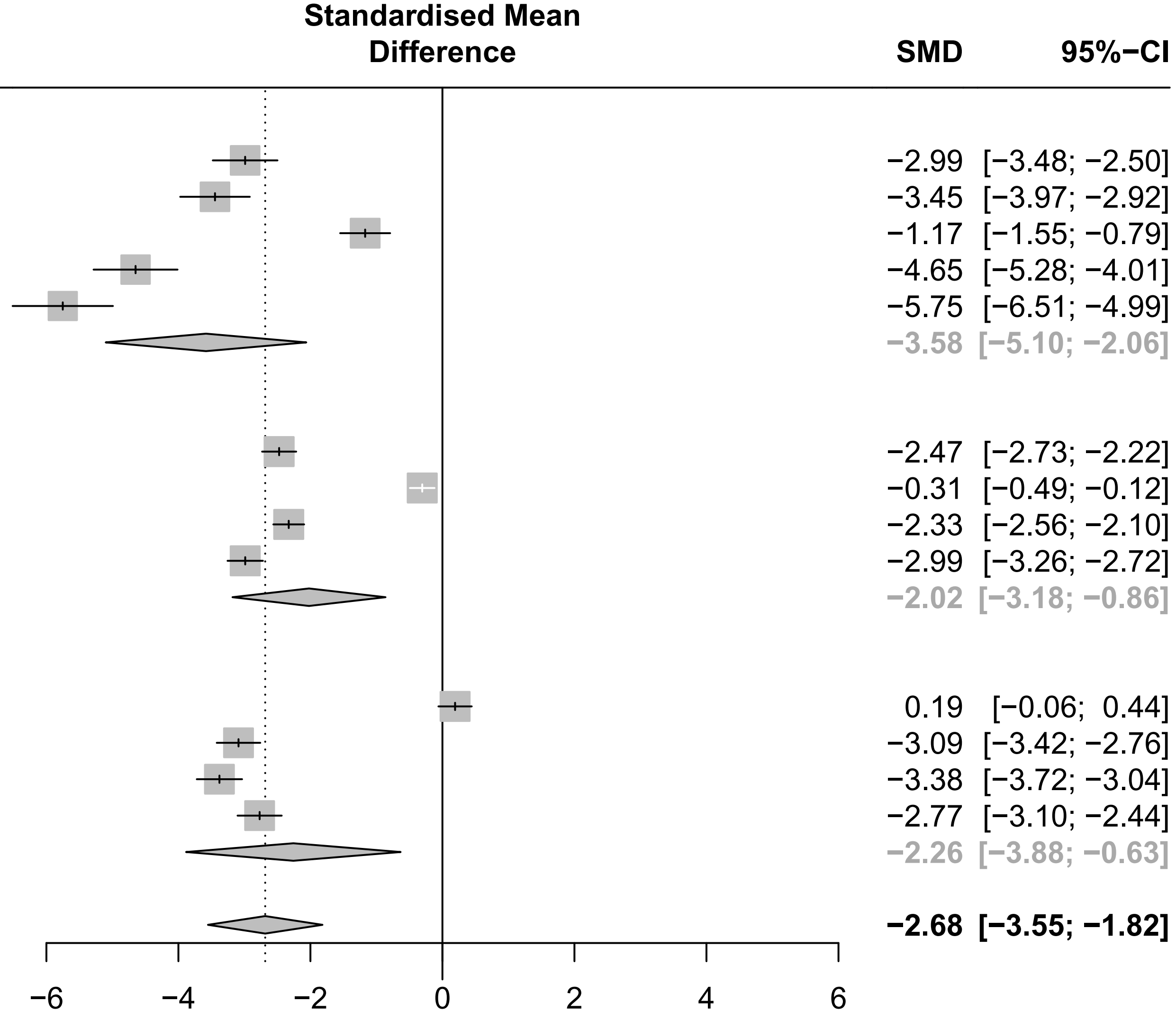

<- Faster progression for fast−progressing subtype | faster progression for slow−progressing subtype ->

Three−level metaanalysis using random effects to calculate an overall SMD estimate for Overall severity across cohorts. The dashed line indicates the overall mean SMD estimate. The solid line indicates no effect.

Forest plot for progression characteristics of symptom domain Apathy

Study	Slow progressing Total	Slow progressing Mean	Slow progressing SD	Fast progressing Total	Fast progressing Mean	Fast progressing SD	SMD	95%−CI
**Cohort = ICEBERG**								
DAS	103	0.08	0.0179	38	0.11	0.0428	−1.11	[−1.50; −0.71]
SAS	104	0.08	0.0303	41	0.12	0.0322	−1.39	[−1.78; −0.99]
UPDRS I Apathy	112	1.18	0.4818	42	1.44	0.8770	−0.41	[−0.77; −0.06]
**Random effects model**	**319**			**121**			**−0.96**	**[−1.54; −0.39]**
**Cohort = LuxPARK**								
SAS	405	0.05	0.0480	153	0.12	0.0369	−1.52	[−1.73; −1.31]
UPDRS I Apathy	408	0.73	0.0431	153	1.86	0.2459	−8.48	[−9.01; −7.95]
**Random effects model**	**813**			**306**			**−4.99**	**[−11.81; 1.82]**
**Cohort = PPMI**								
UPDRS I Apathy	335	2.14	1.2773	74	4.63	2.7266	−1.52	[−1.79; −1.25]
**Random effects model**	**1467**			**501**			**−2.40**	**[−4.80; −0.00]**

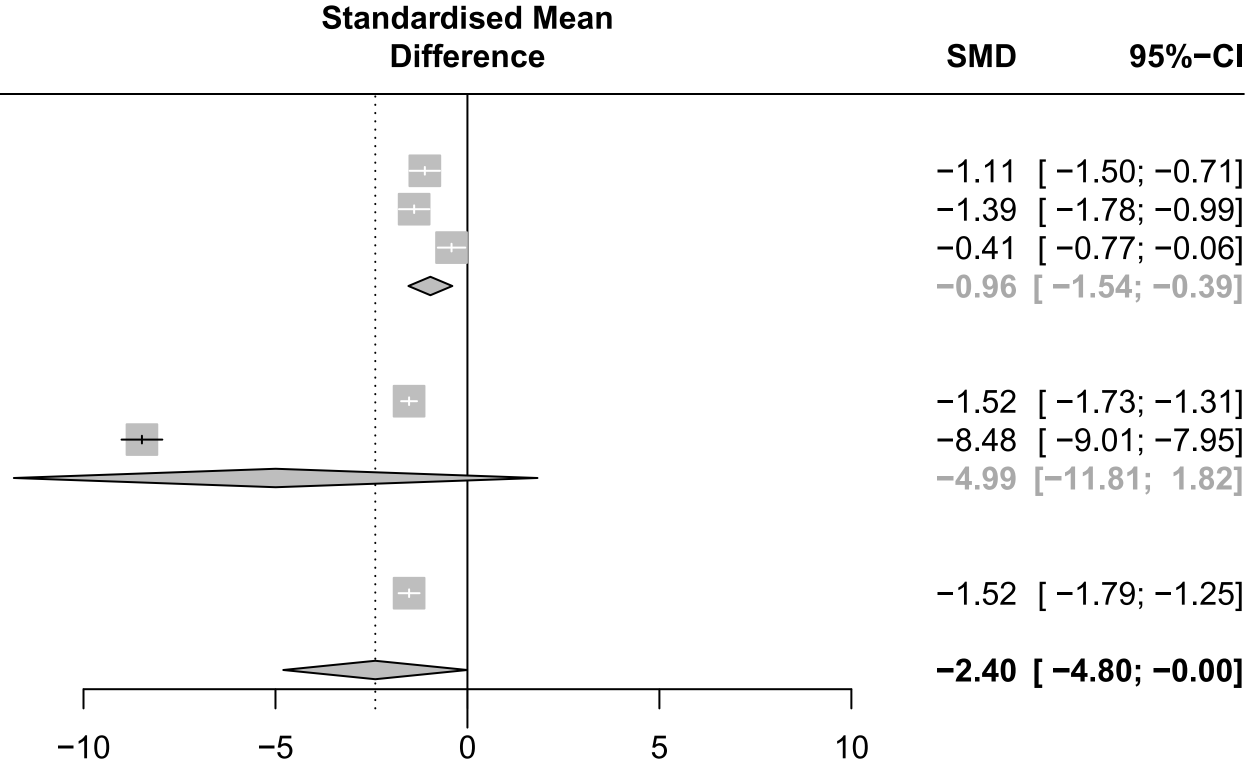

<− Faster progression for fast−progressing subtype | faster progression for slow−progressing subtype −>

Three−level metaanalysis using random effects to calculate an overall SMD estimate for Apathy across cohorts.
The dashed line indicates the overall mean SMD estimate. The solid line indicates no effect.

Forest plot for progression characteristics of symptom domain Sleep

Study	Slow progressing Total	Slow progressing Mean	Slow progressing SD	Fast progressing Total	Fast progressing Mean	Fast progressing SD	SMD	95%−CI
**Cohort = PPMI**								
ESS	324	0.15	0.1390	73	0.28	0.1024	−1.00	[−1.26; −0.73]
UPDRS I Sleep	335	0.25	0.1857	74	0.48	0.2186	−1.18	[−1.44; −0.92]
**Random effects model**	**659**			**147**			**−1.09**	**[−1.28; −0.90]**
**Cohort = ICEBERG**								
ESS	111	0.15	0.1219	41	0.22	0.0565	−0.67	[−1.04; −0.31]
NMSQ Sleep	112	1.08	0.5661	42	2.25	0.1739	−2.37	[−2.81; −1.92]
UPDRS I Sleep	112	0.07	0.0314	42	0.13	0.0082	−2.22	[−2.66; −1.79]
**Random effects model**	**335**			**125**			**−1.75**	**[−2.82; −0.68]**
**Cohort = LuxPARK**								
NMSQ Sleep	408	0.64	0.0903	152	0.83	0.5505	−0.61	[−0.80; −0.42]
PDSS	406	0.10	0.0189	152	0.13	0.0379	−1.29	[−1.49; −1.09]
UPDRS I Sleep	408	0.09	0.0213	153	0.13	0.0592	−1.29	[−1.49; −1.09]
**Random effects model**	**1222**			**457**			**−1.06**	**[−1.51; −0.62]**
**Random effects model**	**2216**			**729**			**−1.31**	**[−1.75; −0.88]**

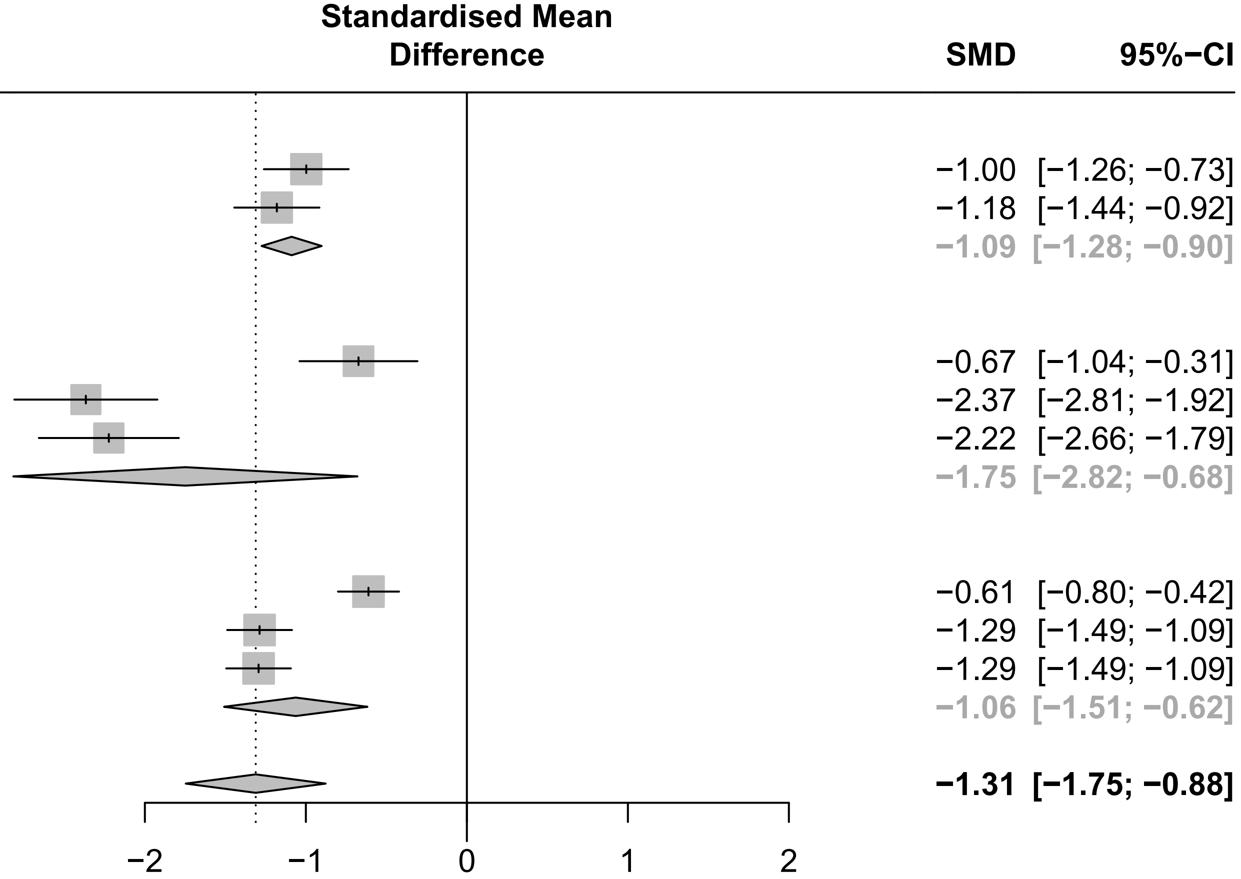

<- Faster progression for fast−progressing subtype | faster progression for slow−progressing subtype ->

Three−level metaanalysis using random effects to calculate an overall SMD estimate for Sleep across cohorts.
The dashed line indicates the overall mean SMD estimate. The solid line indicates no effect.

Forest plot for progression characteristics of symptom domain Overall cognition

Study	Slow progressing Total	Slow progressing Mean	Slow progressing SD	Fast progressing Total	Fast progressing Mean	Fast progressing SD	SMD	95%−CI
**Cohort = LuxPARK**								
FAB	42	0.02	0.0108	15	0.13	0.1039	−1.98	[−2.68; −1.28]
MoCA	408	0.03	0.0236	153	0.18	0.1102	−2.41	[−2.65; −2.18]
NMSQ Cognition	408	1.31	0.6814	151	2.55	1.0627	−1.54	[−1.75; −1.33]
PDQ39 Cognition	408	0.08	0.0242	152	0.16	0.0971	−1.47	[−1.68; −1.27]
SIQCDE	351	0.03	0.0424	122	0.08	0.0683	−0.99	[−1.21; −0.78]
UPDRS I Cognition	408	1.10	1.1392	153	2.17	1.5897	−0.84	[−1.03; −0.65]
**Random effects model**	**2025**			**746**			**−1.52**	**[−2.00; −1.04]**
**Cohort = ICEBERG**								
FAB	104	0.05	0.0378	41	0.02	0.0744	0.57	[0.20; 0.94]
MATTIS	104	0.05	0.0452	41	0.02	0.0664	0.58	[0.22; 0.95]
MoCA	112	0.05	0.0549	42	0.10	0.0770	−0.80	[−1.17; −0.43]
NMSQ Cognition	112	1.69	0.2667	42	1.90	0.3774	−0.70	[−1.07; −0.34]
UPDRS I Cognition	112	0.86	0.3592	42	1.21	1.2353	−0.49	[−0.85; −0.13]
**Random effects model**	**544**			**208**			**−0.17**	**[−0.77; 0.43]**
**Cohort = PPMI**								
MoCA	320	0.02	0.0720	71	0.29	0.2697	−2.08	[−2.37; −1.78]
UPDRS I Cognition	335	1.64	3.2067	74	3.73	5.1644	−0.57	[−0.83; −0.32]
**Random effects model**	**655**			**145**			**−1.32**	**[−2.80; 0.15]**
**Random effects model**	**3224**			**1099**			**−0.98**	**[−1.48; −0.48]**

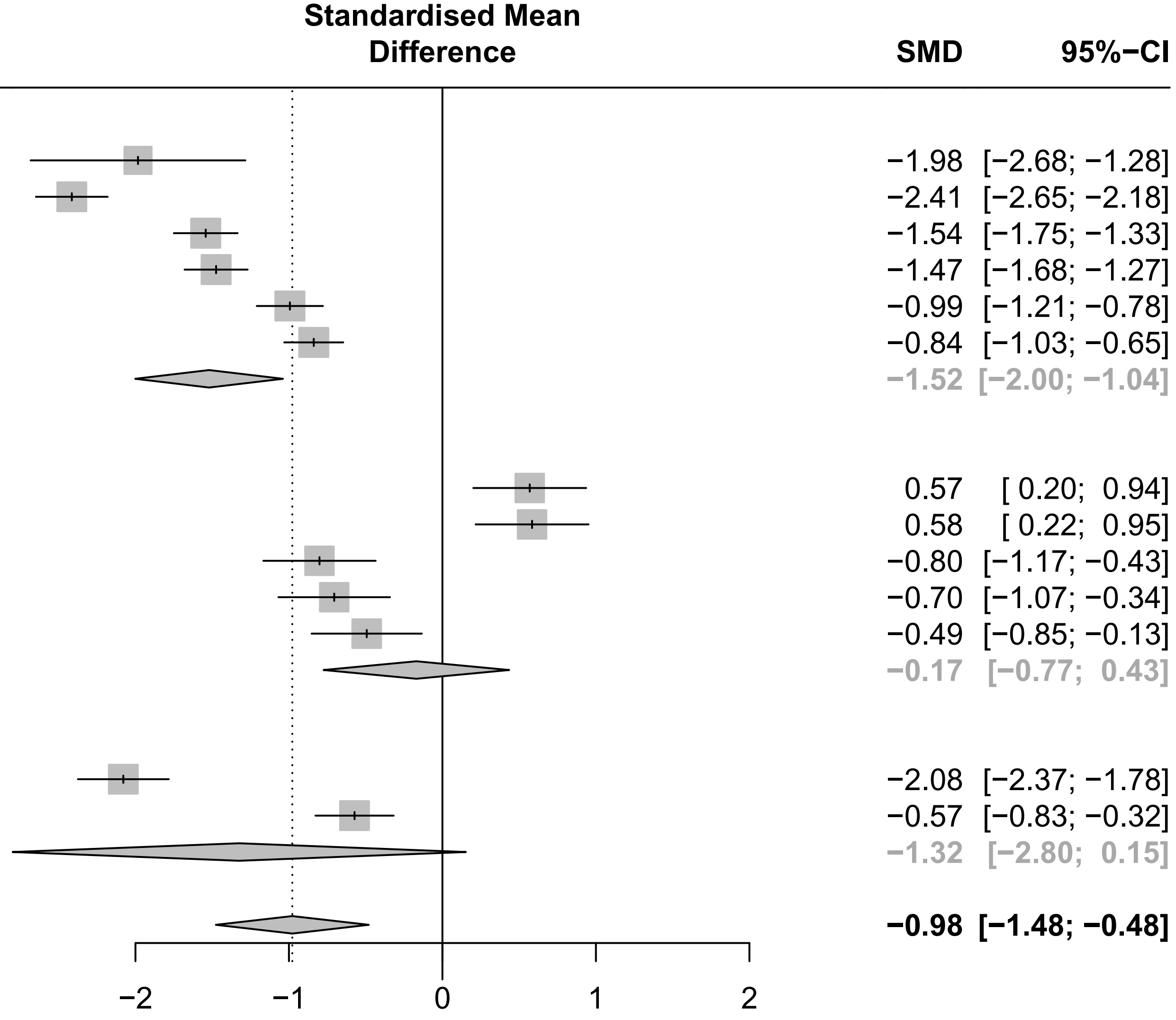

<− Faster progression for fast−progressing subtype | faster progression for slow−progressing subtype −>

Three−level metaanalysis using random effects to calculate an overall SMD estimate for Overall cognition across cohorts. The dashed line indicates the overall mean SMD estimate. The solid line indicates no effect.

Forest plot for progression characteristics of symptom domain Conceptualization

Study	Slow progressing Total	Slow progressing Mean	Slow progressing SD	Fast progressing Total	Fast progressing Mean	Fast progressing SD	SMD	95%−CI
**Cohort = LuxPARK**								
FAB Conceptualization	43	−1.31	0.2084	16	−2.15	1.9839	0.80	[0.21; 1.40]
MOCA Abstraction	408	0.16	0.2392	153	0.43	0.1078	−1.29	[−1.49; −1.09]
**Random effects model**	**451**			**169**			**−0.26**	**[−2.31; 1.79]**
**Cohort = ICEBERG**								
FAB Conceptualization	104	0.97	1.5829	41	0.87	0.2338	0.08	[−0.28; 0.44]
MATTIS Conzeptualization	104	0.00	0.0741	41	0.05	0.0337	−0.74	[−1.11; −0.37]
MOCA Abstraction	112	0.53	0.6543	42	1.72	0.5240	−1.90	[−2.31; −1.48]
**Random effects model**	**320**			**124**			**−0.85**	**[−1.97; 0.27]**
**Cohort = PPMI**								
MOCA Abstraction	320	−1.90	0.9625	71	1.57	1.5654	−3.16	[−3.50; −2.82]
**Random effects model**	**1091**			**364**			**−1.04**	**[−2.17; 0.08]**

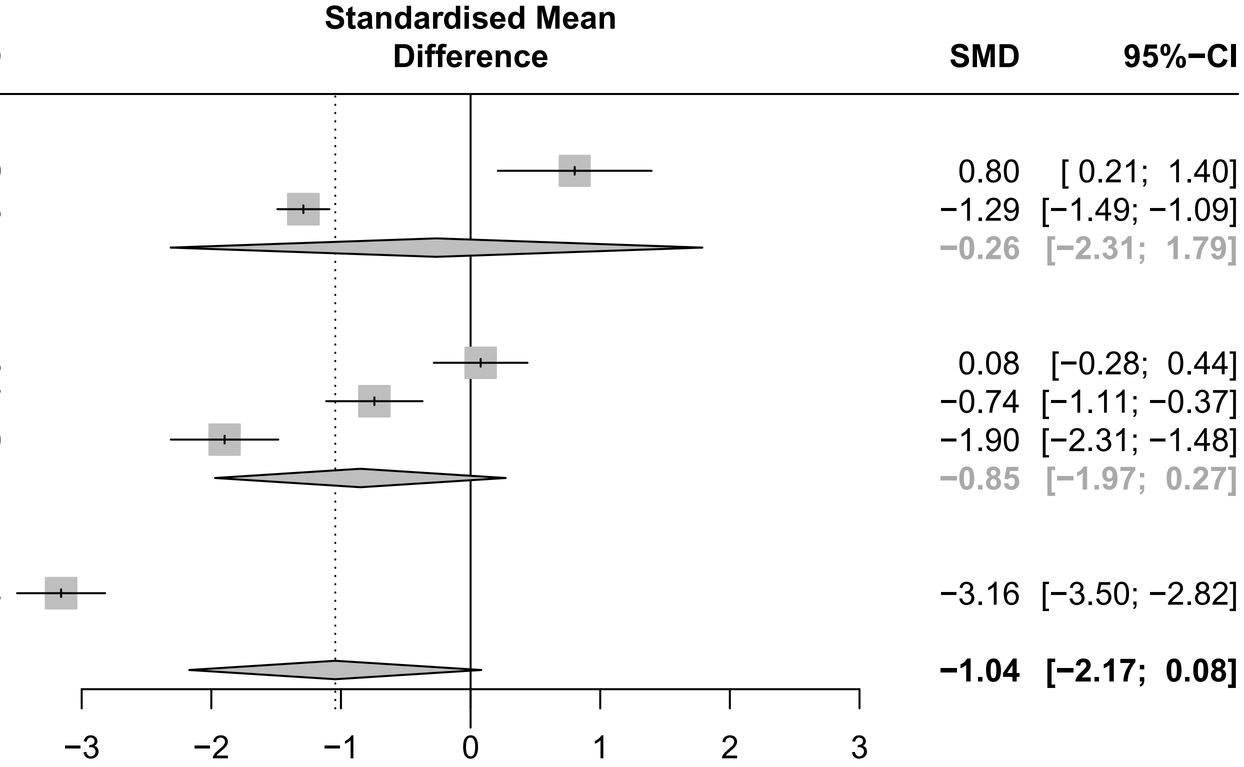

<− Faster progression for fast−progressing subtype | faster progression for slow−progressing subtype −>

Three−level metaanalysis using random effects to calculate an overall SMD estimate for Conceptualization across cohorts. The dashed line indicates the overall mean SMD estimate. The solid line indicates no effect.

Forest plot for progression characteristics of symptom domain Visuo−executive

Study	Slow progressing Total	Slow progressing Mean	Slow progressing SD	Fast progressing Total	Fast progressing Mean	Fast progressing SD	SMD	95%−CI
**Cohort = ICEBERG**								
FAB 3−6	104	0.06	0.0473	41	0.02	0.1003	0.64	[0.27; 1.01]
MATTIS Initiation + Construction	104	0.04	0.0456	41	0.03	0.0611	0.33	[−0.03; 0.69]
MOCA Visuspatial/Executive	112	0.81	0.5368	42	1.37	0.1911	−1.20	[−1.58; −0.82]
**Random effects model**	**320**			**124**			**−0.08**	**[−1.19; 1.04]**
**Cohort = LuxPARK**								
FAB 3−6	42	0.07	0.0238	15	0.09	0.0408	−0.74	[−1.34; −0.13]
MOCA Visuspatial/Executive	408	0.86	0.0973	153	2.12	0.3547	−6.23	[−6.64; −5.82]
Trailmaking A	392	0.02	0.0225	134	0.07	0.0747	−1.17	[−1.38; −0.96]
Trailmaking B	357	0.04	0.0371	99	0.09	0.0523	−1.20	[−1.44; −0.97]
**Random effects model**	**1199**			**401**			**−2.34**	**[−4.89; 0.22]**
**Cohort = PPMI**								
Judgement Line Orientation	320	0.04	0.0357	71	0.17	0.1483	−1.84	[−2.13; −1.55]
MOCA Visuspatial/Executive	320	0.77	1.2703	71	4.01	2.9805	−1.89	[−2.18; −1.60]
Symbol Digit Modalities	320	0.05	0.0727	71	0.21	0.1285	−1.81	[−2.10; −1.52]
**Random effects model**	**960**			**213**			**−1.85**	**[−2.02; −1.68]**
**Random effects model**	**2479**			**738**			**−1.51**	**[−2.67; −0.35]**

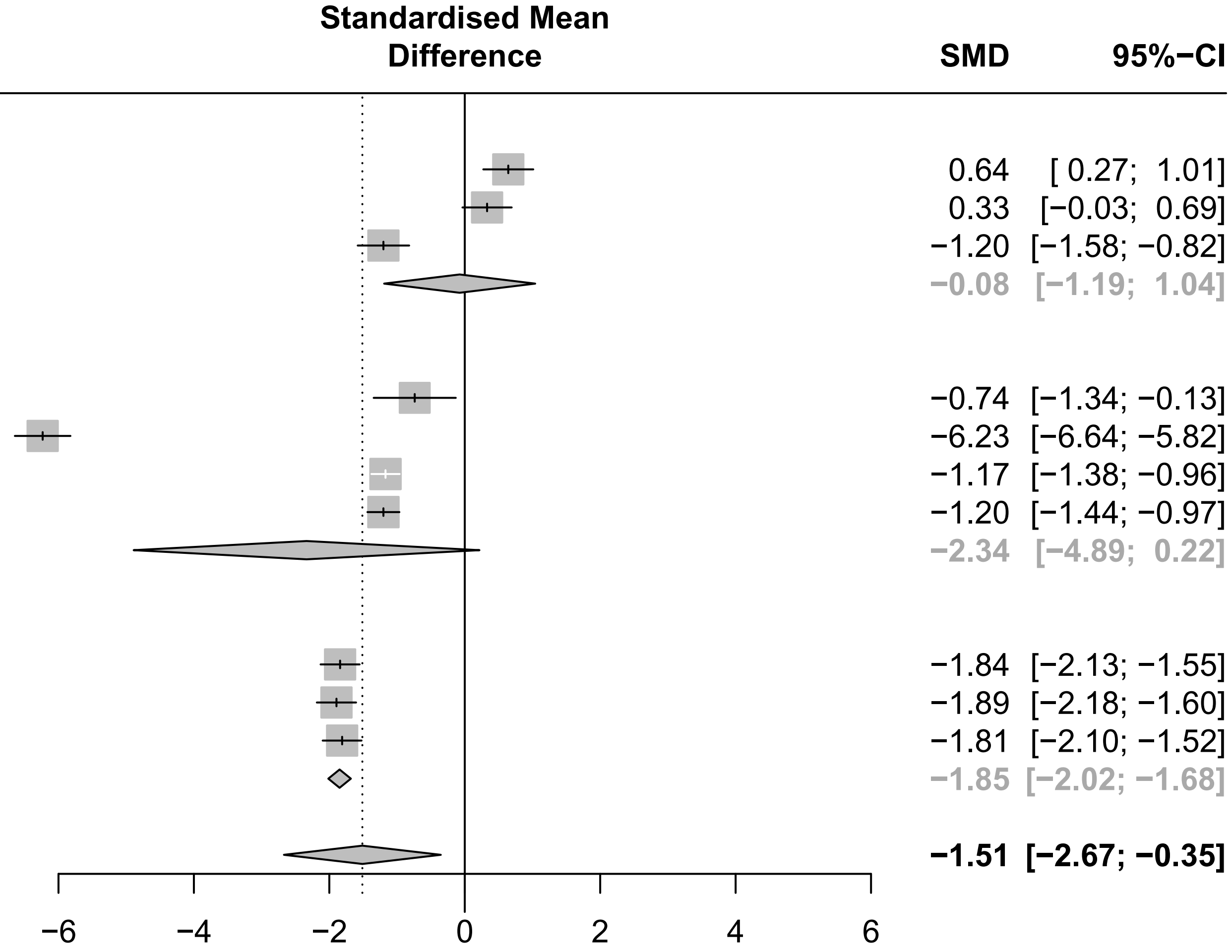

<− Faster progression for fast−progressing subtype | faster progression for slow−progressing subtype −>

Three−level metaanalysis using random effects to calculate an overall SMD estimate for Visuo−executive across cohorts. The dashed line indicates the overall mean SMD estimate. The solid line indicates no effect.

Forest plot for progression characteristics of symptom domain Language

Study	Slow progressing Total	Slow progressing Mean	Slow progressing SD	Fast progressing Total	Fast progressing Mean	Fast progressing SD	SMD	95%−CI
**Cohort = LuxPARK**								
FAB VFT	43	−0.41	1.4483	16	2.00	0.2269	−1.90	[−2.58; −1.23]
MOCA Language + Naming	408	0.03	0.0199	153	0.12	0.0251	−4.32	[−4.63; −4.00]
VFT phonematic F	332	−0.01	0.0148	129	0.03	0.0095	−2.89	[−3.16; −2.61]
VFT phonematic S	50	−0.08	0.0356	21	0.11	0.0389	−5.18	[−6.19; −4.17]
**Random effects model**	**833**			**319**			**−3.55**	**[−4.94; −2.15]**
**Cohort = ICEBERG**								
FAB VFT	104	−0.35	0.5878	41	−1.33	1.6903	0.95	[0.57; 1.33]
MOCA Language + Naming	112	0.03	0.0362	42	0.05	0.0513	−0.60	[−0.96; −0.24]
VFT phonematic F	112	0.03	0.0353	42	0.03	0.0767	−0.12	[−0.47; 0.24]
VFT phonematic S	100	−0.00	0.0072	41	0.03	0.0256	−2.05	[−2.49; −1.61]
VFT semantic supermarket	99	0.06	0.0162	41	0.01	0.0160	3.22	[2.69; 3.74]
**Random effects model**	**527**			**207**			**0.28**	**[−1.44; 1.99]**
**Cohort = PPMI**								
MOCA Language + Naming	320	−0.04	0.0632	71	0.06	0.0917	−1.44	[−1.72; −1.16]
VFT phonematic F	320	−0.05	0.0624	71	0.04	0.0871	−1.34	[−1.61; −1.06]
VFT semantic sum	320	0.02	0.0793	71	0.12	0.1057	−1.15	[−1.42; −0.88]
**Random effects model**	**960**			**213**			**−1.31**	**[−1.48; −1.14]**
**Random effects model**	**2320**			**739**			**−1.39**	**[−2.64; −0.14]**

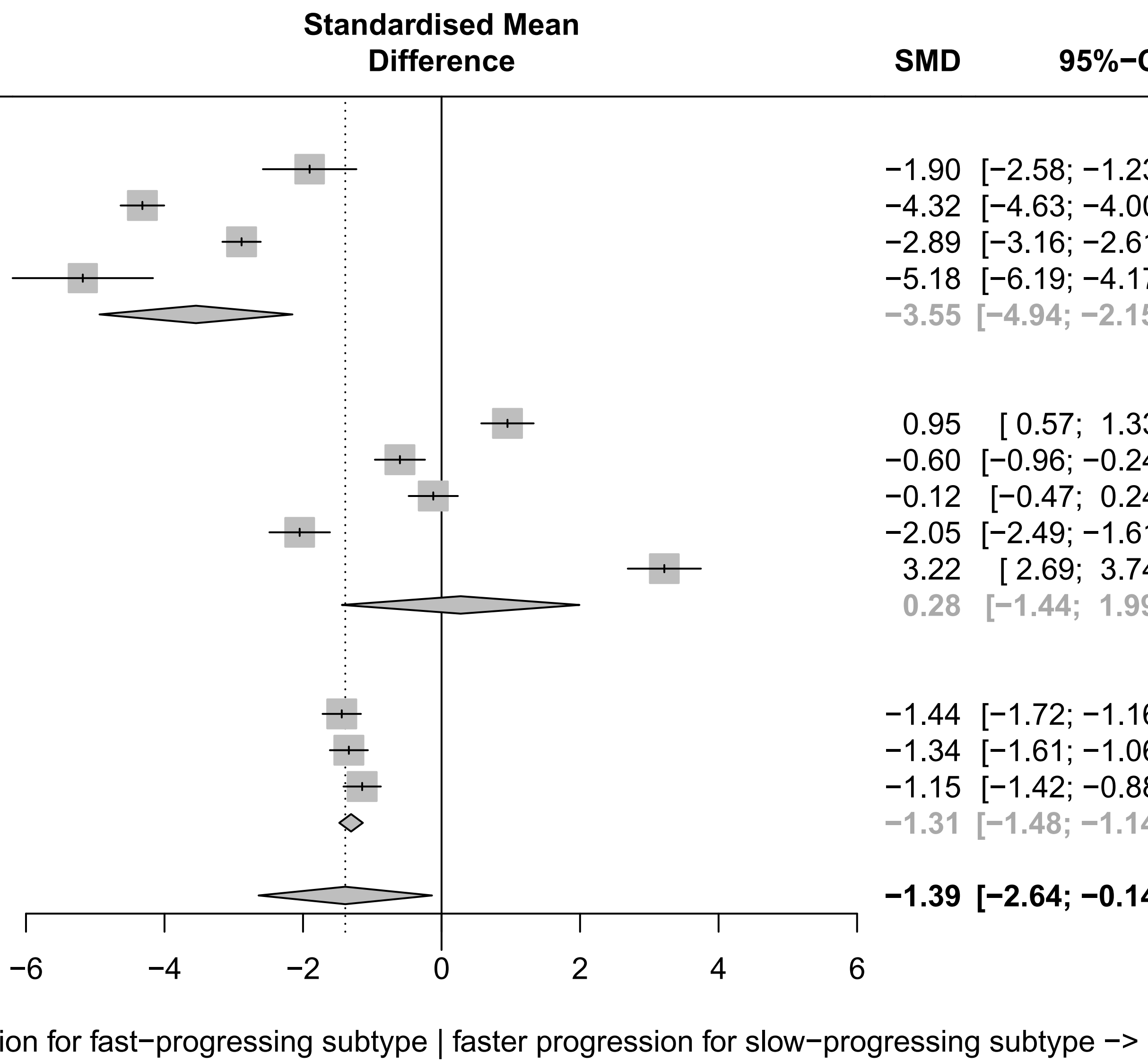

Three−level metaanalysis using random effects to calculate an overall SMD estimate for Language across cohorts.
The dashed line indicates the overall mean SMD estimate. The solid line indicates no effect.

Forest plot for progression characteristics of symptom domain Anxiety

Study	Slow progressing Total	Slow progressing Mean	Slow progressing SD	Fast progressing Total	Fast progressing Mean	Fast progressing SD	SMD	95%−CI
**Cohort = ICEBERG**								
HADS anxiety	112	0.02	0.0221	42	0.08	0.0598	−1.76	[−2.16; −1.35]
NMSQ Anxiety	111	1.38	0.8609	42	1.29	1.9033	0.07	[−0.29; 0.42]
UPDRS I Anxiety	112	0.90	0.0762	42	0.84	1.9574	0.06	[−0.29; 0.42]
**Random effects model**	**335**			**126**			**−0.54**	**[−1.73; 0.65]**
**Cohort = LuxPARK**								
NMSQ Anxiety	408	1.08	0.3337	151	1.74	0.3269	−2.00	[−2.22; −1.77]
PDQ39 Anxiety	408	1.03	0.3142	153	1.67	0.5120	−1.70	[−1.91; −1.49]
UPDRS I Anxiety	408	0.64	0.1651	153	1.25	1.1298	−1.01	[−1.20; −0.81]
**Random effects model**	**1224**			**457**			**−1.57**	**[−2.14; −0.99]**
**Cohort = PPMI**								
STA	323	0.02	0.1127	72	0.23	0.0952	−1.94	[−2.23; −1.65]
UPDRS I Anxiety	335	1.05	2.2489	74	3.37	2.2233	−1.04	[−1.30; −0.77]
**Random effects model**	**658**			**146**			**−1.48**	**[−2.37; −0.60]**
**Random effects model**	**2217**			**729**			**−1.17**	**[−1.75; −0.59]**

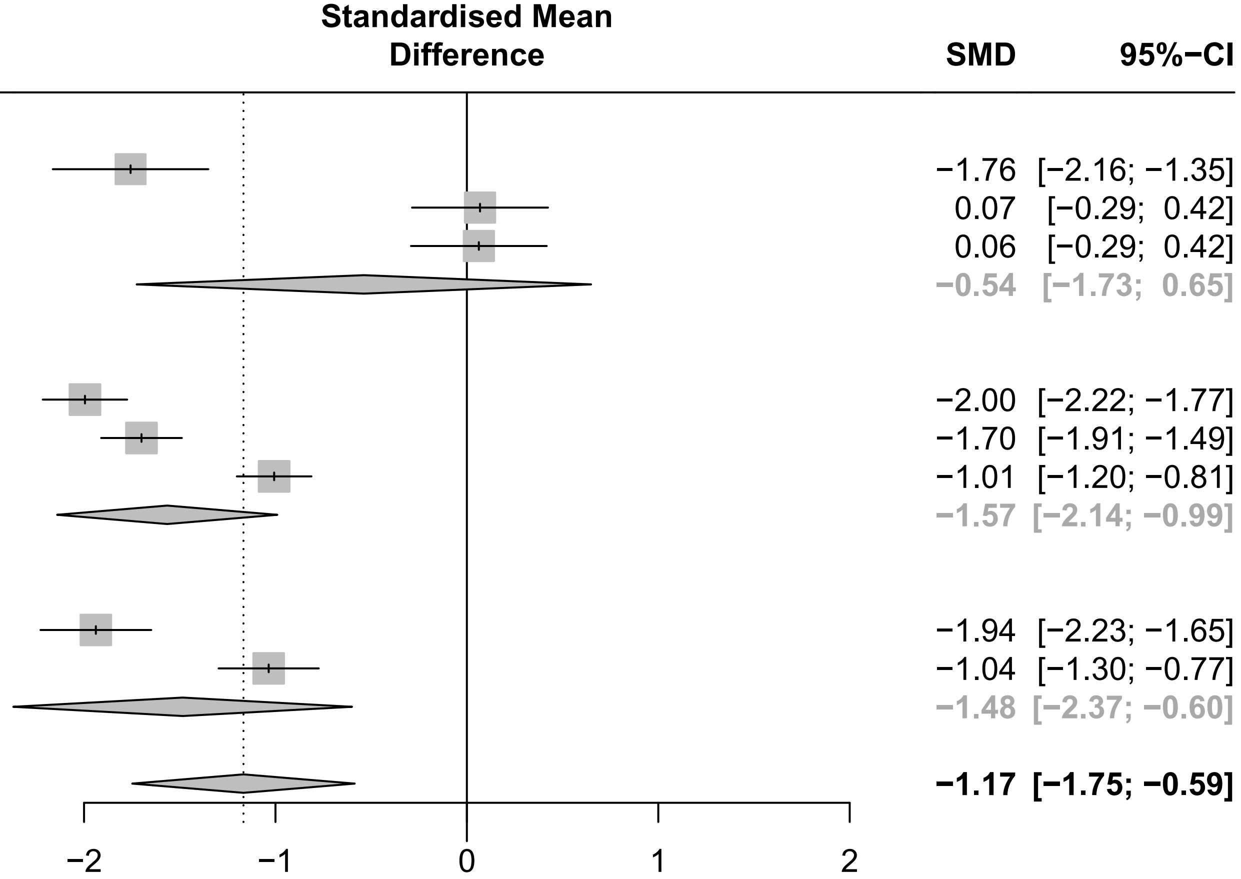

<- Faster progression for fast−progressing subtype | faster progression for slow−progressing subtype ->

Three−level metaanalysis using random effects to calculate an overall SMD estimate for Anxiety across cohorts.
The dashed line indicates the overall mean SMD estimate. The solid line indicates no effect.

Forest plot for progression characteristics of symptom domain Memory

Study	Slow progressing Total	Slow progressing Mean	Slow progressing SD	Fast progressing Total	Fast progressing Mean	Fast progressing SD	SMD	95%−CI
**Cohort = PPMI**								
Hopkins Verbal Learning Test DR	320	0.01	0.0245	71	−0.00	0.0144	0.59	[0.33; 0.85]
Hopkins Verbal Learning Test IR	320	0.04	0.0872	71	0.17	0.1296	−1.32	[−1.60; −1.05]
MOCA Orientation + Memory	320	−0.01	0.0912	71	0.21	0.1801	−1.97	[−2.26; −1.68]
**Random effects model**	**960**			**213**			**−0.90**	**[−2.41; 0.61]**
**Cohort = ICEBERG**								
MATTIS Memory	104	0.07	0.0605	41	0.08	0.0382	−0.21	[−0.57; 0.16]
MOCA Orientation + Memory	112	0.02	0.0179	42	0.03	0.0961	−0.37	[−0.72; −0.01]
NMSQ Memory	111	1.19	1.4812	42	2.28	1.4312	−0.74	[−1.10; −0.37]
**Random effects model**	**327**			**125**			**−0.44**	**[−0.74; −0.13]**
**Cohort = LuxPARK**								
MOCA Orientation + Memory	408	0.01	0.0204	153	0.11	0.0920	−2.05	[−2.27; −1.83]
NMSQ Memory	408	1.13	0.0606	151	2.30	1.2830	−1.75	[−1.96; −1.53]
PDQ39 Memory	408	0.74	0.2966	152	1.58	1.5416	−1.01	[−1.20; −0.81]
SIQCDE Memory	352	0.03	0.0459	122	0.07	0.0527	−0.68	[−0.89; −0.47]
**Random effects model**	**1576**			**578**			**−1.37**	**[−1.99; −0.75]**
**Random effects model**	**2863**			**916**			**−0.95**	**[−1.48; −0.43]**

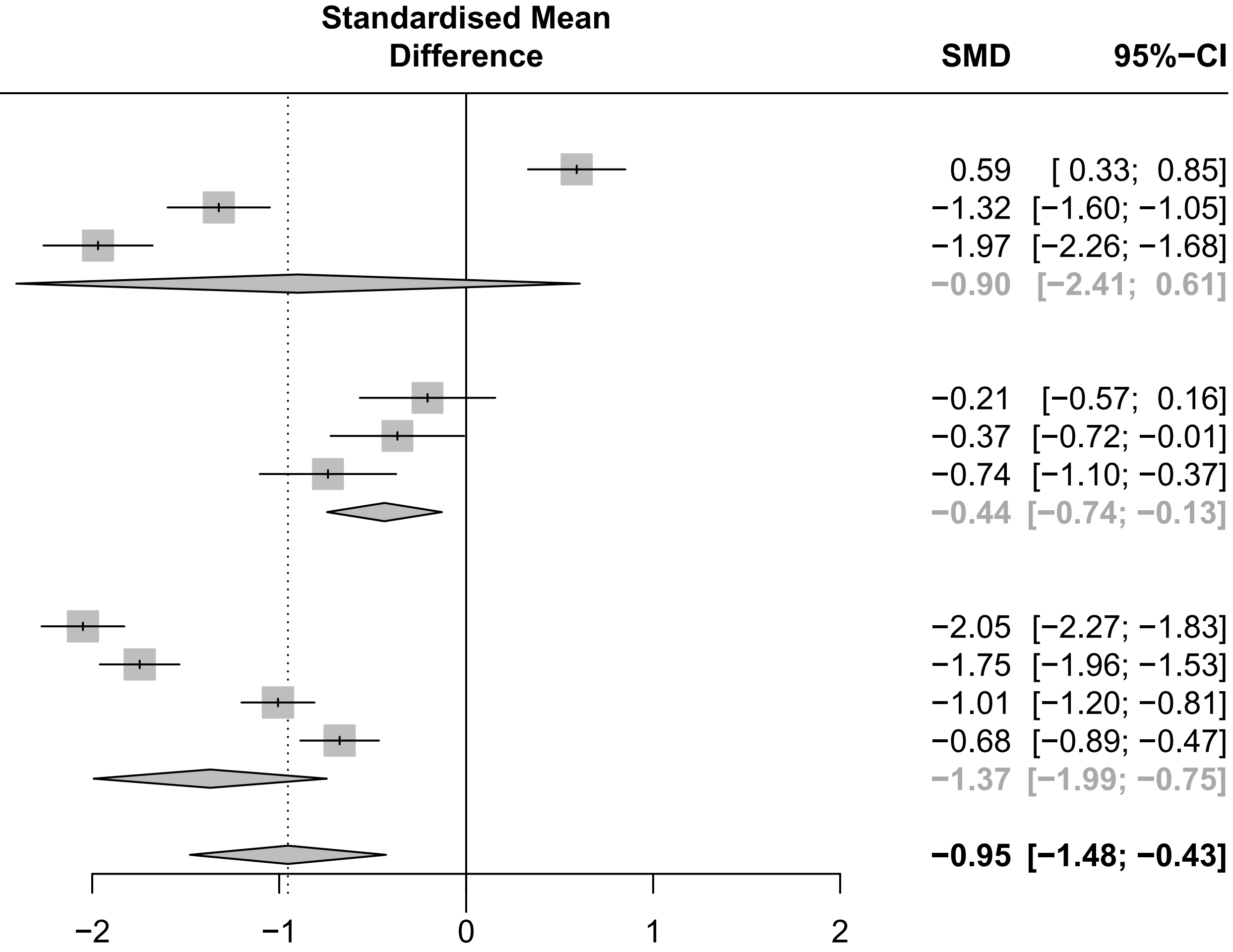

<− Faster progression for fast−progressing subtype | faster progression for slow−progressing subtype −>

Three−level metaanalysis using random effects to calculate an overall SMD estimate for Memory across cohorts.
The dashed line indicates the overall mean SMD estimate. The solid line indicates no effect.

Forest plot for progression characteristics of symptom domain Attention

Study	Slow progressing Total	Slow progressing Mean	Slow progressing SD	Fast progressing Total	Fast progressing Mean	Fast progressing SD	SMD	95%−CI
**Cohort = PPMI**								
Letter Number Sequencing	56	0.03	0.1650	5	−0.04	0.0202	0.40	[−0.52; 1.31]
MOCA Attention	320	1.33	0.7561	71	3.97	2.4125	−2.15	[−2.44; −1.85]
**Random effects model**	**376**			**76**			**−0.91**	**[−3.40; 1.58]**
**Cohort = ICEBERG**								
MATTIS Attention	104	0.00	0.0367	41	−0.02	0.0090	0.71	[0.34; 1.08]
MOCA Attention	112	0.52	0.2299	42	0.94	0.5589	−1.18	[−1.56; −0.80]
NMSQ Attention	111	2.20	0.0771	42	1.79	0.1678	3.71	[3.16; 4.26]
**Random effects model**	**327**			**125**			**1.07**	**[−1.71; 3.86]**
**Cohort = LuxPARK**								
MOCA Attention	408	0.58	0.3055	153	1.52	0.5164	−2.49	[−2.73; −2.25]
NMSQ Attention	408	1.47	1.1380	152	2.09	0.1286	−0.63	[−0.82; −0.44]
PDQ39 Attention	408	1.34	0.2652	153	1.77	0.4815	−1.29	[−1.49; −1.09]
SIQCDE Attention	353	0.49	0.3458	122	1.00	0.8884	−0.94	[−1.15; −0.72]
**Random effects model**	**1577**			**580**			**−1.34**	**[−2.13; −0.54]**
**Random effects model**	**2280**			**781**			**−0.44**	**[−1.66; 0.77]**

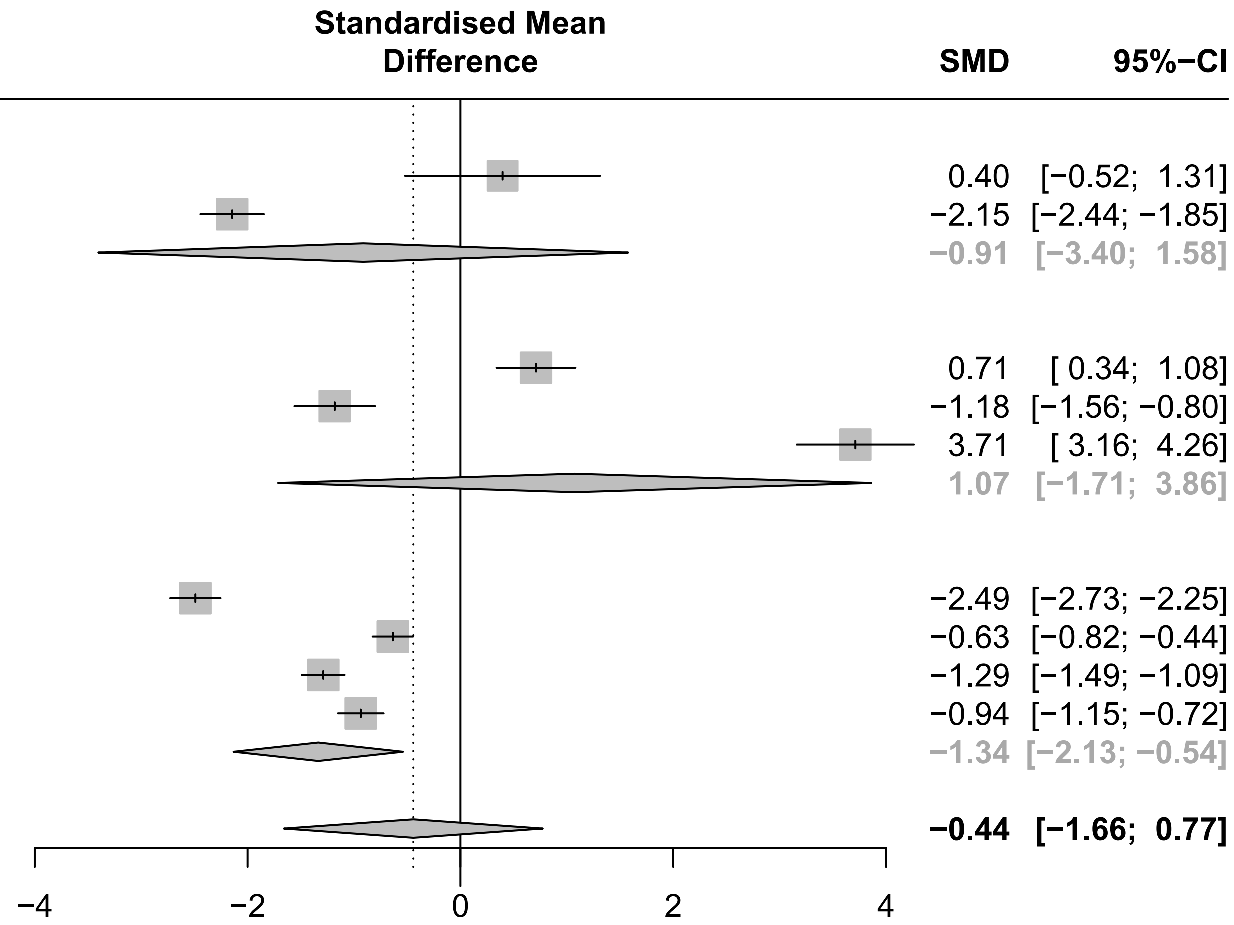

<− Faster progression for fast−progressing subtype | faster progression for slow−progressing subtype −>

Three−level metaanalysis using random effects to calculate an overall SMD estimate for Attention across cohorts. The dashed line indicates the overall mean SMD estimate. The solid line indicates no effect.

Forest plot for progression characteristics of symptom domain Non motor symptoms

Study	Slow progressing Total	Slow progressing Mean	Slow progressing SD	Fast progressing Total	Fast progressing Mean	Fast progressing SD	SMD	95%−CI
**Cohort = LuxPARK**								
NMSQ	402	0.11	0.0262	148	0.20	0.0522	−2.75	[−3.00; −2.50]
UPDRS I	408	0.10	0.0268	153	0.22	0.0835	−2.41	[−2.64; −2.17]
**Random effects model**	**810**			**301**			**−2.58**	**[−2.91; −2.24]**
**Cohort = ICEBERG**								
NMSQ	111	0.20	0.0447	42	0.22	0.0016	−0.50	[−0.86; −0.14]
UPDRS I	112	0.14	0.0182	42	0.19	0.0535	−1.66	[−2.06; −1.25]
**Random effects model**	**223**			**84**			**−1.07**	**[−2.21; 0.06]**
**Cohort = PPMI**								
UPDRS I	335	0.19	0.1170	74	0.55	0.1480	−2.97	[−3.30; −2.65]
**Random effects model**	**1368**			**459**			**−2.06**	**[−2.94; −1.19]**

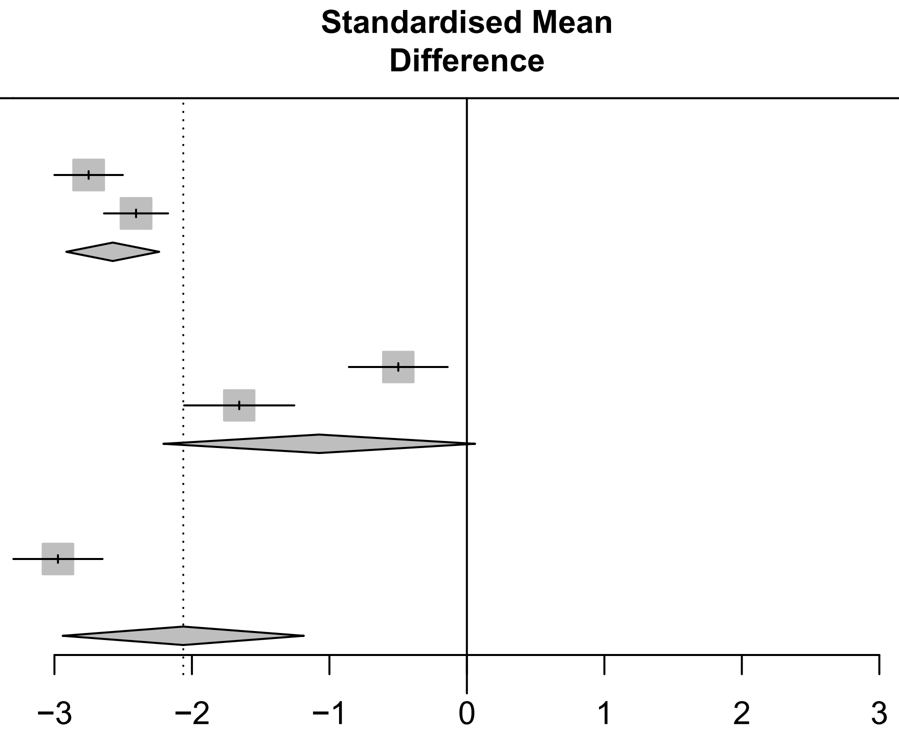

<− Faster progression for fast−progressing subtype | faster progression for slow−progressing subtype −>

Three−level metaanalysis using random effects to calculate an overall SMD estimate for Non motor symptoms across cohorts. The dashed line indicates the overall mean SMD estimate. The solid line indicates no effect.

Forest plot for progression characteristics of symptom domain Autonomic

Study	Slow progressing Total	Slow progressing Mean	Slow progressing SD	Fast progressing Total	Fast progressing Mean	Fast progressing SD	SMD	95%−CI
**Cohort = ICEBERG**								
NMSQ Autonomic	112	0.20	0.0189	42	0.15	0.0325	2.19	[1.75; 2.62]
SCOPA−AUT	112	0.19	0.0539	42	0.22	0.0462	−0.54	[−0.90; −0.18]
UPDRS I Autonomic	112	0.21	0.0522	42	0.19	0.0746	0.40	[0.04; 0.75]
**Random effects model**	**336**			**126**			**0.68**	**[−0.89; 2.24]**
**Cohort = LuxPARK**								
NMSQ Autonomic	403	0.12	0.0237	150	0.17	0.0604	−1.36	[−1.56; −1.16]
SCOPA−AUT	408	0.12	0.0379	153	0.16	0.0429	−0.93	[−1.12; −0.74]
UPDRS I Autonomic	408	0.12	0.0361	153	0.20	0.0612	−1.92	[−2.14; −1.70]
**Random effects model**	**1219**			**456**			**−1.40**	**[−1.97; −0.84]**
**Cohort = PPMI**								
SCOPA−AUT	321	0.14	0.0837	73	0.35	0.0108	−2.71	[−3.02; −2.39]
UPDRS I Autonomic	335	0.17	0.1074	74	0.49	0.1282	−2.83	[−3.15; −2.51]
**Random effects model**	**656**			**147**			**−2.77**	**[−2.99; −2.54]**
**Random effects model**	**2211**			**729**			**−0.97**	**[−2.12; 0.18]**

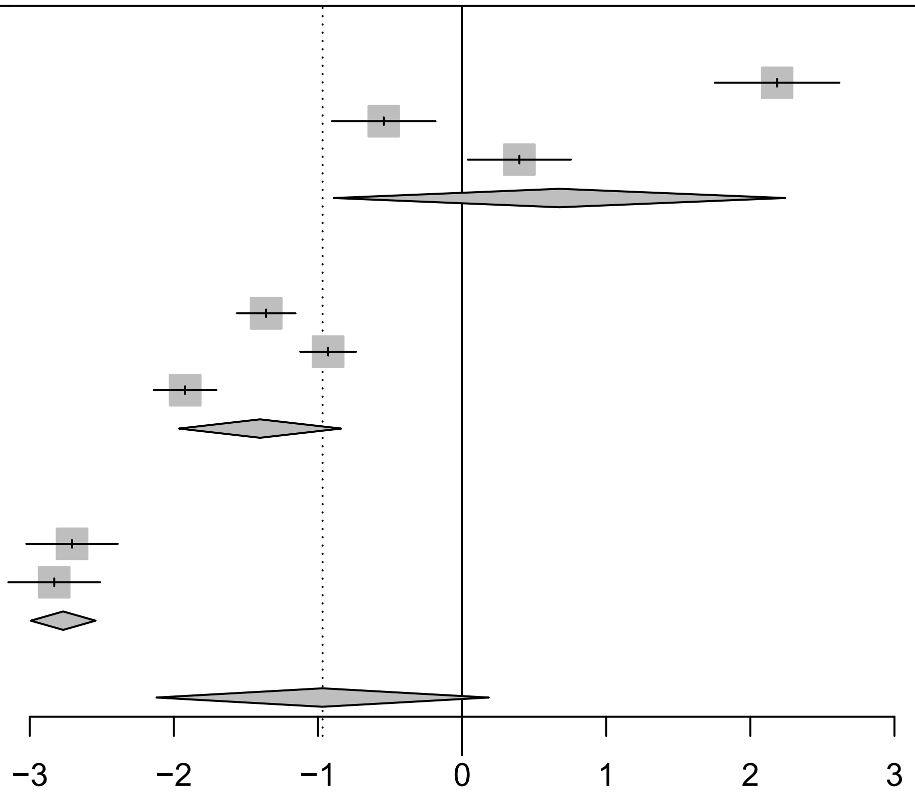

<- Faster progression for fast−progressing subtype | faster progression for slow−progressing subtype ->

Three−level metaanalysis using random effects to calculate an overall SMD estimate for Autonomic across cohorts.
The dashed line indicates the overall mean SMD estimate. The solid line indicates no effect.

Forest plot for progression characteristics of symptom domain Hallucinations

Study	Slow progressing Total	Slow progressing Mean	Slow progressing SD	Fast progressing Total	Fast progressing Mean	Fast progressing SD	SMD	95%−CI
**Cohort = ICEBERG**								
NMSQ Hallucination	112	−2.07	1.7091	42	2.19	1.0470	−2.72	[−3.19; −2.25]
UPDRS I Hallucinations	112	0.72	1.4359	42	4.74	6.1700	−1.17	[−1.55; −0.79]
**Random effects model**	**224**			**84**			**−1.94**	**[−3.46; −0.42]**
**Cohort = LuxPARK**								
NMSQ Hallucination	408	1.63	0.3010	152	1.79	0.6111	−0.39	[−0.58; −0.21]
UPDRS I Hallucinations	408	1.23	0.2917	153	1.33	0.8760	−0.19	[−0.38; −0.00]
**Random effects model**	**816**			**305**			**−0.29**	**[−0.49; −0.09]**
**Cohort = PPMI**								
UPDRS I Hallucinations	335	3.04	0.7427	74	6.04	4.4997	−1.48	[−1.76; −1.21]
**Random effects model**	**1375**			**463**			**−1.18**	**[−2.05; −0.30]**

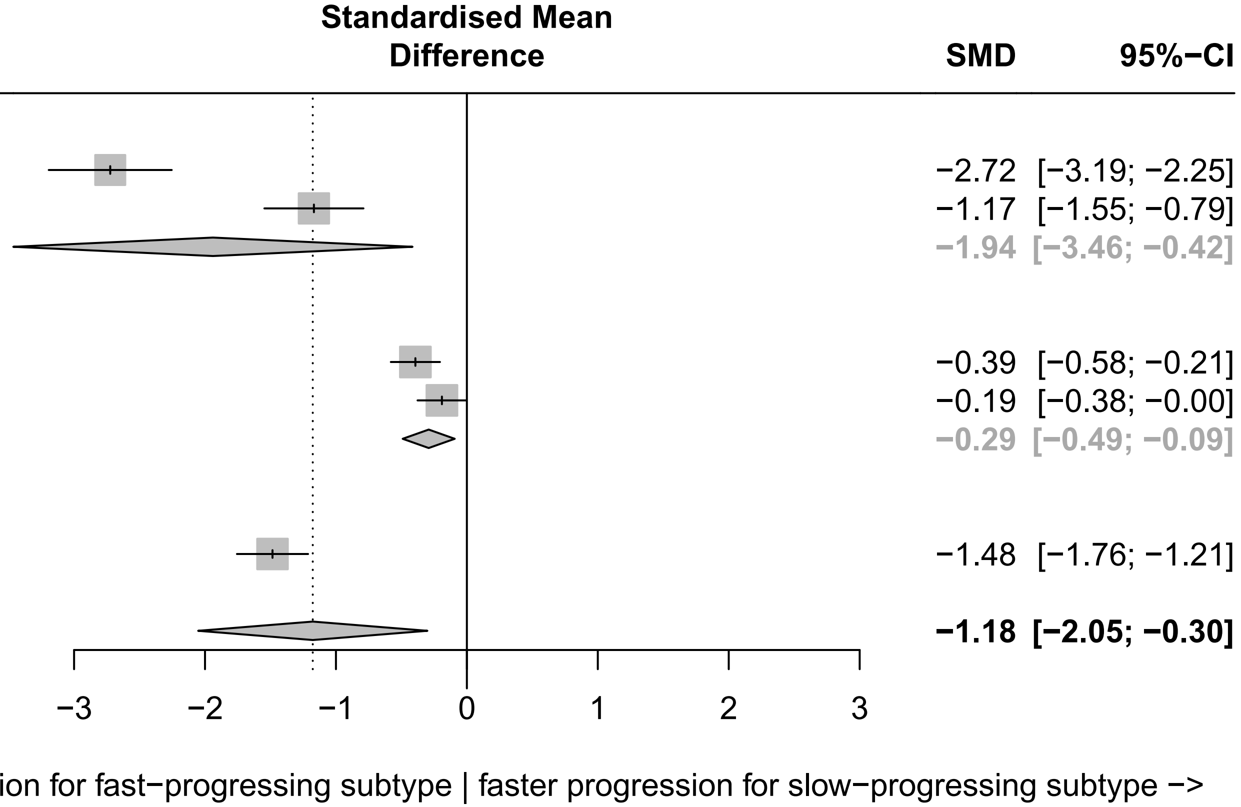

Three−level metaanalysis using random effects to calculate an overall SMD estimate for Hallucinations across cohorts. The dashed line indicates the overall mean SMD estimate. The solid line indicates no effect.

Forest plot for progression characteristics of symptom domain Pain

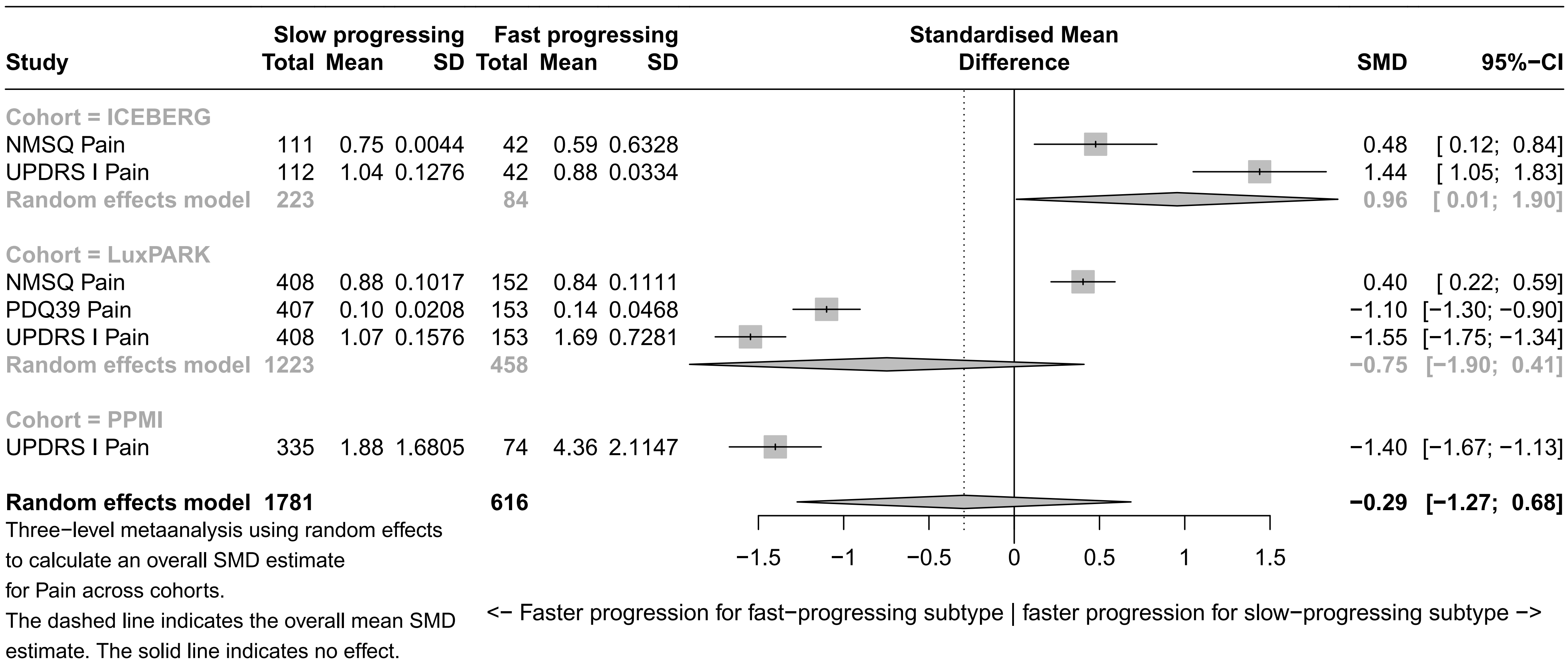

Forest plot for progression characteristics of symptom domain RBD

Study	Slow progressing Total	Slow progressing Mean	Slow progressing SD	Fast progressing Total	Fast progressing Mean	Fast progressing SD	SMD	95%−CI
**Cohort = ICEBERG**								
NMSQ RBD	112	1.73	0.3193	42	0.72	1.1740	1.50	[1.11; 1.89]
RBD−HK	111	0.07	0.0573	41	0.03	0.1471	0.37	[0.01; 0.73]
**Random effects model**	**223**			**83**			**0.93**	**[−0.18; 2.04]**
**Cohort = LuxPARK**								
NMSQ RBD	407	1.58	0.3502	152	1.11	0.3609	1.33	[1.12; 1.53]
RBD−SQ	405	0.10	0.0627	152	0.07	0.0916	0.40	[0.21; 0.59]
**Random effects model**	**812**			**304**			**0.86**	**[−0.04; 1.77]**
**Cohort = PPMI**								
RBD−SQ	324	0.18	0.2022	72	0.18	0.1810	0.03	[−0.23; 0.28]
**Random effects model**	**1359**			**459**			**0.72**	**[0.15; 1.28]**

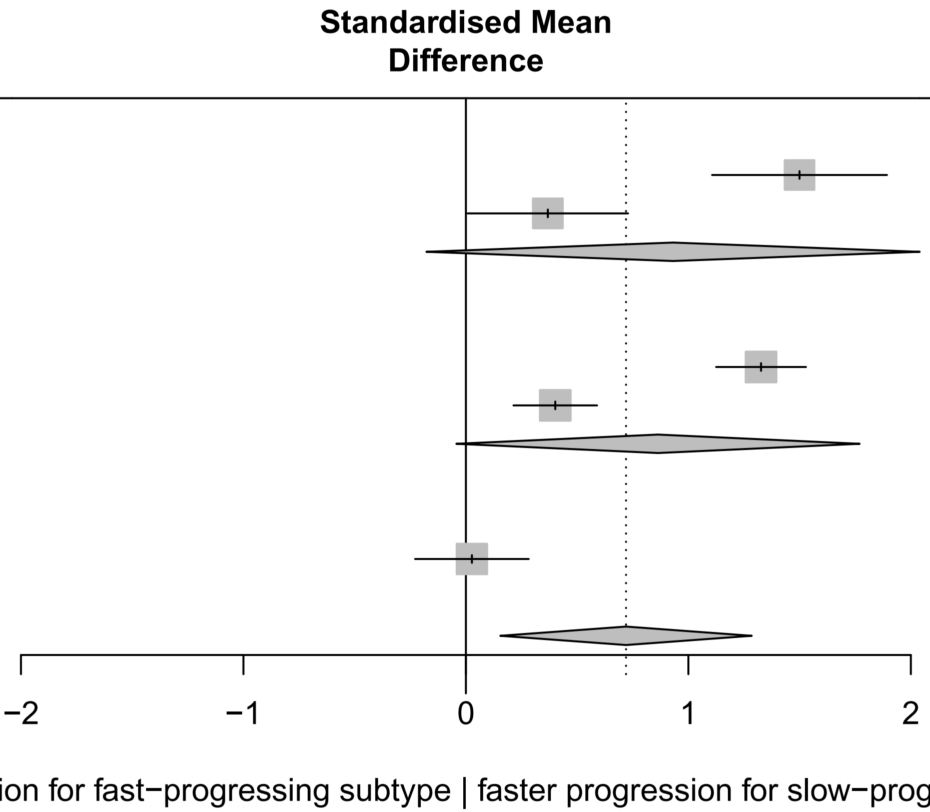

Three−level metaanalysis using random effects to calculate an overall SMD estimate for RBD across cohorts.
The dashed line indicates the overall mean SMD estimate. The solid line indicates no effect.

Forest plot for progression characteristics of symptom domain Smell

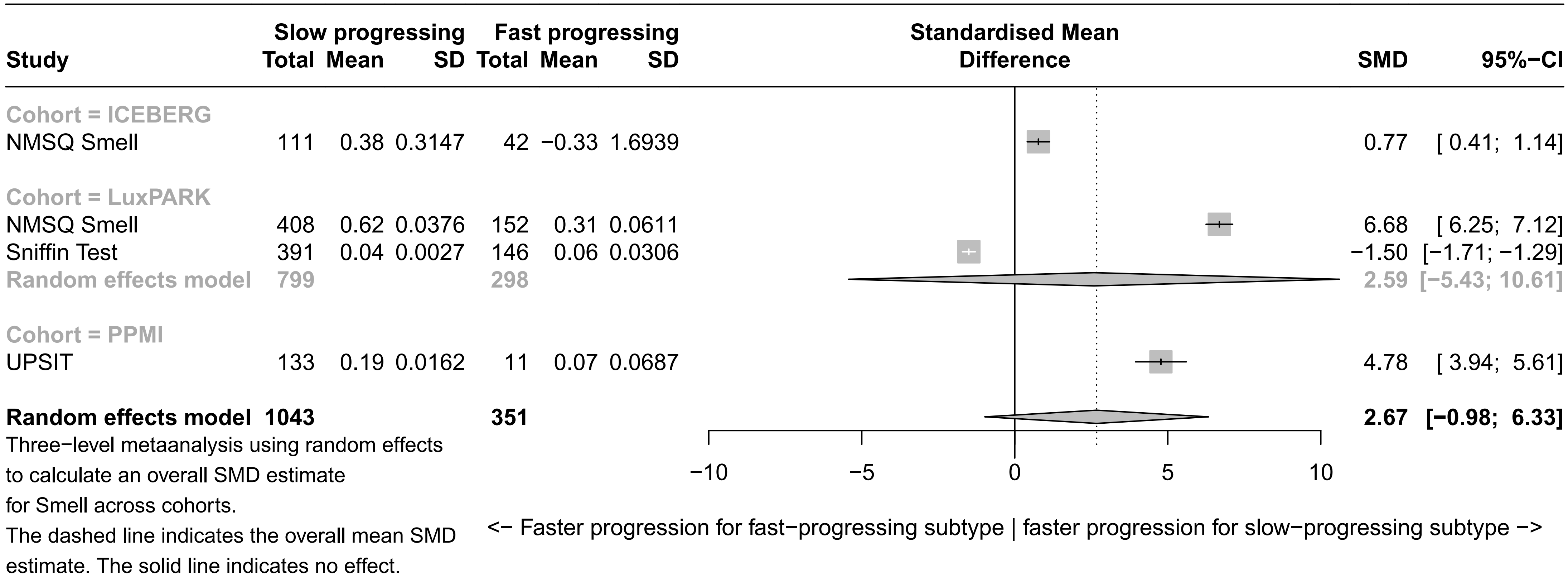

Forest plot for progression characteristics of symptom domain Motor symptoms

Study	Slow progressing Total	Slow progressing Mean	Slow progressing SD	Fast progressing Total	Fast progressing Mean	Fast progressing SD	SMD	95%−CI
**Cohort = LuxPARK**								
PDQ39 ADL	408	0.15	0.0652	152	0.36	0.1909	−1.85	[−2.06; −1.63]
Pegboard	386	0.06	0.0171	128	0.12	0.0374	−2.43	[−2.68; −2.18]
UPDRS II	408	0.15	0.0555	153	0.42	0.1606	−2.75	[−2.99; −2.50]
UPDRS III on	380	0.09	0.0212	139	0.23	0.0707	−3.45	[−3.74; −3.17]
UPDRS IV	408	0.09	0.0759	153	0.11	0.0872	−0.18	[−0.36; 0.01]
**Random effects model**	**1990**			**725**			**−2.13**	**[−3.21; −1.04]**
**Cohort = PPMI**								
UPDRS II	335	0.17	0.1047	74	0.59	0.1604	−3.57	[−3.92; −3.21]
UPDRS III off	335	0.26	0.1491	74	0.58	0.1757	−2.02	[−2.30; −1.73]
UPDRS III on	300	0.03	0.1291	66	0.31	0.2480	−1.77	[−2.06; −1.47]
UPDRS III untreated	335	0.55	0.1221	74	0.61	0.3608	−0.36	[−0.62; −0.11]
UPDRS IV	301	0.26	0.1800	68	0.37	0.2256	−0.59	[−0.86; −0.33]
**Random effects model**	**1606**			**356**			**−1.66**	**[−2.78; −0.53]**
**Cohort = ICEBERG**								
UPDRS II	112	0.20	0.0313	42	0.39	0.0694	−4.20	[−4.79; −3.61]
UPDRS III off	111	0.04	0.0082	42	0.16	0.0983	−2.23	[−2.66; −1.79]
UPDRS III on	105	0.01	0.0023	41	0.09	0.0623	−2.55	[−3.02; −2.09]
UPDRS IV	112	0.07	0.1174	42	0.16	0.1752	−0.64	[−1.00; −0.27]
**Random effects model**	**440**			**167**			**−2.39**	**[−3.82; −0.96]**
**Random effects model**	**4036**			**1248**			**−2.03**	**[−2.69; −1.38]**

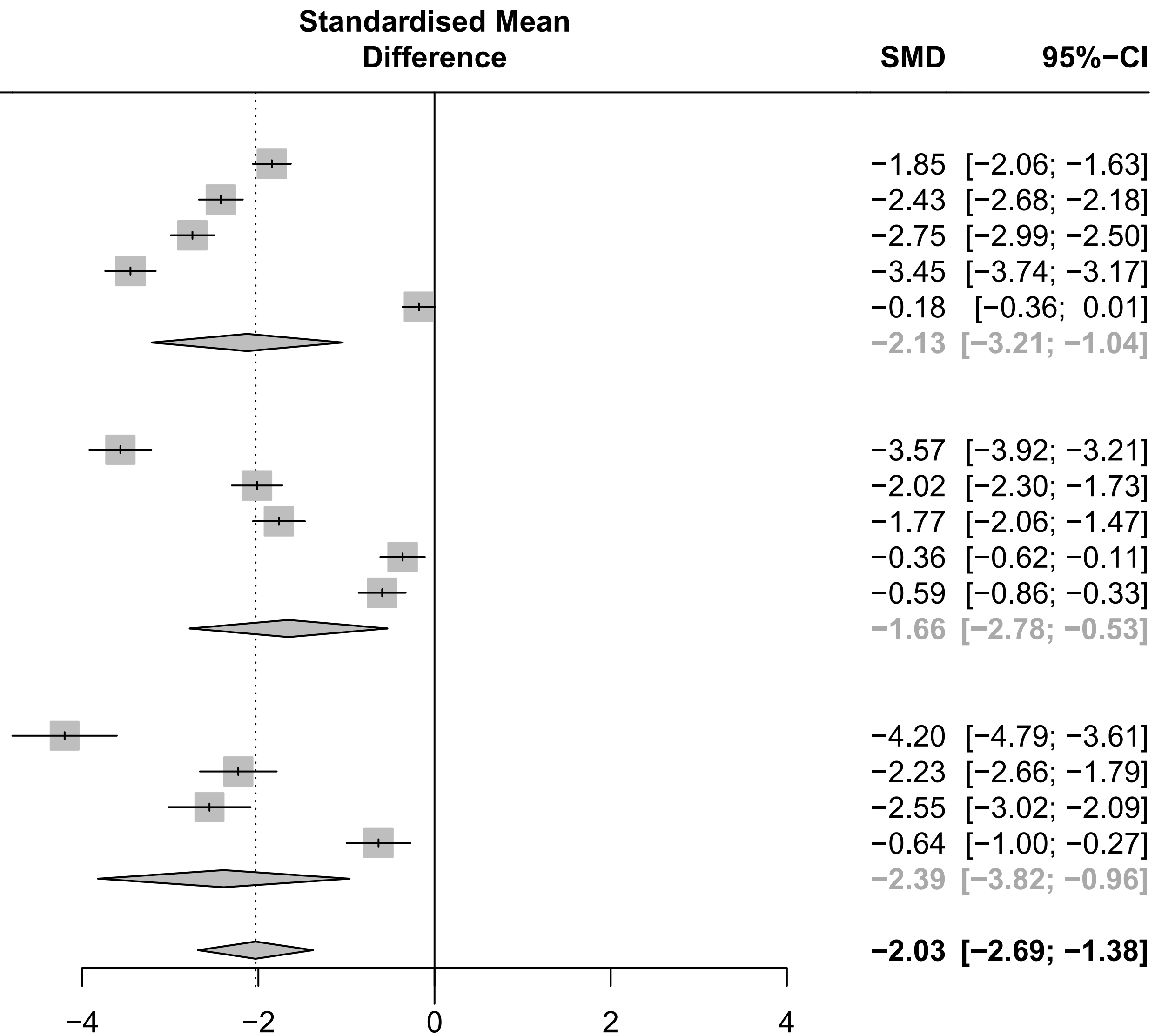

<- Faster progression for fast−progressing subtype | faster progression for slow−progressing subtype ->

Three−level metaanalysis using random effects to calculate an overall SMD estimate for Motor symptoms across cohorts. The dashed line indicates the overall mean SMD estimate. The solid line indicates no effect.

Forest plot for progression characteristics of symptom domain Impulsivity

Study	Slow progressing Total	Slow progressing Mean	Slow progressing SD	Fast progressing Total	Fast progressing Mean	Fast progressing SD	SMD	95%−CI
Cohort = PPMI								
QUIP	324	0.04	0.0547	72	0.07	0.1027	−0.41	[−0.67; −0.16]
Cohort = ICEBERG								
QUIP−RS	112	0.04	0.0690	42	0.09	0.0820	−0.67	[−1.03; −0.31]
**Random effects model**	**436**			**114**			**−0.51**	**[−0.75; −0.27]**

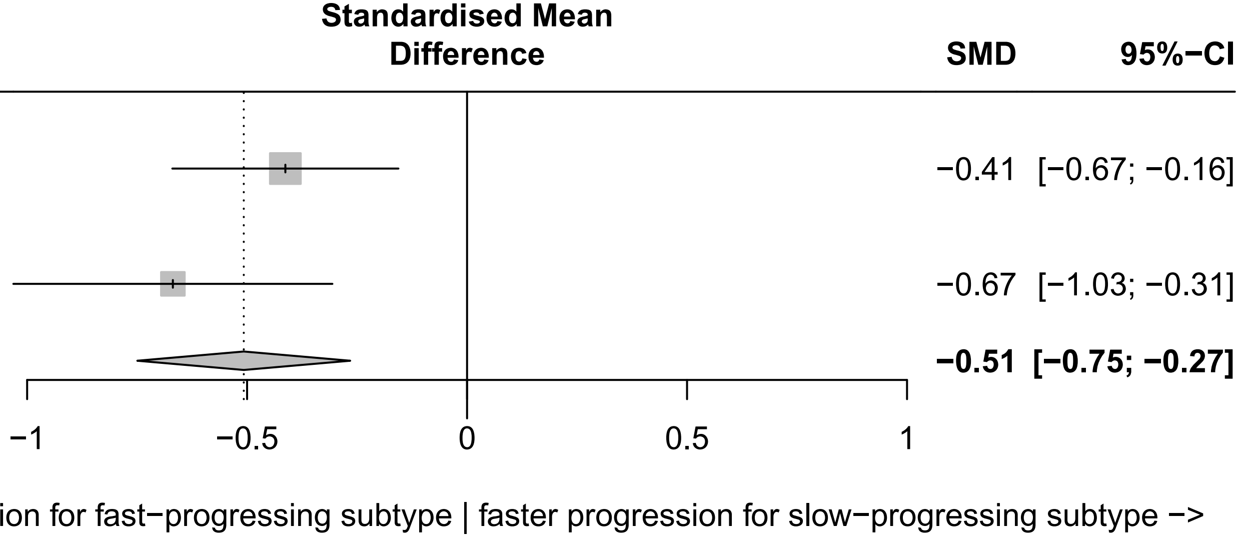

Three−level metaanalysis using random effects to calculate an overall SMD estimate for Impulsivity across cohorts.
The dashed line indicates the overall mean SMD estimate. The solid line indicates no effect.

Forest plots for symptom domain progression (cross-cohort validation)

Forest plot for progression characteristics of symptom domain Axial & PIGD (validation)

Study	Slow progressing Total	Slow progressing Mean	Slow progressing SD	Fast progressing Total	Fast progressing Mean	Fast progressing SD	SMD	95%−CI
**Cohort = ICEBERG (validation)**								
UPDRS III axial off	134	0.12	0.0154	19	0.54	0.2700	−4.47	[−5.16; −3.77]
UPDRS III axial on	127	0.13	0.0067	19	0.16	0.1915	−0.53	[−1.01; −0.04]
GABS Examination	135	0.05	0.0213	19	0.27	0.2062	−3.01	[−3.60; −2.42]
GABS Questionaire	135	0.04	0.0637	19	0.32	0.2745	−2.49	[−3.04; −1.93]
PIGD off	134	0.09	0.0457	19	0.37	0.1549	−4.13	[−4.80; −3.46]
PIGD on	127	0.09	0.0434	19	0.23	0.1583	−1.97	[−2.50; −1.44]
**Random effects model**	**792**			**114**			**−2.75**	**[−3.91; −1.59]**
**Cohort = LuxPARK (validation)**								
UPDRS III axial on	430	0.15	0.0629	90	0.34	0.1203	−2.59	[−2.87; −2.31]
FOGAC	70	0.09	0.0702	10	0.15	0.3365	−0.43	[−1.10; 0.23]
FOGQ	188	0.18	0.1030	42	0.43	0.3730	−1.36	[−1.72; −1.01]
PDQ39 Mobility	461	0.21	0.1023	98	0.51	0.2099	−2.40	[−2.66; −2.14]
PIGD on	430	0.15	0.0996	90	0.39	0.1639	−2.06	[−2.32; −1.80]
TuG	172	0.03	0.0320	24	0.07	0.1145	−0.76	[−1.19; −0.32]
**Random effects model**	**1751**			**354**			**−1.63**	**[−2.33; −0.93]**
**Random effects model**	**2543**			**468**			**−2.17**	**[−2.89; −1.45]**

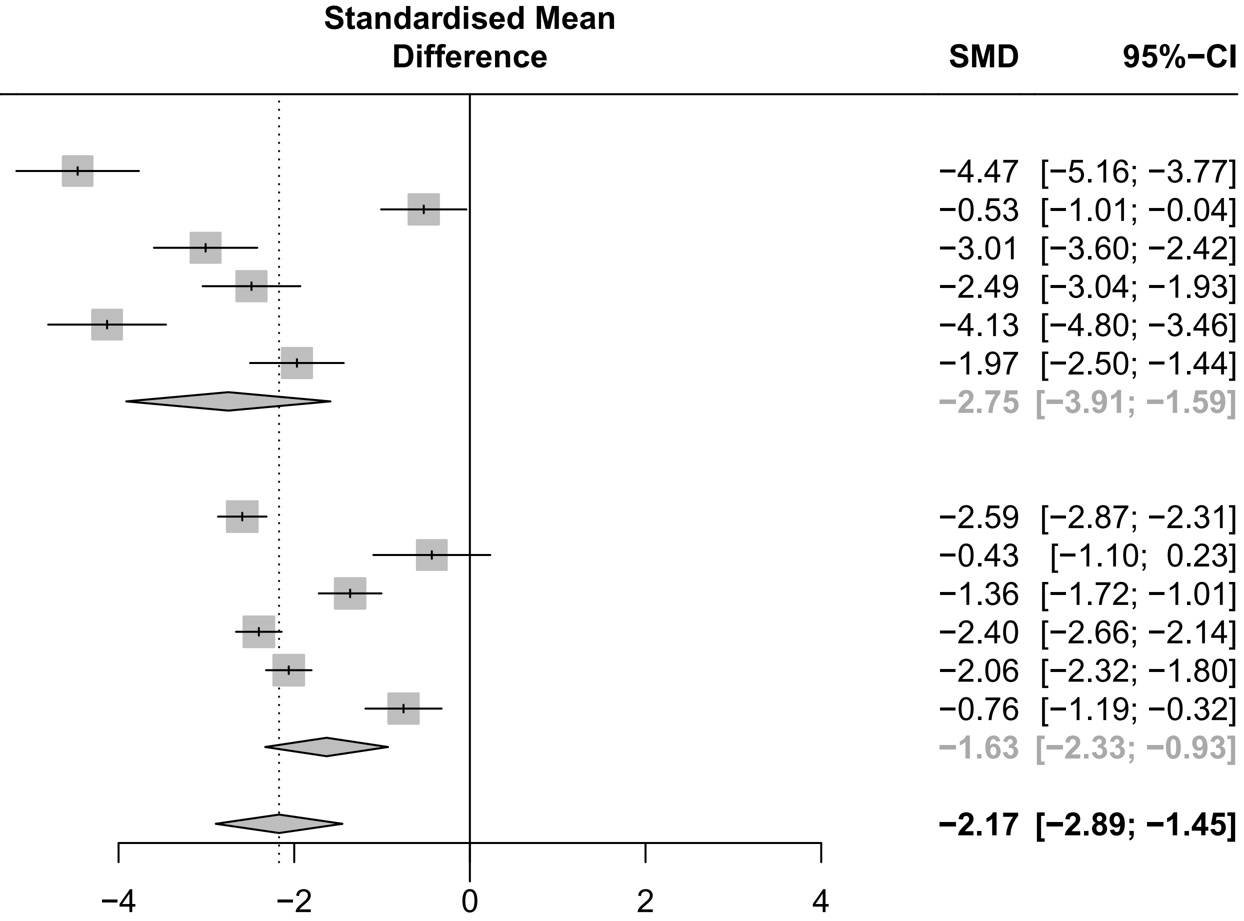

Three−level metaanalysis using random effects to calculate an overall SMD estimate for Axial & PIGD across cohorts. The dashed line indicates the overall mean SMD estimate. The solid line indicates no effect.

<− Faster progression for fast−progressing subtype | faster progression for slow−progressing subtype −>

Forest plot for progression characteristics of symptom domain Depression (validation)

Study	Slow progressing Total	Slow progressing Mean	Slow progressing SD	Fast progressing Total	Fast progressing Mean	Fast progressing SD	SMD	95%−CI
**Cohort = LuxPARK (validation)**								
BDI	459	0.06	0.0305	96	0.14	0.0120	−2.80	[−3.07; −2.52]
NMSQ Depression	462	1.19	0.4669	98	2.22	0.2138	−2.37	[−2.63; −2.11]
PDQ39 Depression	461	0.08	0.0500	98	0.16	0.0658	−1.60	[−1.84; −1.36]
UPDRS I Depression	462	0.92	0.2209	99	1.83	0.7230	−2.51	[−2.77; −2.25]
**Random effects model**	**1844**			**391**			**−2.32**	**[−2.82; −1.81]**
**Cohort = ICEBERG (validation)**								
HADS depression	135	0.11	0.0026	19	0.17	0.0615	−2.86	[−3.44; −2.28]
NMSQ Depression	135	1.83	0.2900	19	1.59	2.8964	0.22	[−0.26; 0.71]
UPDRS I Depression	135	1.32	0.7801	19	1.64	0.6916	−0.42	[−0.91; 0.06]
**Random effects model**	**405**			**57**			**−1.01**	**[−2.85; 0.82]**
**Random effects model**	**2249**			**448**			**−1.77**	**[−2.67; −0.87]**

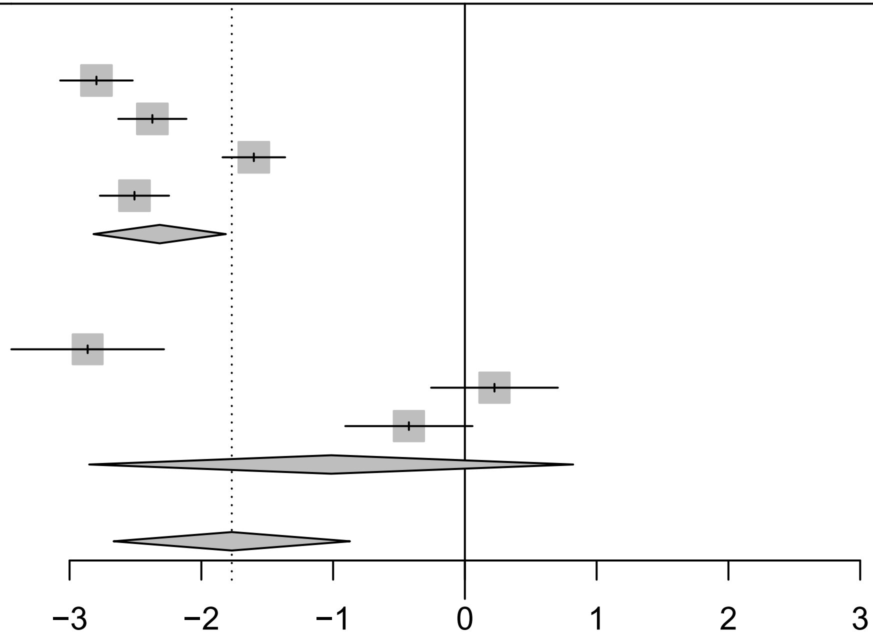

<− Faster progression for fast−progressing subtype | faster progression for slow−progressing subtype −>

Three−level metaanalysis using random effects to calculate an overall SMD estimate for Depression across cohorts.
The dashed line indicates the overall mean SMD estimate. The solid line indicates no effect.

Forest plot for progression characteristics of symptom domain Overall severity (validation)

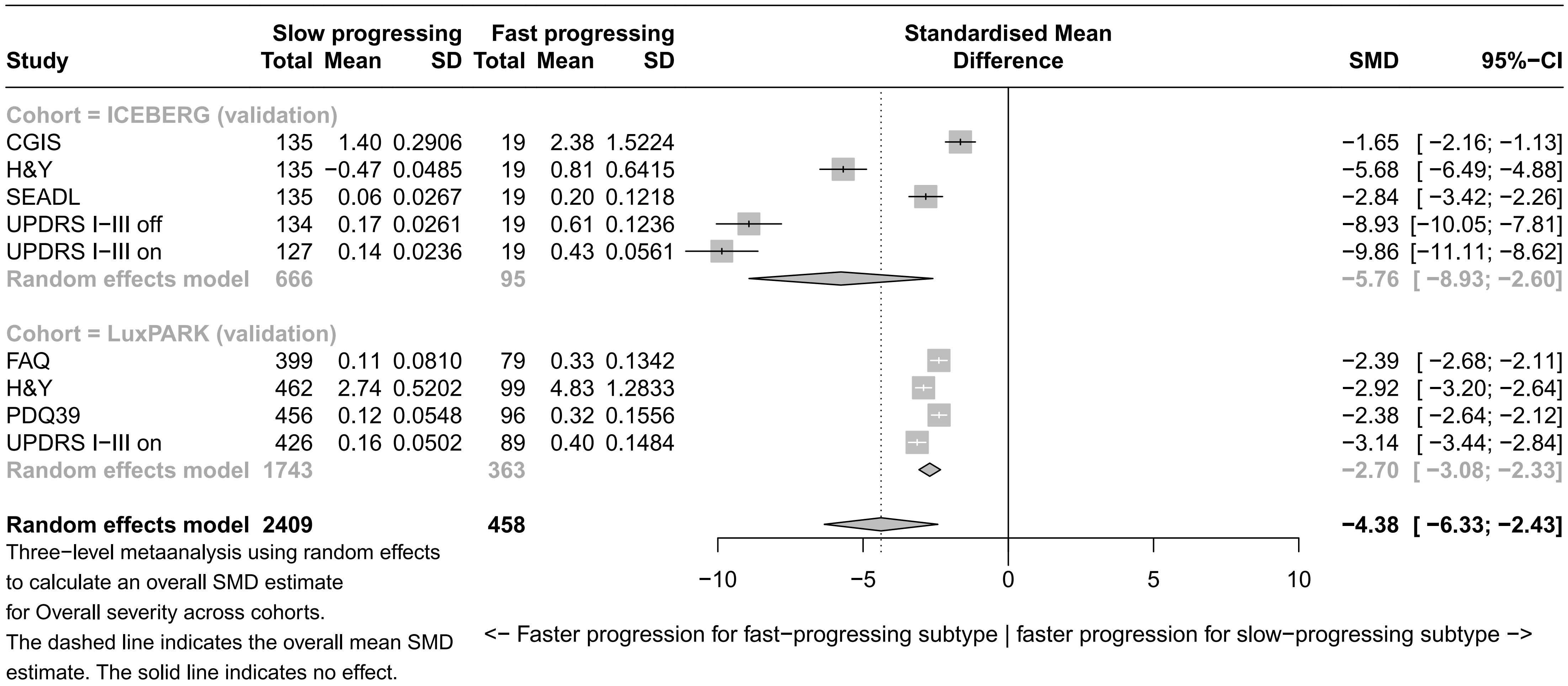

Forest plot for progression characteristics of symptom domain Apathy (validation)

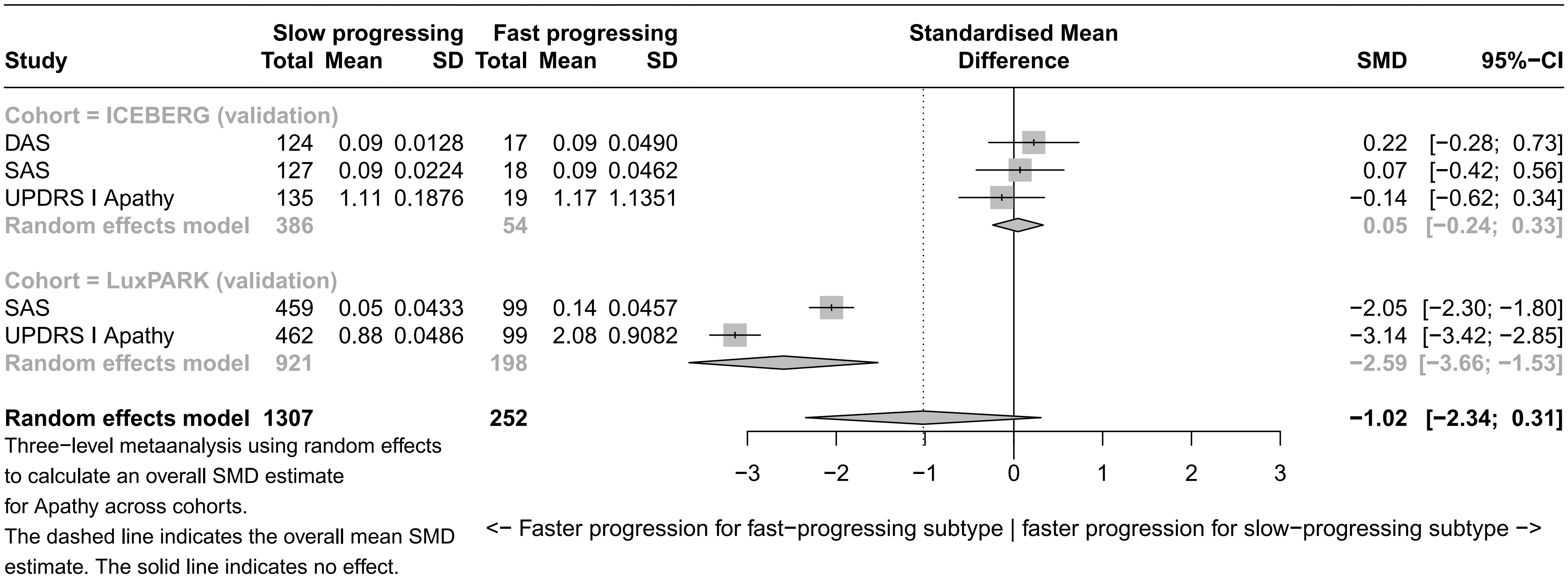

Forest plot for progression characteristics of symptom domain Sleep (validation)

Study	Slow progressing Total	Slow progressing Mean	Slow progressing SD	Fast progressing Total	Fast progressing Mean	Fast progressing SD	SMD	95%−CI
**Cohort = ICEBERG (validation)**								
ESS	133	0.17	0.1063	19	0.16	0.0548	0.09	[−0.39; 0.58]
NMSQ Sleep	135	1.21	0.2211	19	3.35	1.1076	−4.89	[−5.62; −4.16]
UPDRS I Sleep	135	0.08	0.0224	19	0.15	0.0255	−3.05	[−3.64; −2.45]
**Random effects model**	**403**			**57**			**−2.60**	**[−5.46; 0.25]**
**Cohort = LuxPARK (validation)**								
NMSQ Sleep	462	0.68	0.0575	98	0.76	0.3560	−0.53	[−0.75; −0.31]
PDSS	460	0.11	0.0237	98	0.12	0.0355	−0.51	[−0.73; −0.29]
UPDRS I Sleep	462	0.10	0.0325	99	0.12	0.0353	−0.64	[−0.86; −0.42]
**Random effects model**	**1384**			**295**			**−0.56**	**[−0.69; −0.43]**
**Random effects model**	**1787**			**352**			**−1.57**	**[−3.11; −0.03]**

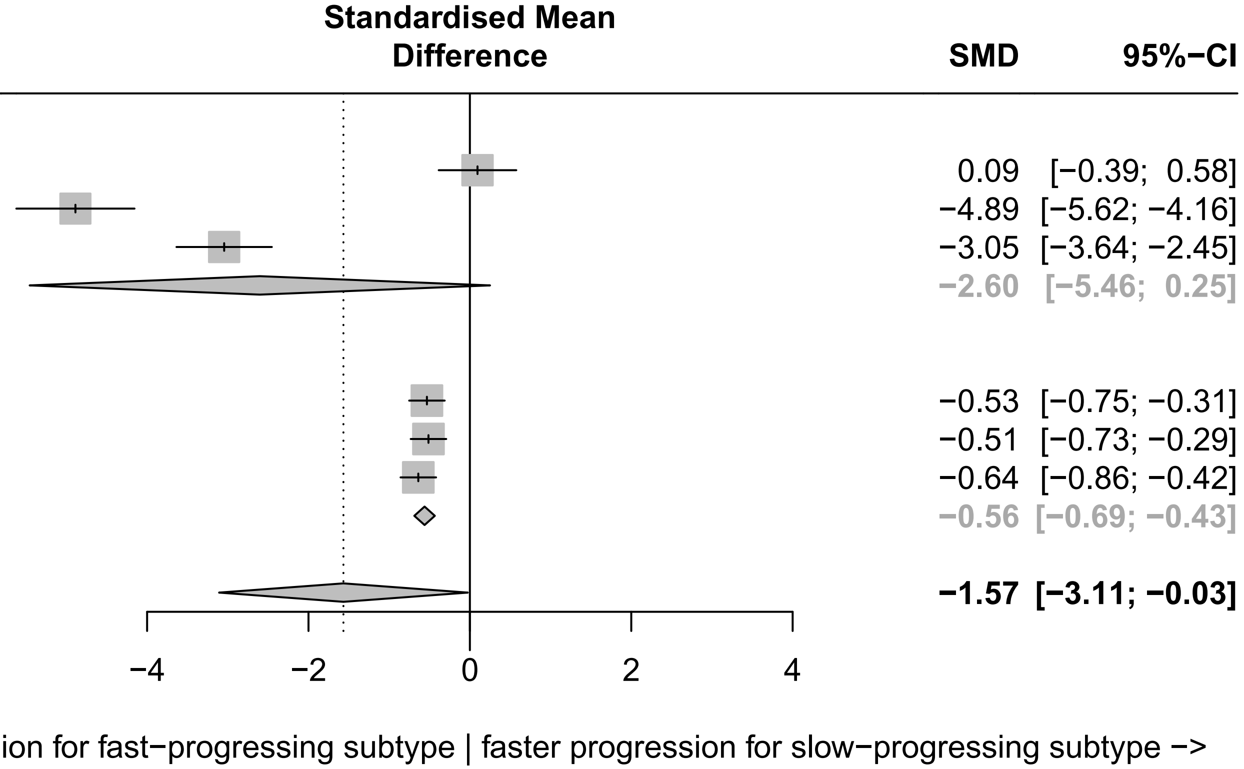

Three−level metaanalysis using random effects to calculate an overall SMD estimate for Sleep across cohorts.
The dashed line indicates the overall mean SMD estimate. The solid line indicates no effect.

Forest plot for progression characteristics of symptom domain Overall cognition (validation)

Study	Slow progressing Total	Slow progressing Mean	Slow progressing SD	Fast progressing Total	Fast progressing Mean	Fast progressing SD	SMD	95%−CI
Cohort = LuxPARK (validation)								
FAB	48	0.03	0.0435	9	0.23	0.2836	−1.69	[−2.47; −0.91]
MoCA	462	0.04	0.0278	99	0.21	0.1602	−2.31	[−2.57; −2.06]
NMSQ Cognition	462	1.36	0.5848	97	3.17	1.3498	−2.34	[−2.60; −2.08]
PDQ39 Cognition	461	0.08	0.0310	99	0.18	0.1010	−1.95	[−2.19; −1.70]
SIQCDE	396	0.04	0.0408	77	0.10	0.0812	−1.16	[−1.42; −0.91]
UPDRS I Cognition	462	1.21	0.1909	99	3.13	1.2061	−3.58	[−3.88; −3.28]
Random effects model	2291			480			−2.18	[−2.85; −1.52]
Cohort = ICEBERG (validation)								
FAB	127	0.05	0.0383	18	0.01	0.0697	1.03	[0.53; 1.54]
MATTIS	127	0.04	0.0346	18	0.07	0.0661	−0.71	[−1.22; −0.21]
MoCA	135	0.05	0.0503	19	0.15	0.0817	−1.80	[−2.33; −1.28]
NMSQ Cognition	135	1.85	0.4908	19	1.30	1.3777	0.83	[0.34; 1.32]
UPDRS I Cognition	135	0.87	0.8917	19	1.09	1.5180	−0.22	[−0.70; 0.26]
Random effects model	659			93			−0.17	[−1.19; 0.85]
**Random effects model**	**2950**			**573**			**−1.27**	**[−2.10; −0.44]**

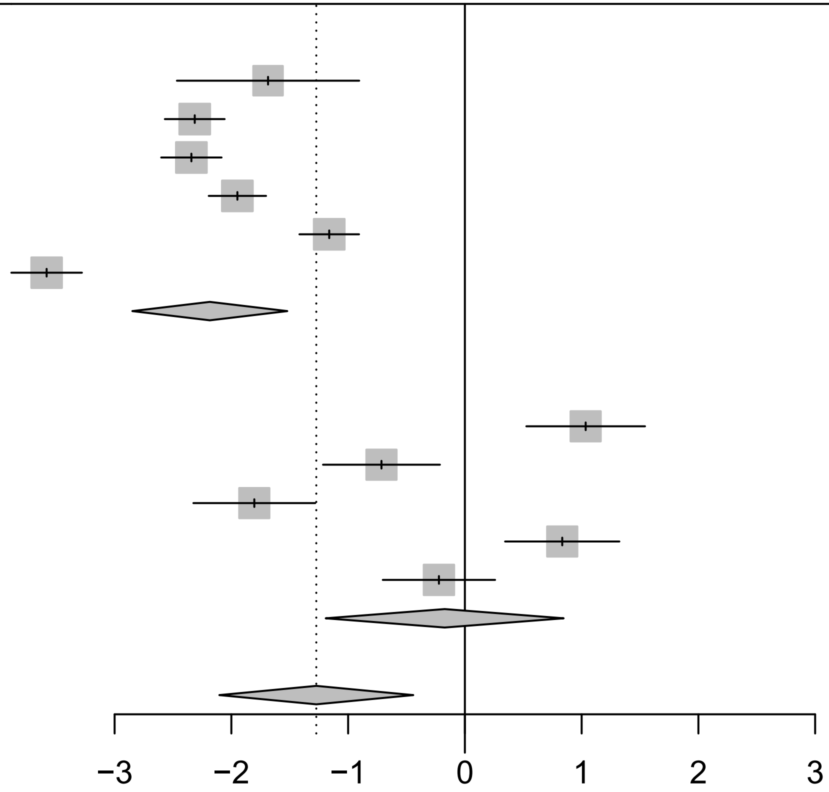

Three−level metaanalysis using random effects to calculate an overall SMD estimate for Overall cognition across cohorts. The dashed line indicates the overall mean SMD estimate. The solid line indicates no effect.

Forest plot for progression characteristics of symptom domain Conceptualization (validation)

Study	Slow progressing Total	Slow progressing Mean	Slow progressing SD	Fast progressing Total	Fast progressing Mean	Fast progressing SD	SMD	95%−CI
**Cohort = ICEBERG (validation)**								
FAB Conceptualization	127	2.45	1.5170	18	0.60	0.2456	1.29	[0.78; 1.81]
MATTIS Conzeptualization	127	0.01	0.0596	18	0.07	0.0491	−1.17	[−1.68; −0.65]
MOCA Abstraction	135	1.01	0.9387	19	0.99	0.2560	0.02	[−0.46; 0.50]
**Random effects model**	**389**			**55**			**0.05**	**[−1.34; 1.44]**
**Cohort = LuxPARK (validation)**								
FAB Conceptualization	49	−1.64	0.9250	10	−0.35	2.5093	−0.97	[−1.67; −0.26]
MOCA Abstraction	462	0.17	0.2063	99	0.41	0.4000	−0.93	[−1.16; −0.71]
**Random effects model**	**511**			**109**			**−0.94**	**[−1.15; −0.72]**
**Random effects model**	**900**			**164**			**−0.35**	**[−1.25; 0.55]**

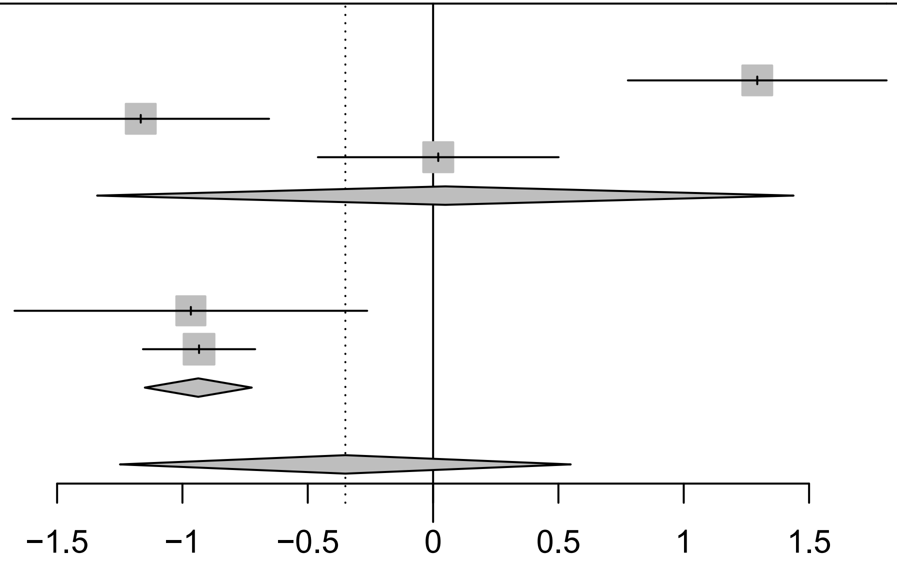

<− Faster progression for fast−progressing subtype | faster progression for slow−progressing subtype −>

Three−level metaanalysis using random effects to calculate an overall SMD estimate for Conceptualization across cohorts. The dashed line indicates the overall mean SMD estimate. The solid line indicates no effect.

Forest plot for progression characteristics of symptom domain Visuo−executive (validation)

Study	Slow progressing Total	Slow progressing Mean	Slow progressing SD	Fast progressing Total	Fast progressing Mean	Fast progressing SD	SMD	95%−CI
**Cohort = LuxPARK (validation)**								
FAB 3−6	48	0.06	0.0214	9	0.22	0.1676	−2.32	[−3.16; −1.48]
MOCA Visuspatial/Executive	462	1.00	0.0008	99	2.73	0.5962	−6.92	[−7.38; −6.46]
Trailmaking A	440	0.02	0.0161	86	0.10	0.1102	−1.72	[−1.97; −1.46]
Trailmaking B	396	0.04	0.0216	60	0.10	0.0701	−1.75	[−2.05; −1.46]
**Random effects model**	**1346**			**254**			**−3.18**	**[−5.64; −0.71]**
**Cohort = ICEBERG (validation)**								
FAB 3−6	127	0.06	0.0507	18	0.01	0.0948	0.89	[0.39; 1.40]
MATTIS Initiation + Construction	127	0.03	0.0602	18	0.07	0.0242	−0.64	[−1.14; −0.14]
MOCA Visuspatial/Executive	135	0.69	0.6431	19	2.93	2.0152	−2.42	[−2.97; −1.87]
**Random effects model**	**389**			**55**			**−0.72**	**[−2.59; 1.16]**
**Random effects model**	**1735**			**309**			**−2.12**	**[−3.91; −0.34]**

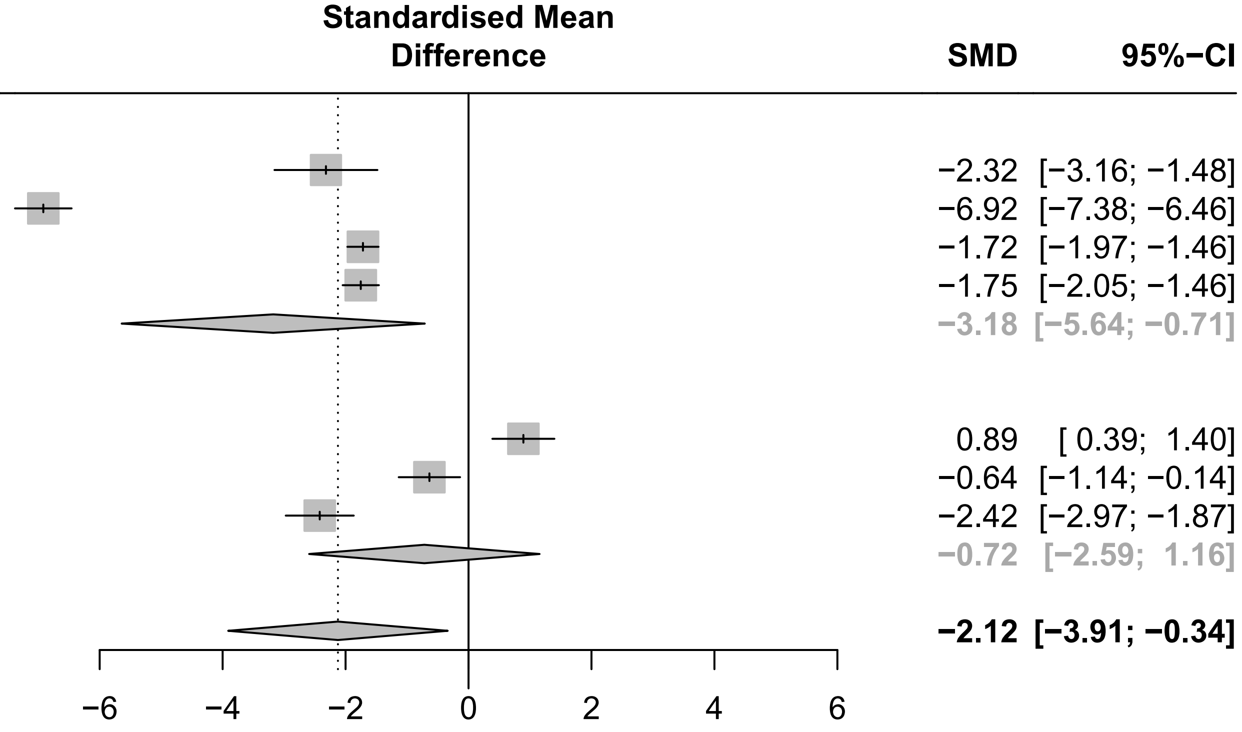

<− Faster progression for fast−progressing subtype | faster progression for slow−progressing subtype −>

Three−level metaanalysis using random effects to calculate an overall SMD estimate for Visuo−executive across cohorts. The dashed line indicates the overall mean SMD estimate. The solid line indicates no effect.

Forest plot for progression characteristics of symptom domain Language (validation)

	Slow progressing			Fast progressing				
**Study**	**Total**	**Mean**	**SD**	**Total**	**Mean**	**SD**	**SMD**	**95%−CI**
**Cohort = LuxPARK (validation)**								
FAB VFT	49	0.61	2.3727	10	2.27	1.7866	−0.71	[−1.41; −0.02]
MOCA Language + Naming	462	0.04	0.0173	99	0.18	0.0729	−4.19	[−4.52; −3.86]
VFT phonematic F	374	−0.00	0.0089	87	0.06	0.0011	−7.27	[−7.80; −6.75]
VFT phonematic S	57	−0.02	0.0422	14	0.17	0.1257	−2.94	[−3.71; −2.18]
**Random effects model**	**942**			**210**			**−3.79**	**[−6.47; −1.11]**
**Cohort = ICEBERG (validation)**								
FAB VFT	127	−0.43	0.5594	18	0.38	0.0389	−1.53	[−2.06; −1.01]
MOCA Language + Naming	135	0.03	0.0221	19	0.08	0.0620	−1.62	[−2.14; −1.11]
VFT phonematic F	135	0.03	0.0352	19	0.04	0.0665	−0.27	[−0.75; 0.22]
VFT phonematic S	123	0.00	0.0105	18	0.03	0.0003	−2.59	[−3.17; −2.01]
VFT semantic supermarket	122	0.05	0.0317	18	0.04	0.0017	0.40	[−0.10; 0.89]
**Random effects model**	**642**			**92**			**−1.12**	**[−2.15; −0.08]**
**Random effects model**	**1584**			**302**			**−2.31**	**[−3.84; −0.77]**

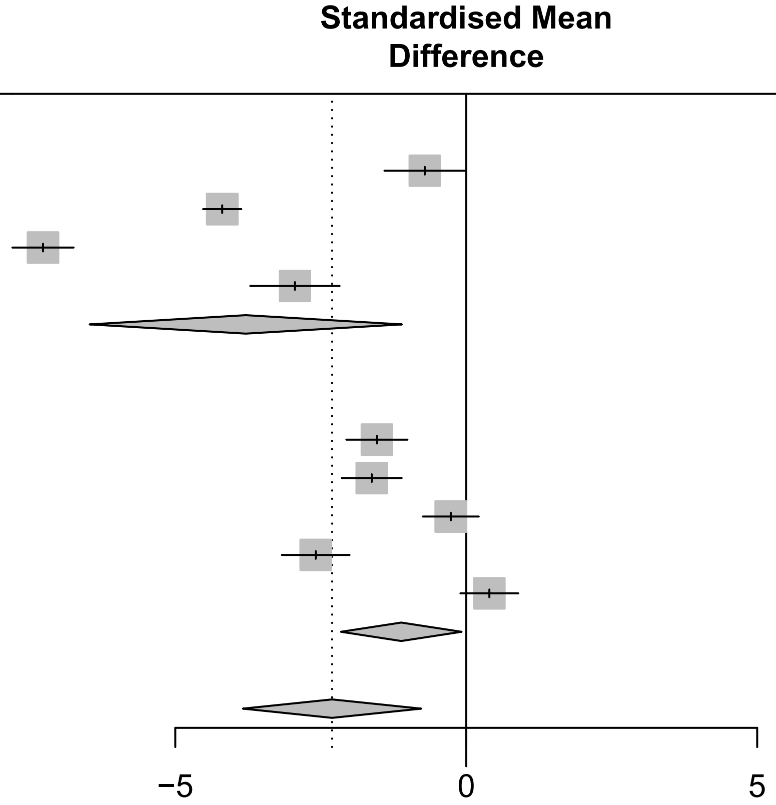

<− Faster progression for fast−progressing subtype | faster progression for slow−progressing subtype −>

Three−level metaanalysis using random effects to calculate an overall SMD estimate for Language across cohorts.
The dashed line indicates the overall mean SMD estimate. The solid line indicates no effect.

Forest plot for progression characteristics of symptom domain Anxiety (validation)

Study	Slow progressing Total	Slow progressing Mean	Slow progressing SD	Fast progressing Total	Fast progressing Mean	Fast progressing SD	SMD	95%−CI
**Cohort = ICEBERG (validation)**								
HADS anxiety	135	0.03	0.0129	19	0.09	0.0045	−4.57	[−5.28; −3.87]
NMSQ Anxiety	134	1.14	0.3856	19	2.46	2.1622	−1.58	[−2.09; −1.07]
UPDRS I Anxiety	135	0.89	0.0973	19	1.66	2.5099	−0.88	[−1.37; −0.39]
**Random effects model**	**404**			**57**			**−2.33**	**[−4.54; −0.12]**
**Cohort = LuxPARK (validation)**								
NMSQ Anxiety	462	1.15	0.3264	97	1.40	0.2377	−0.79	[−1.02; −0.57]
PDQ39 Anxiety	462	1.10	0.2517	99	1.78	1.2006	−1.23	[−1.46; −1.00]
UPDRS I Anxiety	462	0.73	0.2466	99	0.89	0.7241	−0.41	[−0.63; −0.19]
**Random effects model**	**1386**			**295**			**−0.81**	**[−1.27; −0.34]**
**Random effects model**	**1790**			**352**			**−1.55**	**[−2.74; −0.37]**

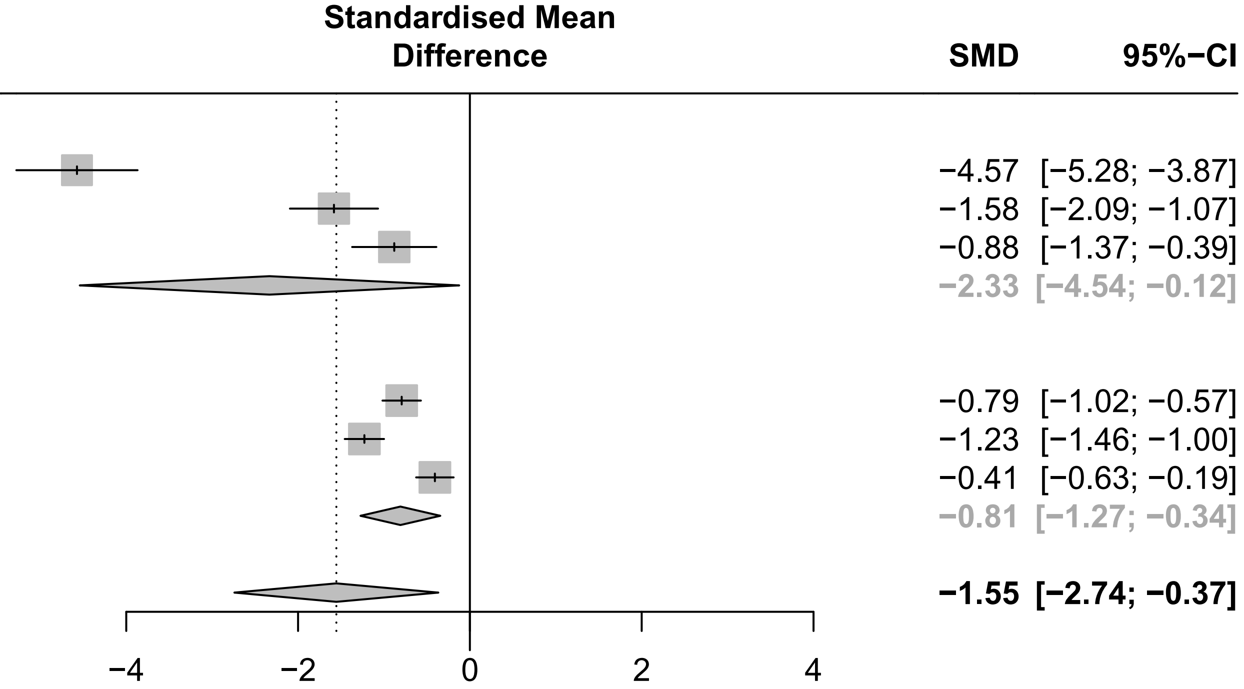

<− Faster progression for fast−progressing subtype | faster progression for slow−progressing subtype −>

Three−level metaanalysis using random effects to calculate an overall SMD estimate for Anxiety across cohorts. The dashed line indicates the overall mean SMD estimate. The solid line indicates no effect.

Forest plot for progression characteristics of symptom domain Memory (validation)

Study	Slow progressing Total	Slow progressing Mean	Slow progressing SD	Fast progressing Total	Fast progressing Mean	Fast progressing SD	SMD	95%−CI
**Cohort = ICEBERG (validation)**								
MATTIS Memory	127	0.07	0.0498	18	0.09	0.0571	−0.48	[−0.98; 0.01]
MOCA Orientation + Memory	135	0.01	0.0232	19	0.06	0.0612	−1.66	[−2.18; −1.14]
NMSQ Memory	134	1.37	1.2394	19	1.95	2.0623	−0.43	[−0.91; 0.06]
**Random effects model**	**396**			**56**			**−0.85**	**[−1.64; −0.07]**
**Cohort = LuxPARK (validation)**								
MOCA Orientation + Memory	462	0.01	0.0199	99	0.14	0.1483	−1.91	[−2.15; −1.67]
NMSQ Memory	462	1.18	0.1844	97	2.77	1.1996	−3.02	[−3.30; −2.74]
SIQCDE Memory	397	0.04	0.0417	77	0.08	0.0691	−0.93	[−1.18; −0.68]
**Random effects model**	**1321**			**273**			**−1.95**	**[−3.14; −0.77]**
**Random effects model**	**1717**			**329**			**−1.42**	**[−2.22; −0.62]**

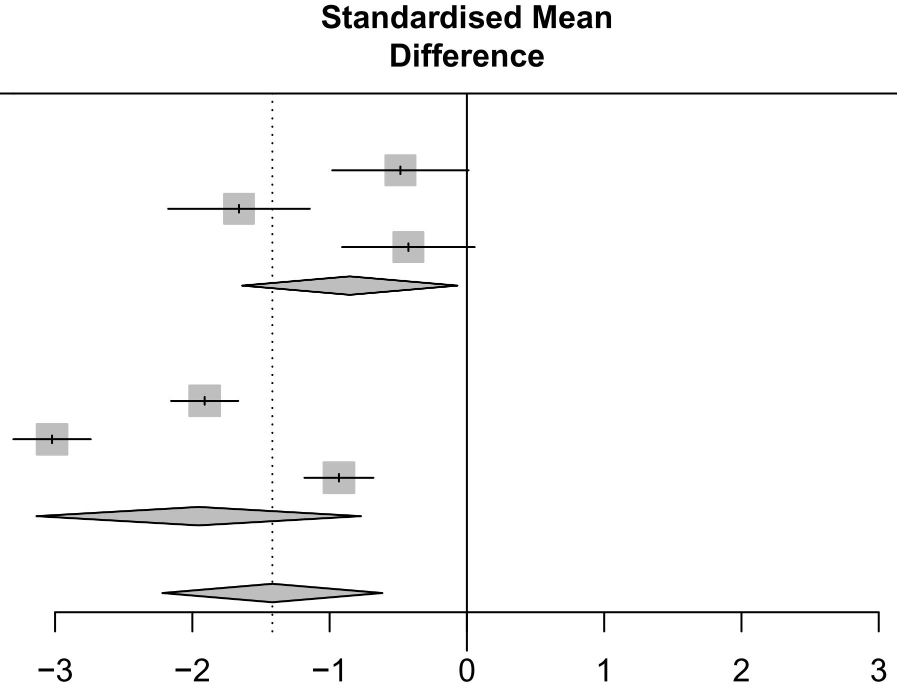

<− Faster progression for fast−progressing subtype | faster progression for slow−progressing subtype −>

Three−level metaanalysis using random effects to calculate an overall SMD estimate for Memory across cohorts.
The dashed line indicates the overall mean SMD estimate. The solid line indicates no effect.

Forest plot for progression characteristics of symptom domain Attention (validation)

Study	Slow progressing Total	Slow progressing Mean	Slow progressing SD	Fast progressing Total	Fast progressing Mean	Fast progressing SD	SMD	95%−CI
Cohort = ICEBERG (validation)								
MATTIS Attention	127	0.01	0.0320	18	−0.07	0.0070	2.63	[2.05; 3.21]
MOCA Attention	135	0.63	0.1829	19	1.40	1.5398	−1.37	[−1.88; −0.87]
NMSQ Attention	134	2.26	0.0973	19	0.47	0.8163	6.02	[5.18; 6.85]
Random effects model	396			56			2.41	[−1.77; 6.60]
Cohort = LuxPARK (validation)								
MOCA Attention	462	0.67	0.2059	99	2.00	0.8758	−3.22	[−3.50; −2.93]
NMSQ Attention	462	1.53	0.9835	98	2.55	0.6917	−1.07	[−1.30; −0.85]
PDQ39 Attention	462	1.41	0.1286	99	1.88	0.9219	−1.17	[−1.40; −0.94]
SIQCDE Attention	398	0.58	0.4896	77	1.30	0.8721	−1.26	[−1.52; −1.00]
Random effects model	1784			373			−1.68	[−2.68; −0.67]
**Random effects model**	**2180**			**429**			**0.06**	**[−2.25; 2.38]**

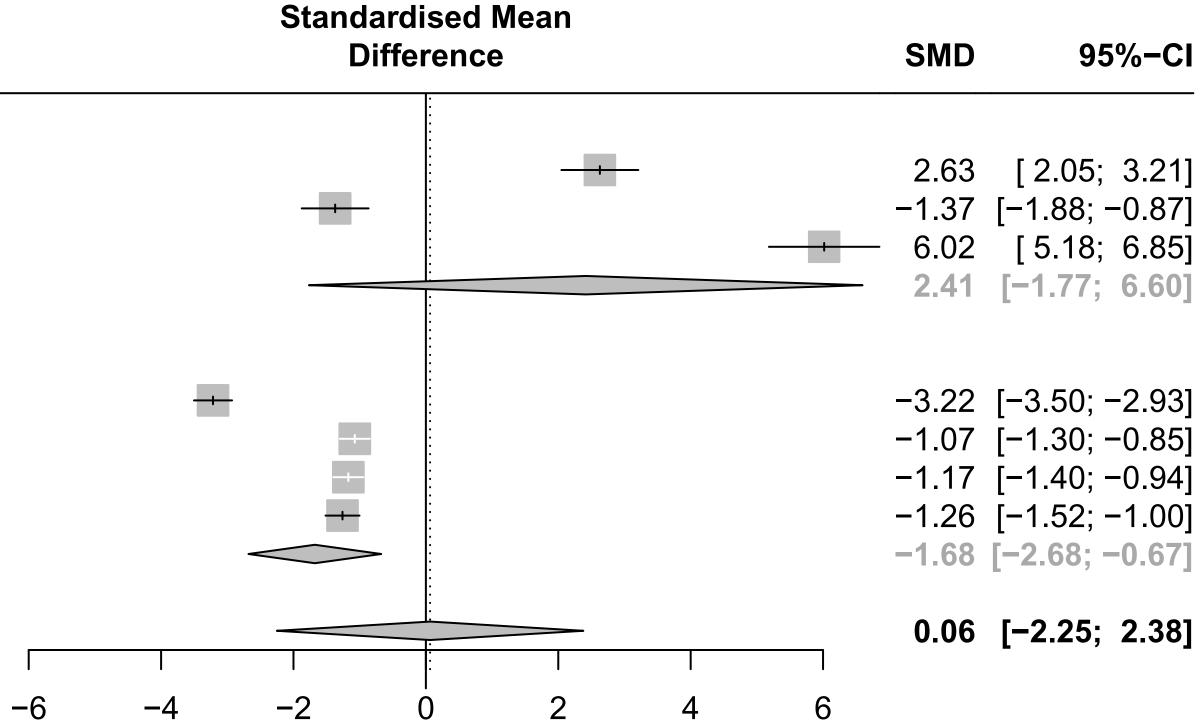

Three−level metaanalysis using random effects to calculate an overall SMD estimate for Attention across cohorts.
The dashed line indicates the overall mean SMD estimate. The solid line indicates no effect.

<− Faster progression for fast−progressing subtype | faster progression for slow−progressing subtype −>

Forest plot for progression characteristics of symptom domain Non motor symptoms (validation)

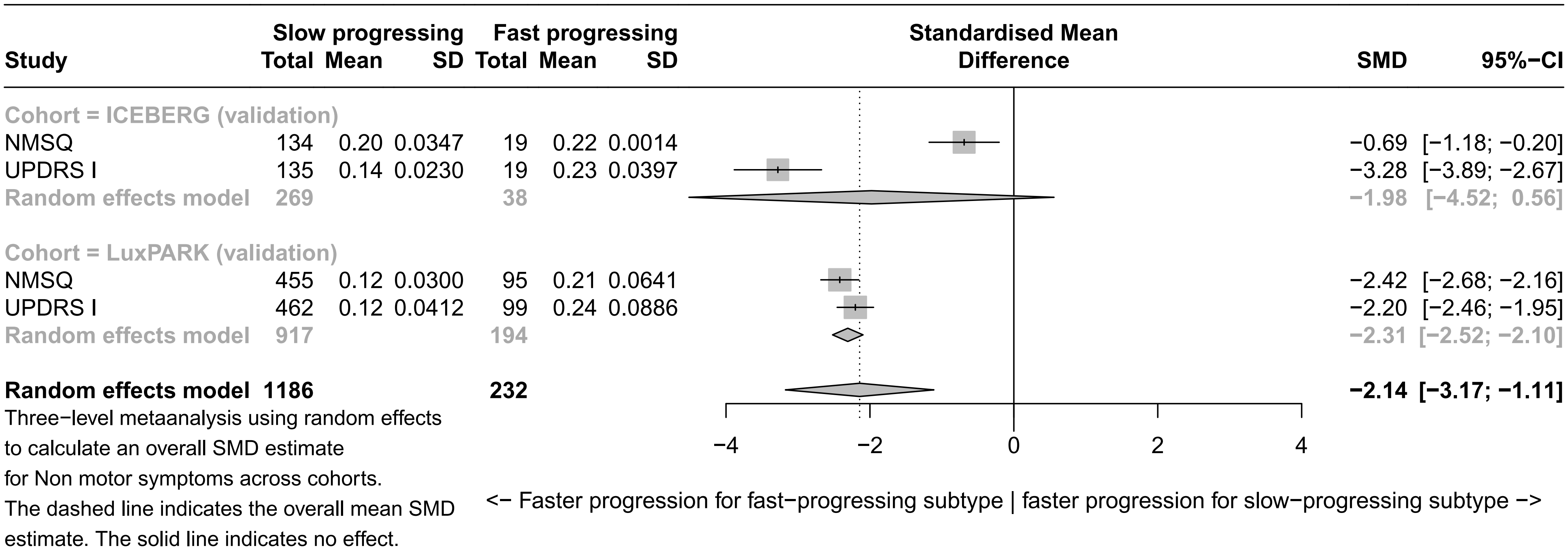

Forest plot for progression characteristics of symptom domain Autonomic (validation)

Study	Slow progressing Total	Slow progressing Mean	Slow progressing SD	Fast progressing Total	Fast progressing Mean	Fast progressing SD	SMD	95%−CI
**Cohort = ICEBERG (validation)**								
NMSQ Autonomic	135	0.19	0.0132	19	0.16	0.0734	1.21	[0.71; 1.71]
SCOPA−AUT	135	0.19	0.0481	19	0.28	0.0830	−1.71	[−2.22; −1.19]
UPDRS I Autonomic	135	0.20	0.0600	19	0.21	0.0328	−0.28	[−0.76; 0.20]
**Random effects model**	**405**			**57**			**−0.26**	**[−1.91; 1.39]**
**Cohort = LuxPARK (validation)**								
NMSQ Autonomic	456	0.13	0.0288	97	0.15	0.0716	−0.62	[−0.84; −0.39]
SCOPA−AUT	462	0.13	0.0363	99	0.17	0.0566	−0.91	[−1.13; −0.68]
UPDRS I Autonomic	462	0.13	0.0428	99	0.22	0.0693	−1.85	[−2.09; −1.60]
**Random effects model**	**1380**			**295**			**−1.12**	**[−1.85; −0.39]**
**Random effects model**	**1785**			**352**			**−0.70**	**[−1.58; 0.18]**

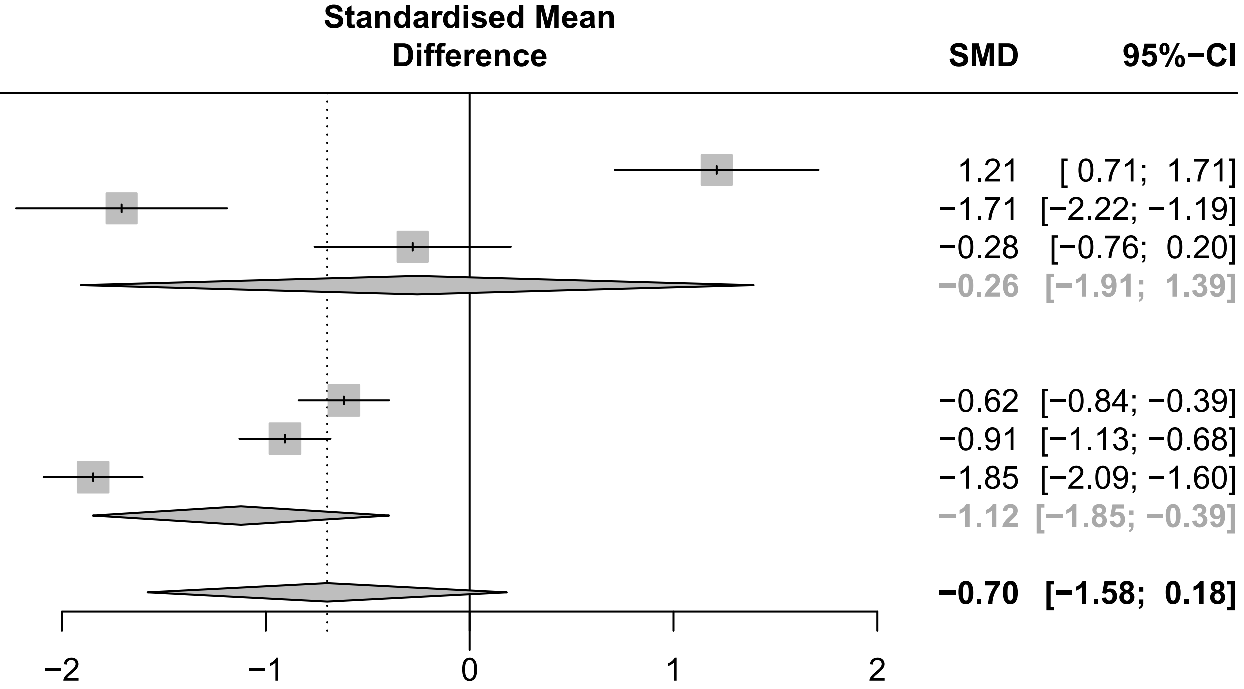

<− Faster progression for fast−progressing subtype | faster progression for slow−progressing subtype −>

Three−level metaanalysis using random effects to calculate an overall SMD estimate for Autonomic across cohorts.
The dashed line indicates the overall mean SMD estimate. The solid line indicates no effect.

Forest plot for progression characteristics of symptom domain Hallucinations (validation)

Study	Slow progressing Total	Slow progressing Mean	Slow progressing SD	Fast progressing Total	Fast progressing Mean	Fast progressing SD	SMD	95%−CI
**Cohort = LuxPARK (validation)**								
NMSQ Hallucination	462	1.67	0.2022	98	1.81	1.0142	−0.30	[−0.52; −0.09]
UPDRS I Hallucinations	462	1.31	0.2786	99	1.38	0.8747	−0.16	[−0.38; 0.06]
**Random effects model**	**924**			**197**			**−0.23**	**[−0.39; −0.08]**
**Cohort = ICEBERG (validation)**								
NMSQ Hallucination	135	0.93	0.9204	19	1.38	0.7406	−0.50	[−0.99; −0.02]
UPDRS I Hallucinations	135	1.03	2.0070	19	8.38	9.9115	−1.88	[−2.40; −1.35]
**Random effects model**	**270**			**38**			**−1.19**	**[−2.53; 0.16]**
**Random effects model**	**1194**			**235**			**−0.69**	**[−1.44; 0.06]**

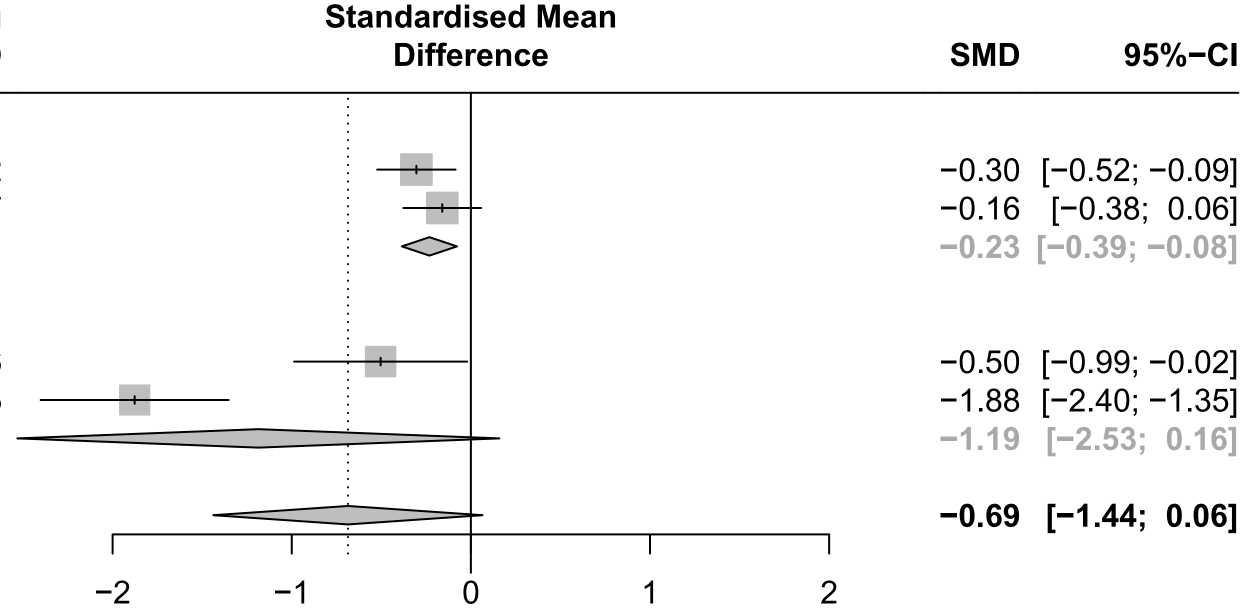

Three−level metaanalysis using random effects to calculate an overall SMD estimate for Hallucinations across cohorts. The dashed line indicates the overall mean SMD estimate. The solid line indicates no effect.

<− Faster progression for fast−progressing subtype | faster progression for slow−progressing subtype −>

Forest plot for progression characteristics of symptom domain Pain (validation)

Study	Slow progressing Total	Slow progressing Mean	Slow progressing SD	Fast progressing Total	Fast progressing Mean	Fast progressing SD	SMD	95%−CI
**Cohort = LuxPARK (validation)**								
NMSQ Pain	462	0.79	0.0950	98	1.35	0.0765	−6.03	[−6.45; −5.62]
PDQ39 Pain	461	0.11	0.0314	99	0.12	0.0169	−0.29	[−0.51; −0.08]
UPDRS I Pain	462	1.17	0.2438	99	1.69	0.8251	−1.26	[−1.49; −1.03]
**Random effects model**	**1385**			**296**			**−2.53**	**[−6.00; 0.95]**
**Cohort = ICEBERG (validation)**								
NMSQ Pain	134	0.68	0.3044	19	0.81	0.4887	−0.39	[−0.88; 0.09]
UPDRS I Pain	135	1.06	0.1449	19	0.69	0.1723	2.47	[1.92; 3.03]
**Random effects model**	**269**			**38**			**1.04**	**[−1.77; 3.85]**
**Random effects model**	**1654**			**334**			**−1.11**	**[−3.81; 1.60]**

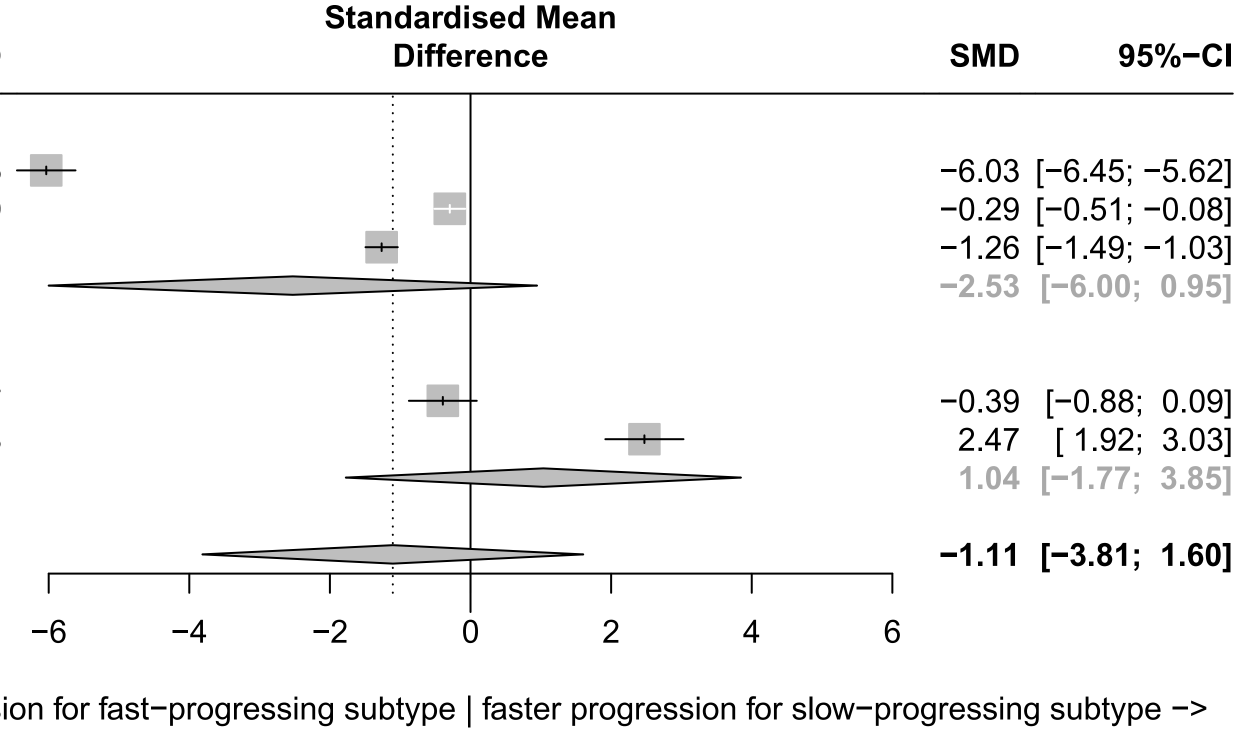

Three−level metaanalysis using random effects to calculate an overall SMD estimate for Pain across cohorts.
The dashed line indicates the overall mean SMD estimate. The solid line indicates no effect.

Forest plot for progression characteristics of symptom domain RBD (validation)

Study	Slow progressing Total	Slow progressing Mean	Slow progressing SD	Fast progressing Total	Fast progressing Mean	Fast progressing SD	SMD	95%−CI
**Cohort = LuxPARK (validation)**								
NMSQ RBD	461	1.57	0.1984	98	0.82	0.9326	1.75	[1.51; 1.99]
RBD−SQ	459	0.10	0.0627	98	0.05	0.0972	0.79	[0.56; 1.01]
**Random effects model**	**920**			**196**			**1.27**	**[0.32; 2.21]**
**Cohort = ICEBERG (validation)**								
NMSQ RBD	135	1.65	0.5255	19	1.19	2.0199	0.54	[0.05; 1.02]
RBD−HK	133	0.07	0.0245	19	−0.01	0.1946	1.02	[0.53; 1.51]
**Random effects model**	**268**			**38**			**0.78**	**[0.30; 1.25]**
**Random effects model**	**1188**			**234**			**1.04**	**[0.51; 1.57]**

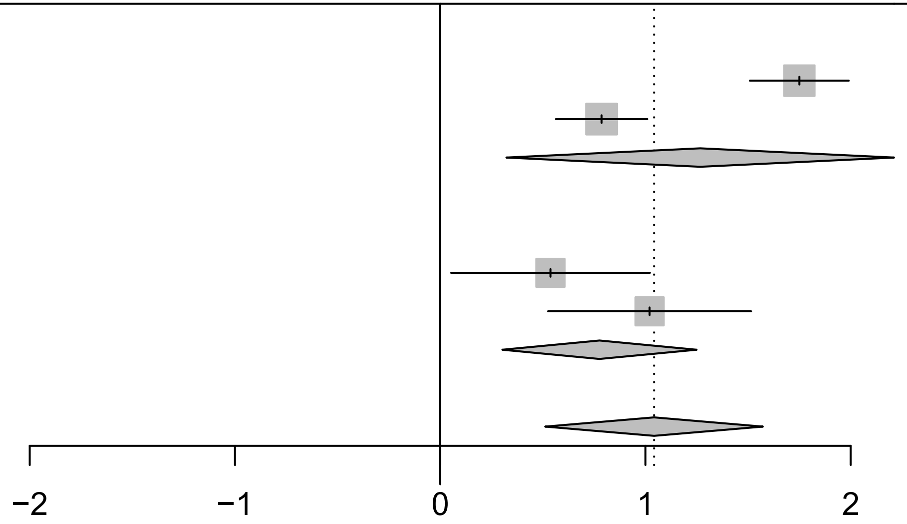

Three−level metaanalysis using random effects to calculate an overall SMD estimate for RBD across cohorts.
The dashed line indicates the overall mean SMD estimate. The solid line indicates no effect.

Forest plot for progression characteristics of symptom domain Smell (validation)

Study	Slow progressing Total	Slow progressing Mean	Slow progressing SD	Fast progressing Total	Fast progressing Mean	Fast progressing SD	SMD	95%−CI
**Cohort = LuxPARK (validation)**								
NMSQ Smell	462	0.58	0.0529	98	0.19	0.7260	1.28	[1.05; 1.51]
Sniffin Test	443	0.04	0.0063	94	0.08	0.0285	−2.79	[−3.07; −2.51]
**Random effects model**	**905**			**192**			**−0.75**	**[−4.75; 3.24]**
**Cohort = ICEBERG (validation)**								
NMSQ Smell	134	0.58	0.2972	19	−1.47	2.1280	2.60	[2.04; 3.16]
**Random effects model**	**1039**			**211**			**0.36**	**[−2.82; 3.54]**

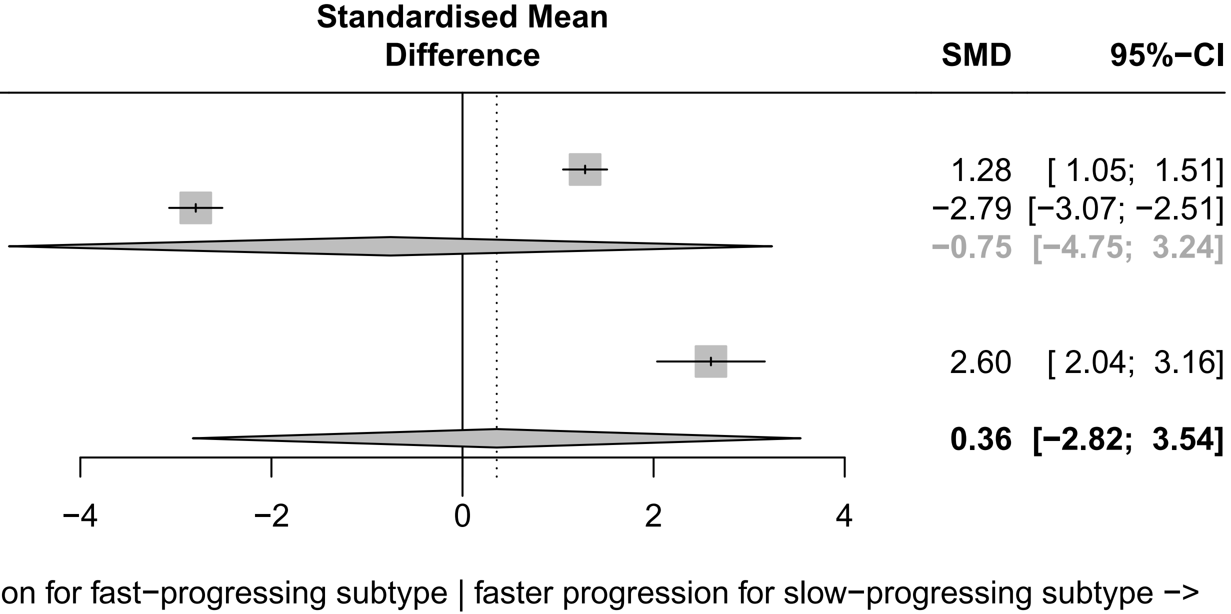

Three−level metaanalysis using random effects to calculate an overall SMD estimate for Smell across cohorts.
The dashed line indicates the overall mean SMD estimate. The solid line indicates no effect.

Forest plot for progression characteristics of symptom domain Motor symptoms (validation)

Study	Slow progressing Total	Slow progressing Mean	Slow progressing SD	Fast progressing Total	Fast progressing Mean	Fast progressing SD	SMD	95%−CI
**Cohort = LuxPARK (validation)**								
PDQ39 ADL	462	0.17	0.0828	98	0.40	0.2464	−1.85	[−2.09; −1.60]
Pegboard	429	0.06	0.0171	85	0.17	0.0578	−3.85	[−4.18; −3.52]
UPDRS II	462	0.17	0.0671	99	0.49	0.2085	−2.99	[−3.27; −2.72]
UPDRS III on	429	0.10	0.0289	90	0.29	0.0919	−4.21	[−4.56; −3.87]
UPDRS IV	462	0.10	0.0806	99	0.09	0.0819	0.12	[−0.09; 0.34]
**Random effects model**	**2244**			**471**			**−2.55**	**[−4.09; −1.01]**
**Cohort = ICEBERG (validation)**								
UPDRS II	135	0.22	0.0408	19	0.65	0.1997	−5.47	[−6.26; −4.69]
UPDRS III off	134	0.07	0.0104	19	0.31	0.1850	−3.64	[−4.28; −3.01]
UPDRS III on	127	0.03	0.0038	19	0.12	0.0846	−2.94	[−3.54; −2.35]
UPDRS IV	135	0.09	0.1311	19	0.21	0.1229	−0.93	[−1.42; −0.44]
**Random effects model**	**531**			**76**			**−3.23**	**[−5.07; −1.39]**
**Random effects model**	**2775**			**547**			**−2.85**	**[−3.98; −1.72]**

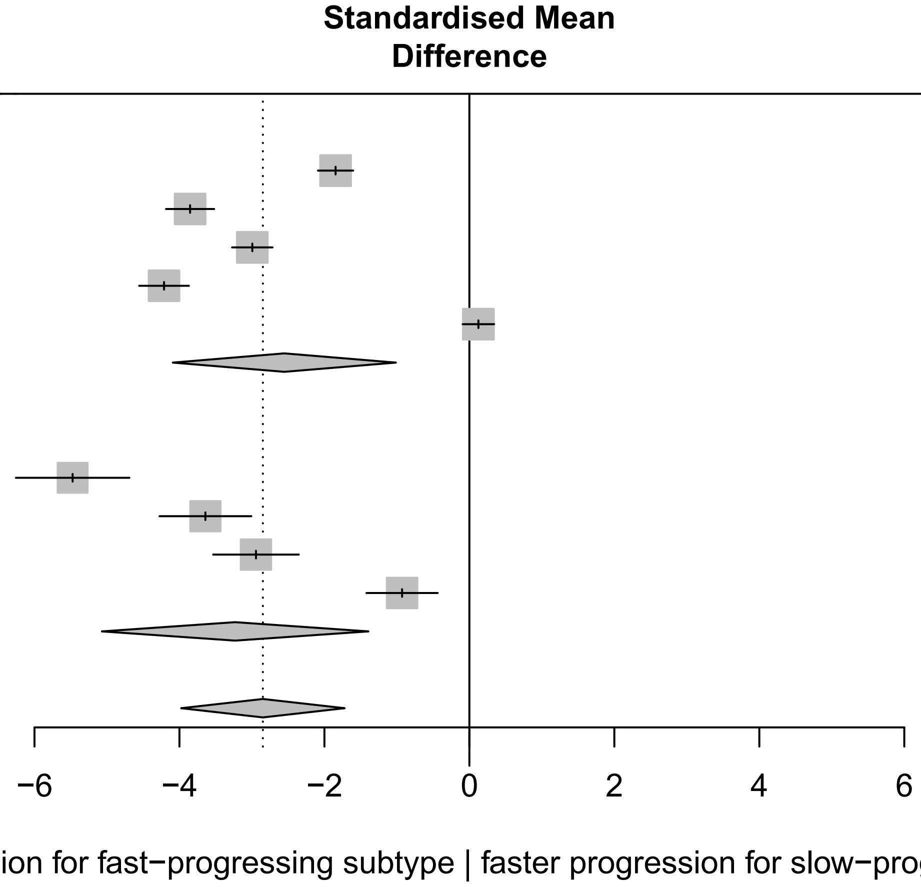

Three−level metaanalysis using random effects to calculate an overall SMD estimate for Motor symptoms across cohorts.
The dashed line indicates the overall mean SMD estimate. The solid line indicates no effect.

Forest plot for progression characteristics of symptom domain Impulsivity (validation)

Study	Slow progressing Total	Slow progressing Mean	Slow progressing SD	Fast progressing Total	Fast progressing Mean	Fast progressing SD	SMD	95%−CI
**Cohort = ICEBERG (validation)**								
QUIP−RS	135	0.06	0.0699	19	0.05	0.0637	0.16	[−0.32; 0.64]

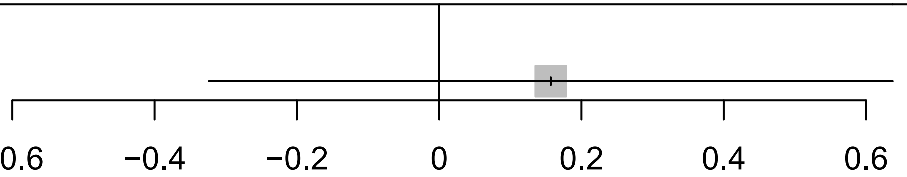

Three−level metaanalysis using random effects to calculate an overall SMD estimate for Impulsivity across cohorts. The dashed line indicates the overall mean SMD estimate. The solid line indicates no effect.

Forest plots for symptom domain baseline associations (in cohort)

Forest plot for baseline characteristics of symptom domain Axial & PIGD

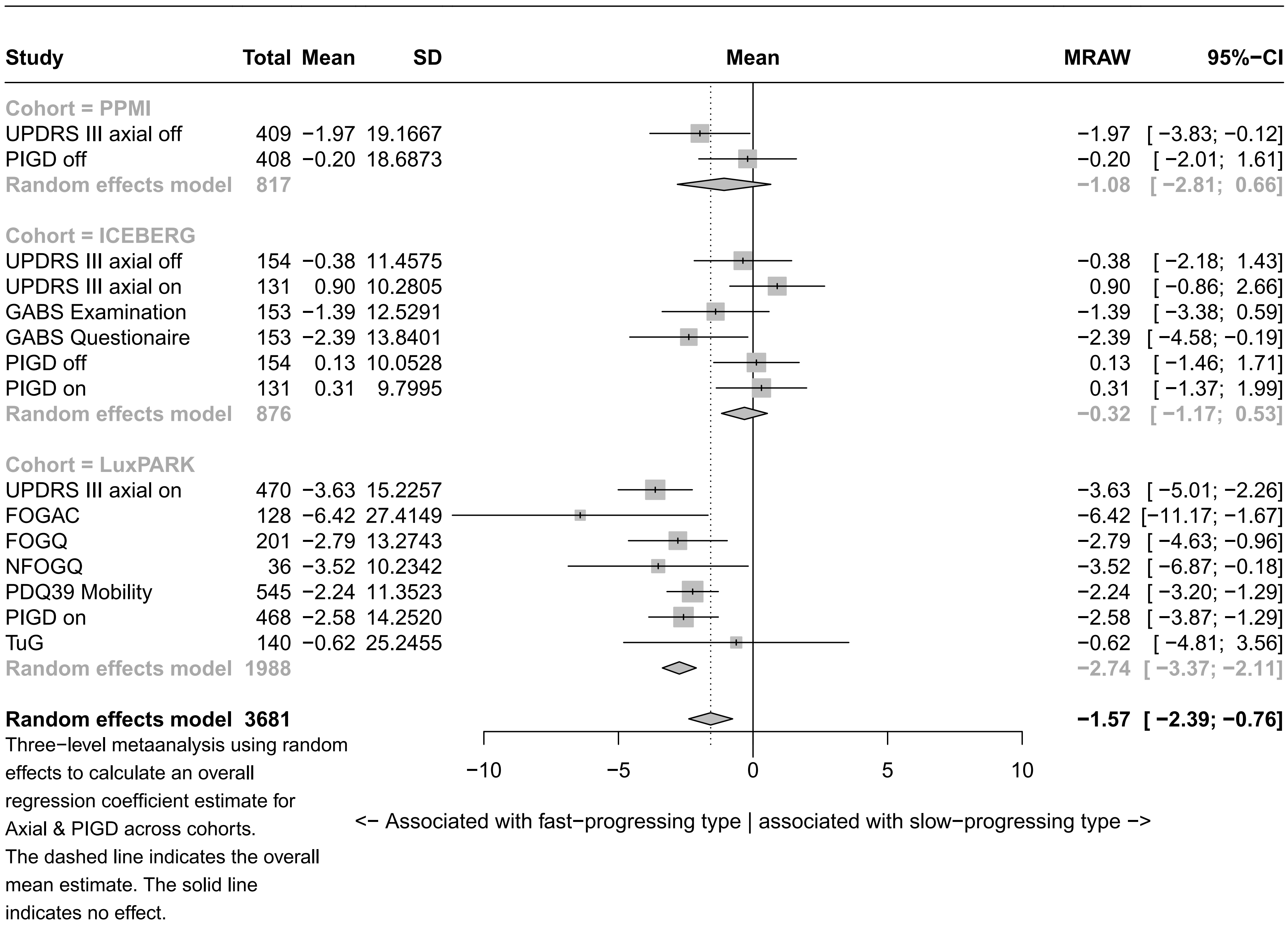

Forest plot for baseline characteristics of symptom domain Depression

Study	Total	Mean	SD	MRAW	95%−CI
**Cohort = LuxPARK**					
BDI	545	−1.35	17.0167	−1.35	[−2.78; 0.08]
NMSQ Depression	543	−0.20	5.9303	−0.20	[−0.70; 0.30]
PDQ39 Depression	548	−0.43	10.7187	−0.43	[−1.33; 0.46]
UPDRS I Depression	558	−0.66	9.7007	−0.66	[−1.47; 0.14]
**Random effects model**	**2194**			**−0.41**	**[−0.78; −0.04]**
**Cohort = PPMI**					
GDS	409	−0.67	14.7383	−0.67	[−2.10; 0.76]
UPDRS I Depression	409	−1.59	17.4597	−1.59	[−3.29; 0.10]
**Random effects model**	**818**			**−1.06**	**[−2.15; 0.04]**
**Cohort = ICEBERG**					
HADS depression	154	−0.11	11.4553	−0.11	[−1.92; 1.70]
NMSQ Depression	154	−0.32	6.0037	−0.32	[−1.27; 0.63]
UPDRS I Depression	154	−0.46	7.3865	−0.46	[−1.62; 0.71]
**Random effects model**	**462**			**−0.34**	**[−1.02; 0.35]**
**Random effects model**	**3474**			**−0.45**	**[−0.76; −0.14]**

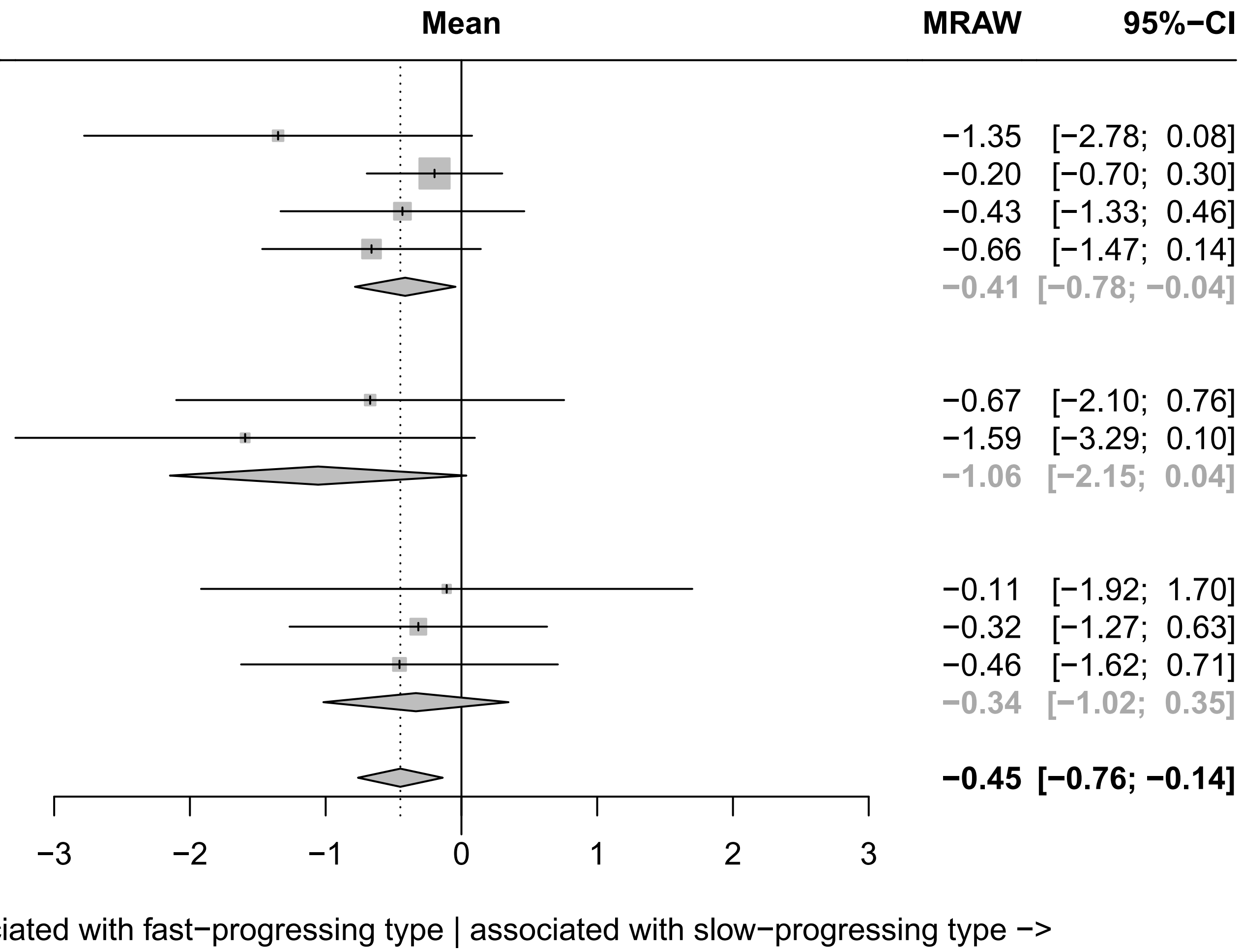

Three−level metaanalysis using random effects to calculate an overall regression coefficient estimate for Depression across cohorts.
The dashed line indicates the overall mean estimate. The solid line indicates no effect.

Forest plot for baseline characteristics of symptom domain Memory

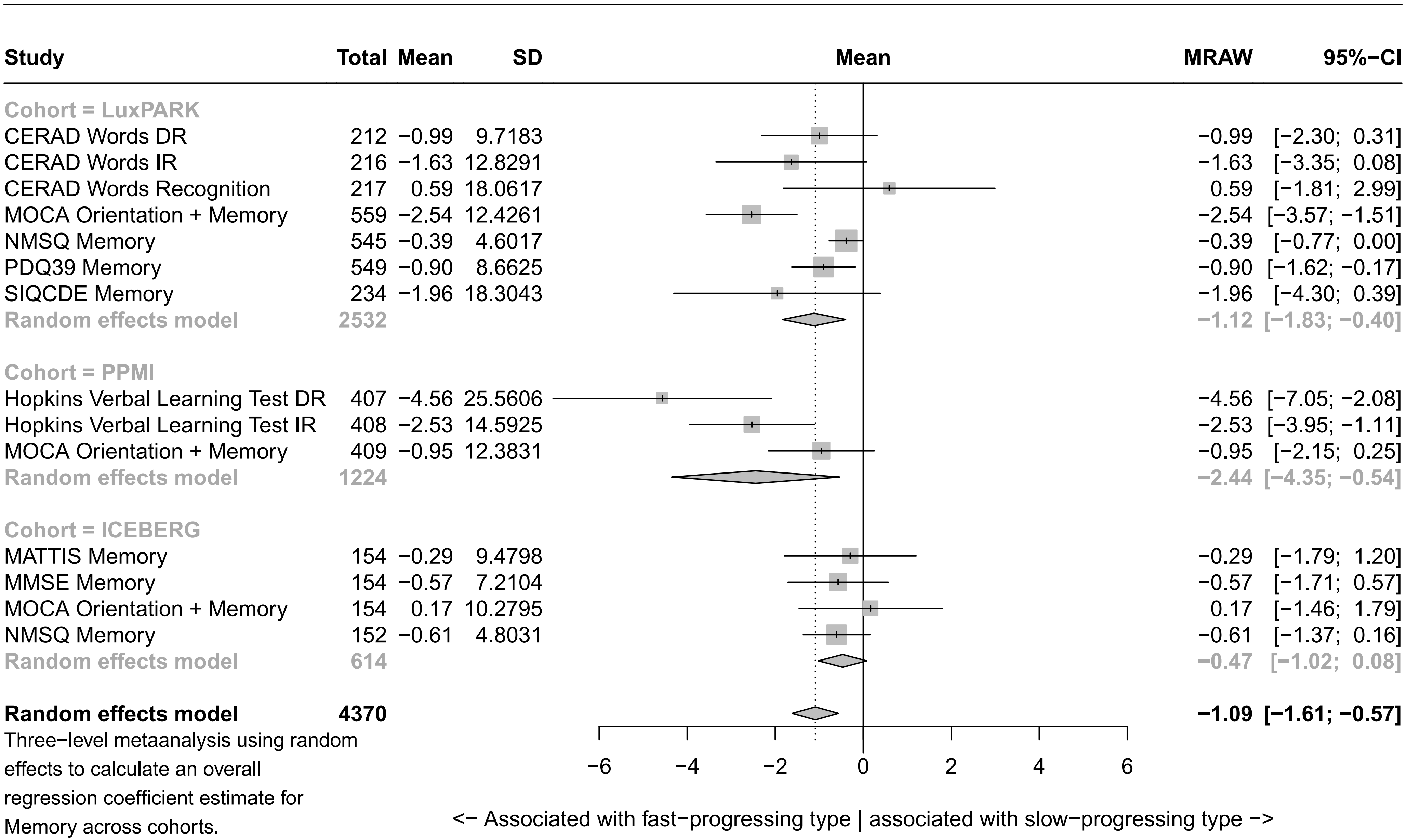

Three−level metaanalysis using random effects to calculate an overall regression coefficient estimate for Memory across cohorts.
The dashed line indicates the overall mean estimate. The solid line indicates no effect.

Forest plot for baseline characteristics of symptom domain Overall severity

Study	Total	Mean	SD	MRAW	95%−CI
**Cohort = ICEBERG**					
CGIS	154	−0.69	10.7670	−0.69	[−2.39; 1.01]
H&Y	154	−2.42	19.1051	−2.42	[−5.44; 0.60]
SEADL	154	−1.45	15.6540	−1.45	[−3.93; 1.02]
UPDRS I−III off	154	−2.13	15.3270	−2.13	[−4.55; 0.29]
UPDRS I−III on	131	−1.14	12.3432	−1.14	[−3.26; 0.97]
**Random effects model**	**747**			**−1.34**	**[−2.34; −0.35]**
**Cohort = LuxPARK**					
FAQ	230	−1.73	11.0542	−1.73	[−3.16; −0.30]
H&Y	560	−3.24	17.6985	−3.24	[−4.70; −1.77]
PDQ39	531	−2.52	13.7787	−2.52	[−3.69; −1.35]
UPDRS I−III on	461	−3.38	16.1434	−3.38	[−4.85; −1.91]
**Random effects model**	**1782**			**−2.68**	**[−3.36; −2.00]**
**Cohort = PPMI**					
H&Y	409	0.12	5.4184	0.12	[−0.40; 0.65]
SEADL	409	−0.78	14.0054	−0.78	[−2.14; 0.57]
UPDRS I−III off	408	−2.63	18.9616	−2.63	[−4.47; −0.80]
**Random effects model**	**1226**			**−0.90**	**[−2.41; 0.62]**
**Random effects model**	**3755**			**−1.75**	**[−2.50; −0.99]**

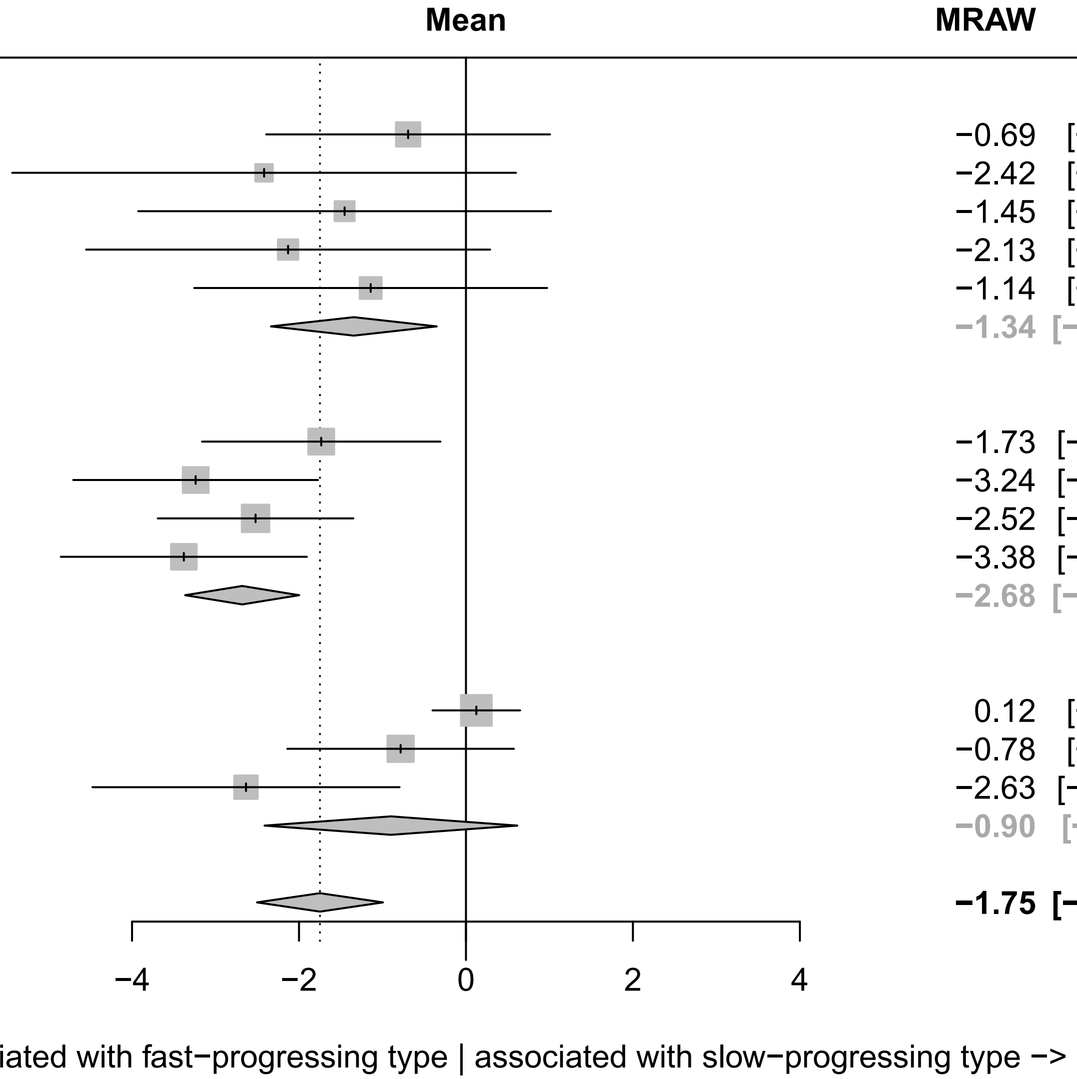

Three−level metaanalysis using random effects to calculate an overall regression coefficient estimate for Overall severity across cohorts. The dashed line indicates the overall mean estimate. The solid line indicates no effect.

Forest plot for baseline characteristics of symptom domain Apathy

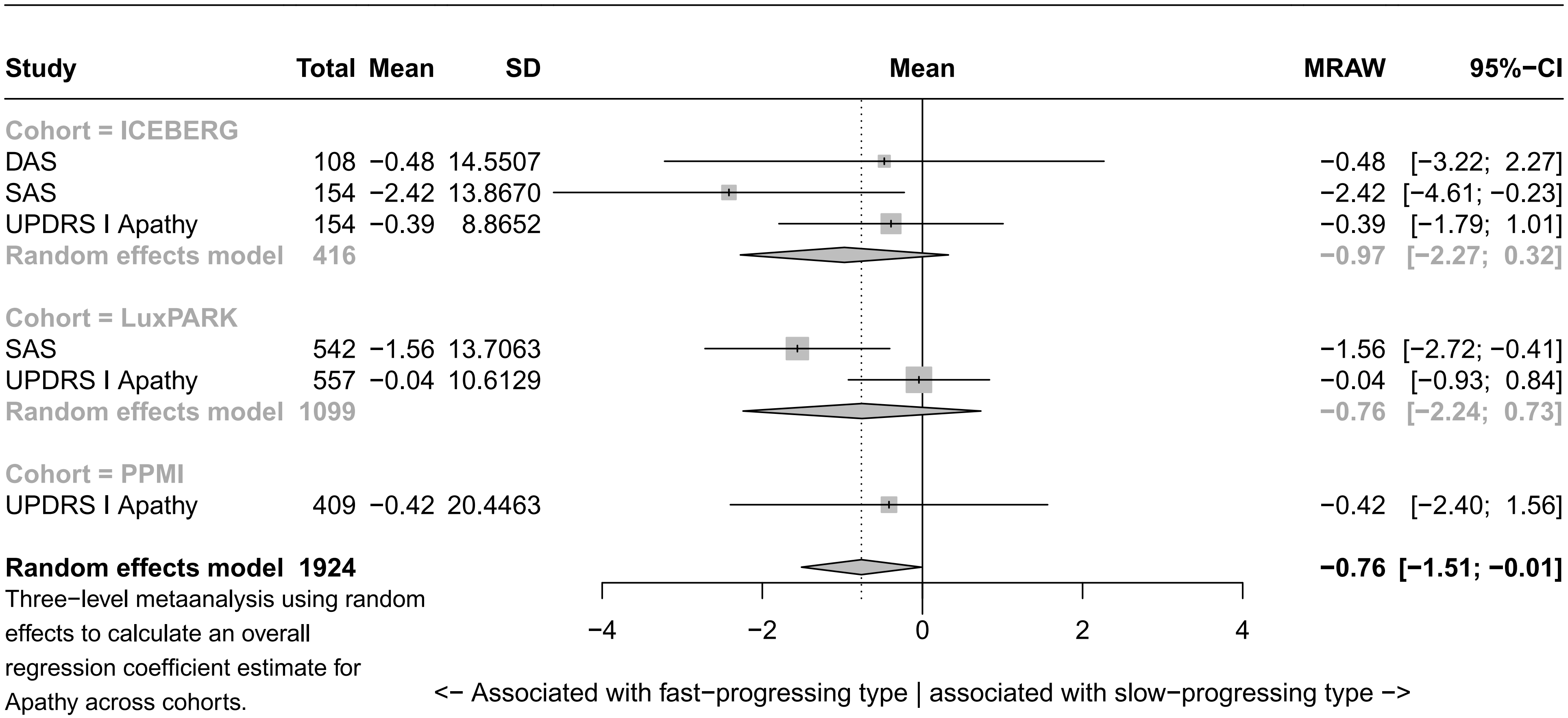

Three−level metaanalysis using random effects to calculate an overall regression coefficient estimate for Apathy across cohorts.
The dashed line indicates the overall mean estimate. The solid line indicates no effect.

Forest plot for baseline characteristics of symptom domain Sleep

Study	Total	Mean	SD	MRAW	95%−CI
**Cohort = PPMI**					
ESS	408	−1.38	12.9174	−1.38	[−2.63; −0.12]
UPDRS I Sleep	409	−1.26	13.3186	−1.26	[−2.55; 0.03]
**Random effects model**	**817**			**−1.32**	**[−2.22; −0.42]**
**Cohort = ICEBERG**					
ESS	143	−0.62	11.0335	−0.62	[−2.43; 1.19]
NMSQ Sleep	154	−0.28	7.4813	−0.28	[−1.46; 0.91]
UPDRS I Sleep	154	0.45	9.1040	0.45	[−0.99; 1.89]
**Random effects model**	**451**			**−0.11**	**[−0.93; 0.70]**
**Cohort = LuxPARK**					
NMSQ Sleep	545	0.03	6.7698	0.03	[−0.54; 0.60]
PDSS	541	−0.88	13.5002	−0.88	[−2.02; 0.26]
UPDRS I Sleep	558	−0.37	10.4775	−0.37	[−1.24; 0.50]
**Random effects model**	**1644**			**−0.23**	**[−0.71; 0.24]**
**Random effects model**	**2912**			**−0.43**	**[−0.86; −0.01]**

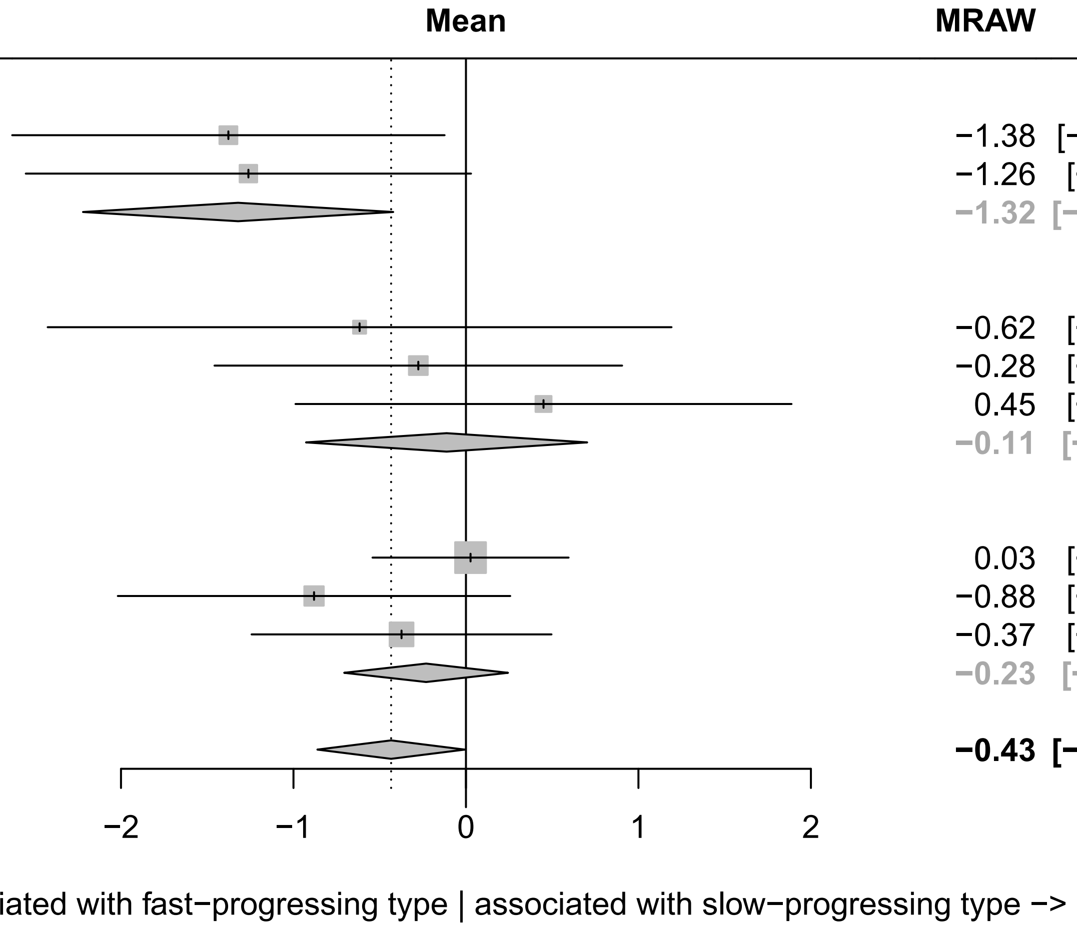

Three−level metaanalysis using random effects to calculate an overall regression coefficient estimate for Sleep across cohorts.
The dashed line indicates the overall mean estimate. The solid line indicates no effect.

Forest plot for baseline characteristics of symptom domain Overall cognition

Study	Total	Mean	SD	MRAW	95%−CI
**Cohort = LuxPARK**					
FAB	235	−3.29	10.8757	−3.29	[−4.68; −1.90]
MoCA	559	−3.97	13.9238	−3.97	[−5.13; −2.82]
NMSQ Cognition	544	−0.59	5.5913	−0.59	[−1.06; −0.12]
PDQ39 Cognition	549	−1.23	9.8271	−1.23	[−2.05; −0.41]
SIQCDE	232	−1.85	18.8396	−1.85	[−4.27; 0.58]
UPDRS I Cognition	558	−1.92	10.6148	−1.92	[−2.80; −1.04]
**Random effects model**	**2677**			**−2.08**	**[−3.17; −0.99]**
**Cohort = ICEBERG**					
FAB	154	−1.38	9.2690	−1.38	[−2.85; 0.08]
MATTIS	154	−1.74	11.4295	−1.74	[−3.54; 0.07]
MMSE	154	−1.07	6.5563	−1.07	[−2.10; −0.03]
MoCA	154	−1.19	10.1073	−1.19	[−2.79; 0.40]
NMSQ Cognition	154	−0.85	6.1851	−0.85	[−1.82; 0.13]
UPDRS I Cognition	154	−1.20	10.4489	−1.20	[−2.85; 0.45]
**Random effects model**	**924**			**−1.13**	**[−1.66; −0.60]**
**Cohort = PPMI**					
MoCA	409	−0.59	14.1844	−0.59	[−1.97; 0.78]
UPDRS I Cognition	409	−1.27	9.1717	−1.27	[−2.16; −0.38]
**Random effects model**	**818**			**−1.07**	**[−1.82; −0.33]**
**Random effects model**	**4419**			**−1.53**	**[−2.07; −1.00]**

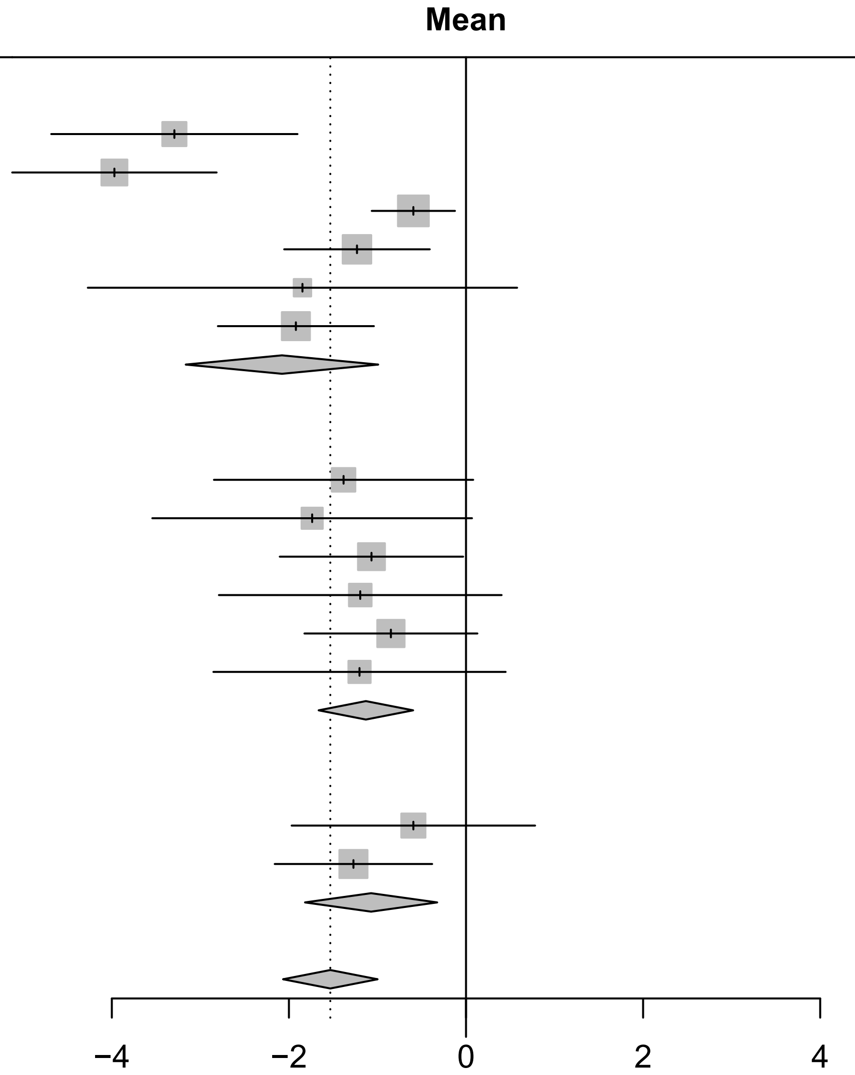

<− Associated with fast−progressing type | associated with slow−progressing type −>

Three−level metaanalysis using random effects to calculate an overall regression coefficient estimate for Overall cognition across cohorts. The dashed line indicates the overall mean estimate. The solid line indicates no effect.

Forest plot for baseline characteristics of symptom domain Conceptualization

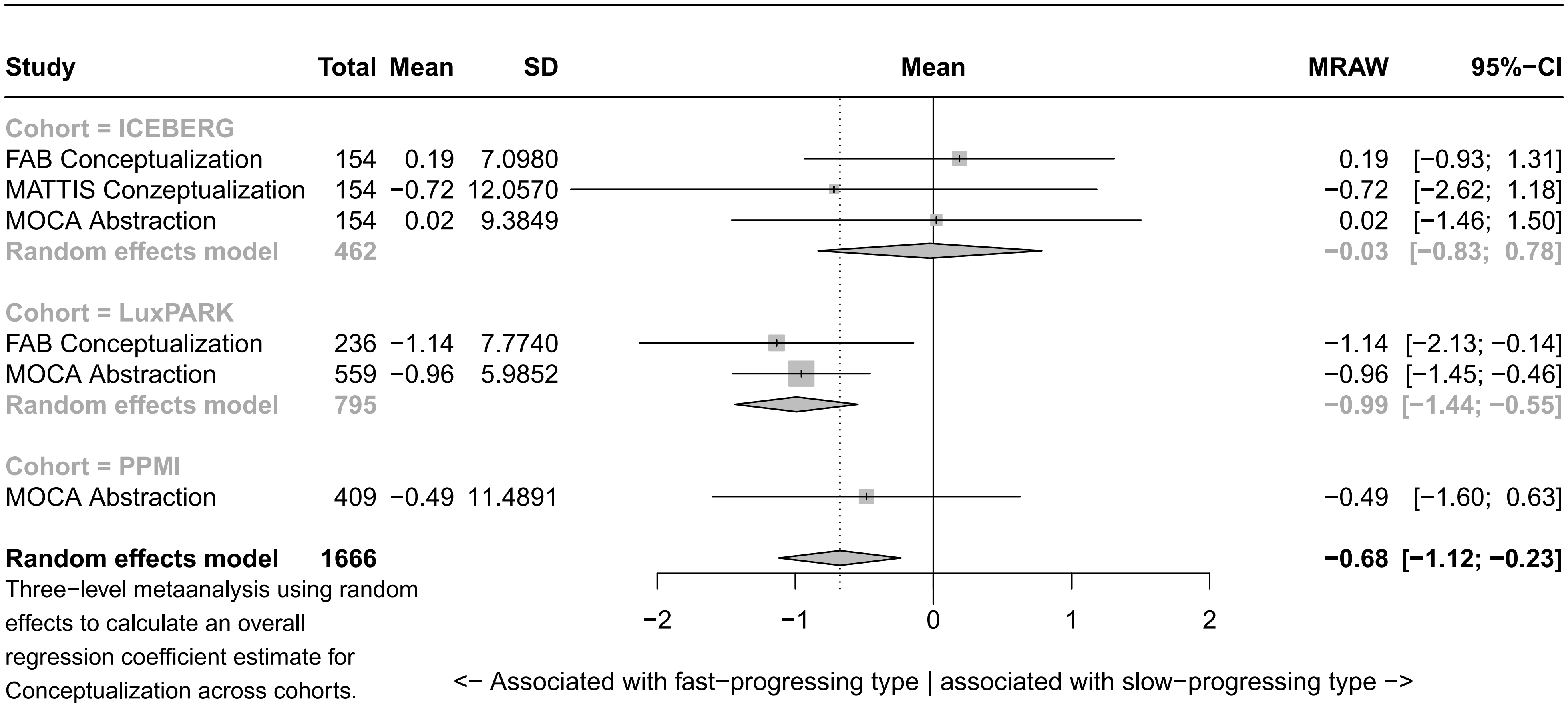

Three−level metaanalysis using random effects to calculate an overall regression coefficient estimate for Conceptualization across cohorts. The dashed line indicates the overall mean estimate. The solid line indicates no effect.

Forest plot for baseline characteristics of symptom domain Visuo−executive

Study	Total	Mean	SD	MRAW	95%−CI
**Cohort = ICEBERG**					
FAB 3−6	154	−1.18	9.2951	−1.18	[−2.65; 0.29]
MATTIS Initiation + Construction	154	−1.16	11.8424	−1.16	[−3.03; 0.71]
MMSE Construction	154	−2.06	15.8255	−2.06	[−4.56; 0.44]
MOCA Visuspatial/Executive	154	−1.15	7.7696	−1.15	[−2.38; 0.08]
**Random effects model**	**616**			**−1.25**	**[−2.05; −0.46]**
**Cohort = LuxPARK**					
FAB 3−6	235	−3.08	10.8871	−3.08	[−4.47; −1.69]
Judgement Line Orientation	214	−2.53	11.8539	−2.53	[−4.12; −0.94]
MOCA Visuspatial/Executive	560	−2.36	9.4592	−2.36	[−3.15; −1.58]
Stroop Errors	208	−7.66	45.0379	−7.66	[−13.78; −1.54]
Stroop Time	208	−3.35	15.9332	−3.35	[−5.51; −1.18]
Trailmaking A	549	−6.75	26.6864	−6.75	[−8.98; −4.51]
Trailmaking B	480	−5.51	18.8538	−5.51	[−7.20; −3.83]
**Random effects model**	**2454**			**−3.94**	**[−5.30; −2.59]**
**Cohort = PPMI**					
Judgement Line Orientation	408	−1.19	11.5763	−1.19	[−2.31; −0.07]
MOCA Visuspatial/Executive	409	−0.50	12.3462	−0.50	[−1.69; 0.70]
Symbol Digit Modalities	408	−5.02	21.9826	−5.02	[−7.15; −2.89]
**Random effects model**	**1225**			**−2.10**	**[−4.71; 0.52]**
**Random effects model**	**4295**			**−2.75**	**[−3.77; −1.74]**

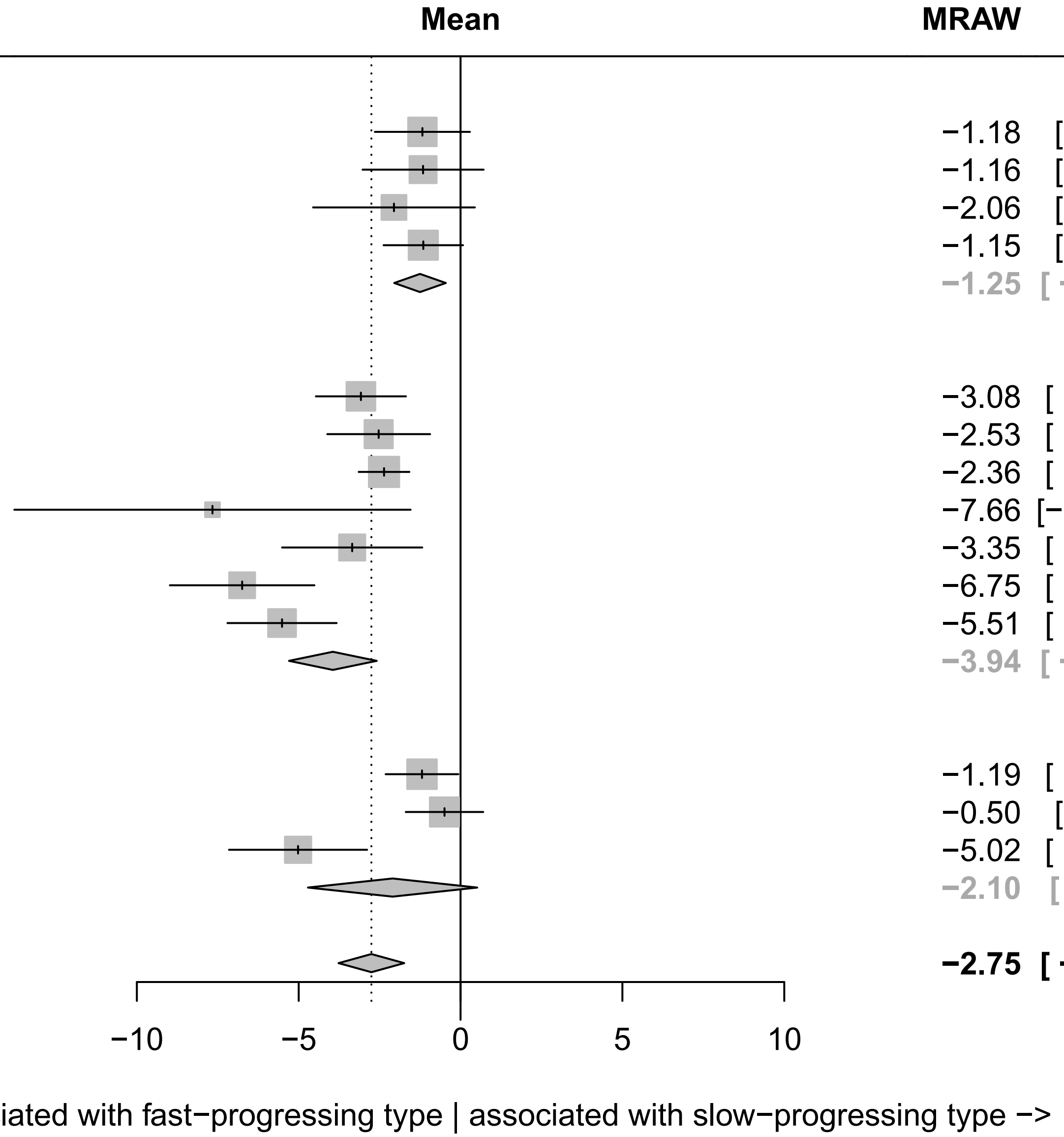

Three−level metaanalysis using random effects to calculate an overall regression coefficient estimate for Visuo−executive across cohorts. The dashed line indicates the overall mean estimate. The solid line indicates no effect.

Forest plot for baseline characteristics of symptom domain Language

Study	Total	Mean	SD	MRAW	95%−CI
**Cohort = ICEBERG**					
FAB VFT	154	−1.18	8.0533	−1.18	[−2.45; 0.09]
MMSE Language	154	0.02	9.3212	0.02	[−1.46; 1.49]
MOCA Language + Naming	154	−0.82	9.2009	−0.82	[−2.28; 0.63]
VFT phonematic F	151	−1.97	11.6530	−1.97	[−3.83; −0.11]
VFT phonematic S	143	−1.10	11.5611	−1.10	[−2.99; 0.80]
VFT semantic supermarket	151	−2.15	14.1365	−2.15	[−4.41; 0.10]
**Random effects model**	**907**			**−1.04**	**[−1.70; −0.38]**
**Cohort = LuxPARK**					
FAB VFT	236	−1.82	8.6299	−1.82	[−2.92; −0.72]
MOCA Language + Naming	559	−2.99	14.1846	−2.99	[−4.16; −1.81]
VFT phonematic F	548	−2.90	14.7073	−2.90	[−4.13; −1.66]
VFT phonematic S	221	−4.26	16.7491	−4.26	[−6.47; −2.05]
VFT semantic sum	210	−3.86	14.1298	−3.86	[−5.77; −1.94]
**Random effects model**	**1774**			**−2.89**	**[−3.67; −2.11]**
**Cohort = PPMI**					
MOCA Language + Naming	409	−0.96	16.1720	−0.96	[−2.53; 0.60]
VFT semantic sum	408	−5.86	23.6273	−5.86	[−8.15; −3.57]
**Random effects model**	**817**			**−3.34**	**[−8.13; 1.46]**
**Random effects model**	**3498**			**−2.18**	**[−2.98; −1.37]**

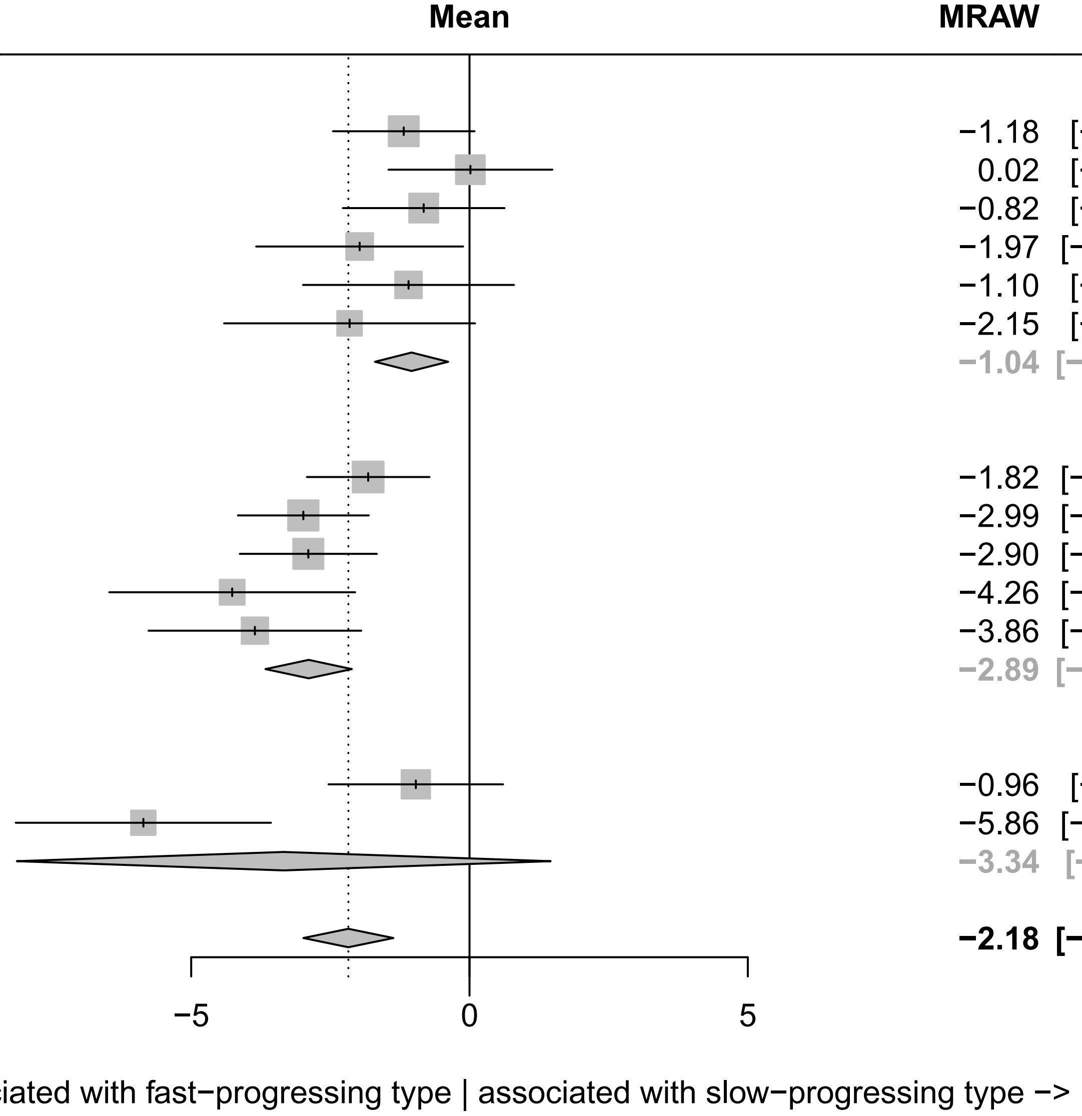

Three−level metaanalysis using random effects to calculate an overall regression coefficient estimate for Language across cohorts.
The dashed line indicates the overall mean estimate. The solid line indicates no effect.

Forest plot for baseline characteristics of symptom domain Anxiety

Study	Total	Mean	SD	MRAW	95%−CI
**Cohort = ICEBERG**					
HADS anxiety	154	1.37	14.0575	1.37	[−0.85; 3.59]
NMSQ Anxiety	152	−1.03	4.7079	−1.03	[−1.78; −0.28]
UPDRS I Anxiety	154	−0.94	9.1543	−0.94	[−2.38; 0.51]
**Random effects model**	**460**			**−0.58**	**[−1.65; 0.49]**
**Cohort = LuxPARK**					
NMSQ Anxiety	543	0.09	5.1173	0.09	[−0.34; 0.52]
PDQ39 Anxiety	550	−0.79	8.5075	−0.79	[−1.50; −0.07]
UPDRS I Anxiety	558	−0.48	10.2466	−0.48	[−1.33; 0.37]
**Random effects model**	**1651**			**−0.32**	**[−0.89; 0.25]**
**Cohort = PPMI**					
STA	405	−0.62	13.8380	−0.62	[−1.97; 0.73]
UPDRS I Anxiety	409	0.10	16.6865	0.10	[−1.52; 1.71]
**Random effects model**	**814**			**−0.33**	**[−1.36; 0.71]**
**Random effects model**	**2925**			**−0.43**	**[−0.85; −0.00]**

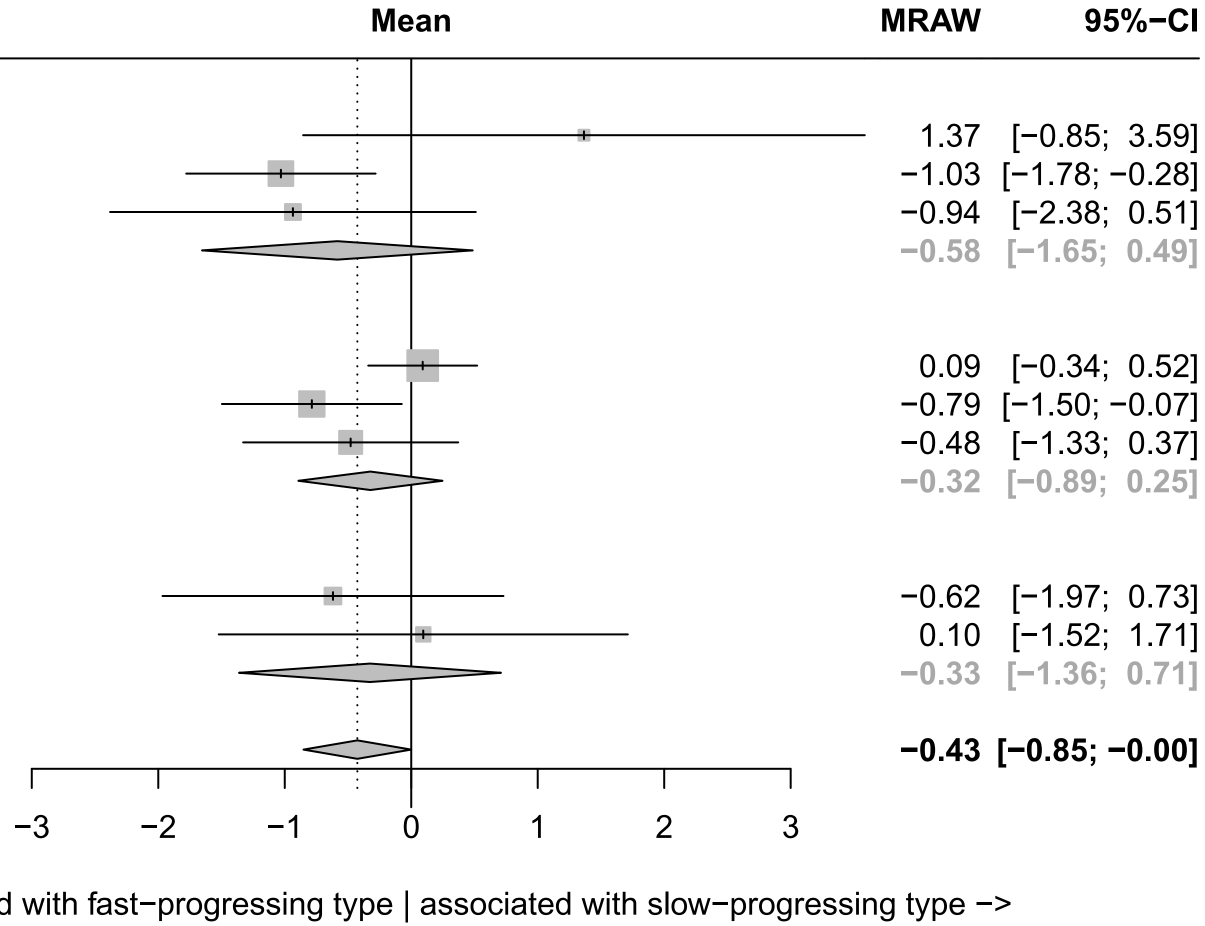

Three−level metaanalysis using random effects to calculate an overall regression coefficient estimate for Anxiety across cohorts.
The dashed line indicates the overall mean estimate. The solid line indicates no effect.

Forest plot for baseline characteristics of symptom domain Attention

Study	Total	Mean	SD	MRAW	95%−CI
**Cohort = ICEBERG**					
MATTIS Attention	154	−2.95	12.5929	−2.95	[−4.94; −0.96]
MMSE Attention	154	−1.53	9.9091	−1.53	[−3.10; 0.03]
MOCA Attention	154	−1.42	8.3050	−1.42	[−2.73; −0.11]
NMSQ Attention	152	−0.59	5.0818	−0.59	[−1.40; 0.22]
**Random effects model**	**614**			**−1.34**	**[−2.22; −0.46]**
**Cohort = LuxPARK**					
MOCA Attention	559	−2.33	13.2315	−2.33	[−3.42; −1.23]
NMSQ Attention	545	−0.44	4.7993	−0.44	[−0.85; −0.04]
PDQ39 Attention	549	−1.03	8.7061	−1.03	[−1.76; −0.30]
SIQCDE Attention	233	0.11	13.0786	0.11	[−1.57; 1.79]
Digit Span	214	−3.67	13.8011	−3.67	[−5.52; −1.82]
**Random effects model**	**2100**			**−1.37**	**[−2.54; −0.20]**
**Cohort = PPMI**					
MOCA Attention	409	−0.08	20.8489	−0.08	[−2.10; 1.94]
**Random effects model**	**3123**			**−1.27**	**[−1.93; −0.62]**

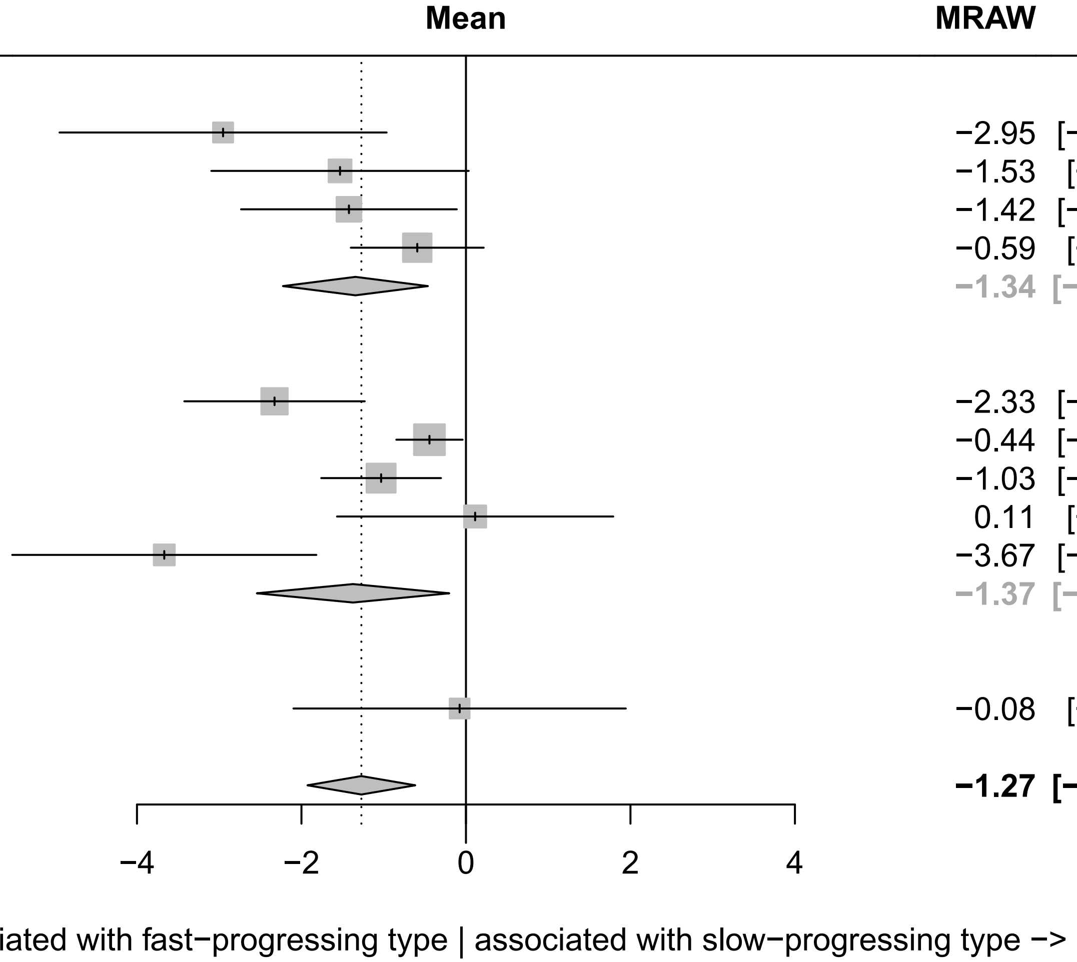

Three−level metaanalysis using random effects to calculate an overall regression coefficient estimate for Attention across cohorts.
The dashed line indicates the overall mean estimate. The solid line indicates no effect.

Forest plot for baseline characteristics of symptom domain Non motor symptoms

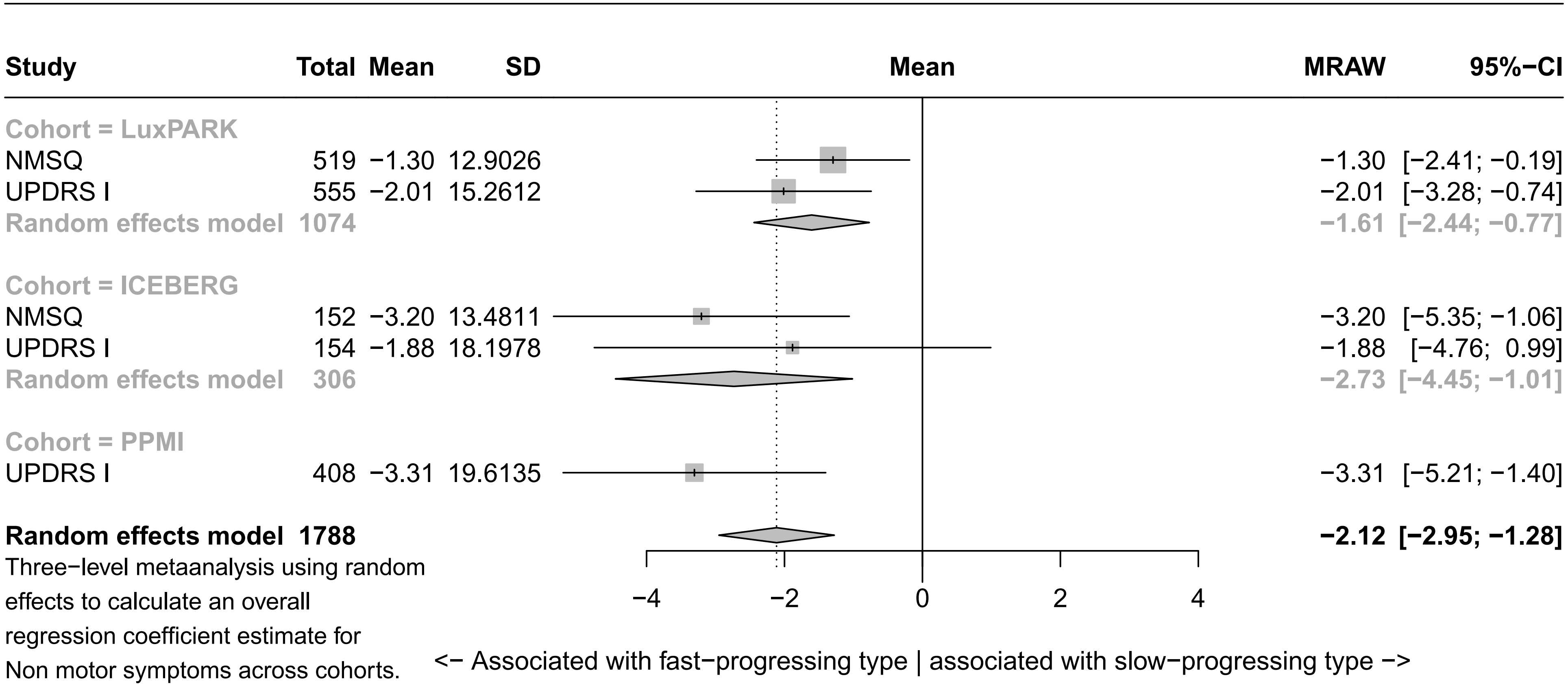

Three−level metaanalysis using random effects to calculate an overall regression coefficient estimate for Non motor symptoms across cohorts. The dashed line indicates the overall mean estimate. The solid line indicates no effect.

Forest plot for baseline characteristics of symptom domain Autonomic

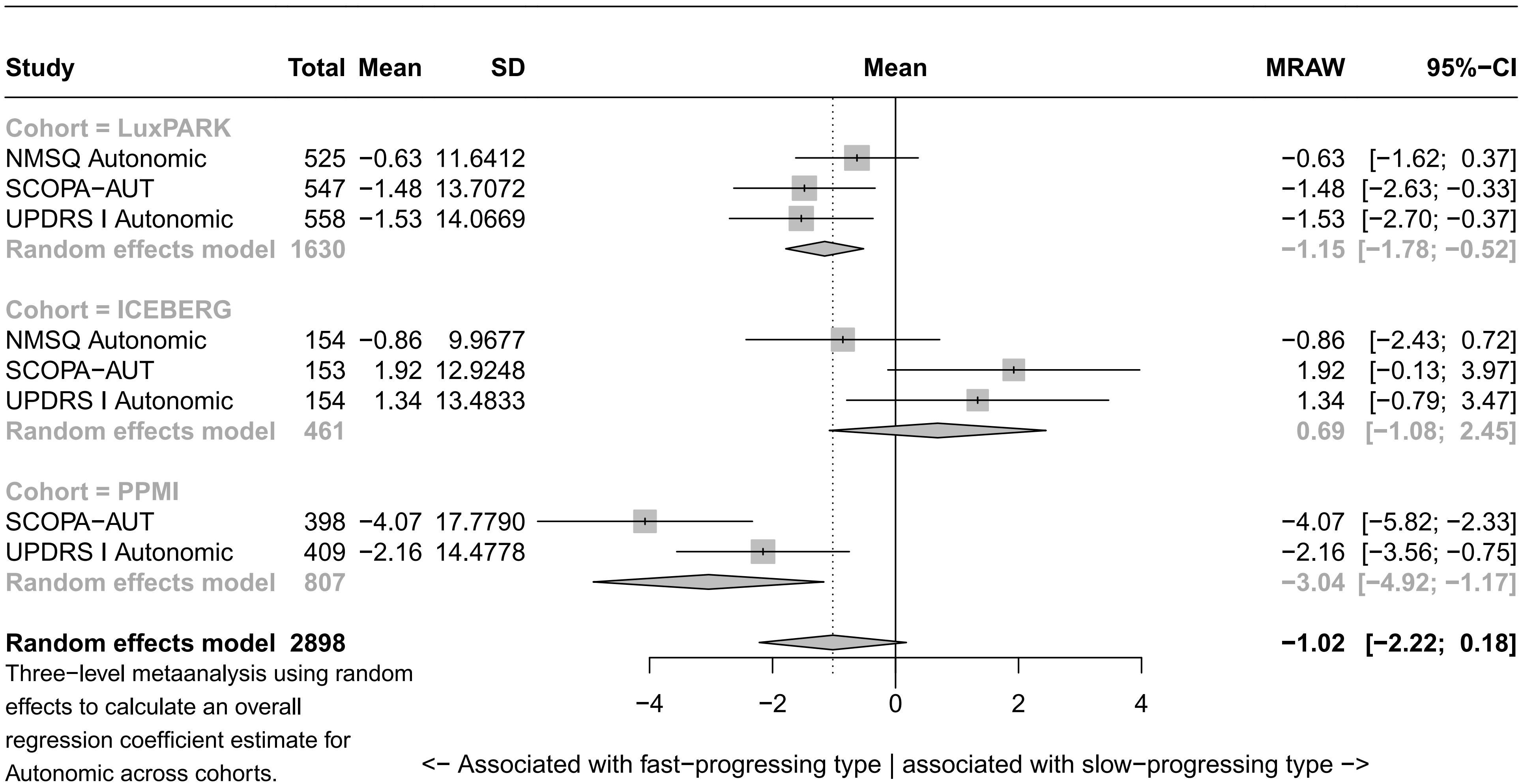

Three−level metaanalysis using random effects to calculate an overall regression coefficient estimate for Autonomic across cohorts. The dashed line indicates the overall mean estimate. The solid line indicates no effect.

Forest plot for baseline characteristics of symptom domain Hallucinations

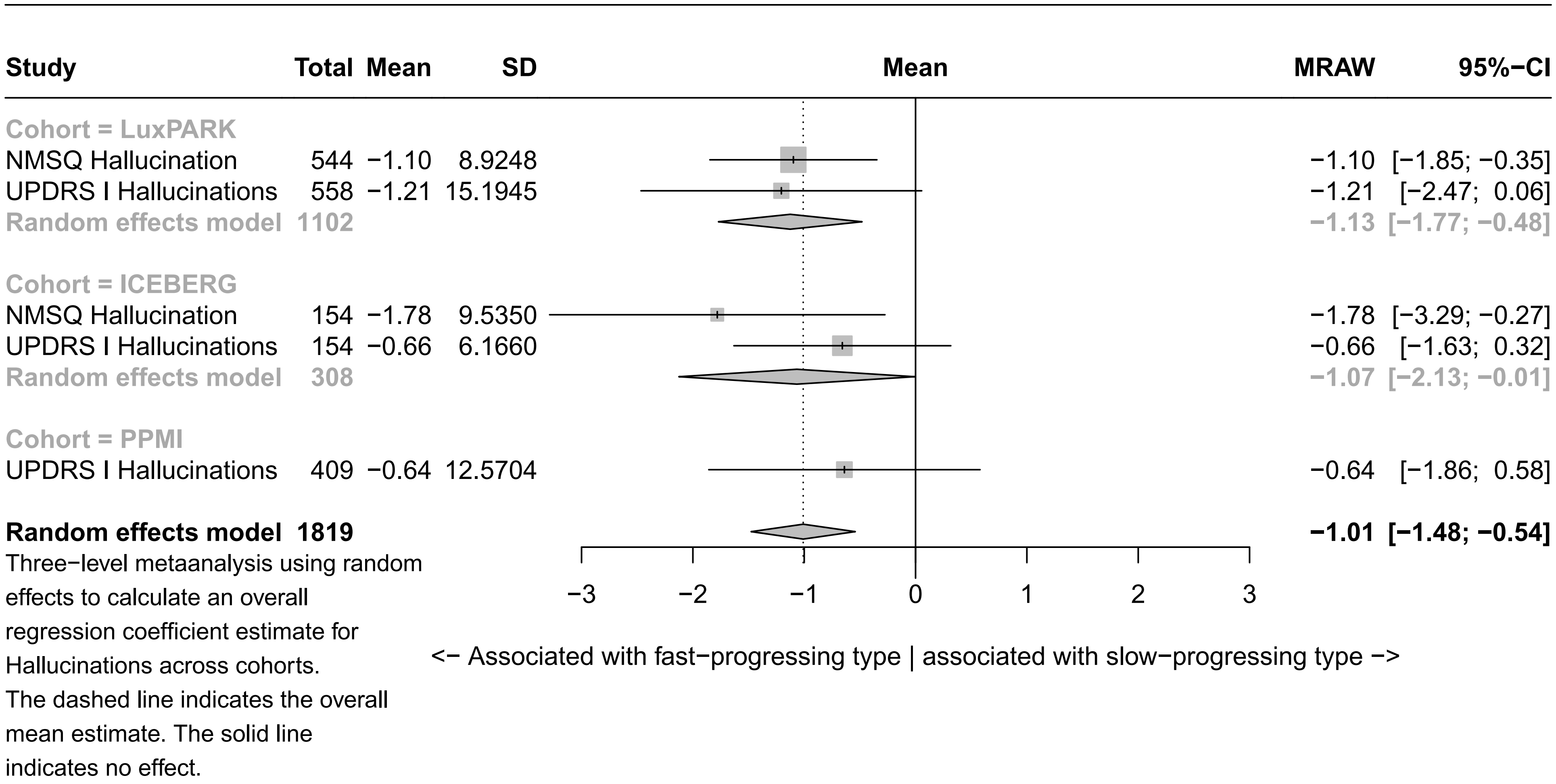

Forest plot for baseline characteristics of symptom domain Pain

Study	Total	Mean	SD	MRAW	95%−CI
**Cohort = LuxPARK**					
NMSQ Pain	546	0.09	5.3917	0.09	[−0.36; 0.54]
PDQ39 Pain	547	−0.18	9.1475	−0.18	[−0.95; 0.59]
UPDRS I Pain	558	−0.46	8.5352	−0.46	[−1.17; 0.25]
**Random effects model**	**1651**			**−0.10**	**[−0.45; 0.26]**
**Cohort = ICEBERG**					
NMSQ Pain	152	−0.59	4.6244	−0.59	[−1.32; 0.15]
UPDRS I Pain	154	−2.27	10.9099	−2.27	[−3.99; −0.55]
**Random effects model**	**306**			**−1.24**	**[−2.85; 0.36]**
**Cohort = PPMI**					
UPDRS I Pain	408	−0.43	12.3475	−0.43	[−1.63; 0.77]
**Random effects model**	**2365**			**−0.34**	**[−0.72; 0.04]**

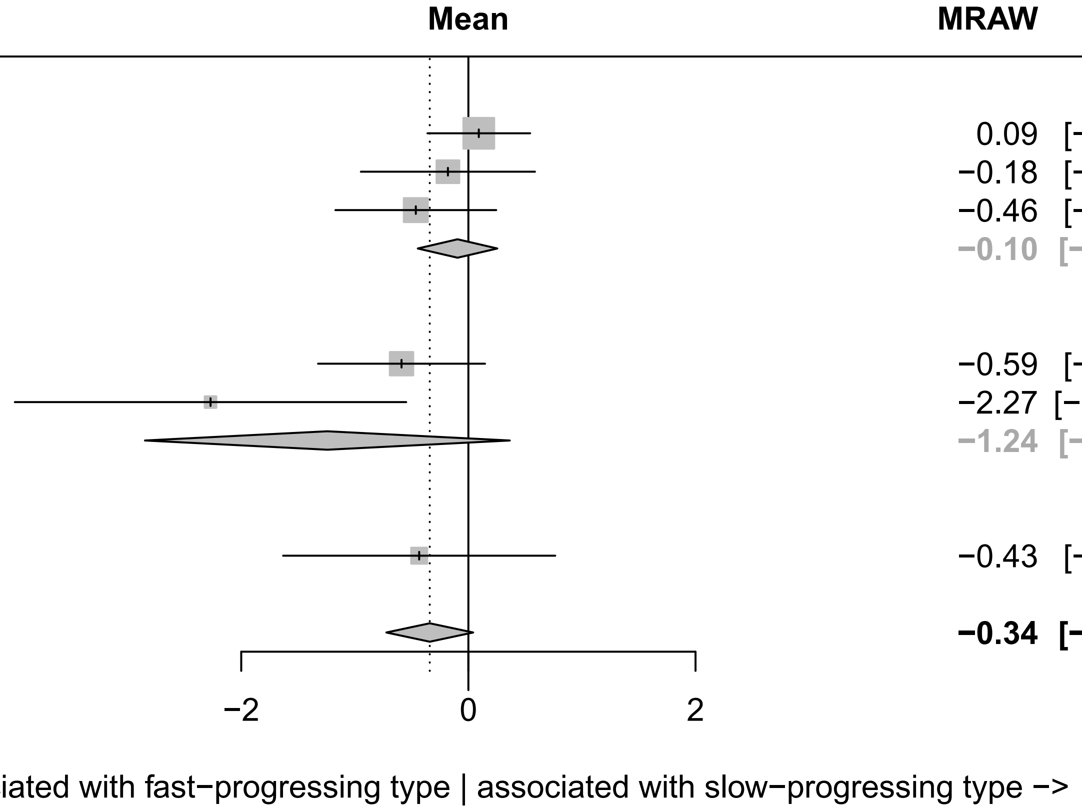

Three−level metaanalysis using random effects to calculate an overall regression coefficient estimate for Pain across cohorts.
The dashed line indicates the overall mean estimate. The solid line indicates no effect.

Forest plot for baseline characteristics of symptom domain RBD

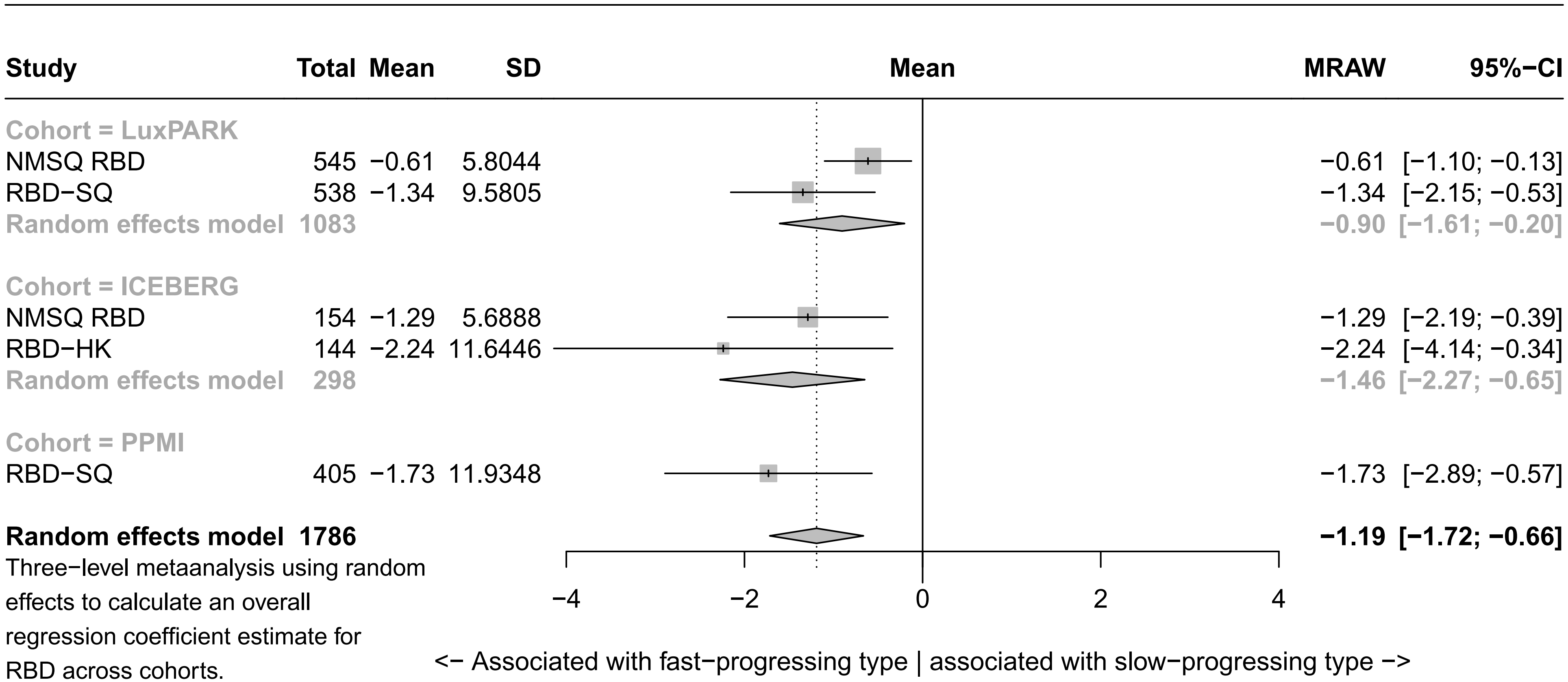

Three−level metaanalysis using random effects to calculate an overall regression coefficient estimate for RBD across cohorts.
The dashed line indicates the overall mean estimate. The solid line indicates no effect.

Forest plot for baseline characteristics of symptom domain Smell

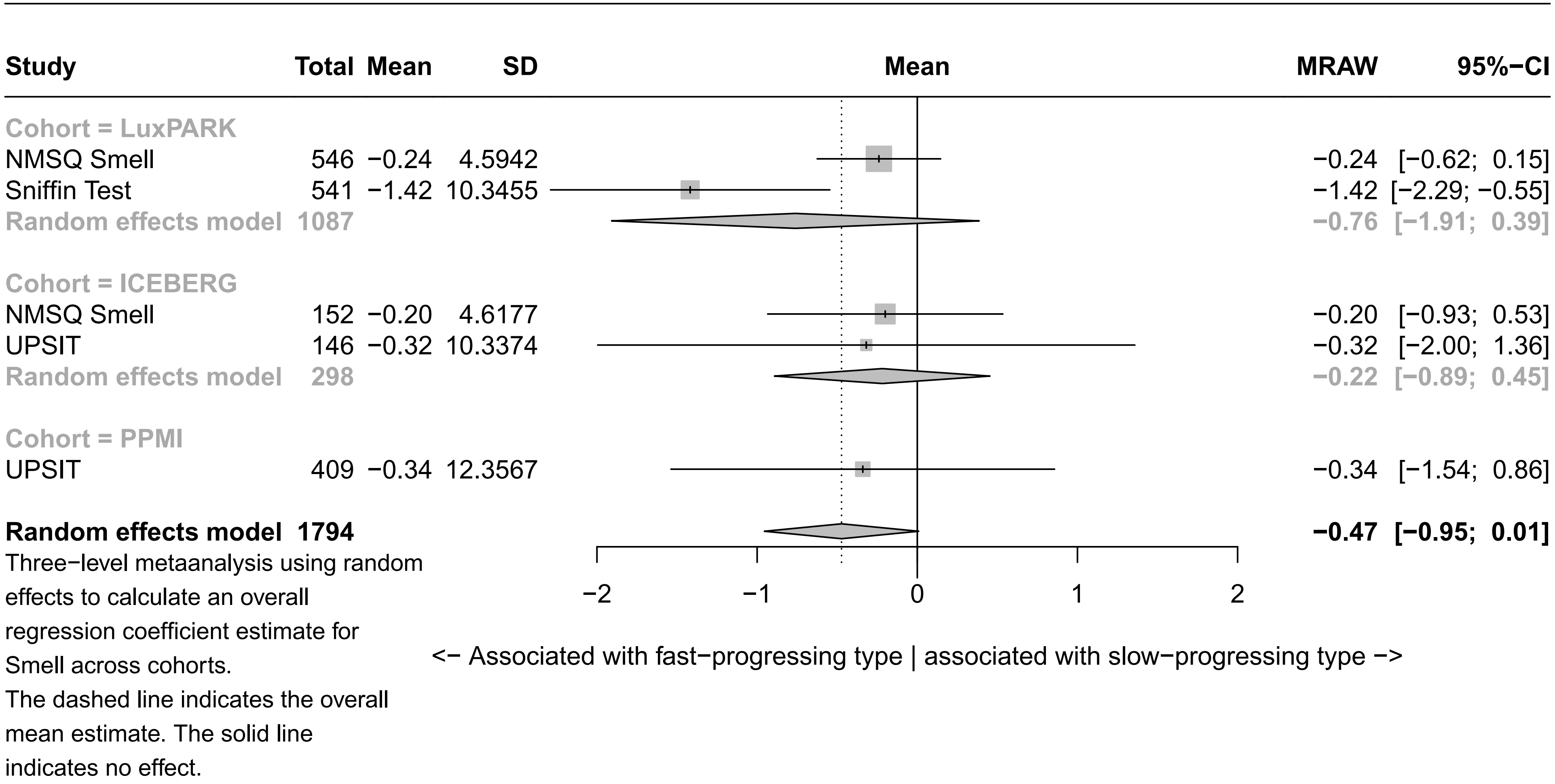

Forest plot for baseline characteristics of symptom domain Motor symptoms

Study	Total	Mean	SD	MRAW	95%−CI
**Cohort = LuxPARK**					
PDQ39 ADL	548	−2.58	11.3106	−2.58	[−3.53; −1.64]
Pegboard	539	−4.01	15.8950	−4.01	[−5.35; −2.67]
UPDRS II	555	−3.24	15.0570	−3.24	[−4.49; −1.99]
UPDRS III on	468	−2.10	14.4076	−2.10	[−3.41; −0.80]
UPDRS IV	556	−0.16	13.0989	−0.16	[−1.25; 0.93]
**Random effects model**	**2666**			**−2.39**	**[−3.67; −1.12]**
**Cohort = ICEBERG**					
UPDRS II	154	−0.16	12.3766	−0.16	[−2.11; 1.80]
UPDRS III off	154	−1.99	15.9456	−1.99	[−4.51; 0.52]
UPDRS III on	131	−0.50	9.9119	−0.50	[−2.19; 1.20]
UPDRS IV	154	−0.67	24.0333	−0.67	[−4.47; 3.12]
**Random effects model**	**593**			**−0.69**	**[−1.78; 0.41]**
**Cohort = PPMI**					
UPDRS II	408	−3.62	18.4277	−3.62	[−5.40; −1.83]
UPDRS III off	409	−0.12	15.3718	−0.12	[−1.61; 1.37]
UPDRS III untreated	409	−0.12	15.3718	−0.12	[−1.61; 1.37]
**Random effects model**	**1226**			**−1.23**	**[−3.47; 1.00]**
**Random effects model**	**4485**			**−1.67**	**[−2.57; −0.78]**

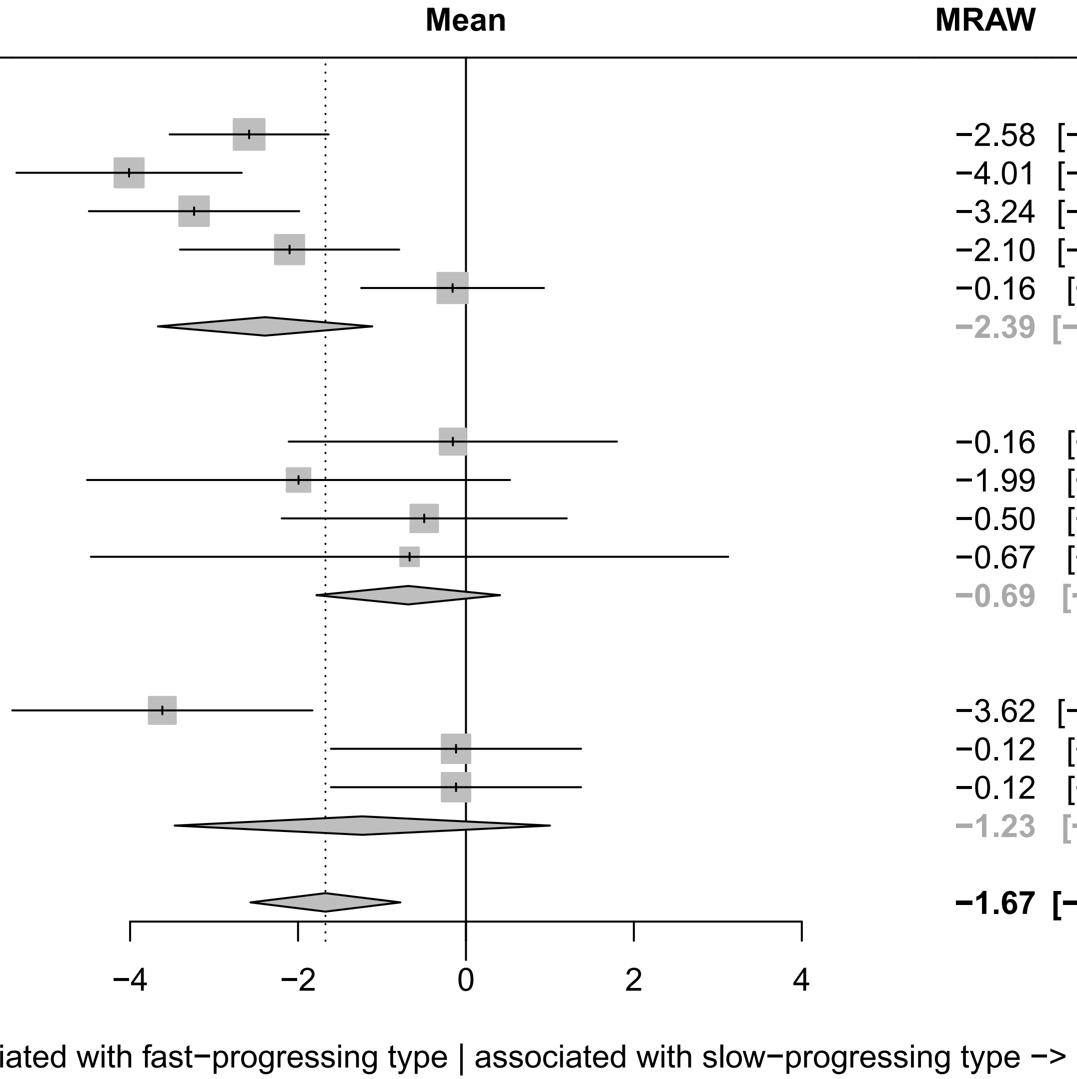

Three−level metaanalysis using random effects to calculate an overall regression coefficient estimate for Motor symptoms across cohorts. The dashed line indicates the overall mean estimate. The solid line indicates no effect.

Forest plot for baseline characteristics of symptom domain Impulsivity

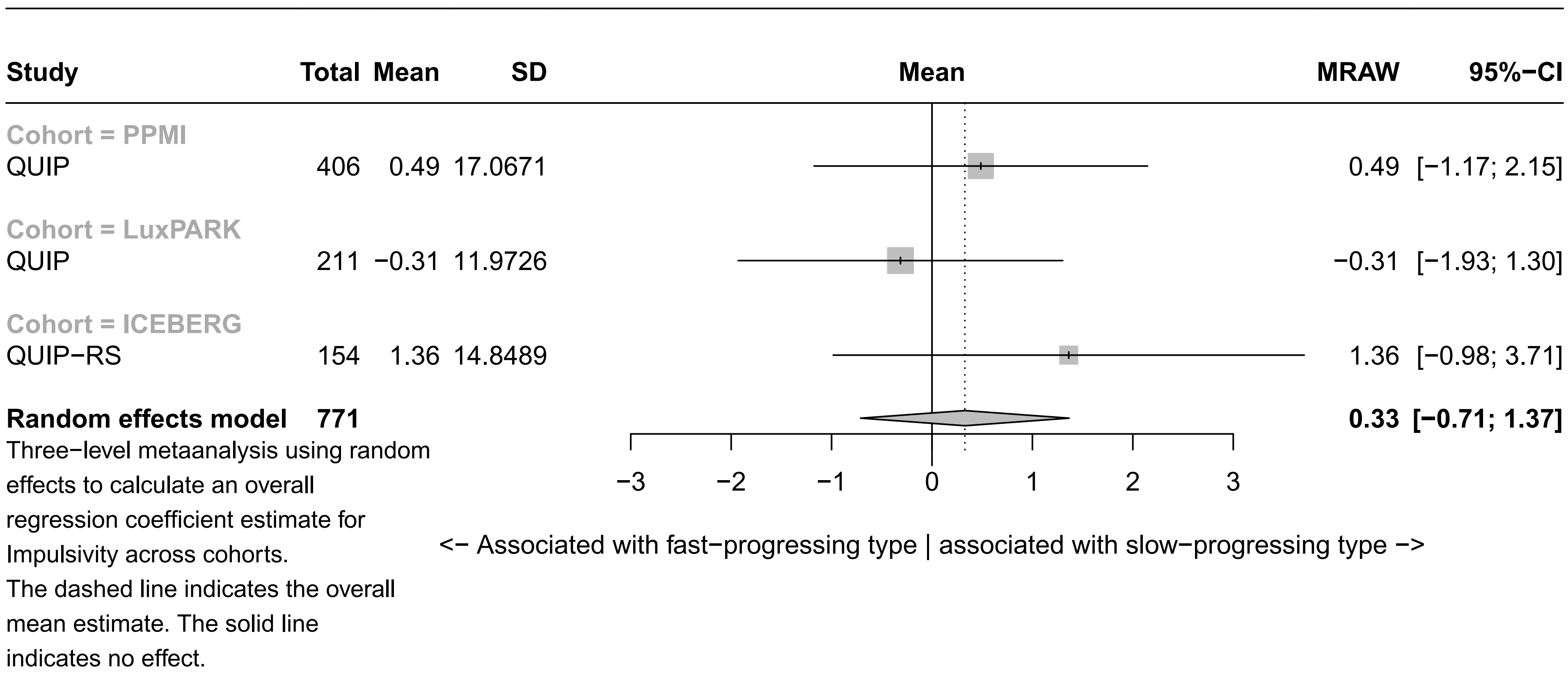

Forest plot for baseline characteristics of symptom domain Tremor

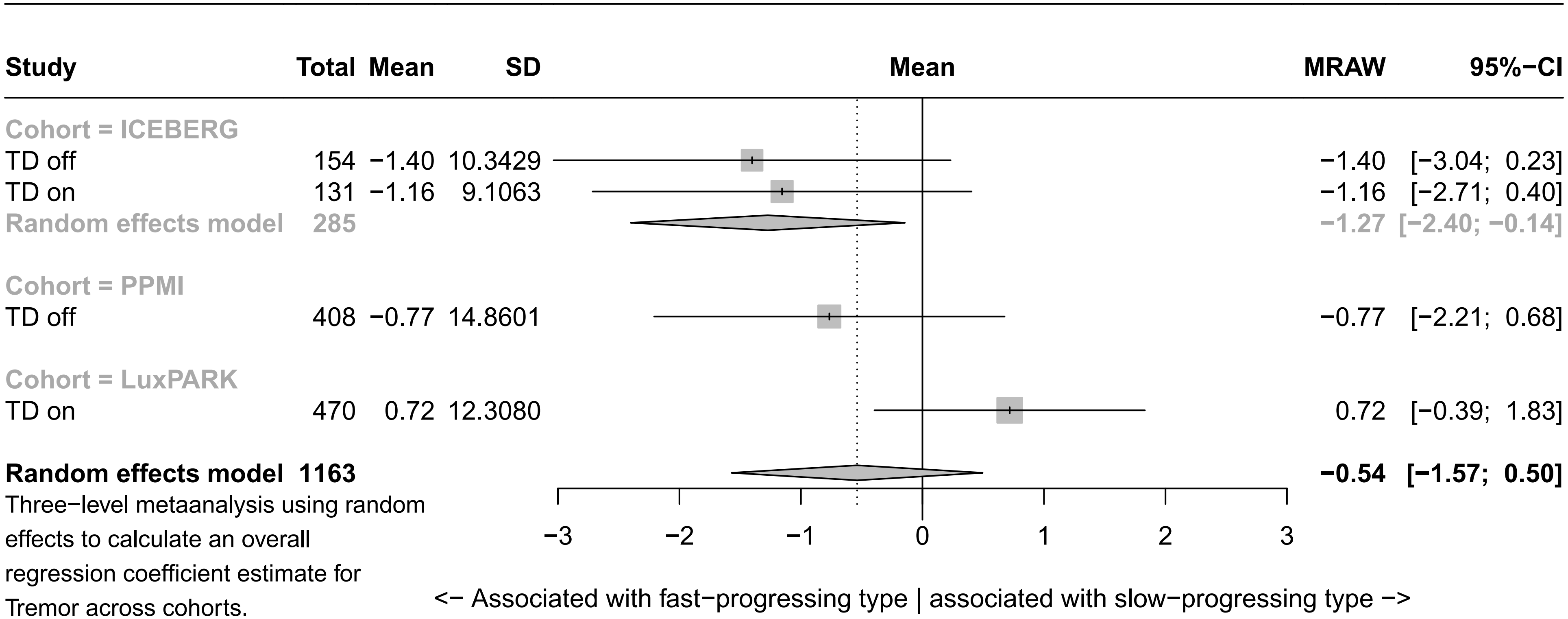

Three−level metaanalysis using random effects to calculate an overall regression coefficient estimate for Tremor across cohorts.
The dashed line indicates the overall mean estimate. The solid line indicates no effect.

Forest plot for baseline characteristics of symptom domain Fatigue

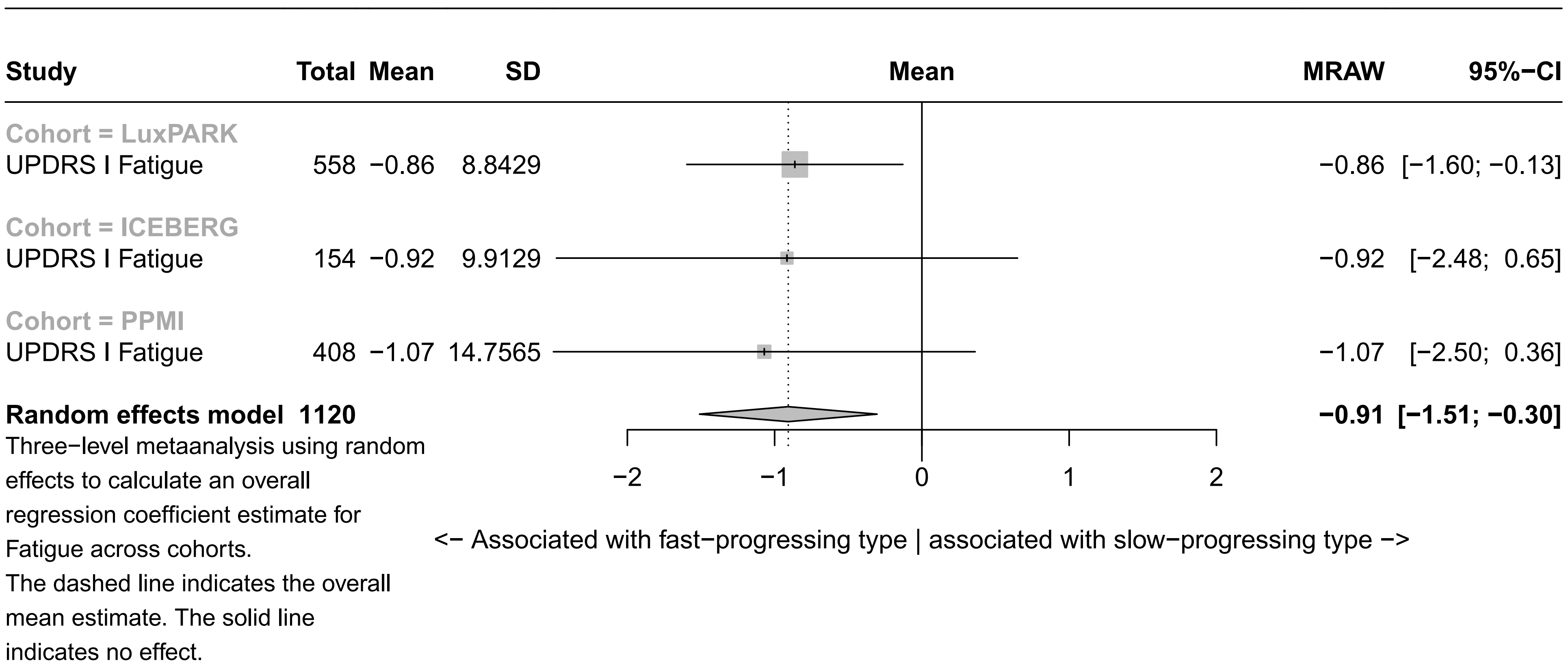

Forest plots for symptom domain baseline associations (cross-cohort validation)

Forest plot for baseline characteristics of symptom domain Axial & PIGD (validation)

Study	Total	Mean	SD	MRAW	95%−CI
**Cohort = ICEBERG (validation)**					
UPDRS III axial off	154	−0.98	15.4066	−0.98	[−3.41; 1.46]
UPDRS III axial on	131	0.24	13.1173	0.24	[−2.01; 2.48]
GABS Examination	153	−0.80	16.1203	−0.80	[−3.36; 1.75]
GABS Questionaire	153	−1.21	15.6229	−1.21	[−3.69; 1.26]
PIGD off	154	−0.27	13.3111	−0.27	[−2.37; 1.83]
PIGD on	131	0.41	12.9408	0.41	[−1.80; 2.63]
**Random effects model**	**876**			**−0.37**	**[−1.32; 0.57]**
**Cohort = LuxPARK (validation)**					
UPDRS III axial on	470	−3.77	16.9217	−3.77	[−5.30; −2.24]
FOGAC	128	−5.80	27.1058	−5.80	[−10.50; −1.11]
FOGQ	201	−2.84	15.0671	−2.84	[−4.92; −0.75]
NFOGQ	36	−5.66	15.1159	−5.66	[−10.60; −0.72]
PDQ39 Mobility	545	−2.20	12.8964	−2.20	[−3.28; −1.12]
PIGD on	468	−2.78	16.2694	−2.78	[−4.25; −1.31]
TuG	140	−1.15	25.7075	−1.15	[−5.41; 3.11]
**Random effects model**	**1988**			**−2.87**	**[−3.62; −2.12]**
**Random effects model**	**2864**			**−1.83**	**[−2.70; −0.95]**

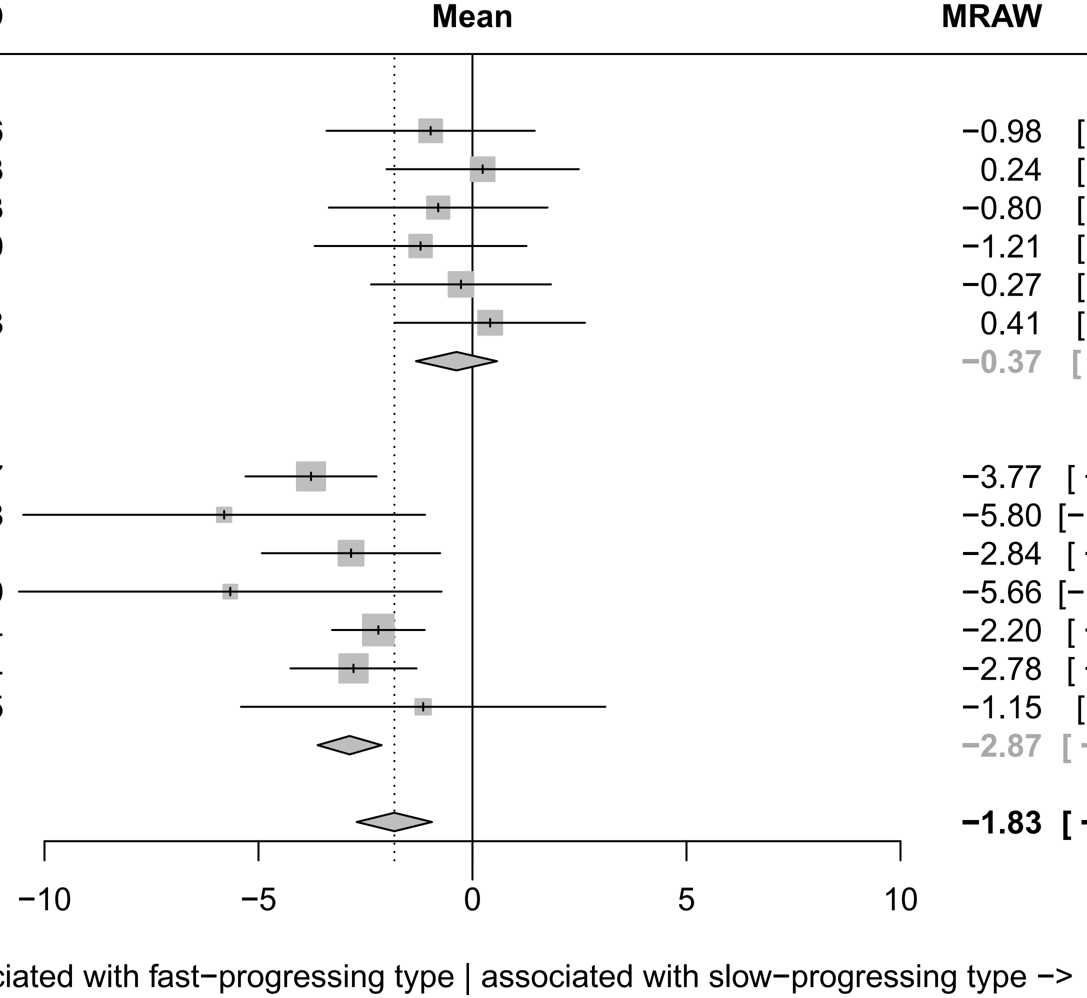

Three−level metaanalysis using random effects to calculate an overall regression coefficient estimate for Axial & PIGD across cohorts. The dashed line indicates the overall mean estimate. The solid line indicates no effect.

Forest plot for baseline characteristics of symptom domain Depression (validation)

Study	Total	Mean	SD	MRAW	95%−CI
**Cohort = LuxPARK (validation)**					
BDI	545	−1.33	19.7130	−1.33	[−2.98; 0.33]
NMSQ Depression	543	−0.28	6.9264	−0.28	[−0.86; 0.30]
PDQ39 Depression	548	−0.40	12.6264	−0.40	[−1.46; 0.66]
UPDRS I Depression	558	−0.27	11.7509	−0.27	[−1.24; 0.71]
**Random effects model**	**2194**			**−0.37**	**[−0.81; 0.06]**
**Cohort = ICEBERG (validation)**					
HADS depression	154	−1.05	14.8282	−1.05	[−3.39; 1.29]
NMSQ Depression	154	−0.76	7.8135	−0.76	[−1.99; 0.48]
UPDRS I Depression	154	−1.00	9.8141	−1.00	[−2.55; 0.55]
**Random effects model**	**462**			**−0.88**	**[−1.77; 0.01]**
**Random effects model**	**2656**			**−0.47**	**[−0.86; −0.08]**

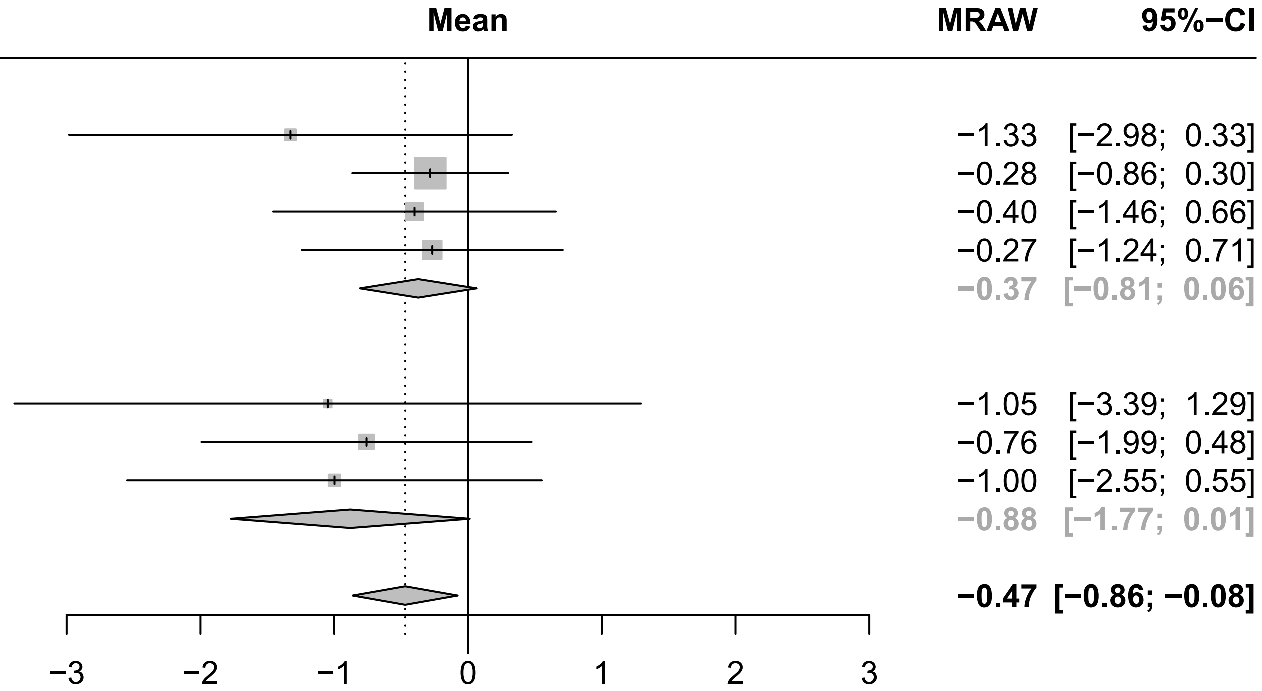

<− Associated with fast−progressing type | associated with slow−progressing type −>

Three−level metaanalysis using random effects to calculate an overall regression coefficient estimate for Depression across cohorts.
The dashed line indicates the overall mean estimate. The solid line indicates no effect.

Forest plot for baseline characteristics of symptom domain Memory (validation)

Study	Total	Mean	SD	MRAW	95%−CI
Cohort = LuxPARK (validation)					
CERAD Words DR	212	−0.89	11.3786	−0.89	[−2.42; 0.65]
CERAD Words IR	216	−1.80	15.0581	−1.80	[−3.81; 0.21]
CERAD Words Recognition	217	0.70	22.9648	0.70	[−2.36; 3.75]
MOCA Orientation + Memory	559	−3.07	14.2284	−3.07	[−4.25; −1.89]
NMSQ Memory	545	−0.59	5.4772	−0.59	[−1.05; −0.13]
PDQ39 Memory	549	−1.08	10.1624	−1.08	[−1.93; −0.23]
SIQCDE Memory	234	−4.86	25.5950	−4.86	[−8.14; −1.58]
Random effects model	2532			−1.49	[−2.46; −0.52]
Cohort = ICEBERG (validation)					
MATTIS Memory	154	−0.02	12.6948	−0.02	[−2.03; 1.98]
MMSE Memory	154	−0.64	9.1126	−0.64	[−2.08; 0.80]
MOCA Orientation + Memory	154	2.03	17.0214	2.03	[−0.66; 4.72]
NMSQ Memory	152	−0.96	6.6616	−0.96	[−2.01; 0.10]
Random effects model	614			−0.48	[−1.26; 0.31]
**Random effects model**	**3146**			**−1.04**	**[−1.76; −0.33]**

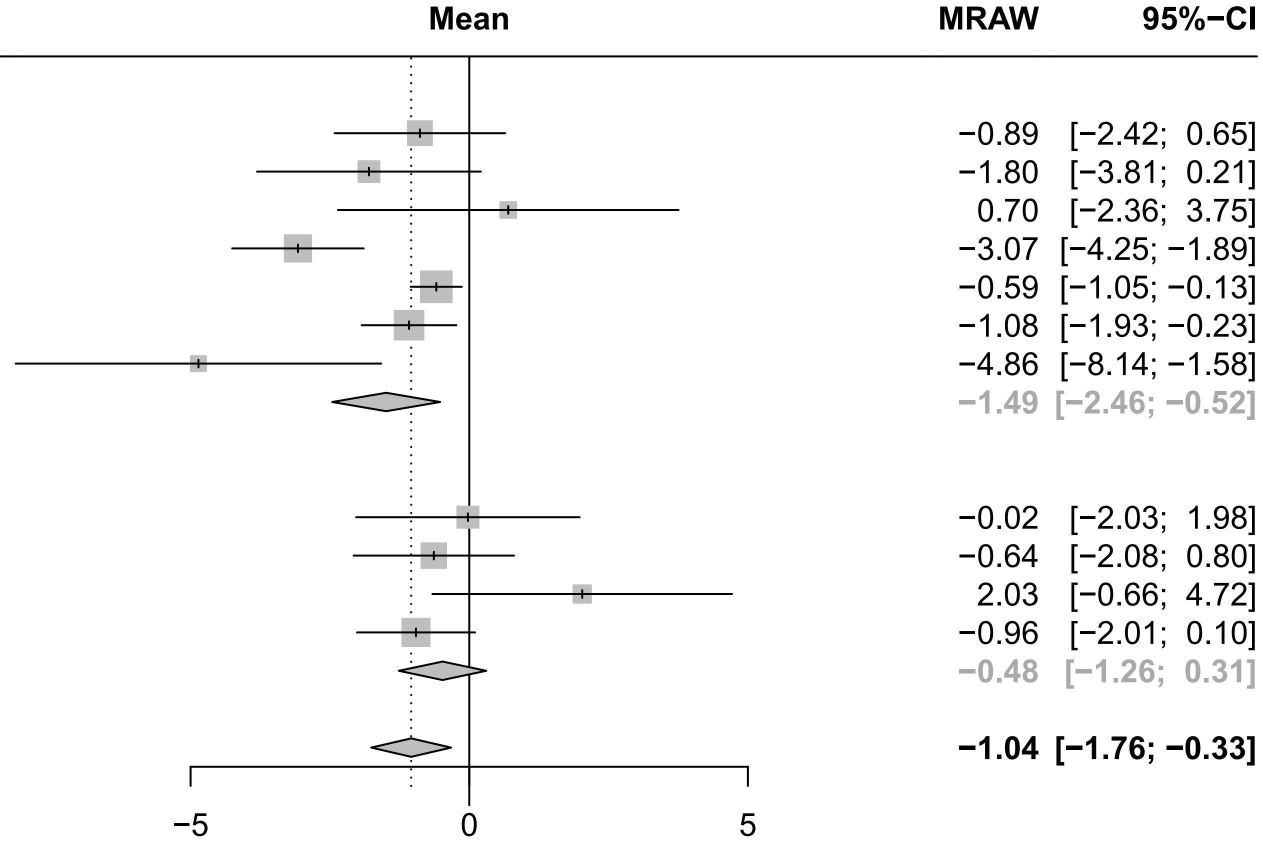

<− Associated with fast−progressing type | associated with slow−progressing type −>

Three−level metaanalysis using random effects to calculate an overall regression coefficient estimate for Memory across cohorts.
The dashed line indicates the overall mean estimate. The solid line indicates no effect.

Forest plot for baseline characteristics of symptom domain Overall severity (validation)

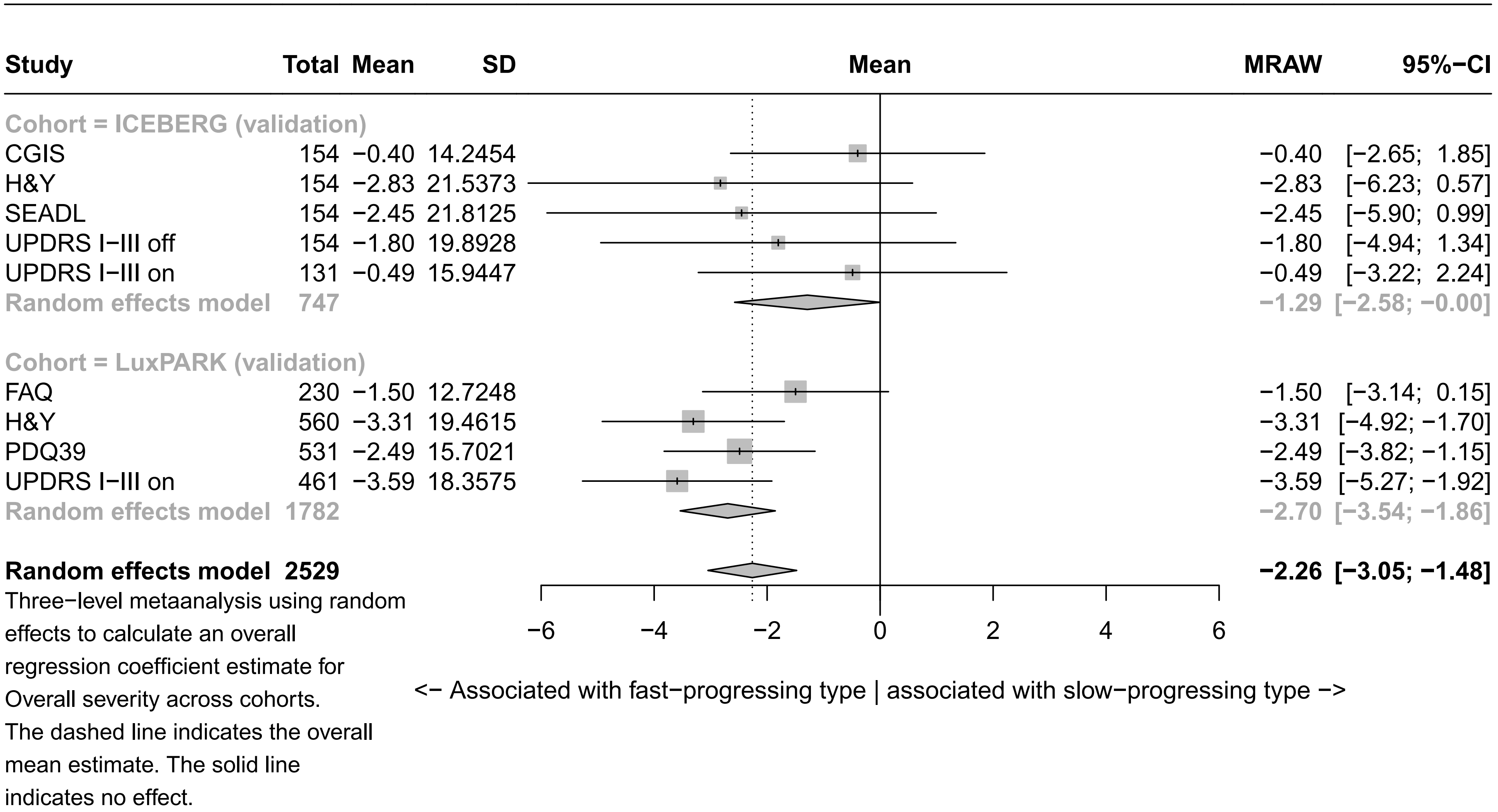

Forest plot for baseline characteristics of symptom domain Apathy (validation)

Study	Total	Mean	SD	MRAW	95%−CI
**Cohort = ICEBERG (validation)**					
DAS	108	−0.09	20.9958	−0.09	[−4.05; 3.87]
SAS	154	−4.30	18.2740	−4.30	[−7.18; −1.41]
UPDRS I Apathy	154	−1.40	10.8177	−1.40	[−3.11; 0.31]
**Random effects model**	**416**			**−2.02**	**[−4.15; 0.11]**
**Cohort = LuxPARK (validation)**					
SAS	542	−1.81	15.7765	−1.81	[−3.14; −0.48]
UPDRS I Apathy	557	0.43	13.4736	0.43	[−0.69; 1.55]
**Random effects model**	**1099**			**−0.66**	**[−2.85; 1.53]**
**Random effects model**	**1515**			**−1.30**	**[−2.78; 0.18]**

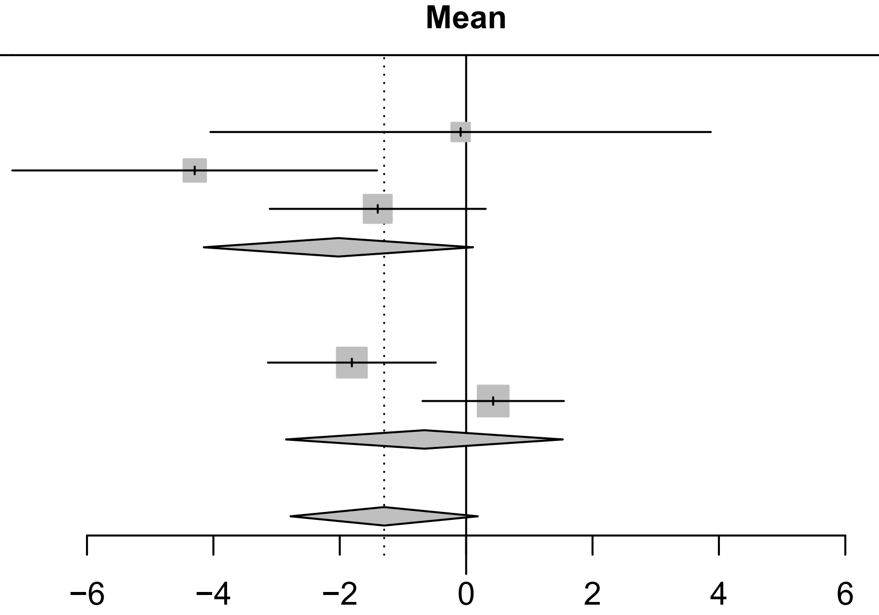

<− Associated with fast−progressing type | associated with slow−progressing type −>

Three−level metaanalysis using random effects to calculate an overall regression coefficient estimate for Apathy across cohorts.
The dashed line indicates the overall mean estimate. The solid line indicates no effect.

Forest plot for baseline characteristics of symptom domain Sleep (validation)

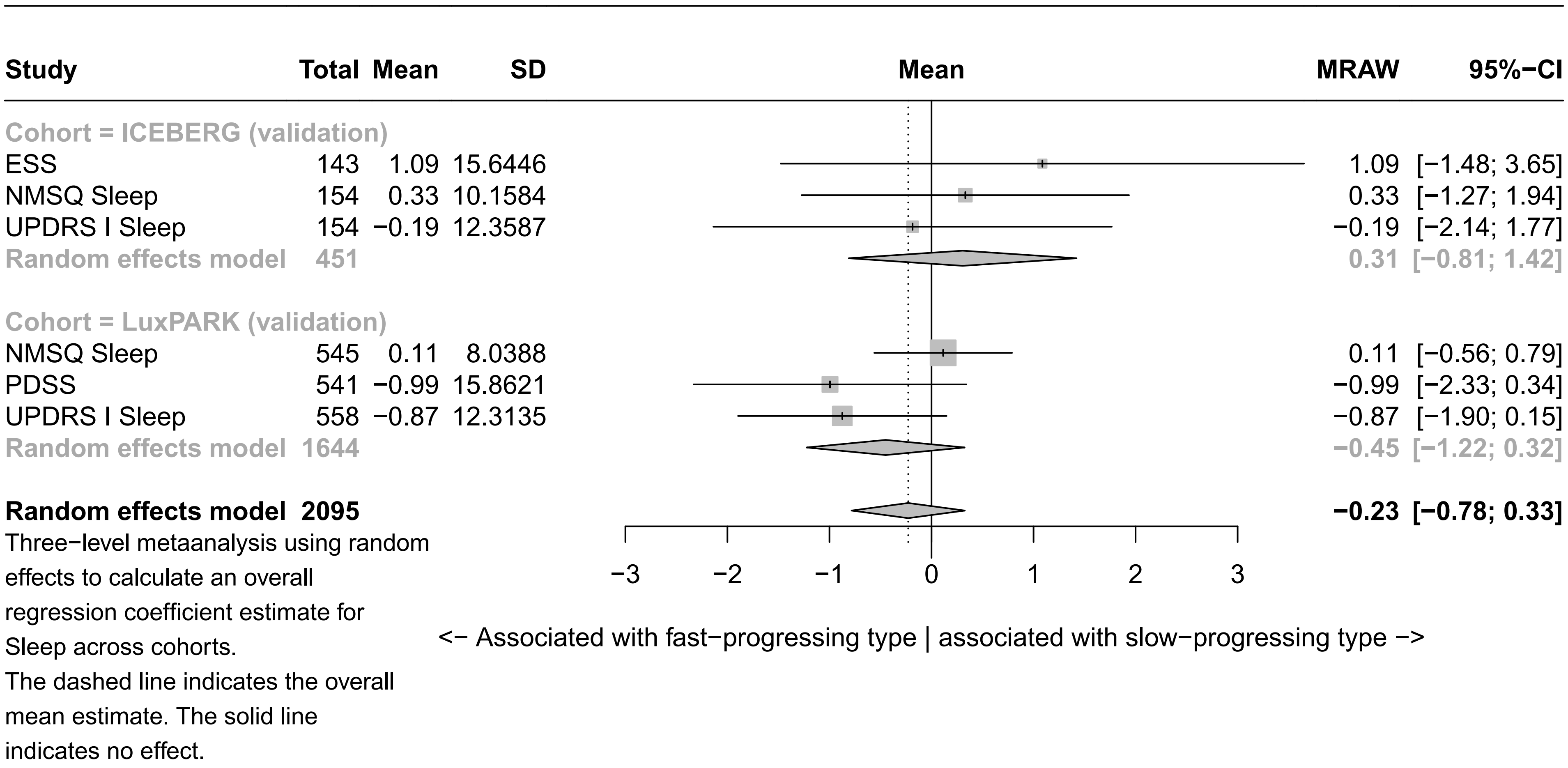

Forest plot for baseline characteristics of symptom domain Overall cognition (validation)

Study	Total	Mean	SD	MRAW	95%−CI
**Cohort = ICEBERG (validation)**					
FAB	154	−0.58	12.0418	−0.58	[−2.48; 1.33]
MATTIS	154	−1.71	14.6102	−1.71	[−4.02; 0.59]
MMSE	154	−1.60	8.5313	−1.60	[−2.95; −0.25]
MoCA	154	0.07	13.7346	0.07	[−2.10; 2.24]
NMSQ Cognition	154	−0.98	8.2256	−0.98	[−2.28; 0.32]
UPDRS I Cognition	154	−0.97	13.8921	−0.97	[−3.16; 1.23]
**Random effects model**	**924**			**−1.05**	**[−1.75; −0.35]**
**Cohort = LuxPARK (validation)**					
FAB	235	−1.92	11.2959	−1.92	[−3.36; −0.47]
MoCA	559	−3.77	14.7988	−3.77	[−5.00; −2.55]
NMSQ Cognition	544	−0.72	6.6093	−0.72	[−1.28; −0.17]
PDQ39 Cognition	549	−1.45	11.4380	−1.45	[−2.41; −0.50]
SIQCDE	232	−4.61	25.8950	−4.61	[−7.95; −1.28]
UPDRS I Cognition	558	−1.64	12.1066	−1.64	[−2.64; −0.64]
**Random effects model**	**2677**			**−2.02**	**[−3.03; −1.00]**
**Random effects model**	**3601**			**−1.55**	**[−2.16; −0.93]**

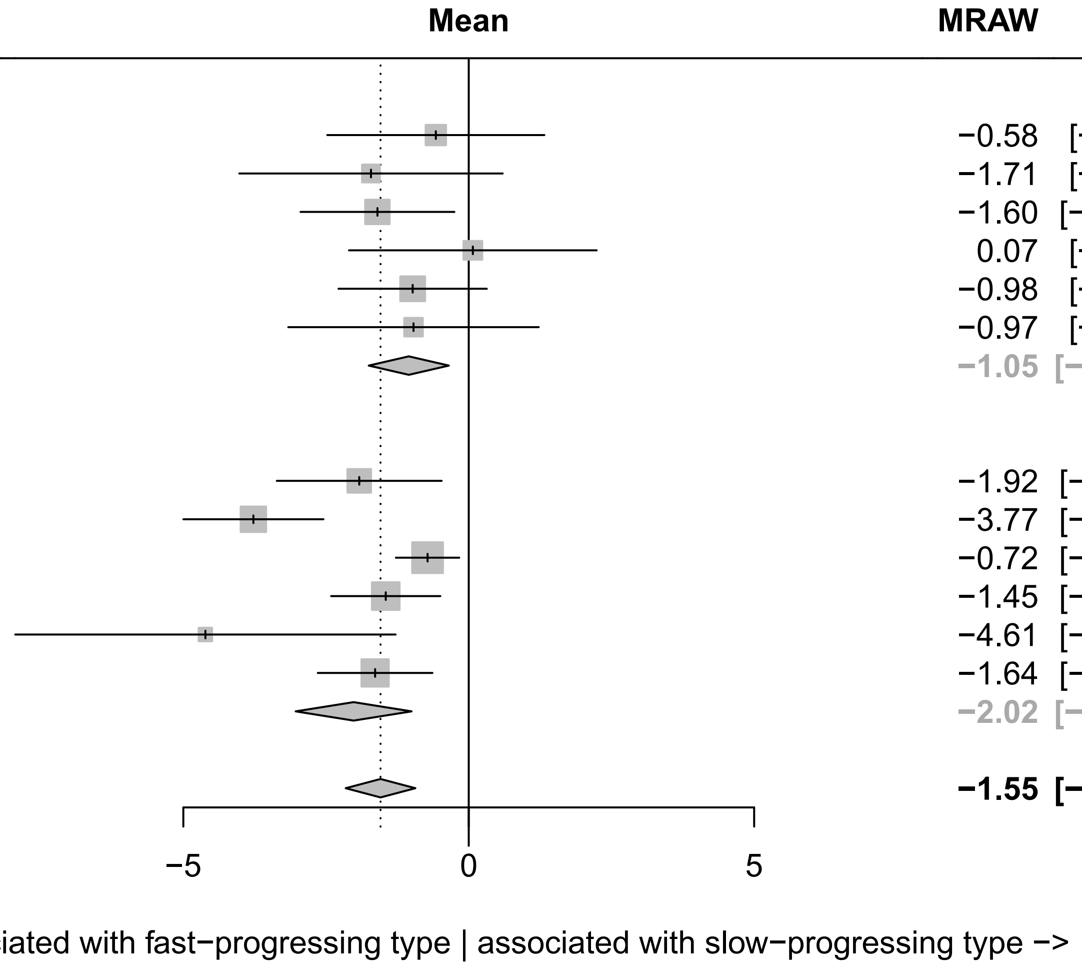

Three−level metaanalysis using random effects to calculate an overall regression coefficient estimate for Overall cognition across cohorts. The dashed line indicates the overall mean estimate. The solid line indicates no effect.

Forest plot for baseline characteristics of symptom domain Conceptualization (validation)

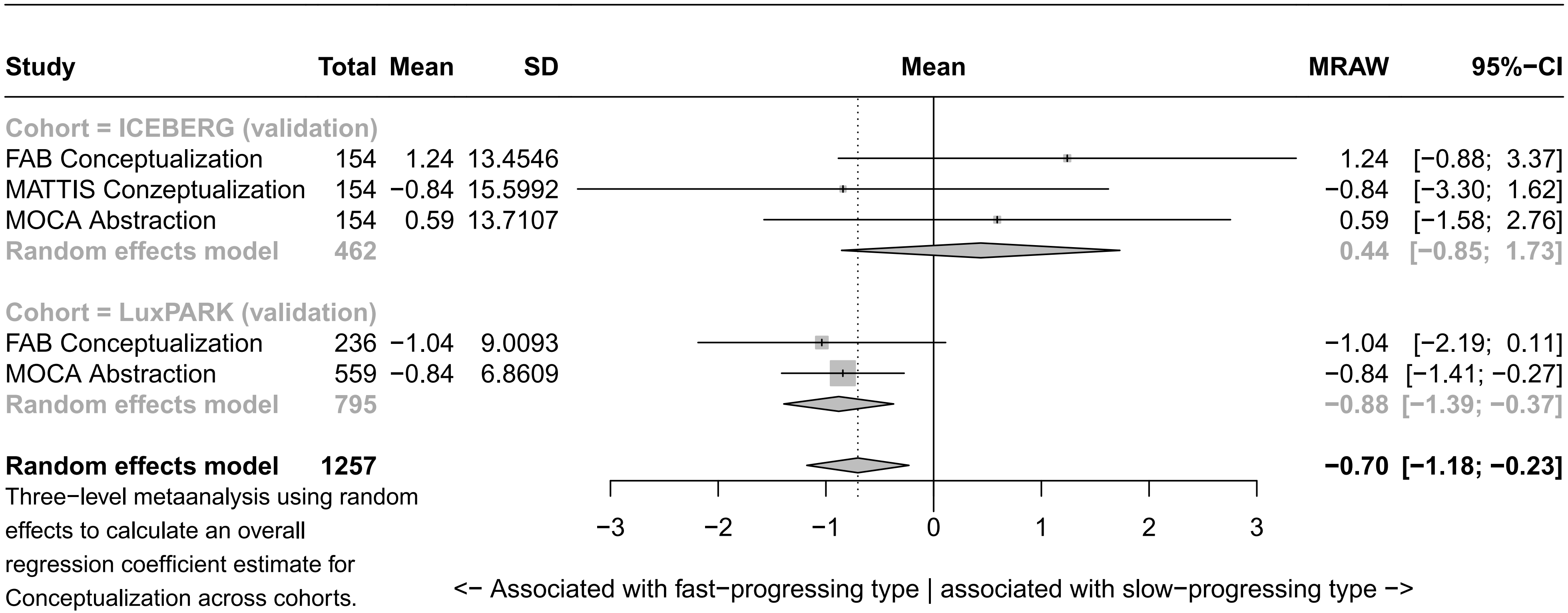

Three−level metaanalysis using random effects to calculate an overall regression coefficient estimate for Conceptualization across cohorts. The dashed line indicates the overall mean estimate. The solid line indicates no effect.

Forest plot for baseline characteristics of symptom domain Visuo−executive (validation)

Study	Total	Mean	SD	MRAW	95%−CI
**Cohort = ICEBERG (validation)**					
FAB 3−6	154	−0.72	12.0433	−0.72	[−2.62; 1.18]
MATTIS Initiation + Construction	154	−0.78	15.7835	−0.78	[−3.28; 1.71]
MMSE Construction	154	−3.23	16.4080	−3.23	[−5.83; −0.64]
MOCA Visuspatial/Executive	154	−1.45	9.5593	−1.45	[−2.96; 0.06]
**Random effects model**	**616**			**−1.41**	**[−2.39; −0.42]**
**Cohort = LuxPARK (validation)**					
FAB 3−6	235	−1.65	11.5494	−1.65	[−3.12; −0.17]
Judgement Line Orientation	214	−2.22	13.3302	−2.22	[−4.01; −0.44]
MOCA Visuspatial/Executive	560	−2.41	10.6503	−2.41	[−3.30; −1.53]
Stroop Errors	208	−6.85	45.6635	−6.85	[−13.05; −0.64]
Stroop Time	208	−3.66	17.3974	−3.66	[−6.02; −1.29]
Trailmaking A	549	−5.82	25.9216	−5.82	[−7.99; −3.66]
Trailmaking B	480	−4.18	19.9336	−4.18	[−5.96; −2.40]
**Random effects model**	**2454**			**−3.27**	**[−4.45; −2.10]**
**Random effects model**	**3070**			**−2.63**	**[−3.56; −1.70]**

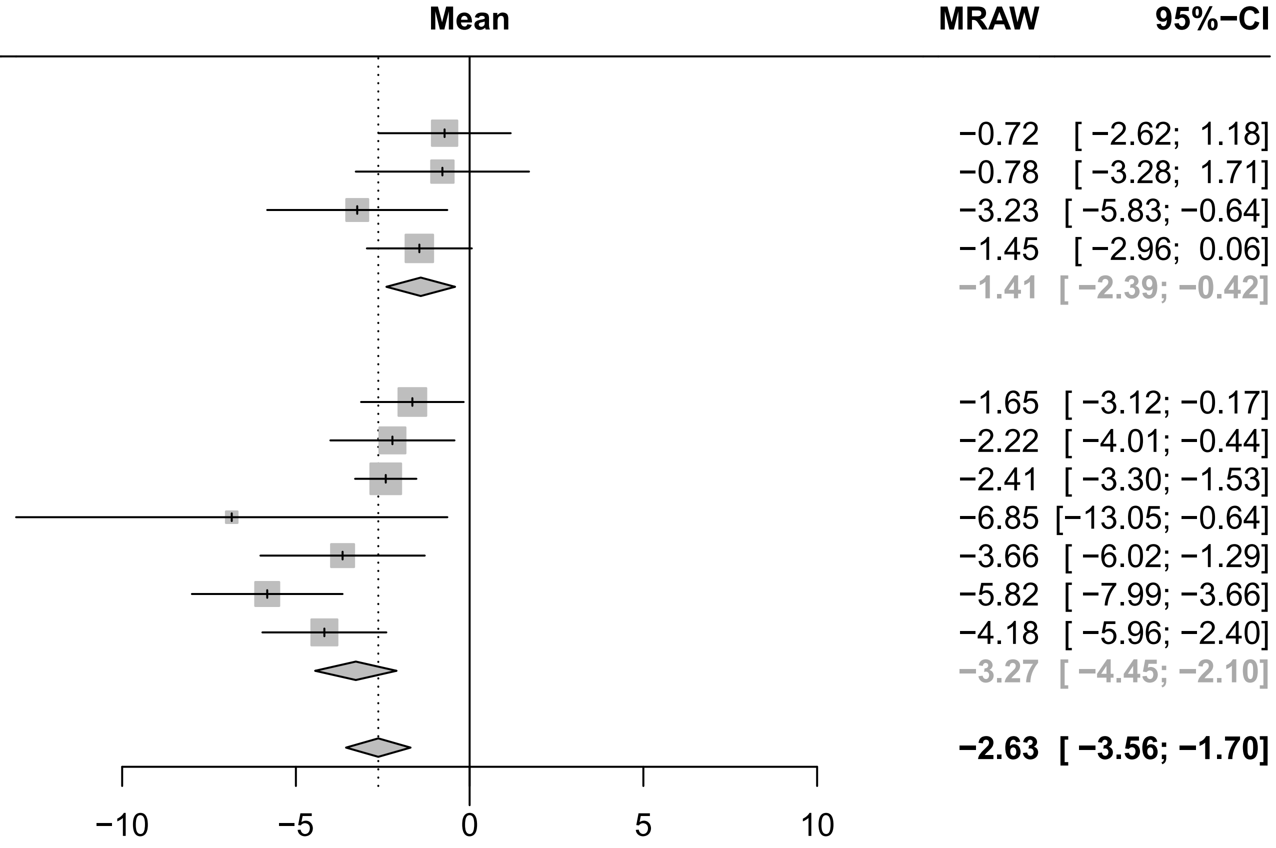

<− Associated with fast−progressing type | associated with slow−progressing type −>

Three−level metaanalysis using random effects to calculate an overall regression coefficient estimate for Visuo−executive across cohorts. The dashed line indicates the overall mean estimate. The solid line indicates no effect.

Forest plot for baseline characteristics of symptom domain Language (validation)

Study	Total	Mean	SD	MRAW	95%−CI
**Cohort = ICEBERG (validation)**					
FAB VFT	154	−0.55	10.5285	−0.55	[−2.21; 1.11]
MMSE Language	154	−0.48	11.9227	−0.48	[−2.36; 1.41]
MOCA Language + Naming	154	−0.84	11.6445	−0.84	[−2.68; 1.00]
VFT phonematic F	151	−1.39	15.1994	−1.39	[−3.81; 1.03]
VFT phonematic S	143	−0.53	15.4382	−0.53	[−3.06; 2.00]
VFT semantic supermarket	151	−2.42	19.5949	−2.42	[−5.55; 0.70]
**Random effects model**	**907**			**−0.84**	**[−1.70; 0.01]**
**Cohort = LuxPARK (validation)**					
FAB VFT	236	−1.28	10.0470	−1.28	[−2.56; 0.00]
MOCA Language + Naming	559	−2.63	15.7566	−2.63	[−3.94; −1.32]
VFT phonematic F	548	−3.19	17.7775	−3.19	[−4.67; −1.70]
VFT phonematic S	221	−3.32	19.1413	−3.32	[−5.84; −0.79]
VFT semantic sum	210	−4.27	16.9366	−4.27	[−6.56; −1.98]
**Random effects model**	**1774**			**−2.70**	**[−3.70; −1.71]**
**Random effects model**	**2681**			**−1.85**	**[−2.62; −1.09]**

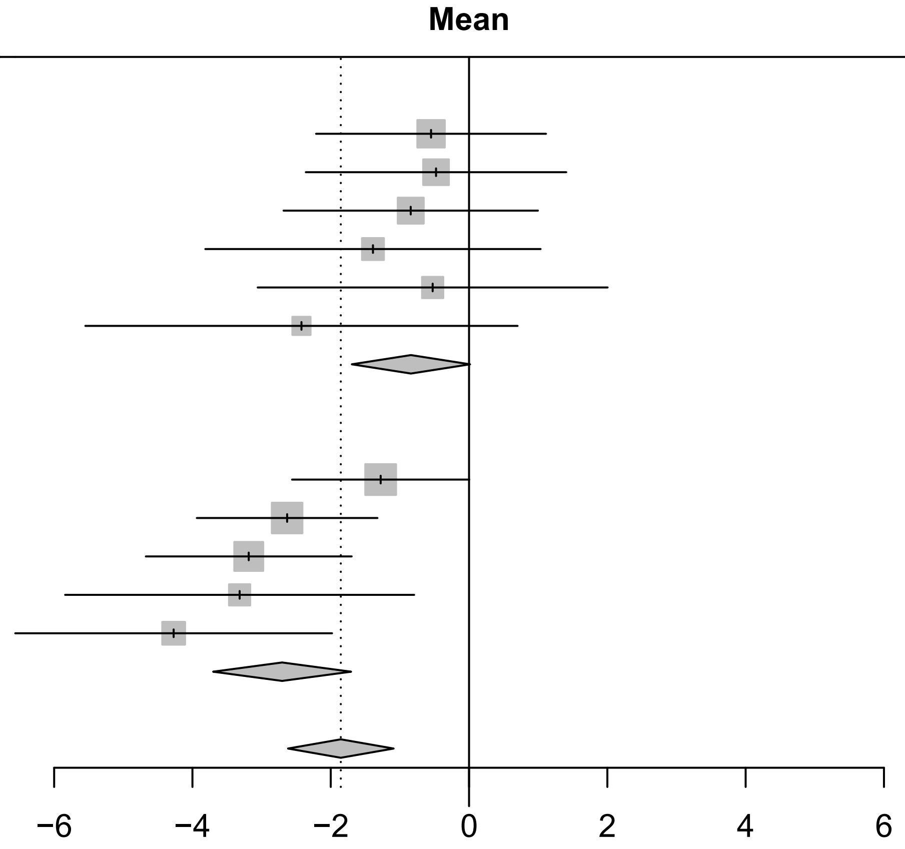

<− Associated with fast−progressing type | associated with slow−progressing type −>

Three−level metaanalysis using random effects to calculate an overall regression coefficient estimate for Language across cohorts.
The dashed line indicates the overall mean estimate. The solid line indicates no effect.

Forest plot for baseline characteristics of symptom domain Anxiety (validation)

Study	Total	Mean	SD	MRAW	95%−CI
**Cohort = ICEBERG (validation)**					
HADS anxiety	154	0.08	17.9763	0.08	[−2.76; 2.92]
NMSQ Anxiety	152	−0.83	6.4206	−0.83	[−1.85; 0.19]
UPDRS I Anxiety	154	−0.49	12.1763	−0.49	[−2.41; 1.44]
**Random effects model**	**460**			**−0.68**	**[−1.54; 0.18]**
**Cohort = LuxPARK (validation)**					
NMSQ Anxiety	543	0.13	6.1216	0.13	[−0.38; 0.65]
PDQ39 Anxiety	550	−0.50	10.0075	−0.50	[−1.33; 0.34]
UPDRS I Anxiety	558	0.36	13.3960	0.36	[−0.75; 1.47]
**Random effects model**	**1651**			**0.01**	**[−0.39; 0.42]**
**Random effects model**	**2111**			**−0.15**	**[−0.59; 0.28]**

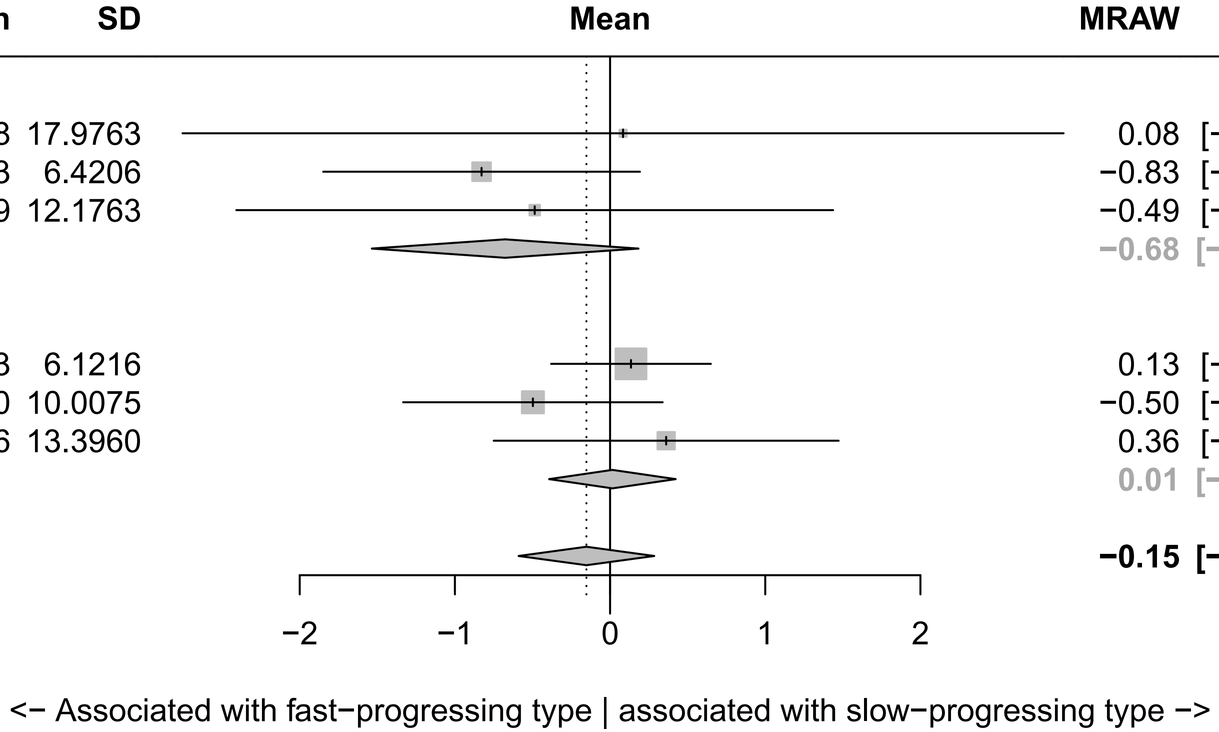

Three−level metaanalysis using random effects to calculate an overall regression coefficient estimate for Anxiety across cohorts.
The dashed line indicates the overall mean estimate. The solid line indicates no effect.

Forest plot for baseline characteristics of symptom domain Attention (validation)

Study	Total	Mean	SD	MRAW	95%−CI
Cohort = ICEBERG (validation)					
MATTIS Attention	154	−3.94	15.4627	−3.94	[−6.38; −1.49]
MMSE Attention	154	−1.81	11.8440	−1.81	[−3.68; 0.06]
MOCA Attention	154	0.15	12.0695	0.15	[−1.76; 2.05]
NMSQ Attention	152	−0.61	6.8590	−0.61	[−1.70; 0.48]
Random effects model	614			−1.37	[−2.90; 0.16]
Cohort = LuxPARK (validation)					
MOCA Attention	559	−1.78	14.4209	−1.78	[−2.97; −0.58]
NMSQ Attention	545	−0.40	5.6128	−0.40	[−0.87; 0.07]
PDQ39 Attention	549	−1.21	10.1009	−1.21	[−2.05; −0.36]
SIQCDE Attention	233	−0.84	16.8653	−0.84	[−3.00; 1.33]
Digit Span	214	−2.38	15.1360	−2.38	[−4.41; −0.35]
Random effects model	2100			−1.11	[−1.81; −0.40]
**Random effects model**	**2714**			**−1.14**	**[−1.74; −0.55]**

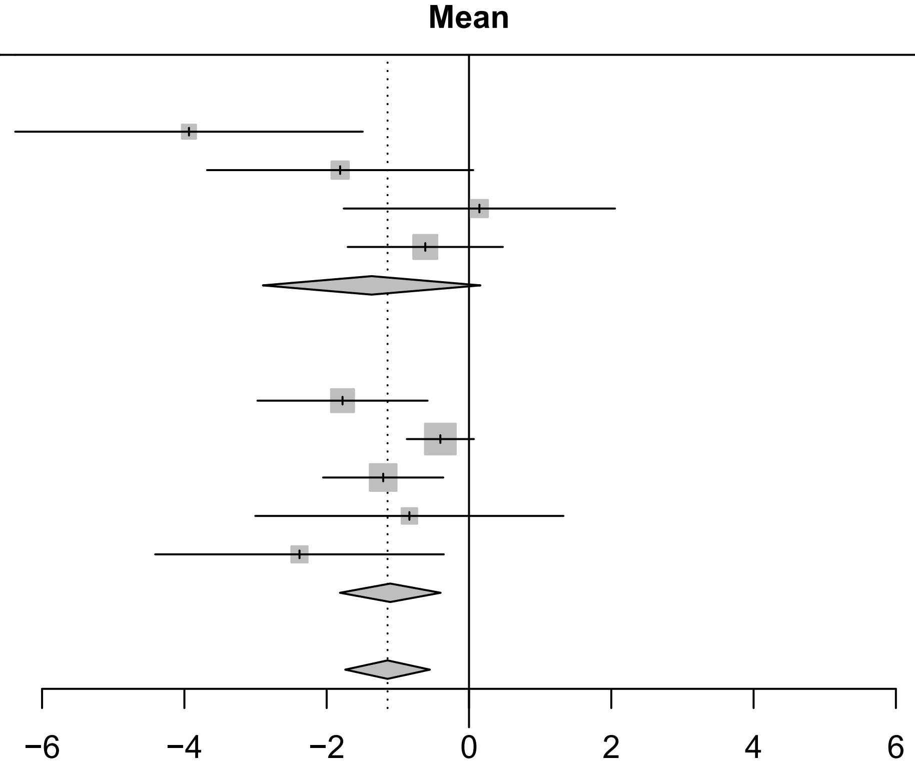

<− Associated with fast−progressing type | associated with slow−progressing type −>

Three−level metaanalysis using random effects to calculate an overall regression coefficient estimate for Attention across cohorts.
The dashed line indicates the overall mean estimate. The solid line indicates no effect.

Forest plot for baseline characteristics of symptom domain Non motor symptoms (validation)

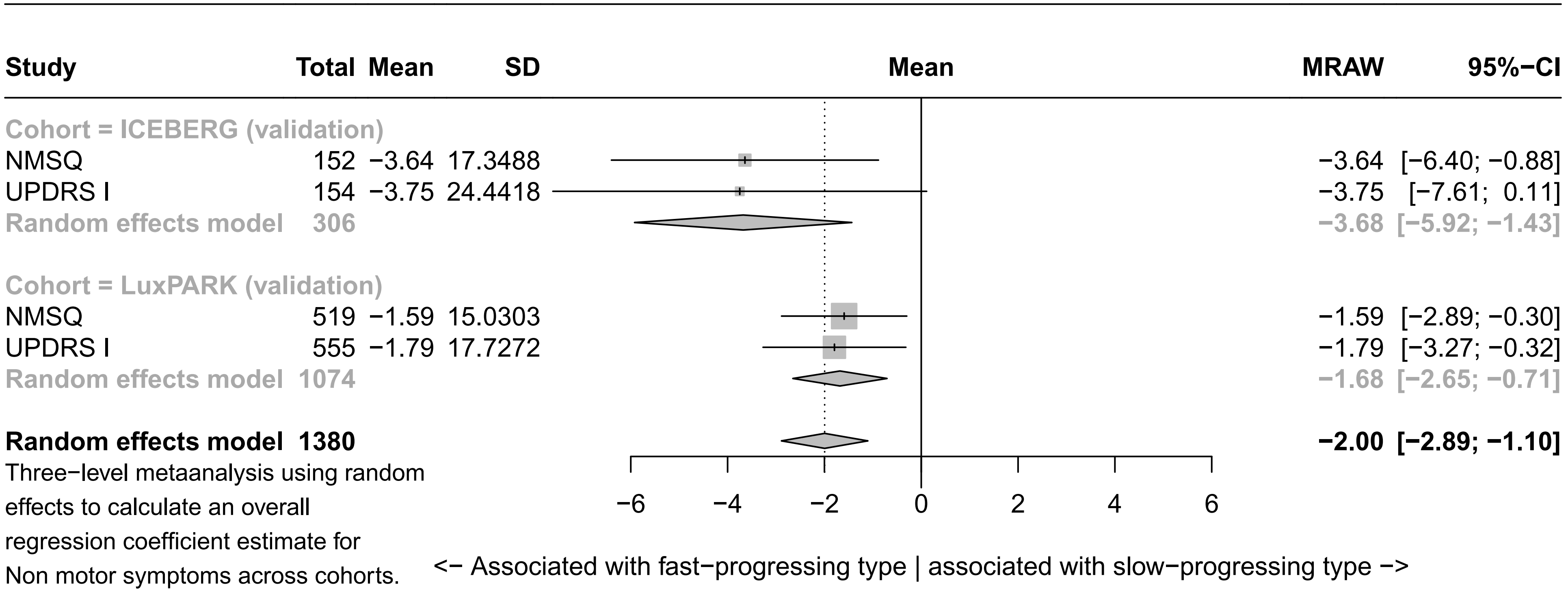

Three−level metaanalysis using random effects to calculate an overall regression coefficient estimate for Non motor symptoms across cohorts. The dashed line indicates the overall mean estimate. The solid line indicates no effect.

Forest plot for baseline characteristics of symptom domain Autonomic (validation)

Study	Total	Mean	SD	MRAW	95%−CI
Cohort = LuxPARK (validation)					
NMSQ Autonomic	525	−1.20	13.7781	−1.20	[−2.38; −0.03]
SCOPA−AUT	547	−2.53	16.0077	−2.53	[−3.88; −1.19]
UPDRS I Autonomic	558	−2.09	16.3374	−2.09	[−3.44; −0.73]
Random effects model	1630			−1.89	[−2.70; −1.08]
Cohort = ICEBERG (validation)					
NMSQ Autonomic	154	−1.51	13.3861	−1.51	[−3.63; 0.60]
SCOPA−AUT	153	0.78	16.5699	0.78	[−1.85; 3.40]
UPDRS I Autonomic	154	−0.55	16.3947	−0.55	[−3.14; 2.04]
Random effects model	461			−0.59	[−1.98; 0.80]
**Random effects model**	**2091**			**−1.56**	**[−2.29; −0.82]**

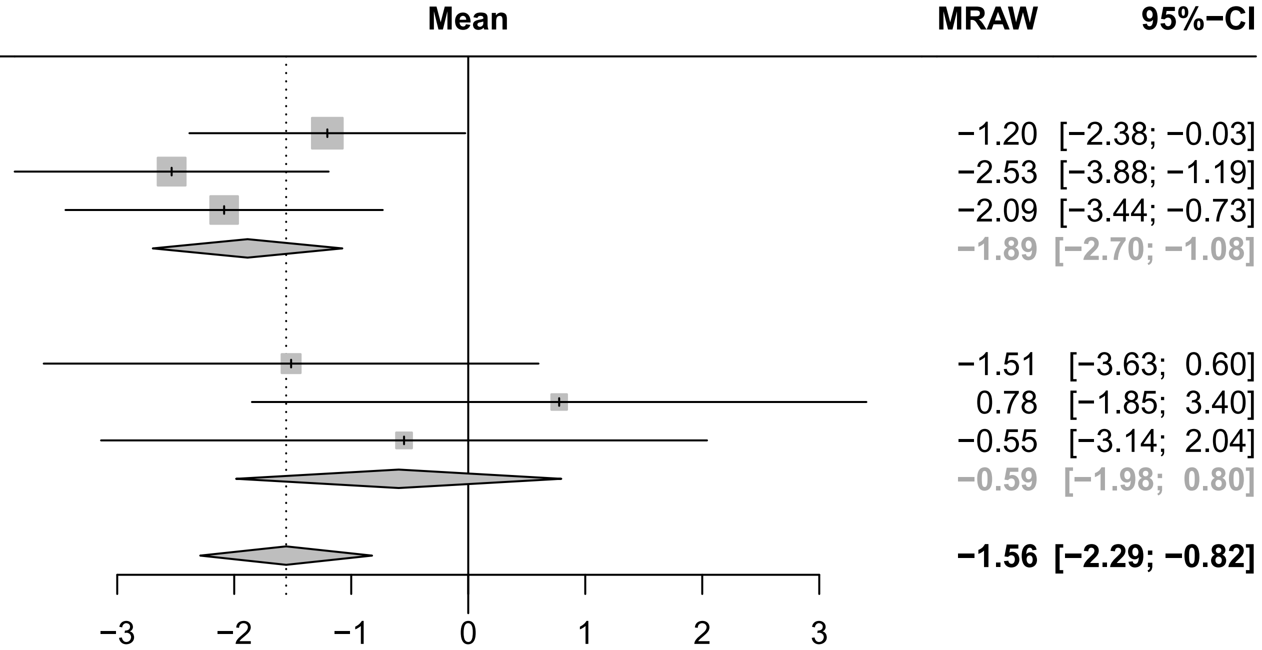

<− Associated with fast−progressing type | associated with slow−progressing type −>

Three−level metaanalysis using random effects to calculate an overall regression coefficient estimate for Autonomic across cohorts.
The dashed line indicates the overall mean estimate. The solid line indicates no effect.

Forest plot for baseline characteristics of symptom domain Hallucinations (validation)

Study	Total	Mean	SD	MRAW	95%−CI
**Cohort = LuxPARK (validation)**					
NMSQ Hallucination	544	−1.11	9.9470	−1.11	[−1.95; −0.28]
UPDRS I Hallucinations	558	−0.97	17.4361	−0.97	[−2.41; 0.48]
**Random effects model**	**1102**			**−1.08**	**[−1.80; −0.35]**
**Cohort = ICEBERG (validation)**					
NMSQ Hallucination	154	−1.32	10.2670	−1.32	[−2.94; 0.30]
UPDRS I Hallucinations	154	−0.42	7.9031	−0.42	[−1.67; 0.82]
**Random effects model**	**308**			**−0.76**	**[−1.75; 0.23]**
**Random effects model**	**1410**			**−0.96**	**[−1.55; −0.38]**

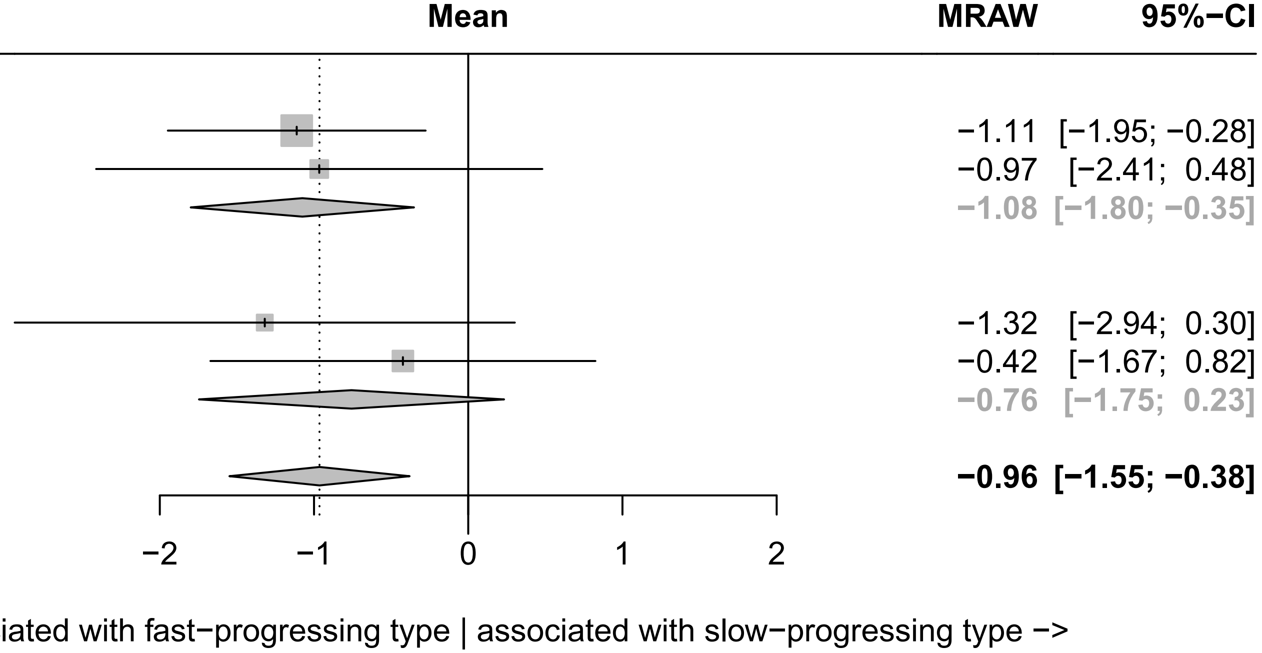

Three−level metaanalysis using random effects to calculate an overall regression coefficient estimate for Hallucinations across cohorts. The dashed line indicates the overall mean estimate. The solid line indicates no effect.

Forest plot for baseline characteristics of symptom domain Pain (validation)

Study	Total	Mean	SD	MRAW	95%−CI
**Cohort = LuxPARK (validation)**					
NMSQ Pain	546	0.22	6.5957	0.22	[−0.33; 0.77]
PDQ39 Pain	547	−0.21	10.8280	−0.21	[−1.12; 0.70]
UPDRS I Pain	558	−0.37	10.1509	−0.37	[−1.22; 0.47]
**Random effects model**	**1651**			**−0.01**	**[−0.42; 0.40]**
**Cohort = ICEBERG (validation)**					
NMSQ Pain	152	−0.44	6.3159	−0.44	[−1.44; 0.57]
UPDRS I Pain	154	−2.83	14.6445	−2.83	[−5.15; −0.52]
**Random effects model**	**306**			**−1.40**	**[−3.70; 0.90]**
**Random effects model**	**1957**			**−0.22**	**[−0.67; 0.24]**

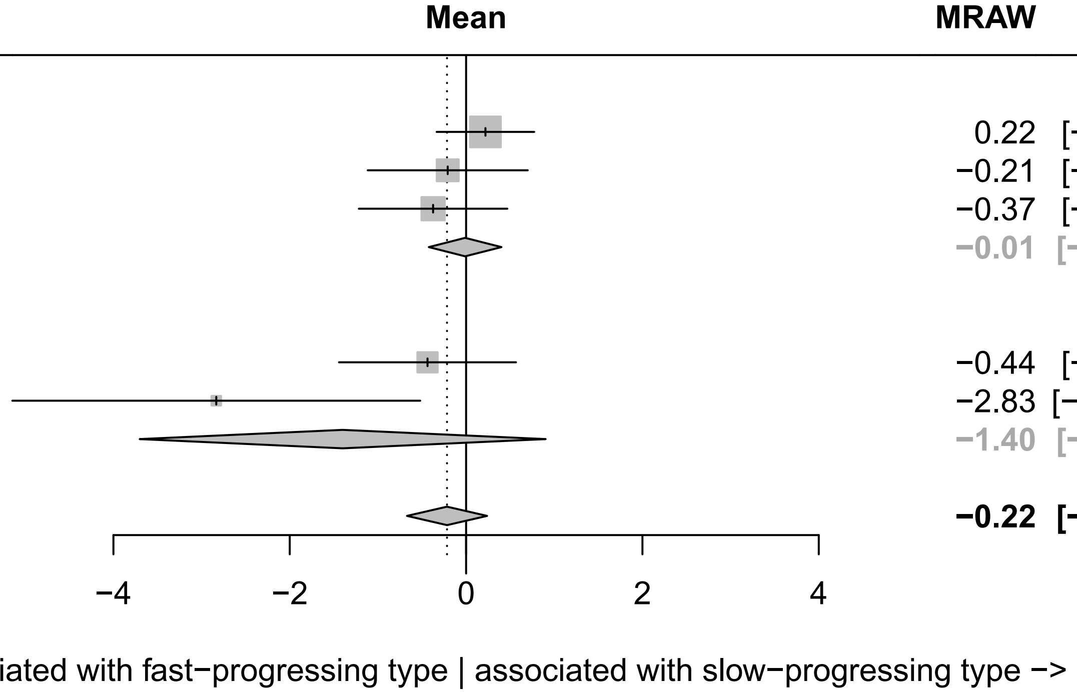

Three−level metaanalysis using random effects to calculate an overall regression coefficient estimate for Pain across cohorts.
The dashed line indicates the overall mean estimate. The solid line indicates no effect.

Forest plot for baseline characteristics of symptom domain RBD (validation)

Study	Total	Mean	SD	MRAW	95%−CI
**Cohort = ICEBERG (validation)**					
NMSQ RBD	154	−1.04	7.4724	−1.04	[−2.22; 0.14]
RBD−HK	144	−2.59	13.7635	−2.59	[−4.84; −0.34]
**Random effects model**	**298**			**−1.51**	**[−2.90; −0.11]**
**Cohort = LuxPARK (validation)**					
NMSQ RBD	545	−0.55	6.7819	−0.55	[−1.12; 0.02]
RBD−SQ	538	−1.19	11.0920	−1.19	[−2.13; −0.26]
**Random effects model**	**1083**			**−0.76**	**[−1.35; −0.16]**
**Random effects model**	**1381**			**−0.92**	**[−1.46; −0.37]**

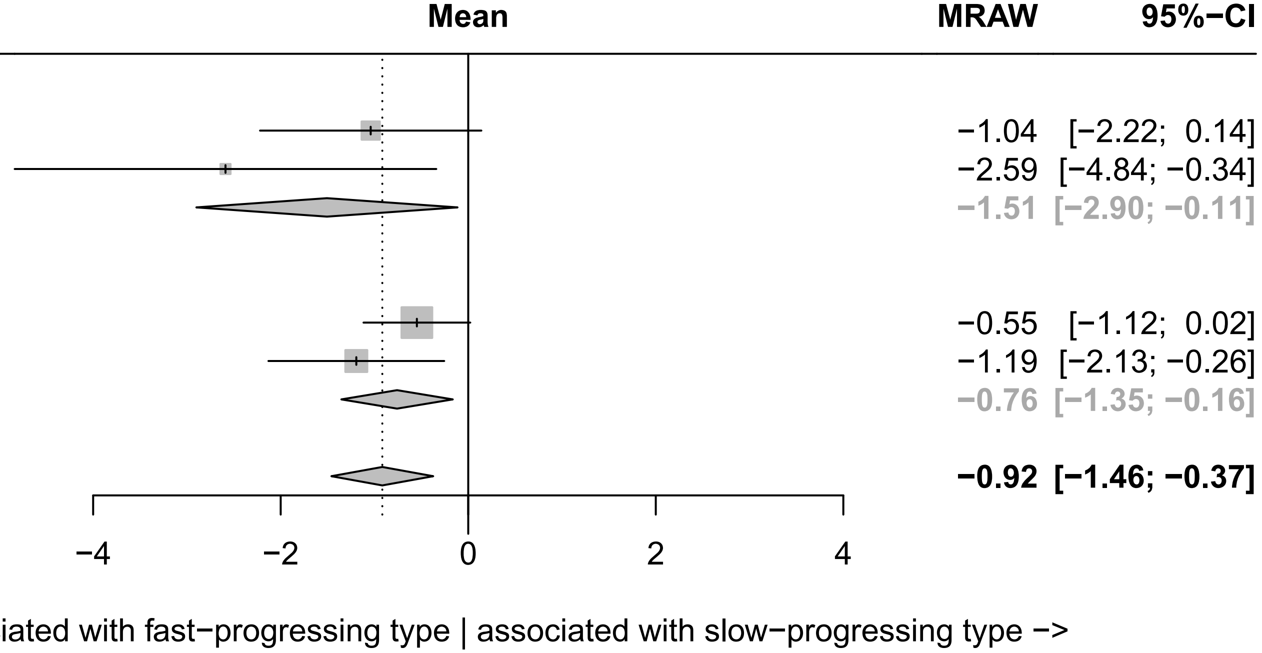

Three−level metaanalysis using random effects to calculate an overall regression coefficient estimate for RBD across cohorts.
The dashed line indicates the overall mean estimate. The solid line indicates no effect.

Forest plot for baseline characteristics of symptom domain Smell (validation)

Study	Total	Mean	SD	MRAW	95%−CI
Cohort = ICEBERG (validation)					
NMSQ Smell	152	−0.46	6.3205	−0.46	[−1.47; 0.54]
UPSIT	146	0.56	13.9305	0.56	[−1.70; 2.82]
Random effects model	298			−0.29	[−1.21; 0.62]
Cohort = LuxPARK (validation)					
NMSQ Smell	546	−0.25	5.3725	−0.25	[−0.70; 0.21]
Sniffin Test	541	−1.47	12.1039	−1.47	[−2.49; −0.45]
Random effects model	1087			−0.77	[−1.95; 0.42]
**Random effects model**	**1385**			**−0.53**	**[−1.17; 0.11]**

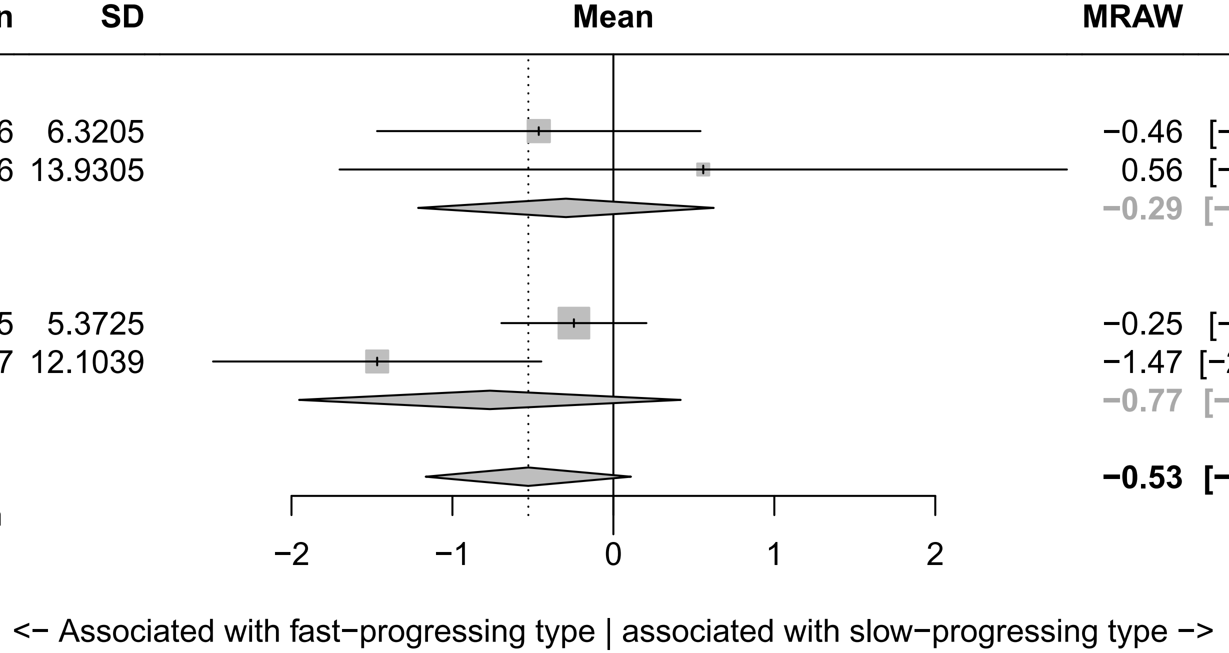

Three−level metaanalysis using random effects to calculate an overall regression coefficient estimate for Smell across cohorts.
The dashed line indicates the overall mean estimate. The solid line indicates no effect.

Forest plot for baseline characteristics of symptom domain Motor symptoms (validation)

Study	Total	Mean	SD	MRAW	95%−CI
**Cohort = LuxPARK (validation)**					
PDQ39 ADL	548	−3.01	12.9061	−3.01	[−4.09; −1.93]
Pegboard	539	−5.08	18.8444	−5.08	[−6.67; −3.48]
UPDRS II	555	−3.34	17.0696	−3.34	[−4.76; −1.92]
UPDRS III on	468	−2.49	16.6776	−2.49	[−4.00; −0.98]
UPDRS IV	556	0.74	18.2493	0.74	[−0.78; 2.26]
**Random effects model**	**2666**			**−2.63**	**[−4.46; −0.81]**
**Cohort = ICEBERG (validation)**					
UPDRS II	154	1.01	16.5643	1.01	[−1.60; 3.63]
UPDRS III off	154	−1.53	20.9631	−1.53	[−4.84; 1.78]
UPDRS III on	131	0.02	12.8990	0.02	[−2.19; 2.23]
UPDRS IV	154	−2.18	26.0911	−2.18	[−6.30; 1.94]
**Random effects model**	**593**			**−0.23**	**[−1.64; 1.18]**
**Random effects model**	**3259**			**−1.86**	**[−3.28; −0.45]**

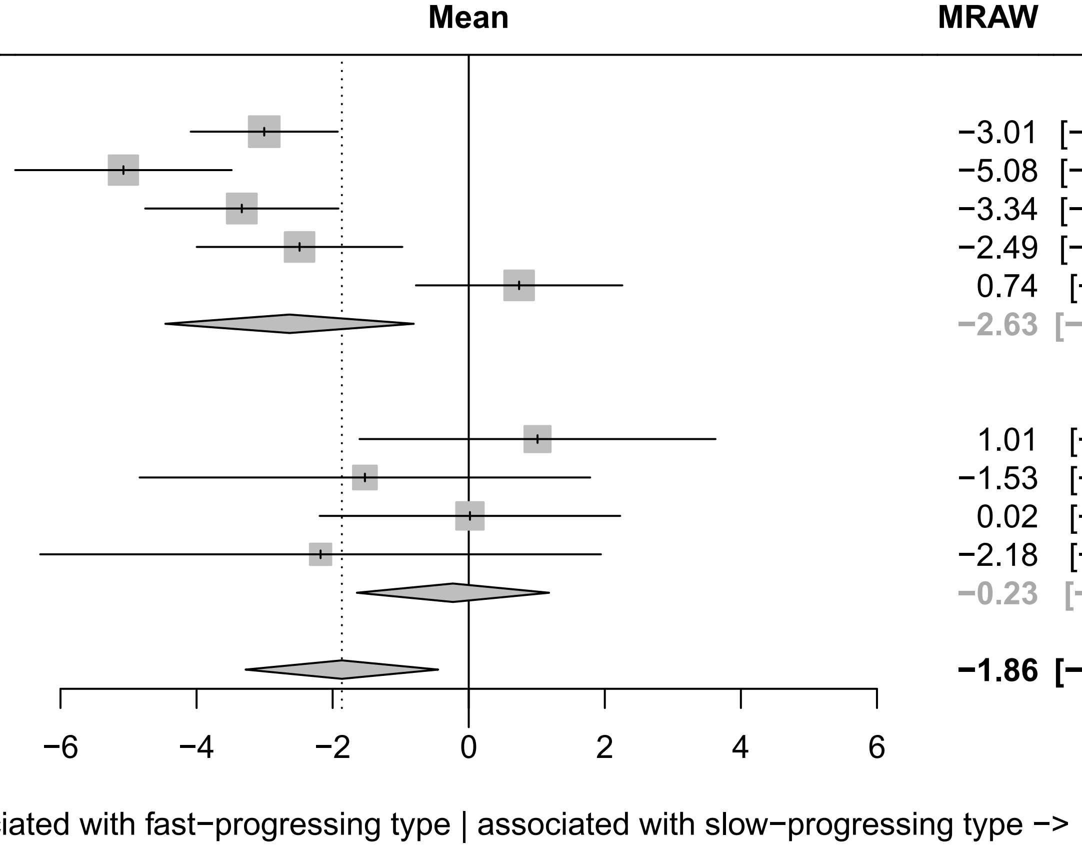

Three−level metaanalysis using random effects to calculate an overall regression coefficient estimate for Motor symptoms across cohorts. The dashed line indicates the overall mean estimate. The solid line indicates no effect.

Forest plot for baseline characteristics of symptom domain Impulsivity (validation)

Study	Total	Mean	SD	MRAW	95%−CI
**Cohort = LuxPARK (validation)**					
QUIP	211	−1.31	13.5582	−1.31	[−3.14; 0.52]
**Cohort = ICEBERG (validation)**					
QUIP−RS	154	6.31	52.0460	6.31	[−1.91; 14.53]
**Random effects model**	**365**			**1.40**	**[−5.75; 8.55]**

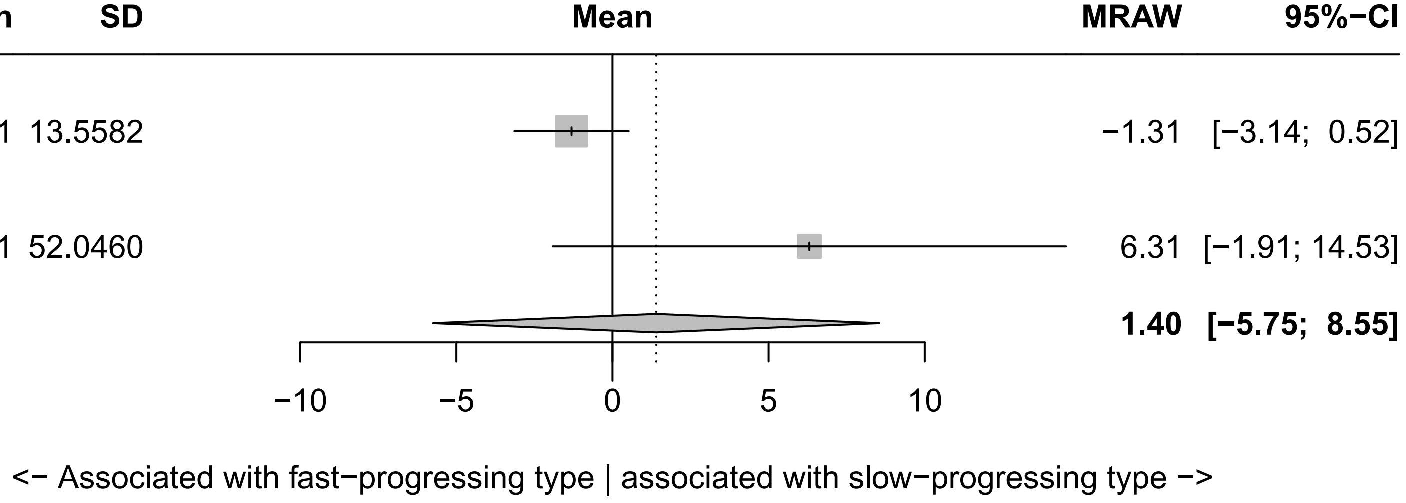

Three−level metaanalysis using random effects to calculate an overall regression coefficient estimate for Impulsivity across cohorts.
The dashed line indicates the overall mean estimate. The solid line indicates no effect.

Forest plot for baseline characteristics of symptom domain Tremor (validation)

Study	Total	Mean	SD	MRAW	95%−CI
Cohort = ICEBERG (validation)					
TD off	154	0.65	14.2244	0.65	[−1.60; 2.90]
TD on	131	0.70	12.0952	0.70	[−1.37; 2.78]
Random effects model	285			0.68	[−0.84; 2.20]
Cohort = LuxPARK (validation)					
TD on	470	1.13	15.2370	1.13	[−0.25; 2.50]
**Random effects model**	**755**			**0.93**	**[−0.10; 1.95]**

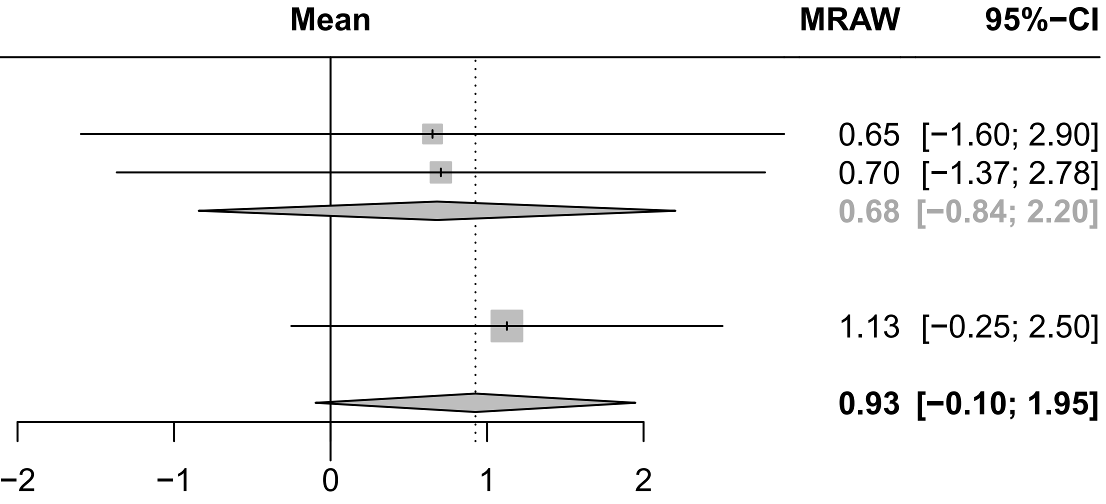

<− Associated with fast−progressing type | associated with slow−progressing type −>

Three−level metaanalysis using random effects to calculate an overall regression coefficient estimate for Tremor across cohorts.
The dashed line indicates the overall mean estimate. The solid line indicates no effect.

Forest plot for baseline characteristics of symptom domain Fatigue (validation)

Study	Total	Mean	SD	MRAW	95%−CI
**Cohort = LuxPARK (validation)**					
UPDRS I Fatigue	558	−0.47	10.5204	−0.47	[−1.35; 0.40]
**Cohort = ICEBERG (validation)**					
UPDRS I Fatigue	154	−0.59	13.2677	−0.59	[−2.68; 1.51]
**Random effects model**	**712**			**−0.49**	**[−1.30; 0.32]**

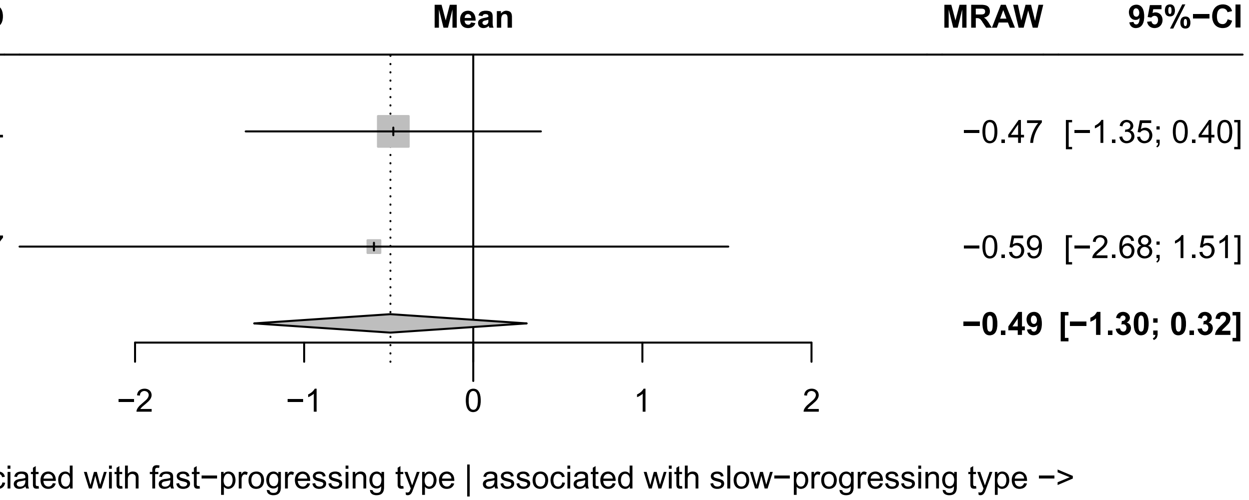

Three−level metaanalysis using random effects to calculate an overall regression coefficient estimate for Fatigue across cohorts.
The dashed line indicates the overall mean estimate. The solid line indicates no effect.

ICEBERG study group

Steering committee: Marie Vidailhet, MD, PhD, (Pitié-Salpêtrière Hospital, Paris, principal investigator of ICEBERG), Jean-Christophe Corvol, MD, PhD (Pitié-Salpêtrière Hospital, Paris, scientific lead), Isabelle Arnulf, MD, PhD (Pitié-Salpêtrière Hospital, Paris, member of the steering committee), Stéphane Lehericy, MD, PhD (Pitié-Salpêtrière Hospital, Paris, member of the steering committee);

Clinical data: Marie Vidailhet, MD, PhD, (Pitié-Salpêtrière Hospital, Paris, coordination), Graziella Mangone, MD, PhD (Pitié-Salpêtrière Hospital, Paris, co-coordination), Jean-Christophe Corvol, MD, PhD (Pitié-Salpêtrière Hospital, Paris), Isabelle Arnulf, MD, PhD (Pitié-Salpêtrière Hospital, Paris), Sara Sambin, MD (Pitié-Salpêtrière Hospital, Paris), Poornima Menon, MD (Pitié-Salpêtrière Hospital, Paris, Jonas Ihle, MD (Pitié-Salpêtrière Hospital, Paris), Caroline Weill, MD, (Pitié-Salpêtrière Hospital, Paris), David Grabli, MD, PhD (Pitié-Salpêtrière Hospital, Paris); Florence Cormier-Dequaire, MD (Pitié-Salpêtrière Hospital, Paris); Louise Laure Mariani, MD, PhD (Pitié-Salpêtrière Hospital, Paris), Bertrand Degos, MD, PhD (Avicenne Hospital, Bobigny);

Neuropsychological data: Richard Levy, MD (Pitié-Salpêtrière Hospital, Paris, coordination), Fanny Pineau, MS (Pitié-Salpêtrière Hospital, Paris, neuropsychologist), Julie Socha, MS (Pitié-Salpêtrière Hospital, Paris, neuropsychologist), Eve Benchetrit, MS (La Timone Hospital, Marseille, neuropsychologist), Virginie Czernecki, MS (Pitié-Salpêtrière Hospital, Paris, neuropsychologist), Marie-Alexandrine, MS (Pitié-Salpêtrière Hospital, Paris, neuropsychologist);

Eye movement: Sophie Rivaud-Pechoux, PhD (ICM, Paris, coordination); Elodie Hainque, MD, PhD (Pitié-Salpêtrière Hospital, Paris);

Sleep assessment: Isabelle Arnulf, MD, PhD (Pitié-Salpêtrière Hospital, Paris, coordination), Smaranda Leu Semenescu, MD (Pitié-Salpêtrière Hospital, Paris), Pauline Dodet, MD (Pitié-Salpêtrière Hospital, Paris);

Genetic data: Jean-Christophe Corvol, MD, PhD (Pitié-Salpêtrière Hospital, Paris, coordination), Graziella Mangone, MD, PhD (Pitié-Salpêtrière Hospital, Paris, co-coordination), Samir Bekadar, MS (Pitié-Salpêtrière Hospital, Paris, biostatistician), Alexis Brice, MD (ICM, Pitié-Salpêtrière Hospital, Paris), Suzanne Lesage, PhD (INSERM, ICM, Paris, genetic analyses);

Metabolomics: Fanny Mochel, MD, PhD (Pitié-Salpêtrière Hospital, Paris, coordination), Farid Ichou, PhD (ICAN, Pitié-Salpêtrière Hospital, Paris), Vincent Perlbarg, PhD, Pierre and Marie Curie University), Benoit Colsch, PhD (CEA, Saclay), Arthur Tenenhaus, PhD (Supelec, Gif-sur-Yvette, data integration);

Brain MRI data: Stéphane Lehericy, MD, PhD (Pitié-Salpêtrière Hospital, Paris, coordination), Rahul Gaurav, MS, (Pitié-Salpêtrière Hospital, Paris, data analysis), Nadya Pyatigorskaya, MD, PhD, (Pitié-Salpêtrière Hospital, Paris, data analysis); Lydia Yahia-Cherif, PhD (ICM, Paris, Biostatistics), Romain Valabregue, PhD (ICM, Paris, data analysis), Cécile Galléa, PhD (ICM, Paris);

Datscan imaging data: Marie-Odile Habert, MCU-PH (Pitié-Salpêtrière Hospital, Paris, coordination);

Voice recording: Dijana Petrovska, PhD (Telecom Sud Paris, Evry, coordination), Laetitia Jeancolas, MS (Telecom Sud Paris, Evry);

Study management: Alizé Chalançon (Pitié-Salpêtrière Hospital, Paris, Project manager), Carole Dongmo-Kenfack (Pitié-Salpêtrière Hospital, Paris, clinical research assistant); Christelle Laganot (Pitié-Salpêtrière Hospital, Paris, clinical research assistant), Valentine Maheo (Pitié-Salpêtrière Hospital, Paris, clinical research assistant), Manon Gomes (Pitié-Salpêtrière Hospital, Paris, clinical research assistant)

Study sponsoring: The ICEBERG Study was funded by the Programme d'investissements d’avenir (ANR-10-IAIHU-06), the Paris Institute of Neurosciences – IHU (IAIHU-06), the Agence Nationale de la Recherche (ANR-11-INBS-0006), and Électricité de France (Fondation d’Entreprise EDF).

Progression subtypes in Parkinson’s disease identified by a data driven multi cohort analysis

NCER-PD/LuxPARK consortium

We would like to thank all participants of the Luxembourg Parkinson’s Study for their important support to our research. Furthermore, we acknowledge the joint effort of the National Centre of Excellence in Research on Parkinson’s Disease (NCER-PD) Consortium members from the partner institutions Luxembourg Centre for Systems Biomedicine, Luxembourg Institute of Health, Centre Hospitalier de Luxembourg, and Laboratoire National de Santé generally contributing to the Luxembourg Parkinson’s Study as listed below:

Geeta ACHARYA[2], Gloria AGUAYO[2], Myriam ALEXANDRE[2], Muhammad ALI[1], Wim AMMERLANN[2], Giuseppe ARENA[1], Rudi BALLING[1], Michele BASSIS[1], Katy BEAUMONT[2], Regina BECKER[1], Camille BELLORA[2], Guy BERCHEM[3], Daniela BERG[11], Alexandre BISDORFF[5], Ibrahim BOUSSAAD[1], Kathrin BROCKMANN[11], Jessica CALMES[2], Lorieza CASTILLO[2], Gessica CONTESOTTO[2], Nico DIEDERICH[3], Rene DONDELINGER[5], Daniela ESTEVES[2], Guy FAGHERAZZI[2], Jean-Yves FERRAND[2], Manon GANTENBEIN[2], Thomas GASSER[11], Piotr GAWRON[1], Soumyabrata GHOSH[1], Marijus GIRAITIS[2,3], Enrico GLAAB[1], Elisa GÓMEZ DE LOPE[1], Jérôme GRAAS[2], Mariella GRAZIANO[17], Valentin GROUES[1], Anne GRÜNEWALD[1], Wei GU[1], Gaël HAMMOT[2], Anne-Marie HANFF[2,20,21], Linda HANSEN[1,3], Michael HENEKA[1], Estelle HENRY[2], Sylvia HERBRINK[6], Sascha HERZINGER[1], Michael HEYMANN[2], Michele HU[8], Alexander HUNDT[2], Nadine JACOBY[18], Jacek JAROSLAW LEBIODA[1], Yohan JAROSZ[1], Sonja JÓNSDÓTTIR[2], Quentin KLOPFENSTEIN[1], Jochen KLUCKEN[1,2,3], Rejko KRÜGER[1,2,3], Pauline LAMBERT[2], Zied LANDOULSI[1], Roseline LENTZ[7], Inga LIEPELT[11], Robert LISZKA[14], Laura LONGHINO[3], Victoria LORENTZ[2], Paula Cristina LUPU[2], Tainá M. MARQUES[1], Clare MACKAY[10], Walter MAETZLER[15], Katrin MARCUS[13], Guilherme MARQUES[2], Patricia MARTINS CONDE[1], Patrick MAY[1], Deborah MCINTYRE[2], Chouaib MEDIOUNI[2], Francoise MEISCH[1], Myriam MENSTER[2], Maura MINELLI[2], Michel MITTELBRONN[1,4], Brit MOLLENHAUER[12], Friedrich MÜHLSCHLEGEL[4], Romain NATI[3], Ulf NEHRBASS[2], Sarah NICKELS[1], Beatrice NICOLAI[3], Jean-Paul NICOLAY[19], Fozia NOOR[2], Marek OSTASZEWSKI[1], Clarissa P. C. GOMES[1], Sinthuja PACHCHEK[1], Claire PAULY[1,3], Laure PAULY[2,20], Lukas PAVELKA[1,3], Magali PERQUIN[2], Nancy E. RAMIA[1,] Rosalina RAMOS LIMA[2], Armin RAUSCHENBERGER[1], Rajesh RAWAL[1], Dheeraj REDDY BOBBILI[1], Kirsten ROOMP[1], Eduardo ROSALES[2], Isabel ROSETY[1], Estelle SANDT[2], Stefano SAPIENZA[1], Venkata SATAGOPAM[1], Margaux SCHMITT[2], Sabine SCHMITZ[1], Reinhard SCHNEIDER[1], Jens SCHWAMBORN[1], Amir SHARIFY[2], Ekaterina SOBOLEVA[1], Kate SOKOLOWSKA[2], Hermann THIEN[2], Elodie THIRY[3], Rebecca TING JIIN LOO[1], Christophe TREFOIS[1], Johanna TROUET[2], Olena TSURKALENKO[2], Michel VAILLANT[2], Mesele VALENTI[2], Gilles VAN CUTSEM[1,3], Carlos VEGA[1], Liliana VILAS BOAS[3], Maharshi VYAS[1], Richard WADE-MARTINS[9], Paul WILMES[1], Evi WOLLSCHEID-LENGELING[1], Gelani ZELIMKHANOV[3]

1. Luxembourg Centre for Systems Biomedicine, University of Luxembourg, Esch-sur-Alzette, Luxembourg
2. Luxembourg Institute of Health, Strassen, Luxembourg
3. Centre Hospitalier de Luxembourg, Strassen, Luxembourg
4. Laboratoire National de Santé, Dudelange, Luxembourg
5. Centre Hospitalier Emile Mayrisch, Esch-sur-Alzette, Luxembourg
6. Centre Hospitalier du Nord, Ettelbrück, Luxembourg
7. Parkinson Luxembourg Association, Leudelange, Luxembourg
8. Oxford Parkinson's Disease Centre, Nuffield Department of Clinical Neurosciences, University of Oxford, Oxford, UK
9. Oxford Parkinson's Disease Centre, Department of Physiology, Anatomy and Genetics, University of Oxford, South Parks Road, Oxford, UK
10. Oxford Centre for Human Brain Activity, Wellcome Centre for Integrative Neuroimaging, Department of Psychiatry, University of Oxford, Oxford, UK
11. Center of Neurology and Hertie Institute for Clinical Brain Research, Department of Neurodegenerative Diseases, University Hospital Tübingen, Germany
12. Paracelsus-Elena-Klinik, Kassel, Germany
13. Ruhr-University of Bochum, Bochum, Germany
14. Westpfalz-Klinikum GmbH, Kaiserslautern, Germany
15. Department of Neurology, University Medical Center Schleswig-Holstein, Kiel, Germany

16. Department of Neurology Philipps, University Marburg, Marburg, Germany
17. Association of Physiotherapists in Parkinson's Disease Europe, Esch-sur-Alzette, Luxembourg
18. Private practice, Ettelbruck, Luxembourg
19. Private practice, Luxembourg, Luxembourg
20. Faculty of Science, Technology and Medicine, University of Luxembourg, Esch-sur-Alzette, Luxembourg
21. Department of Epidemiology, CAPHRI School for Public Health and Primary Care, Maastricht University Medical Centre+, Maastricht, the Netherlands
