# Supplementary material for: Progression subtypes in Parkinson’s disease identified by a data-driven multi cohort analysis
Source: NPJ Parkinsons Dis. 2024 May 2;10:95. doi: 10.1038/s41531-024-00712-3 (PMC11066039; doi:10.1038/s41531-024-00712-3)
Supplement: Supplementary file 2 — Reporting summary [file 41531_2024_712_MOESM2_ESM.pdf]

nature portfolio

Corresponding author(s): Tom Hähnel

Last updated by author(s): Jan 22, 2024

# Reporting Summary

Nature Portfolio wishes to improve the reproducibility of the work that we publish. This form provides structure for consistency and transparency in reporting. For further information on Nature Portfolio policies, see our Editorial Policies and the Editorial Policy Checklist.

## Statistics

For all statistical analyses, confirm that the following items are present in the figure legend, table legend, main text, or Methods section.

| n/a | Confirmed | |
|---|---|---|
| ☐ | ☒ | The exact sample size (*n*) for each experimental group/condition, given as a discrete number and unit of measurement |
| ☐ | ☒ | A statement on whether measurements were taken from distinct samples or whether the same sample was measured repeatedly |
| ☐ | ☒ | The statistical test(s) used AND whether they are one- or two-sided<br>*Only common tests should be described solely by name; describe more complex techniques in the Methods section.* |
| ☐ | ☒ | A description of all covariates tested |
| ☐ | ☒ | A description of any assumptions or corrections, such as tests of normality and adjustment for multiple comparisons |
| ☐ | ☒ | A full description of the statistical parameters including central tendency (e.g. means) or other basic estimates (e.g. regression coefficient) AND variation (e.g. standard deviation) or associated estimates of uncertainty (e.g. confidence intervals) |
| ☒ | ☐ | For null hypothesis testing, the test statistic (e.g. $F$, $t$, $r$) with confidence intervals, effect sizes, degrees of freedom and $P$ value noted<br>*Give P values as exact values whenever suitable.* |
| ☒ | ☐ | For Bayesian analysis, information on the choice of priors and Markov chain Monte Carlo settings |
| ☒ | ☐ | For hierarchical and complex designs, identification of the appropriate level for tests and full reporting of outcomes |
| ☐ | ☒ | Estimates of effect sizes (e.g. Cohen's $d$, Pearson's $r$), indicating how they were calculated |

*Our web collection on statistics for biologists contains articles on many of the points above.*

## Software and code

Policy information about availability of computer code

| | |
|---|---|
| Data collection | This study is a retrospective analysis of multiple clinical datasets. Data collection has been performed by the individual study groups (PPMI: www.ppmi-info.org, ICEBERG: marie.vidailhet@psl.aphp.fr, LuxPARK: rejko.krueger@uni.lu) and requires individual requests for data access. |
| Data analysis | The following software and packages were used for analysis: Python (Version 3.10), R (Version 4.3), R-packages: LTJMM (Version 1.2), rstan (2.21.8), lme4 (1.1-33), ordinal (2022.11-26), meta (6.5-0), longpower (1.0.24); Python packages: Vader (0.0.1), pingouin (0.5.4), factor-analyzer (0.5.0), statsmodels (0.13.5), scikit-learn (1.1.3), scipy (1.9.3) and lifelines (0.28.0).The underlying code used to train LTJMM, VaDER and all relevant statistical analyses for this study will be published on github under the MIT licence upon acceptance of the paper and can be accessed via https://github.com/t-haehnel/PD-Progression-Subtypes.The LTJMM package can be downloaded from github (https://github.com/mcdonohue/rstanarm) and was first published in "Bayesian latent time joint mixed effect models for multicohort longitudinal data" (Li et al., 2017). The VaDER package can be downloaded from github (https://github.com/yalchik/VaDER) and was first published in "Deep learning for clustering of multivariate clinical patient trajectories with missing values" (de Jong, 2019). |

For manuscripts utilizing custom algorithms or software that are central to the research but not yet described in published literature, software must be made available to editors and reviewers. We strongly encourage code deposition in a community repository (e.g. GitHub). See the Nature Portfolio guidelines for submitting code & software for further information.

# Data

Policy information about availability of data

All manuscripts must include a data availability statement. This statement should provide the following information, where applicable:

- Accession codes, unique identifiers, or web links for publicly available datasets
- A description of any restrictions on data availability
- For clinical datasets or third party data, please ensure that the statement adheres to our policy

As this is a retrospective analysis, the availability of clinical data depends on the individual study groups (PPMI: www.ppmi-info.org, ICEBERG: marie.vidailhet@psl.aphp.fr, LuxPARK: rejko.krueger@uni.lu) and requires individual requests for data access.

# Research involving human participants, their data, or biological material

Policy information about studies with human participants or human data. See also policy information about sex, gender (identity/presentation), and sexual orientation and race, ethnicity and racism.

| | |
|---|---|
| Reporting on sex and gender | The term sex was used to refer to the biological definition. Sex was reported on the basis of self-report by people with PD. Gender information was not collected by the individual study groups. We corrected for potential sex differences in the LTJMM model by using sex as a covariate. No sex differences were found between Parkinson's disease progression subtypes. The number of female and male PD patients included was as follows:<br>PPMI: 273 male, 136 female<br>ICEBERG: 96 male, 58 female<br>LuxPARK: 379 male, 182 female |
| Reporting on race, ethnicity, or other socially relevant groupings | The PPMI dataset included 377 White, 8 Asian, 6 Black and 18 Other (unspecified in the dataset) people with Parkinson's disease. ICEBERG included 137 Caucasian, 9 North African, 4 Other, 2 Black African, 1 West Indian and 1 Asian people with Parkinson's disease. No ethnicity was provided in the LuxPARK data set. Ethnicity information was self-reported by people with Parkinson's disease. |
| Population characteristics | The median age [interquartile range] of the three cohorts was 63.0 years [55.2-69.3] for PPMI, 63.7 years [57.1-69.4] for ICEBERG and 67.8 years [59.4-73.1] for LuxPARK. The sex distribution is reported in the fields above. More demographic details are outlined in the manuscript. |
| Recruitment | Recriutment was the subject of the individual study groups.<br>PPMI: 409 people with Parkinson's Disease (PwPD) from the publicly available Parkinson's Progression Markers Initiative (PPMI) with clinical visits between 2011 and 2020 were analyzed. All PwPD had a clinical diagnosis of Parkinson's Disease (PD) and a pathological dopamine transporter SPECT (DaTSCAN).<br>ICEBERG: 154 PwPD from the ICEBERG cohort study (NCT02305147), an ongoing four-year observational study of PwPD with recent onset of PD conducted at the Paris Brain Institute (Institut du Cerveau-ICM, Pitié-Salpêtrière Hospital, Paris, France), were analyzed. Visits were performed between 2014 and 2022. PD was diagnosed according to UK Parkinson's Disease Society Brain Bank criteria and PwPD with DaTSCANs showing no dopaminergic deficit were excluded. Inclusion was restricted to disease onset not more than three years before baseline visit.<br>LuxPARK: 561 PwPD from the LuxPARK trial, an ongoing observational study of all disease stages PwPD from Luxembourg and the Greater Region with up to four years follow up, were analyzed. Visits were performed between 2015 and 2022. PD was diagnosed according to UK Parkinson's Disease Society Brain Bank criteria. |
| Ethics oversight | The PPMI project was approved by the Institutional Review Board or Independent Ethics Committee of all participating sites in Europe, including Attikon University Hospital (Greece), Hospital Clinic de Barcelona and Hospital Universitario Donostia (Spain), Innsbruck University (Austria), Paracelsus-Elena-Klinic Kassel/University of Marburg (Germany), Imperial College London (UK), Pitié-Salpêtrière Hospital (France), University of Salerno (Italy), and in the USA, including Emory University, Johns Hopkins University, University of Alabama at Birmingham, PD and Movement Disorders Center of Boca Raton, Boston University, Northwestern University, University of Cincinnati, Cleveland Clinic Foundation, Baylor College of Medicine, Institute for Neurodegenerative Disorders, Columbia University Medical Center, Beth Israel Medical Center, University of Pennsylvania, Oregon Health and Science University, University of Rochester, University of California at San Diego, and University of California, San Francisco. Informed consent was provided according to the Declaration of Helsinki.<br>ICEBERG received approval from the local ethical committee (IRBParis VI, RCB: 2014-A00725-42).<br>LuxPARK was approved by the National Ethics Board in Luxembourg (CNER Ref: 201407/13). |

Note that full information on the approval of the study protocol must also be provided in the manuscript.

# Field-specific reporting

Please select the one below that is the best fit for your research. If you are not sure, read the appropriate sections before making your selection.

☒ Life sciences ☐ Behavioural & social sciences ☐ Ecological, evolutionary & environmental sciences

For a reference copy of the document with all sections, see nature.com/documents/nr-reporting-summary-flat.pdf

# Life sciences study design

All studies must disclose on these points even when the disclosure is negative.

| | |
|---|---|
| Sample size | PPMI: 409<br>ICEBERG: 154<br>LuxPARK: 561<br>The sample size was not directly determined for our progression subtype analysis because this was a retrospective analysis and recruitment was done by the individual study groups. In total, we included over 1,100 people with Parkinson's disease in our analysis. Therefore, this is one of the largest studies on Parkinson's disease progression subtype identification. |
| Data exclusions | PPMI: We restricted our analysis to untreated de-novo people with Parkinson's disease (PwPD) in PPMI. Therefore, we included only PwPD with clinical diagnosis not more than two years before baseline visit, Hoehn & Yahr stage 0-2 and no dopaminergic treatment at baseline visit. We further restricted our analysis to PwPD with age > 30 years and at least one additional visit as we require longitudinal information.<br>ICEBERG: PwPD with DaTSCANs showing no dopaminergic deficit were excluded by the study group. Furthermore, inclusion was restricted to disease onset not more than three years before baseline visit. We further restricted our analysis to PwPD with at least two visits as we require longitudinal information.<br>LuxPARK: We restricted our analysis to PwPD with at least two visits as we require longitudinal information. |
| Replication | We replicated our findings in three cohorts (PPMI, ICEBERG, LuxPARK). |
| Randomization | PPMI, ICEBERG, and LuxPARK are observational studies. Therefore, there are no different study arms and no randomization. |
| Blinding | PPMI, ICEBERG and LuxPARK are observational studies. Thus, there are no different study arms which could be blinded. |

# Reporting for specific materials, systems and methods

We require information from authors about some types of materials, experimental systems and methods used in many studies. Here, indicate whether each material, system or method listed is relevant to your study. If you are not sure if a list item applies to your research, read the appropriate section before selecting a response.

## Materials & experimental systems

| n/a | Involved in the study |
|---|---|
| ☒ | ☐ Antibodies |
| ☒ | ☐ Eukaryotic cell lines |
| ☒ | ☐ Palaeontology and archaeology |
| ☒ | ☐ Animals and other organisms |
| ☒ | ☐ Clinical data |
| ☒ | ☐ Dual use research of concern |
| ☒ | ☐ Plants |

## Methods

| n/a | Involved in the study |
|---|---|
| ☒ | ☐ ChIP-seq |
| ☒ | ☐ Flow cytometry |
| ☒ | ☐ MRI-based neuroimaging |

## Plants

| | |
|---|---|
| Seed stocks | n/a because no plants were used |
| Novel plant genotypes | n/a because no plants were used |
| Authentication | n/a because no plants were used |
